# Individual repeatability of avian migration phenology: A systematic review and meta‐analysis

**DOI:** 10.1111/1365-2656.13697

**Published:** 2022-04-18

**Authors:** Kirsty A. Franklin, Malcolm A. C. Nicoll, Simon J. Butler, Ken Norris, Norman Ratcliffe, Shinichi Nakagawa, Jennifer A. Gill

**Affiliations:** ^1^ School of Biological Sciences University of East Anglia Norwich UK; ^2^ Institute of Zoology Zoological Society of London London UK; ^3^ Natural History Museum London UK; ^4^ British Antarctic Survey Cambridge UK; ^5^ Ecology & Evolution Research Centre, School of Biological, Earth and Environmental Sciences The University of New South Wales Sydney NSW Australia

**Keywords:** annual cycle, bird migration, consistent individual differences, individual variation, intraclass correlation coefficient, timing

## Abstract

1. Changes in phenology and distribution are being widely reported for many migratory species in response to shifting environmental conditions. Understanding these changes and the situations in which they occur can be aided by understanding consistent individual differences in phenology and distribution and the situations in which consistency varies in strength or detectability.
2. Studies tracking the same individuals over consecutive years are increasingly reporting migratory timings to be a repeatable trait, suggesting that flexible individual responses to environmental conditions may contribute little to population‐level changes in phenology and distribution. However, how this varies across species and sexes, across the annual cycle and in relation to study (tracking method, study design) and/or ecosystem characteristics is not yet clear.
3. Here, we take advantage of the growing number of publications in movement ecology to perform a phylogenetic multilevel meta‐analysis of repeatability estimates for avian migratory timings to investigate these questions. Of 2,433 reviewed studies, 54 contained suitable information for meta‐analysis, resulting in 177 effect sizes from 47 species.
4. Individual repeatability of avian migratory timings averaged 0.414 (95% confidence interval: 0.3–0.5) across landbirds, waterbirds and seabirds, suggesting consistent individual differences in migratory timings is a common feature of migratory systems. Timing of departure from the non‐breeding grounds was more repeatable than timings of arrival at or departure from breeding grounds, suggesting that conditions encountered on migratory journeys and outcome of breeding attempts can influence individual variation.
5. Population‐level shifts in phenology could arise through individual timings changing with environmental conditions and/or through shifts in the numbers of individuals with different timings. Our findings suggest that, in addition to identifying the conditions associated with individual variation in phenology, exploring the causes of between‐individual variation will be key in predicting future rates and directions of changes in migratory timings. We therefore encourage researchers to report the within‐ and between‐ individual variance components underpinning the reported repeatability estimates to aid interpretation of migration behaviour. In addition, the lack of studies in the tropics means that levels of repeatability in less strongly seasonal environments are not yet clear.

## Graphical abstract

Repeated tracking of individuals on migratory journeys reveals that there are consistent individual differences in the timing of birds arriving and departing across the annual cycle. Identifying the conditions associated with individual variation in phenology and exploring the causes of between‐individual variation will be key in predicting future rates and directions of changes in migratory timings.

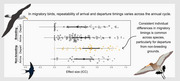

## 1 INTRODUCTION

Rapid environmental change is having profound impacts on the distribution, abundance, behaviour and interactions of species (Walther et al., 2002). For migratory species, identifying and ultimately tackling the problems caused by environmental change are particularly difficult because of the range of sites and conditions experienced by individuals across the annual cycle (Alves et al., 2013; Gilroy et al., 2016; Knudsen et al., 2011). Therefore, changes in conditions across all or part of migratory ranges could have strong implications in terms of survival rates and population dynamics at local and global scales (Newton, 2004), raising concerns regarding the effectiveness of existing protected area networks (Hanson et al., 2020; Méndez et al., 2017). The complexity and unpredictability of how migratory systems respond to environmental change represents a major challenge for conservation planners.

Changes in migratory behaviour in response to climate change have been documented in many species (Ambrosini et al., 2019). The most frequent responses are shifts in phenology in parallel with climate warming, for example migrant arrival dates at the breeding grounds in spring are getting earlier in many species (Gordo, 2007; Gunnarsson & Tómasson, 2011; Lawrence et al., 2022). In some species, shifts in migratory routes and wintering destinations (Dias et al., 2011; Sutherland, 1998) or reduced propensity for migration have been recorded, such that part or all of a population has become resident (Chapman et al., 2011; van Vliet et al., 2009). Migratory species currently showing little or no phenological change are more likely to be those experiencing population declines (Gilroy et al., 2016; Møller et al., 2008; Newton, 2008), possibly arising from a reduction in synchrony with the phenology of prey abundance (known as trophic mismatch; Thackery et al., 2010). Therefore, identifying the mechanisms through which shifts in migratory routes and/or timings occur may be key to mitigating the effects of rapid environmental change on declining migratory species (Gill et al., 2019; Knudsen et al., 2011).

In migratory systems, there are two processes that could lead to shifts in migration routes and/or timings: (a) behavioural flexibility, whereby individuals adjust their migratory behaviour according to the environmental conditions they experience (Charmantier & Gienapp, 2014) and (b) generational change, whereby the proportion of new recruits using particular locations or schedules differs from previous generations, as a result of changes in the conditions influencing those behaviours and/or the associated survival rates (Gill et al., 2014; Gill et al., 2019; Verhoeven et al., 2018). The rate and direction of shifts in migratory routes and/or timings could vary greatly with each mechanism, with behavioural flexibility facilitating relatively rapid and, potentially, directional change. By contrast, generational change would likely result in slower changes, especially for long‐lived species, as the direction and magnitude of change depends on the number of annual recruits in a population, the proportion of those experiencing different conditions that influence individual routes and phenologies, and their subsequent survival rates (Gill et al., 2019).

A key first step towards assessing the likelihood of migratory routes and timings altering in response to environmental changes is therefore quantifying when individuals show consistent differences in these behaviours. This requires repeated measurements from individuals across years to assess the amount of variation in behaviour attributable to differences among individuals. In animal movement studies, this individual‐based approach has become increasingly possible due to recent advances in remote‐tracking technology (Geen et al., 2019; López‐López, 2016), primarily satellite telemetry and more recently through light‐level geolocators (GLS). Before this, most studies of migratory behaviour have been conducted by means of visual observations or, more specifically for birds, through ringing studies (e.g. Møller, 2001; Potti, 1998; Rees, 1989). Repeated tracking of multiple individuals over multiple years can allow estimation of the variation in migratory behaviours that is explained by between‐individual variation relative to both between‐ and within‐individual variation (and measurement error; termed ‘repeatability’ (*R*) or the ‘intra‐class correlation coefficient’ (ICC; Nakagawa & Schielzeth, 2010)). High repeatability estimates could indicate a consistent behaviour within individuals relative to high variation between individuals (Lessells & Boag, 1987; Nakagawa & Schielzeth, 2010; but see Cleasby et al., 2015; Sánchez‐Tójar et al., 2022). For example, changes in phenology have long been assumed to be caused by within‐individual effects, but between‐individual effects could also contribute to changes, making it key that we understand the contributions of within‐ and between‐individual variation to repeatability estimates and interpretation.

Repeatability in migratory behaviour has been explored across taxa, including amphibians (Semlitsch et al., 1993), insects (Kent & Rankin, 2001), fishes (Brodersen et al., 2014; Thorsteinsson et al., 2012; Villegas‐Ríos et al., 2017), bats (Lehnert et al., 2018), ungulates (Laforge et al., 2021), sea turtles (Schofield et al., 2010) and birds (see Table S1). Previous meta‐analyses of behavioural repeatability have extracted repeatability estimates for migratory behaviours (Bell et al., 2009; Holtmann et al., 2017) but many possible sources of variation in levels of repeatability have not yet been explored. For example, in addition to variation as a result of different sampling designs and/or between sexes (Bell et al., 2009; Holtmann et al., 2017), repeatability may vary with tracking method, species and/or among different stages of the annual cycle. Differences in sampling strategies (e.g. number of individuals tracked, number of observations per individual) can influence estimates of repeatability (Dingemanse & Dochtermann, 2013; Wolak et al., 2012). An increase in both individual‐ and population‐level variation in migratory behaviours might be expected if individuals are tracked for longer (e.g. Berthold et al., 2004; Catry et al., 1999), and variability may be underestimated if sample sizes are small, as estimates will be less likely to capture the total population variation (Conklin et al., 2013).

Repeatability may also be affected by the methods used to track individuals. The earliest estimates of repeatability in avian migration used conventional ringing methods such as ring recaptures, and colour‐ring re‐sightings, which have the advantages that they last for most or all of marked individuals' lifetime, and are much cheaper, allowing samples of hundreds and even thousands of individuals. These Eulerian sampling methods (i.e. fixed in space) rely on re‐capturing the marked birds (and recovery rates are generally low) or depend highly on the spatiotemporal distribution of observers. Detection of individuals with this method may be incomplete, which may introduce variable lags in observation of the timing of migratory arrivals and/or departures. Lagrangian tracking of individuals through time and space (i.e. animal‐borne tracking devices) may therefore be more suited to studies of the timing of individual movements (Phillips et al., 2019). For example, the accuracy of estimates of timing of arrival at the breeding grounds as observed through conventional studies may be low in comparison to more recent methods, such as satellite telemetry, GPS and GLS (Korner‐Nievergelt et al., 2012). The general trade‐offs between these methods therefore include temporal and spatial resolution, life span and the mass and cost of each unit (Wakefield et al., 2009). Satellite and GPS loggers have good temporal (e.g. on a minute or hourly basis) and spatial accuracy (within ~150 m and 10 m, respectively) but until recently their mass restricted them to species of larger body size (Hobson et al., 2019). In contrast, GLS have low power requirements, allowing the devices to be considerably lighter (<1 g; Bridge et al., 2011), and are relatively cheap but provide only two locations per day with varying levels of spatial inaccuracy (Halpin et al., 2021; Phillips et al., 2004).

Repeatability values of migration parameters may also vary across the annual cycle. For example, we might expect the pre‐breeding stages of migratory species to be more time‐sensitive than post‐breeding stages (Alerstam et al., 2003; McNamara et al., 1998). Repeatability in timing of arrival at breeding grounds has been demonstrated for several species (e.g. Conklin et al., 2013; Krietsch et al., 2017; Stanley et al., 2012), and may be related to the benefits of synchronous arrival times with mates (Gunnarsson et al., 2004; Morrison et al., 2019), and/or to exploiting consistently timed local resource peaks (Alerstam et al., 2003). Familiarity with conditions at a certain location and time may improve chances of survival and breeding success compared to using a different site, or the same site at a different time (McNamara & Dall, 2010; Shimada et al., 2019). By contrast, timing of other stages (e.g. departure from breeding ground) may be less time sensitive, but constraints may still exist if carryover effects influence performance later in the annual cycle (Stutchbury et al., 2011).

In bird migration studies, repeatability has become standard for describing consistent individual differences in migratory behaviour. These studies are increasingly reporting high repeatability in migratory timings, but how repeatability varies across the annual cycle and in relation to study and/or ecosystem characteristics is not yet clear. To address these issues, we performed a systematic review and phylogenetic multilevel meta‐analysis to synthesise the current literature and quantitatively assess the repeatability of avian migratory timings and possible sources of variation in repeatability estimates. We focus on the following five questions: Does repeatability vary (a) across the annual cycle, (b) with tracking method, (c) across ecological groups (seabirds, landbirds and waterbirds; Geen et al., 2019), (d) between males and females, and (e) with the number of observations per individual?

## 2 MATERIALS AND METHODS

### 2.1 Literature search

We aimed to conduct a comprehensive search for studies estimating repeatability of temporal parameters of avian migration using a combination of approaches. We focused on arrival at, and departure from, breeding and non‐breeding grounds. First, we performed a systematic search for published studies using the Web of Science and Scopus online databases on 1st June 2021. Second, we consulted a recently published meta‐analysis of hormonal, metabolic and behavioural repeatability in birds (Holtmann et al., 2017), which included repeatability estimates of migration. We manually checked each entry from those sources to confirm suitability for our purposes and extracted additional moderator variables to be used in our analyses (see below). Finally, to add to—and validate the accuracy of—the results of the literature search, we searched the reference lists of papers already in our accepted reference library. The details of these search strategies and the Boolean search strings used are presented in our Supporting Information, along with a flow diagram (often referred to as a PRISMA flow chart—the Preferred Reporting Items in Systematic Reviews and Meta‐Analyses; Moher et al., 2009; O'Dea et al., 2021; Figure S1) which shows the stages at which studies were disqualified or eventually used in the current study.

### 2.2 Inclusion and exclusion criteria

To be included in our analyses, observational studies needed to adhere to five main criteria. First, studies had to report repeatability estimates in the form of intraclass correlation coefficients (ICC) using an ANOVA based (Lessells & Boag, 1987) or Linear Mixed Model (LMM)‐based approach (Nakagawa & Schielzeth, 2010), or a Spearman/Pearson correlation coefficient (*r*; cf. Barbosa & Morrissey, 2021). If both ICC and *r* estimates were reported using the same data, we only included the ICC estimates in our data as this was the most commonly reported (>90%) repeatability metric in our dataset. Second, studies which calculated repeatability using dates when certain latitudes were crossed were excluded unless they were explicitly stated as the arrival or departure dates for the species. We relied on authors' descriptions as to what determines arrival at/departure from the breeding and non‐breeding grounds. Third, we restricted all datasets to breeding adults only. We used this criterion because the refinement of migratory behaviour has shown to be a progressive process mediated by age and experience, particularly for long‐lived species (Campioni et al., 2019). Fourth, only English‐language studies were included. Finally, in addition to repeatability estimates, studies also needed to report sample sizes, and moderator variables were extracted where reported and included in our analyses (see below). Where any of the repeatability estimates or sample size data were missing, we attempted to contact authors (*n* = 2 studies) for this information. One author replied but was unable to provide the requested data, and so neither of these studies was included.

### 2.3 Study selection

The exact number of screened and included studies are shown in Figure S1, and a list of all studies included in the analyses can be found in the Data sources section. We used Rayyan software to screen titles and abstracts (Ouzzani et al., 2016). One person (KAF) screened the abstracts, using a decision tree (Figure S2). Approximately 93% of the 2,433 abstracts were excluded after screening. We performed full‐text screening for the remaining 160 papers included after abstract screening, from which 47 were included for data extraction. After searching the reference lists of these papers accepted for data extraction, we found an additional six suitable for our analyses, and included two repeatability estimates from our own paper (Franklin, Norris, et al., 2022), providing a total of 54 papers.

### 2.4 Data collection

Data were extracted from text, tables or figures. To extract data from figures, we used WebPlotDigitizer software (Rohatgi, 2015). All data were extracted by one author (KAF). In addition to the repeatability estimates (*r* or ICC) from each study, we also extracted the following moderator variables: the annual event for which repeatability was estimated (arrival at, or departure from, breeding or non‐breeding grounds), the method used to track individuals, the coordinates of tagging, and whether this was on the breeding or non‐breeding grounds, study species, sex (male, female, mixed/unknown), the number of individuals (*n*), the mean number of observations per individual (*k*) and year of publication. For studies that did not state *k* but reported the total number of observations, we calculated *k* by dividing the number of observations by the number of individuals. The methods used to track individuals were grouped into three categories, which represent the type of sampling method (Eulerian or Lagrangian) and the spatial and temporal accuracy of the method: (a) conventional (bird ringing, colour‐ringing); (b) geolocation (geolocators); and (c) GPS (GPS, satellite, PTTs, radio‐telemetry). If studies used >1 type of tracking method on different groups of individuals, we included both repeatability estimates. Finally, we recorded the statistic that was used to report repeatability (ICC or *r*), whether any fixed or random effects (in addition to individual as random effect) were included when calculating repeatability (i.e. agreement vs. adjusted repeatability; Nakagawa & Schielzeth, 2010), and whether those calculating (ANOVA based or LMM based) repeatability reported the unstandardised variance components. The full list of moderators is found in our Supporting Information.

### 2.5 Data analysis

Studies included in our dataset varied in sample size, number of samples per individual and in how repeatability was estimated. Thus, it was important to weight studies appropriately and to convert reported repeatabilities to a comparable statistic. We therefore converted all repeatability estimates (ICC and *r*) to the standardised effect size Fisher's *Z* (*Zr*) along with the corresponding sampling variance for each study (as described in Holtmann et al., 2017 and McGraw & Wong, 1996). As correlation‐ and ANOVA‐based repeatabilities can produce negative values, often reflecting noise around a statistical zero (Nakagawa & Schielzeth, 2010), we set the negative repeatability estimates/*Zr* values in our dataset (*n* = 13) to zero for our analyses. We used these *Zr* values and sampling variances (see below) in all meta‐analytical models, but when plotting and reporting parameter estimates we back‐transformed effect sizes to ICC to aid interpretation. The results of all the meta‐analytic and meta‐regression models when including the negative repeatability estimates are reported in the Supporting Information (Tables S12–19).

### 2.6 Meta‐analysis

We fit meta‐analytic and meta‐regression multilevel linear mixed‐effects models, using the rma.mv function in the metafor package (v. 3.0.2; Viechtbauer, 2010) in r (v. 3.6.2; R Core Team, 2019). Our data contained multiple levels and different types of non‐independence (Noble et al., 2017). We partially accounted for this non‐independence with random‐effects and sampling variance–covariance matrices.

All models included the following random effects: (a) paper ID, which encompasses multiple effect sizes extracted from the same paper, (b) cohort ID, which encompasses multiple effect sizes obtained from the same group of birds within the same paper, (c) species ID, which encompasses multiple effect sizes from the same species across papers, and (d) effect ID, which is a unit‐level random effect representing residual/within‐study variance. In addition to species ID (a non‐phylogenetic measure), we also included (e) phylogeny (modelled with a phylogenetic relatedness correlation matrix), to account for species similarities due to evolutionary history (Cinar et al., 2022). To generate the phylogeny, we used a phylogenetic tree from Jetz et al. (2012), provided by Holtmann et al. (2017) and prepared on the basis of Hackett backbone (Hackett tree; Hackett et al., 2008). After trimming the tree using the species names in our dataset, we computed branch lengths using Grafen's method (Grafen, 1989) in the compute.brlen function in the r package ape (v. 5.5; Paradis & Schliep, 2019). For the final phylogenetic tree, see Figure S3.

Multiple repeatability estimates were measured on the same animals within a paper (cohort ID) which induces a correlation between sampling error variances (Noble et al., 2017). Thus, we constructed variance–covariance matrices to model shared sampling error for effect sizes from the same cohort, assuming a 0.5 correlation (Noble et al., 2017). We also ran the phylogenetic meta‐analytic model assuming a 0.25 and 0.75 correlation between estimates from the same cohort. All three correlations yielded qualitatively similar results; thus, we assume a 0.5 correlation throughout, and present the results for the other correlation values in the ‘Sensitivity Analysis’ section in our Supporting Information (Table S11).

A multilevel intercept‐only meta‐analytic model was fitted to estimate the overall mean of the effect sizes with the random effects listed above. To evaluate the effects of moderators, we ran a univariate multilevel meta‐regression model for each of the following: (a) tracking method, (b) ecological group, (c) sex, (d) annual event and (e) *k*, the number of observations per individual. Interaction terms were not included between ecological group and (a) method or (b) annual event, due to insufficient sample sizes of certain levels of categorical variables.

For meta‐analytic models, we quantified a multilevel version of the ‘heterogeneity’ measures (*I* ^2^), which indicate the amount of variance unexplained after controlling for sampling variance (Higgins & Thompson, 2002; Nakagawa & Santos, 2012) while, for meta‐regression, we estimated the percentage of heterogeneity explained by the moderators using marginal *R* ^2^ (Nakagawa & Schielzeth, 2013) using the function ‘r2_ml’ in the r package orchard v.0.0.0.9000 (Nakagawa et al., 2021). Missing and unreported data were not included in the meta‐regressions (i.e. we ran complete‐case analyses). Results of the main effect model and meta‐regressions with categorical moderators were graphically represented as orchard plots using code adapted from the r package orchard.

All model specifications, model selection procedures and associated coding are provided in our Supporting Information. We followed reporting guidelines outlined in the PRISMA‐EcoEvo checklist for this study (O'Dea et al., 2021).

### 2.7 Sensitivity analysis and publication bias

To test for small‐study bias, we fitted a multilevel meta‐regression with sampling standard error (i.e. the square root of sampling variance) as a moderator (a modification of Egger's regression). Likewise, to test for time‐lag bias (i.e. a decline effect), we fitted a multilevel meta‐regression with the year of publication (mean‐centred, to help with interpretation) as a continuous moderator. Finally, we fitted an ‘all‐in’ publication bias test, which included the sampling standard error and year of publication to test for small‐study bias and time‐lag bias, as well as the moderators (above) to account for heterogeneity in our data (Nakagawa et al., 2022).

## 3 RESULTS

A total of 177 effect sizes covering dates of arrival at and departure from breeding and non‐breeding grounds were obtained from 54 papers, including 87 cohorts of birds (Table 1). These effect sizes represent 47 species, comprising 18 landbird, 15 seabird and 14 waterbird species. For most species, estimates were only reported by one study and only a few species had estimates from several studies (five studies estimated repeatability for Black‐tailed godwit *Limosa limosa*, three for Bar‐tailed godwit *Limosa lapponica*, three for Pied flycatcher *Ficedula hypoleuca* and two for Barn swallow *Hirundo rustica*).

**TABLE 1:** Number of effect sizes, cohorts, studies, the median (range) sample size of individuals and the median (range) repeated measures per individual (*k*) analysed in the meta‐analyses. The total dataset is summarised separately for the overall meta‐analysis, followed by a summary that illustrates the distribution of data based on ecological group (as described by Geen et al., 2019) and tracking method of individuals included in the analyses

Meta‐analysis		Effect sizes	Cohort	Studies	Median *n* (range)	Median *k* (range)
All data		177	87	54^a^	12 (3–1,232)	2.3 (1.1–12.4)
**Ecological group**	**Tracking method**					
Landbird	Conventional	19	19	11	39 (12–480)	2.3 (2.0–5.2)
	GLS	19	6	6	9 (3–33)	2 (2.0–2.3)
	Satellite	16	4	3	6 (3–25)	3.55 (2.6–5.0)
Waterbird	Conventional	21	18	12	44 (11–180)	2.7 (2.0–12.4)
	GLS	18	6	4	16 (6–36)	2.5 (2.0–2.9)
	Satellite	16	5	5	12 (5–35)	3 (2.0–4.5)
Seabird	Conventional	2	2	1	940 (648–1,232)	4.35 (4.3–4.4)
	GLS	54	24	10	7 (3–76)	2 (1.1–4.3)
	Satellite	12	3	3	4 (4–82)	2.93 (2.5–3.5)

^a^ Note that the total number of studies is one less than the sum of the number of studies when divided by ecological group and tracking method as one study tracked the same species using two different methods.

**FIGURE 1:**
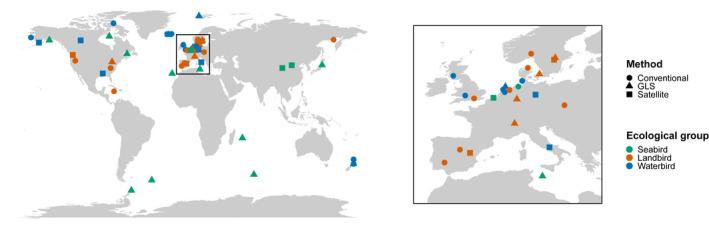
The marking locations of birds for all studies with repeatability estimates collated from the literature and included in analyses, coloured by ecological group (waterbird, seabird or landbird), and shaped by tracking method (conventional, satellite or GLS)

The median and mean sample sizes (number of individuals tracked) per effect size were 12 and 39.5, respectively (range: 3–1,232; Table 1). Conventional methods (ringing and colour‐ringing) allowed for a larger number of individuals to be tracked across all three ecological groups compared to GLS and satellite methods and over a longer period (Table 1). Most studies tracked individuals over 2, 3 or 4 years, although one study tracked some individuals for up to 20 years (*k* of study = 12.4 years). The majority of the extracted repeatability values originated from temperate latitudes in Europe and North America (77.9%; Figure 1). Of the articles calculating ANOVA‐ or LMM‐based repeatability, only 26% reported the unstandardised estimates for both within‐ and among‐individual variances.

### 3.1 Overall repeatability and heterogeneity

**FIGURE 2:**
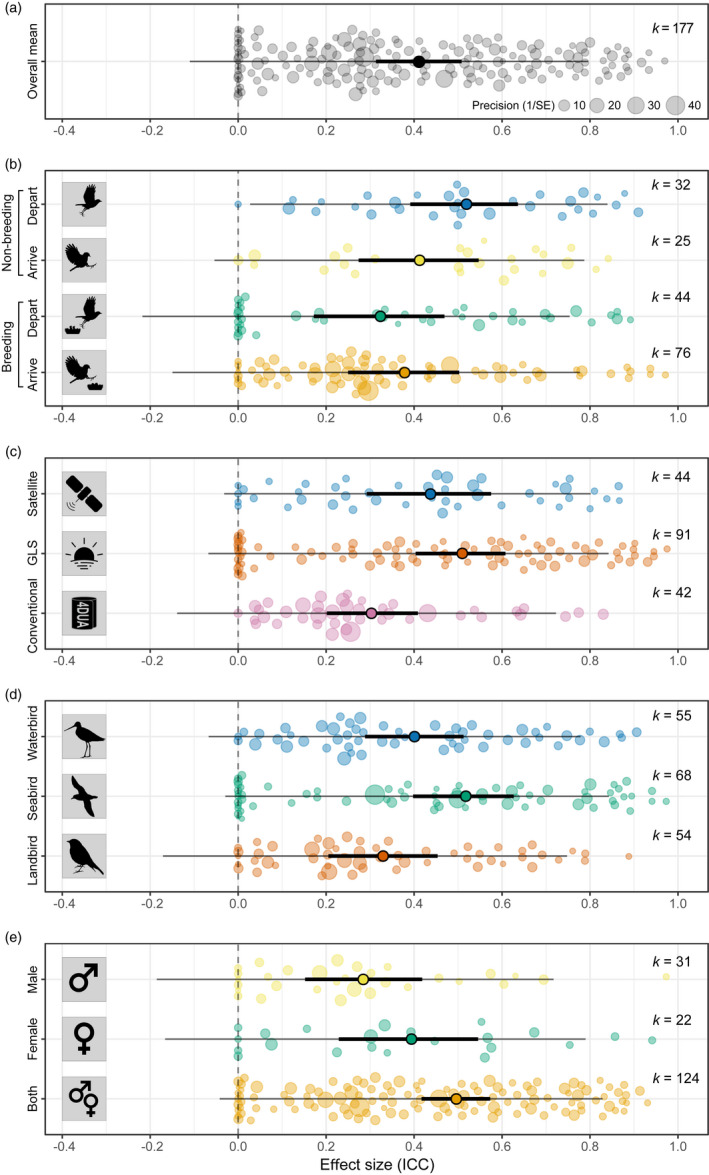
Repeatability of avian migration timing for (a) all estimates together; (b) annual migration events; (c) tracking methods; (d) ecological groups and (e) sex. Plots show mean(s) with 95% confidence intervals (thick lines, indicating uncertainty around the overall estimate) and 95% prediction intervals (thin lines, indicating the possible range for a new effect size [without sampling errors]), observed effect sizes (back‐transformed to ICC) scaled by precision (circles) and *k* = number of effect sizes

The phylogenetic multilevel meta‐analysis (intercept‐only) model revealed a mean repeatability estimate (ICC) for all avian migratory timings across the whole annual cycle of 0.414 (95% confidence interval, hereafter, CI = [0.313–0.508]; Figure 2a; Table S3). A similar model, but without controlling for phylogeny, also showed a statistically significant overall repeatability (multilevel meta‐analysis: ICC_[all]_ = 0.421, CI = [0.348:0.490]; Table S3). The total heterogeneity in the dataset was high (*I* ^2^_[total]_ = 84.2%), which is common across ecological meta‐analyses (Senior et al., 2016). When *I* ^2^ was partitioned, 49.7% was attributed to effect ID, 0% to paper ID, 0% to cohort ID, 27.3% to species ID and 7.2% to phylogeny.

### 3.2 Variation in repeatability estimates

Repeatability values vary across the annual cycle, with departure from the non‐breeding grounds being the most repeatable, and departure from the breeding grounds being the least repeatable (ICC_[depart non‐breeding]_ = 0.522, CI = [0.391:0.636]; ICC_[arrival breeding]_ = 0.381, CI = [0.250:0.503]; ICC_[arrival non‐breeding]_ = 0.416, CI = [0.274:0.547]; ICC_[depart breeding]_ = 0.326, CI = [0.172:0.469]; Figure 2b; Table S4). However, there were only statistically significant differences between departure from the breeding grounds and (a) arrival at and (b) departure from, the non‐breeding grounds, and between arrival at the breeding grounds and departure from the non‐breeding grounds (Table S4).

There was no statistically significant difference in repeatability between males and females, but there was between males and the ‘mixed’ (both/unknown) group (ICC_[male]_ = 0.287, CI = [0.152:0.419]; ICC_[female]_ = 0.397, CI = [0.229:0.545]; ICC_[mixed]_ = 0.499, CI = [0.417:0.573]; Figure 2e; Table S6). However, this effect seemed to be due to the fact that the majority of repeatability estimates measured for males only were represented by the two least repeatable annual events (arrival at breeding grounds, *n* = 22; departure from the breeding grounds, *n* = 7; out of 31), and sample sizes for males and females only were small. None of the other moderators (tracking method (ICC_[conventional]_ = 0.306, CI = [0.202:0.409]; ICC_[GLS]_ = 0.512, CI = [0.404:0.608]; ICC_[satellite]_ = 0.440, CI = [0.292:0.575]; Figure 2c; Table S5), ecological group (ICC_[seabird]_ = 0.520, CI = [0.398:0.626]; ICC_[waterbird]_ = 0.404, CI = [0.289:0.513]; ICC_[landbird]_ = 0.333, CI = [0.205:0.454]; Figure 2d; Table S7) or number of samples per individual (slope = −0.011, CI = [−0.062:0.041]; Figure S4; Table S8)) showed statistically significant influences on repeatability.

### 3.3 Model selection and multi‐model inference

We found five candidate models within two units of AICc from the best‐fitting model. All five moderators tested in our univariate models were included in the top five models, with annual event being the most important predictor (Table S9). Our model‐averaging approach highlighted the most repeatable period of the annual cycle to be departure from the non‐breeding grounds, with statistically significant differences in repeatability between that period and (a) arrival at and (b) departure from, the breeding grounds. Arrival at the non‐breeding grounds was also statistically significantly more repeatable than departure from the breeding grounds (Table S10). The importance of this moderator is consistent with our univariate models. However, the association we observed in our univariate meta‐regression with sex included as a moderator was not robust to the model averaging. Finally, in our top model, we found repeatability of avian migratory behaviours to be statistically significantly influenced by annual event and ecological group (Table S9).

### 3.4 Sensitivity analysis and publication bias

In the univariate meta‐regression models to test for bias, our results revealed little statistical sign of small‐study or time‐lag bias. The slope of sampling standard error was not statistically significant (slope = 0.213, CI = [−0.326:0.752]), indicating that effect sizes with larger SEs (i.e. more uncertain effect sizes) do not tend to be larger (Table S20), and the estimated effect of publication year was very close to zero (slope = 0.008, CI = [−0.002:0.019]), suggesting there has been no linear change in effect sizes over time since the first effect size was published (Table S21). These results were consistent with those from the multi‐moderator meta‐regression which explained a sizeable amount of the heterogeneity in our data (*R* ^2^ = ~21%; Figures S5–S6; Table S22).

## 4 DISCUSSION

Advances in tracking technology have allowed the movements of individual birds on repeated journeys to be recorded, which has fuelled interest in the scale of individual variation in migratory journeys. Our meta‐analysis of avian studies tracking the repeat journeys of individuals reveals that repeatability estimates (ICC) of avian migration timing averaged 0.414 (95% CI = 0.3–0.5), although there existed a high heterogeneity (*I* ^2^_[total]_ > 84%). Repeatability estimates of the four annual events (arrival at, and departure from, breeding and non‐breeding grounds) focused on in this study were found to vary, with departure from the non‐breeding grounds being the most repeatable. However, there was no statistically significant difference in repeatability across ecological groups, the tracking method used to calculate repeatability, between sexes, or with the number of measurements per individual.

Our overall ICC of 0.414 was similar to the migration repeatability estimate from an earlier meta‐analysis (ICC = ~0.46; Holtmann et al., 2017). Given the spread of migratory timings that is typical for migratory bird populations (Kikuchi & Reinhold, 2021), our findings suggest that consistent individual differences in arrival at, and departure from, breeding and non‐breeding grounds is a common feature of avian migration. Population‐level shifts in phenology of many migratory species are common at present (Gordo, 2007; Gunnarsson & Tómasson, 2011), and these could arise from individuals responding directionally to changing environmental conditions and/or by generational changes in the frequency of individuals with different timings within populations. For example, Gill et al. (2014) showed individual Icelandic black‐tailed godwits (*L. l. islandica*) to be consistent in spring arrival dates, and that advancing spring arrival dates were driven by new recruits to the population with differing phenology distributions than their predecessors. Changes in the distribution of phenologies within a population could reflect changes in the conditions influencing the development of individual phenologies and/or their subsequent survival rates (Gill et al., 2019), and could be influenced by heritable components of migratory behaviours (see Dochtermann et al., 2019). Consequently, a focus on understanding (a) the environmental and/or demographic factors influencing between‐individual phenological variation and (b) the extent to which individual variation in phenology is directional with respect to changing environmental conditions is likely to be needed to understand how phenological change happens, and thus how rapidly species may adapt to changing environmental conditions.

Repeatability values were found to vary significantly across the annual cycle and, contrary to our predictions, departure from the non‐breeding grounds was found to be the most repeatable. This suggests that the other annual events likely have higher within‐individual variation relative to between‐individual variation. The significantly higher repeatability of departure from the non‐breeding grounds than arrival at the breeding grounds might suggest that the environmental conditions experienced on migration can influence timing of arrival, which may be especially true for long‐distance migrants (Drake et al., 2014; Carneiro et al., 2019; but see Brown et al., 2021). Departure from the breeding grounds and hence arrival at the non‐breeding grounds may also be constrained by events during the breeding season. For example, the timing of departure from the breeding grounds is likely to vary with the timing and outcome of breeding attempts, which can vary across years and individuals. For example, in many seabirds, successful breeders tend to leave later than failed breeders (Catry et al., 2013), while many migratory passerines and waders may lay replacement clutches following nest loss (Morrison et al., 2019), with knock‐on effects for departure dates. This may therefore increase within‐individual variation in these timings and thereby decrease repeatability. However, relatively few studies have considered the effect of breeding outcome on individual repeatability in migratory timing (Catry et al., 2013; Phillips et al., 2005; Yamamoto et al., 2014).

Across the three ecological groups (waterbird, seabird and landbird), there was no statistically significant variation in repeatability values, suggesting consistent individual differences in migratory timings is a common feature of migratory systems (Gill et al., 2014). However, most studies that have investigated repeatability in migration have focused on species breeding at temperate and polar latitudes. The locations extracted for studies in this review represent where individuals were tagged (which were the breeding grounds for 89% of studies), but many species spend their non‐breeding period in the tropics. Our review has highlighted a lack of studies exploring repeatability of species breeding in the tropics (but see Jaeger et al., 2017; Franklin, Norris, et al., 2022), where seasonality is less marked and, particularly for seabirds, resources are often less predictable than at higher latitudes (Weimerskirch, 2007). We therefore propose this should be a priority for future research. For some tropical species, at least for most tropical seabirds, the timing of breeding tends to be more variable at the population level compared to higher latitudes with some species breeding year‐round, while others show flattened peaks that extend over several months. Consequently, repeatability may be naturally inflated when a large number of viable phenologies exist in a population. However, many tropical species do not make long‐distance migrations, which may make finding information on arrival and departure timings difficult. A recent study on a population of blue tits *Cyanistes caeruleus* showed there to be substantial individual variation and high repeatability in the timing of arrival at the breeding grounds (Gilsenan et al., 2020), suggesting that repeatability in timings may be a common feature even in species that are generally considered non‐migratory.

Despite the different temporal and spatial resolutions of the three tracking methods considered in this study, there was no statistically significant effect of tracking method on repeatability estimates. Considering that conventional methods rely on the spatiotemporal distribution of colour‐ring observers and/or the activity of ringing stations, whereas geolocators and GPS/satellite tags are more likely to be tracking individuals in real time, it is perhaps surprising that repeatability is captured equally well by all three methods. However, it is likely that there will be lower confidence in repeatability estimates measured using methods with lower resolution (see Korner‐Nievergelt et al., 2012; Strandberg et al., 2009). Very few studies have used two or more different methods to estimate repeatability of a single species, but those that did reported no variation with type of device (Senner et al., 2019). This may be different, however, when estimating spatial repeatability due to the different spatial resolutions and measurement errors of each method (see Dingemanse et al., 2022). For example, geolocators can have large errors around location estimates (Halpin et al., 2021; Phillips et al., 2004), which may underestimate repeatability due to uncertainty when a bird reaches an exact location. Nonetheless, it is important to note the costs and limitations associated with each tracking method that is likely to be a constraint of the study system.

The number of studies tracking repeated individual migratory journeys has increased greatly over the past decade, but the number that actually report repeatability of key elements of these journeys is much lower. Reasons as to why these estimates have not been reported, if given, have included the number of individuals with repeat tracks being too small (e.g. *n* = 9; van Bemmelen et al., 2019). However, we have identified studies calculating repeatability with as few as three individuals (Vardanis et al., 2016; Wellbrock et al., 2017; but see Wolak et al., 2012). Regardless of the method used, our study showed no effect of the number of measurements per individual on repeatability suggesting that calculating repeatability is always worthwhile, although it is important to note that the power of those estimates with small samples may be low (Dingemanse & Dochtermann, 2013).

The repeatability estimates used in this study were all for breeding adults, and it is possible that migratory timings could vary with age, especially if they are refined with age and experience (e.g. Campioni et al., 2019). This age‐related variation may be especially true for long‐lived individuals; however, shifts in migratory timings with age would need to be directional in order for ontogeny to drive phenological change. In addition, a potential caveat, which may affect repeatability estimates and thus comparisons across studies, is the different definitions and calculations of breeding and non‐breeding locations across studies. For example, arrival at the breeding grounds can range from entry into the nest/burrow (Yamamoto et al., 2014), entry to breeding territory (Kentie et al., 2017) and entry into region/area (Carneiro et al., 2019), which may cause noise and, potentially, systematic bias in repeatability estimates across studies. For example, arrival into a breeding territory could be more repeatable than arrival into the breeding region. This again, may be down to the tracking method used and its resolution, and the species in question.

Repeatability represents the proportion of the total phenotypic variation (sum of between‐individual variance, within‐individual variance and measurement error) in the sampled population that can be attributed to variation between groups (usually individuals). Therefore, it is important to note that the same repeatability estimates can arise from different patterns of these variance components (see Dochtermann & Royauté, 2019). Interpreting repeatability would therefore be aided greatly by knowing the spread of variation that exists in the sampled population and estimations of measurement error. Only 26% of studies included in our meta‐analysis provided unstandardised estimates for both within‐ and among‐individual variances, which is slightly lower than that found by Sánchez‐Tójar et al. (2022) (30.7%, 95% CI = 22.0 to 41.0), and none formally quantified measurement error. While we included tracking method in our meta‐analysis to investigate how repeatability varies with devices with varying measurement errors, this component can also vary with environmental conditions (Dingemanse et al., 2022) and thus is likely to add noise to comparative patterns in repeatability. We therefore support the recommendation that authors report the variance components and measurement errors underpinning the reported repeatability estimates where possible, as well as the coefficients of variation for each hierarchical level (Dingemanse & Wright, 2020; Sánchez‐Tójar et al., 2022), and the specific details of model structure (error structures, transformations and structure of random and fixed effects) to aid evaluation of differences in specific variance components (Pick et al., 2019; Royauté & Dochtermann, 2021; Sánchez‐Tójar et al., 2022). Very few of the studies in our literature search reported these elements, which may have reduced the power of our models.

In addition to repeatability in migratory timing, it is also important to consider repeatability in migratory routes and locations. This aspect of migration was not touched upon in this study, but many studies also report high levels of fidelity to breeding and wintering locations (e.g. Delord et al., 2019; Grist et al., 2014; Ramírez et al., 2016), and migratory routes (López‐López et al., 2014; but see also Dias et al., 2011; Dias et al., 2013). Throughout the literature, a variety of methods have been used to investigate spatial repeatability (e.g. Dias et al., 2013; Fayet et al., 2016; Ramírez et al., 2016), making comparisons across studies difficult. However, understanding repeatability of migration in both space and time will be crucial for understanding how species will adapt to environmental change.

In conclusion, the similar repeatability estimates of avian migration timing reported by studies of many different species suggest that consistent individual differences in migratory timings is likely to be a common feature of migratory systems. In many cases, repeated collection of individual migration data is not intentional, but rather a by‐product of retrieving a tracking device two or more years post‐deployment. There is also a current gap in the literature with limited information on tropical species, which may limit our understanding of how these species may respond to environmental change in less strongly seasonal environments. As phenological responses to environmental change will depend on the processes that drive within‐ and between‐individual variation and change in migratory timings, methods to disentangle within‐ and between‐individual variation should be incorporated into study designs, for example through structured sampling of individuals across phenological ranges. As migration phenologies are often associated with variation in demographic rates, understanding the consequences of phenological variation will be important for future conservation management strategies and understanding population change.

## CONFLICT OF INTEREST

The authors declare no conflict of interest.

## AUTHORS' CONTRIBUTIONS

K.A.F., J.A.G., M.A.C.N., K.N., N.R. and S.J.B. conceived the idea of the study; K.A.F. collected the data, conducted the statistical analyses and wrote the manuscript; S.N. provided statistical advice and support. All authors critically revised the manuscript, contributed to interpreting results and gave the final approval for publication.

## Supporting information


Appendix S1

## Data Availability

Data, code and lists of screened studies are available from the Dryad Digital Repository https://doi.org/10.5061/dryad.n02v6wx09 (Franklin, Nicoll, et al., 2022).
