# Supplementary material for: Individual repeatability of avian migration phenology: A systematic review and meta‐analysis
Source: J Anim Ecol. 2022 Apr 18;91(7):1416–30. doi: 10.1111/1365-2656.13697 (PMC9546039; doi:10.1111/1365-2656.13697)
Supplement: Supplementary file 1 — Appendix S1 [file JANE-91-1416-s001.html]

Individual repeatability of avian migration phenology: a systematic review and meta-analysis


Code 

- Show All Code
- Hide All Code
- Download Rmd

# Individual repeatability of avian migration phenology: a systematic review and meta-analysis

### Appendix S1

#### Kirsty A. Franklin\*, Malcolm A. C. Nicoll, Simon J. Butler, Ken Norris, Norman Ratcliffe, Shinichi Nakagawa & Jennifer A. Gill

#### 19 March 2022

## Setups

### Loading packages and custom functions

```
# loading packages
#devtools::install_github("itchyshin/orchard_plot", subdir = "orchaRd", force = TRUE, build_vignettes = TRUE)
pacman::p_load(SciViews,
               tidyverse,
               dplyr,
               metafor, # package for meta-analysis
               rotl, # package for phylogeny
               ape, # package for phylogeny
               cowplot, # combining multiple plots
               here, # making folder path usable for all
               clubSandwich, # package to assist metafor
               orchaRd, # plotting orchaRd plots
               MuMIn, # multi-model inference
               kableExtra, # making nice tables
               patchwork, # putting ggplots together
               png, # reading png files
               grid, # graphic layout manipulation
               pander, # nice tables
               ggplot2 # making figures
)
```

#### Custom functions

We have 4 custom functions named : `Zr_transformation()`, `Calc_SV()`, `Zr_to_ICC()`, and `cont_gen()`, all of which are used later (see below for their functionality) and the code is included here.

```
# Custom functions

# Getting Zr and its sampling variance from repeatability values and sample
# size information Function for Fisher's Z transformation (Zr) for
# correlation-based repeatabilities (r) and ICC (Holtmann et al. 2017, Table 1)
Zr_transformation <- function(r, K, Est) {

    if (Est == "ICC") {
        Zr <- 0.5 * ln(((1 + (K - 1) * r)/(1 - r)))
    }
    if (Est == "r") {
        Zr <- 0.5 * ln(((1 + r)/(1 - r)))
    }
    Zr
}

# Function for sampling variance for correlation-based repeatabilities (r) and
# ICC (Holtmann et al. 2017, Table 1)
Calc_SV <- function(K, N, Est) {

    if (Est == "ICC") {
        VZr <- K/(2 * ((N - 2) * (K - 1)))
    }
    if (Est == "r") {
        VZr <- 1/(N - 3)
    }
    VZr
}

# Function for back-transforming Zr to ICC
Zr_to_ICC <- function(x, k) {
    (exp(2 * x) - 1)/(exp(2 * x) + k - 1)
}

# Contrast name generator for tibble tables (from Hayward et al. 2021)
cont_gen <- function(name) {
    combination <- combn(name, 2)
    name_dat <- t(combination)
    names <- paste(name_dat[, 1], name_dat[, 2], sep = "-")
    return(names)
}
```

## Supplementary Methods

### Supplementary information for the literature search

We aimed to conduct a comprehensive search for studies estimating repeatability of temporal parameters of avian migration using a combination of approaches. We focused on arrival at, and departure from, breeding and non-breeding grounds. First, we performed a systematic search for published studies using the Web of Science and Scopus online databases on 1st June 2021 (Timespan: all years). Second, we consulted a recently published meta-analysis of hormonal, metabolic and behavioural repeatability in birds (Holtmann et al., 2017), which included repeatability estimates of migration. We manually checked each entry from those sources to confirm suitability for our purposes and extracted additional moderator variables to be used in our analyses (see below). Finally, in order to add to – and validate the accuracy of – the results of the literature search, we searched the reference lists of papers already in our accepted reference library. The details of these search strategies and the Boolean search strings used are presented below, along with a flow diagram (often referred to as a PRISMA flow chart – the Preferred Reporting Items in Systematic Reviews and Meta-Analyses; Figure S1) which shows the stages at which studies were disqualified or eventually used in the current study.

**Web of Science Core Collection:**

(TS=(“repeat\*” OR “intraclass correlation” OR “ICC” OR “individual variation” OR “intra-individual variation” OR “between-individual variation” OR “consisten\*” OR “flexib\*”) AND TS=(“migration” OR “migratory”) AND TS=(“\*bird\*” OR “aves” OR “avian”)) AND (SU=(Behavioral Sciences OR Biodiversity & Conservation OR Environmental Sciences & Ecology OR Evolutionary Biology OR Genetics & Heredity OR Marine & Freshwater Biology OR Oceanography OR Veterinary Sciences OR Zoology))

**Scopus:**

TITLE-ABS-KEY ( “repeat\*” OR “intraclass correlation” OR “ICC” OR “individual variation” OR “intra-individual variation” OR “between-individual variation” OR “consisten\*” OR “flexib\*” ) AND TITLE-ABS-KEY ( “migration” OR “migratory” ) AND TITLE-ABS-KEY ( “\*bird\*” OR “aves” OR “avian” ) AND ( LIMIT-TO ( SUBJAREA , “AGRI” ) OR LIMIT-TO ( SUBJAREA , “ENVI” ) OR LIMIT-TO ( SUBJAREA , “VETE” ) )

We followed reporting guidelines outlined in the PRISMA-EcoEvo checklist (O’Dea et al., 2021).

### PRISMA flow chart

#### Figure S1

**Figure S1.** PRISMA flow chart summarising search methods and screening for studies included in analyses, and reasons for excluding studies.

### Decision tree

#### Figure S2

**Figure S2.** Decision tree used to evaluate studies for inclusion and exclusion at the stage of title and abstract screening.

## The Repeatability Dataset

### Table of the dataset

Below is the dataset used for our meta-analysis, followed by explanations of the variables extracted from the papers included (not all variables were used for our analyses).

#### Table S1

The meta-analytic dataset of this study.

```
# getting the data and formating some variables (turning chraracter vectors to
# factors)
df <- read_csv(here("data", "Meta-analysis_data-2022.csv"), na = "NA", show_col_types = F) %>%
    mutate_if(is.character, as.factor)

# making a scrollable table of dataset
kable(df, "html") %>%
    kable_styling("striped", position = "left") %>%
    scroll_box(width = "100%", height = "500px")
```

| es\_ID | paper\_ID | cohort\_ID | species\_ID | species\_common | species\_latin | taxa | sex | n | k | est | R | fixed\_yn | fixed\_var | unstandardized\_variance | method | annual\_event | location | country | long | lat | tag\_period | notes | data\_location | data\_presentation | title | pub\_year | authors | journal | DOI | Papers for Data Extraction::fulltext\_ID |
| --- | --- | --- | --- | --- | --- | --- | --- | --- | --- | --- | --- | --- | --- | --- | --- | --- | --- | --- | --- | --- | --- | --- | --- | --- | --- | --- | --- | --- | --- | --- |
| es001 | p001 | c001 | s039 | American redstart | Setophaga ruticilla | Landbird | B | 74 | 2.64 | ICC | 0.3820 | N | NA | no | Conventional | Nonbreed\_depart | southwestern Jamaica at the Font Hill Nature reserve | North America | -77.950000 | 18.03000 | Non-breeding |  | p. 3440, text, results | text | Rainfall-induced changes in food availability modify the spring departure programme of a migratory bird | 2011 | Studds, C. E. and Marra, P. P. | Proceedings of the Royal Society B: Biological Sciences | 10.1098/rspb.2011.0332 | ft161 |
| es002 | p002 | c002 | s029 | American white pelican | Pelecanus erythrorhynchos | Waterbird | B | 12 | 2.00 | ICC | 0.0200 | N | NA | no | Satellite | Depart\_breed | Multiple areas in US | North America | -89.883000 | 32.48000 | Non-breeding |  | Table 1, p. 4 | text | Advances and environmental conditions of spring migration phenology of American white pelicans | 2017 | King, D.T. and Wang, G. and Yang, Z. and Fischer, J. W. | Scientific Reports | 10.1038/srep40339 | ft163 |
| es003 | p002 | c002 | s029 | American white pelican | Pelecanus erythrorhynchos | Waterbird | B | 12 | 2.00 | ICC | 0.3180 | N | NA | no | Satellite | Arrival\_breed | Multiple areas in US | North America | -89.883000 | 32.48000 | Non-breeding |  | Table 1, p. 4 | text | Advances and environmental conditions of spring migration phenology of American white pelicans | 2017 | King, D.T. and Wang, G. and Yang, Z. and Fischer, J. W. | Scientific Reports | 10.1038/srep40339 | ft163 |
| es004 | p002 | c002 | s029 | American white pelican | Pelecanus erythrorhynchos | Waterbird | B | 12 | 2.00 | ICC | 0.3670 | N | NA | no | Satellite | Nonbreed\_arrival | Multiple areas in US | North America | -89.883000 | 32.48000 | Non-breeding |  | Table 1, p. 4 | text | Advances and environmental conditions of spring migration phenology of American white pelicans | 2017 | King, D.T. and Wang, G. and Yang, Z. and Fischer, J. W. | Scientific Reports | 10.1038/srep40339 | ft163 |
| es005 | p002 | c002 | s029 | American white pelican | Pelecanus erythrorhynchos | Waterbird | B | 12 | 2.00 | ICC | 0.7630 | N | NA | no | Satellite | Nonbreed\_depart | Multiple areas in US | North America | -89.883000 | 32.48000 | Non-breeding |  | Table 1, p. 4 | text | Advances and environmental conditions of spring migration phenology of American white pelicans | 2017 | King, D.T. and Wang, G. and Yang, Z. and Fischer, J. W. | Scientific Reports | 10.1038/srep40339 | ft163 |
| es006 | p003 | c003 | s016 | Barn swallow | Hirundo rustica | Landbird | M | 15 | 2.00 | ICC | 0.5000 | N | NA | no | Conventional | Arrival\_breed | Badajoz, Southern Spain | Europe | -6.490000 | 38.53000 | Breeding |  | p. 58, text, results | text | Antioxidants and condition-dependence of arrival date in a migratory passerine | 2004 | Ninni, P. and de Lope, F. and Saino, N. and Haussy, C. and Moller, A. P. | Oikos | 10.1111/j.0030-1299.2004.12516.x | ft012 |
| es007 | p004 | c004 | s016 | Barn swallow | Hirundo rustica | Landbird | M | 23 | 5.20 | ICC | 0.5100 | N | NA | no | Conventional | Arrival\_breed | Kraghede, Denmark | Europe | 10.000000 | 57.20000 | Breeding |  | p. 204, text, results | text | Heritability of arrival date in a migratory bird | 2001 | Moller, A. P. | Proceedings of the Royal Society B-Biological Sciences | 10.1098/rspb.2000.1351 | ft062 |
| es008 | p005 | c005 | s023 | Bar-tailed godwit | Limosa lapponica baueri | Waterbird | B | 11 | 2.00 | ICC | 0.3800 | N | NA | no | Conventional | Nonbreed\_depart | western Firth of Thames, NZ | Not | 175.316000 | -37.18300 | Non-breeding |  | p. 518, text, results | text | Consistent annual schedules in a migratory shorebird | 2006 | Battley, P. F. | Biology Letters | 10.1098/rsbl.2006.0535 | ft029 |
| es009 | p006 | c006 | s023 | Bar-tailed godwit | Limosa lapponica baueri | Waterbird | B | 6 | 2.00 | ICC | 0.4700 | N | NA | yes | GLS | Depart\_breed | Manawatu River estuary, New Zealand | Not | 175.360000 | -40.78300 | Non-breeding |  | Table 1, p. 6 | text | Absolute Consistency: Individual versus Population Variation in Annual-Cycle Schedules of a Long-Distance Migrant Bird | 2013 | Conklin, J. R. and Battley, P. F. and Potter, M. A. | PLoS ONE | 10.1371/journal.pone.0054535 | ft001 |
| es010 | p006 | c007 | s023 | Bar-tailed godwit | Limosa lapponica baueri | Waterbird | B | 44 | 2.00 | ICC | 0.6600 | N | NA | yes | Conventional | Nonbreed\_arrival | Manawatu River estuary, New Zealand | Not | 175.360000 | -40.78300 | Non-breeding |  | Table 1, p. 6 | text | Absolute Consistency: Individual versus Population Variation in Annual-Cycle Schedules of a Long-Distance Migrant Bird | 2013 | Conklin, J. R. and Battley, P. F. and Potter, M. A. | PLoS ONE | 10.1371/journal.pone.0054535 | ft001 |
| es011 | p006 | c006 | s023 | Bar-tailed godwit | Limosa lapponica baueri | Waterbird | B | 8 | 2.00 | ICC | 0.7700 | N | NA | yes | GLS | Nonbreed\_arrival | Manawatu River estuary, New Zealand | Not | 175.360000 | -40.78300 | Non-breeding |  | Table 1, p. 6 | text | Absolute Consistency: Individual versus Population Variation in Annual-Cycle Schedules of a Long-Distance Migrant Bird | 2013 | Conklin, J. R. and Battley, P. F. and Potter, M. A. | PLoS ONE | 10.1371/journal.pone.0054535 | ft001 |
| es012 | p006 | c007 | s023 | Bar-tailed godwit | Limosa lapponica baueri | Waterbird | B | 49 | 2.00 | ICC | 0.8200 | N | NA | yes | Conventional | Nonbreed\_depart | Manawatu River estuary, New Zealand | Not | 175.360000 | -40.78300 | Non-breeding |  | Table 1, p. 6 | text | Absolute Consistency: Individual versus Population Variation in Annual-Cycle Schedules of a Long-Distance Migrant Bird | 2013 | Conklin, J. R. and Battley, P. F. and Potter, M. A. | PLoS ONE | 10.1371/journal.pone.0054535 | ft001 |
| es013 | p005 | c005 | s023 | Bar-tailed godwit | Limosa lapponica baueri | Waterbird | B | 38 | 2.20 | ICC | 0.8300 | N | NA | no | Conventional | Nonbreed\_depart | western Firth of Thames, NZ | Not | 175.316000 | -37.18300 | Non-breeding |  | p. 518, text, results | text | Consistent annual schedules in a migratory shorebird | 2006 | Battley, P. F. | Biology Letters | 10.1098/rsbl.2006.0535 | ft029 |
| es014 | p007 | c008 | s023 | Bar-tailed godwit | Limosa lapponica baueri | Waterbird | B | 45 | 3.00 | ICC | 0.8360 | N | NA | yes | Conventional | Depart\_breed | Manawatu River estuary, New Zealand | Not | 175.360000 | -40.78300 | Non-breeding |  | p. 856, text, results | text | Impacts of wind on individual migration schedules of New Zealand bar-tailed godwits | 2011 | Conklin, J. R. and Battley, P. F. | Behavioral Ecology | 10.1093/beheco/arr054 | ft065 |
| es015 | p006 | c006 | s023 | Bar-tailed godwit | Limosa lapponica baueri | Waterbird | B | 8 | 2.00 | ICC | 0.8600 | N | NA | yes | GLS | Nonbreed\_depart | Manawatu River estuary, New Zealand | Not | 175.360000 | -40.78300 | Non-breeding |  | Table 1, p. 6 | text | Absolute Consistency: Individual versus Population Variation in Annual-Cycle Schedules of a Long-Distance Migrant Bird | 2013 | Conklin, J. R. and Battley, P. F. and Potter, M. A. | PLoS ONE | 10.1371/journal.pone.0054535 | ft001 |
| es016 | p006 | c006 | s023 | Bar-tailed godwit | Limosa lapponica baueri | Waterbird | B | 8 | 2.00 | ICC | 0.9100 | N | NA | yes | GLS | Arrival\_breed | Manawatu River estuary, New Zealand | Not | 175.360000 | -40.78300 | Non-breeding |  | Table 1, p. 6 | text | Absolute Consistency: Individual versus Population Variation in Annual-Cycle Schedules of a Long-Distance Migrant Bird | 2013 | Conklin, J. R. and Battley, P. F. and Potter, M. A. | PLoS ONE | 10.1371/journal.pone.0054535 | ft001 |
| es017 | p008 | c009 | s012 | Bewick swan | Cygnus columbianus bewickii | Waterbird | B | 67 | 12.40 | ICC | 0.0100 | N | NA | yes | Conventional | Nonbreed\_arrival | Slimbridge | Europe | -2.400000 | 51.74000 | Non-breeding |  | calculated values from Table 1, p. 388 | text | Consistency in the timing of migration for individual Bewick’s swans | 1989 | Rees, E. C. | Animal Behaviour | 10.1016/S0003-3472(89)80031-4 | ft028 |
| es018 | p008 | c009 | s012 | Bewick swan | Cygnus columbianus bewickii | Waterbird | B | 67 | 12.40 | ICC | 0.0300 | N | NA | yes | Conventional | Nonbreed\_depart | Slimbridge | Europe | -2.400000 | 51.74000 | Non-breeding |  | calculated values from Table 1, p. 388 | text | Consistency in the timing of migration for individual Bewick’s swans | 1989 | Rees, E. C. | Animal Behaviour | 10.1016/S0003-3472(89)80031-4 | ft028 |
| es019 | p009 | c010 | s037 | Black-legged kittiwake | Rissa tridactyla | Seabird | B | 48 | 2.00 | ICC | 0.3900 | Y | AnyChick+Sex+Treatment+AnyChick\*Treatment + (1|Band) + (1|Year) | no | GLS | Arrival\_breed | Middleton Island | North America | -146.320000 | 59.44000 | Breeding |  | p. 3, text, results | text | Increased summer food supply decreases non-breeding movement in black-legged kittiwakes | 2020 | Whelan, S. and Hatch, S. A. and Irons D. B. and McKnight A. and Elliott K. H. | Biology Letters | 10.1098/rsbl.2019.0725 | ft165 |
| es020 | p009 | c010 | s037 | Black-legged kittiwake | Rissa tridactyla | Seabird | B | 48 | 2.00 | ICC | 0.7390 | Y | AnyChick+Sex+Treatment+AnyChick\*Treatment + (1|Band) + (1|Year) | no | GLS | Depart\_breed | Middleton Island | North America | -146.320000 | 59.44000 | Breeding |  | p. 3, text, results | text | Increased summer food supply decreases non-breeding movement in black-legged kittiwakes | 2020 | Whelan, S. and Hatch, S. A. and Irons D. B. and McKnight A. and Elliott K. H. | Biology Letters | 10.1098/rsbl.2019.0725 | ft165 |
| es021 | p010 | c011 | s024 | Black-tailed godwit | Limosa limosa limosa | Waterbird | B | 36 | 2.30 | ICC | 0.1390 | N | NA | yes | GLS | Depart\_breed | southwest Fryslan, The Netherlands | Europe | 5.540000 | 53.50000 | Breeding |  | Figure 2. E, p. 5 | figure | Variation From an Unknown Source: Large Inter-individual Differences in Migrating Black-Tailed Godwits | 2019 | Verhoeven, M. A. and Loonstra, A. H. J. and Senner, N. R. and McBride, A. D. and Both, C. and Piersma, T. | Frontiers in Ecology and Evolution | 10.3389/fevo.2019.00031 | ft142 |
| es022 | p011 | c012 | s024 | Black-tailed godwit | Limosa limosa islandica | Waterbird | B | 46 | 2.46 | ICC | 0.1800 | N | NA | no | Conventional | Arrival\_breed | Iceland | Europe | -21.747000 | 64.45400 | Breeding |  | p. 1122, text, results | text | Population-scale drivers of individual arrival times in migratory birds | 2006 | Gunnarsson, T. G. and Gill, J. A. and Atkinson, P. W. and Gelinaud, G. and Potts, P. M. and Croger, R. E. and Gudmundsson, G. A. and Appleton, G. F. and Sutherland, W. J. | Journal of Animal Ecology | 10.1111/j.1365-2656.2006.01131.x | ft116 |
| es023 | p012 | c013 | s024 | Black-tailed godwit | Limosa limosa limosa | Waterbird | M | 70 | 3.30 | ICC | 0.1800 | N | NA | no | Conventional | Arrival\_breed | Workumerwaard, the Netherlands | Europe | 5.400000 | 52.40000 | both? |  | Table 1, p. 1027 | text | Repeatable timing of northward departure, arrival and breeding in Black-tailed Godwits Limosa l. limosa, but no domino effects | 2011 | Louren<e7>o, P. M. and Kentie, R. and Schroeder, J. and Groen, N. M. and Hooijmeijer, J. C. E. W. and Piersma, T. | Journal of Ornithology | 10.1007/s10336-011-0692-3 | ft121 |
| es024 | p013 | c015 | s024 | Black-tailed godwit | Limosa limosa limosa | Waterbird | B | 180 | 3.10 | ICC | 0.2400 | N | NA | no | Conventional | Arrival\_breed | southwest Friesland, the Netherlands | Europe | 5.083000 | 52.91600 | both? |  | p. 2816, text, results | text | Does wintering north or south of the Sahara correlate with timing and breeding performance in black-tailed godwits? | 2017 | Kentie, R. and Marquez-Ferrando, R. and Figuerola, J. and Gangoso, L. and Hooijmeijer, J. C. E. W. and Loonstra, A. H. J. and Robin, F. and Sarasa, M. and Senner, N. and Valkema, H. and Verhoeven, M. A. and Piersma, T. | Ecology and Evolution | 10.1002/ece3.2879 | ft041 |
| es025 | p013 | c016 | s024 | Black-tailed godwit | Limosa limosa limosa | Waterbird | B | 131 | 2.70 | ICC | 0.2400 | N | NA | no | Conventional | Arrival\_breed | southwest Friesland, the Netherlands | Europe | 5.083000 | 52.91600 | both? |  | p. 2816, text, results | text | Does wintering north or south of the Sahara correlate with timing and breeding performance in black-tailed godwits? | 2017 | Kentie, R. and Marquez-Ferrando, R. and Figuerola, J. and Gangoso, L. and Hooijmeijer, J. C. E. W. and Loonstra, A. H. J. and Robin, F. and Sarasa, M. and Senner, N. and Valkema, H. and Verhoeven, M. A. and Piersma, T. | Ecology and Evolution | 10.1002/ece3.2879 | ft041 |
| es026 | p012 | c014 | s024 | Black-tailed godwit | Limosa limosa limosa | Waterbird | F | 81 | 2.90 | ICC | 0.2900 | N | NA | no | Conventional | Arrival\_breed | Workumerwaard, the Netherlands | Europe | 5.400000 | 52.40000 | both? |  | Table 1, p. 1027 | text | Repeatable timing of northward departure, arrival and breeding in Black-tailed Godwits Limosa l. limosa, but no domino effects | 2011 | Louren<e7>o, P. M. and Kentie, R. and Schroeder, J. and Groen, N. M. and Hooijmeijer, J. C. E. W. and Piersma, T. | Journal of Ornithology | 10.1007/s10336-011-0692-3 | ft121 |
| es027 | p010 | c011 | s024 | Black-tailed godwit | Limosa limosa limosa | Waterbird | B | 24 | 2.10 | ICC | 0.3330 | N | NA | yes | GLS | Arrival\_breed | southwest Fryslan, The Netherlands | Europe | 5.540000 | 53.50000 | Breeding |  | Figure 2. F, p. 5 | figure | Variation From an Unknown Source: Large Inter-individual Differences in Migrating Black-Tailed Godwits | 2019 | Verhoeven, M. A. and Loonstra, A. H. J. and Senner, N. R. and McBride, A. D. and Both, C. and Piersma, T. | Frontiers in Ecology and Evolution | 10.3389/fevo.2019.00031 | ft142 |
| es028 | p014 | c017 | s024 | Black-tailed godwit | Limosa limosa islandica | Waterbird | B | 54 | 5.40 | ICC | 0.5100 | N | NA | no | Conventional | Arrival\_breed | Iceland | Europe | -17.190000 | 64.58000 | Breeding |  | p. 3, text, results | text | Why is timing of bird migration advancing when individuals are not? | 2014 | Gill, J. A. and Alves, J. A. and Sutherland, W. J. and Appleton, G. F. and Potts, P. M. and Gunnarsson, T. G. | Proceedings of the Royal Society B: Biological Sciences | 10.1098/rspb.2013.2161 | ft150 |
| es029 | p010 | c011 | s024 | Black-tailed godwit | Limosa limosa limosa | Waterbird | B | 29 | 2.30 | ICC | 0.6030 | N | NA | yes | GLS | Nonbreed\_arrival | southwest Fryslan, The Netherlands | Europe | 5.540000 | 53.50000 | Breeding |  | Figure 2. E, p. 5 | figure | Variation From an Unknown Source: Large Inter-individual Differences in Migrating Black-Tailed Godwits | 2019 | Verhoeven, M. A. and Loonstra, A. H. J. and Senner, N. R. and McBride, A. D. and Both, C. and Piersma, T. | Frontiers in Ecology and Evolution | 10.3389/fevo.2019.00031 | ft142 |
| es030 | p010 | c011 | s024 | Black-tailed godwit | Limosa limosa limosa | Waterbird | B | 25 | 2.00 | ICC | 0.9370 | N | NA | yes | GLS | Nonbreed\_depart | southwest Fryslan, The Netherlands | Europe | 5.540000 | 53.50000 | Breeding |  | Figure 2. F, p. 5 | figure | Variation From an Unknown Source: Large Inter-individual Differences in Migrating Black-Tailed Godwits | 2019 | Verhoeven, M. A. and Loonstra, A. H. J. and Senner, N. R. and McBride, A. D. and Both, C. and Piersma, T. | Frontiers in Ecology and Evolution | 10.3389/fevo.2019.00031 | ft142 |
| es031 | p015 | c018 | s008 | Brown skua | Catharacta antarctica lonnbergi | Seabird | F | 11 | 2.10 | ICC | 0.4770 | Y | year | no | GLS | Depart\_breed | King George Island | Not | -59.583300 | -62.31660 | Breeding |  | Table 1, p. 220 | text | Consistent variation in individual migration strategies of brown skuas | 2017 | Krietsch, J. and Hahn, S. and Kopp, M. and Phillips, R. A. and Peter, H. U. and Lisovski, S. | Marine Ecology Progress Series | 10.3354/meps11932 | ft032 |
| es032 | p015 | c019 | s008 | Brown skua | Catharacta antarctica lonnbergi | Seabird | M | 4 | 2.50 | ICC | 0.4900 | Y | year | no | GLS | Depart\_breed | King George Island | Not | -59.583300 | -62.31660 | Breeding |  | Table 1, p. 220 | text | Consistent variation in individual migration strategies of brown skuas | 2017 | Krietsch, J. and Hahn, S. and Kopp, M. and Phillips, R. A. and Peter, H. U. and Lisovski, S. | Marine Ecology Progress Series | 10.3354/meps11932 | ft032 |
| es033 | p015 | c018 | s008 | Brown skua | Catharacta antarctica lonnbergi | Seabird | F | 12 | 2.10 | ICC | 0.8710 | Y | year | no | GLS | Arrival\_breed | King George Island | Not | -59.583300 | -62.31660 | Breeding |  | Table 1, p. 220 | text | Consistent variation in individual migration strategies of brown skuas | 2017 | Krietsch, J. and Hahn, S. and Kopp, M. and Phillips, R. A. and Peter, H. U. and Lisovski, S. | Marine Ecology Progress Series | 10.3354/meps11932 | ft032 |
| es034 | p015 | c019 | s008 | Brown skua | Catharacta antarctica lonnbergi | Seabird | M | 4 | 2.50 | ICC | 0.9720 | Y | year | no | GLS | Arrival\_breed | King George Island | Not | -59.583300 | -62.31660 | Breeding |  | Table 1, p. 220 | text | Consistent variation in individual migration strategies of brown skuas | 2017 | Krietsch, J. and Hahn, S. and Kopp, M. and Phillips, R. A. and Peter, H. U. and Lisovski, S. | Marine Ecology Progress Series | 10.3354/meps11932 | ft032 |
| es035 | p016 | c020 | s013 | Collared flycatcher | Ficedula albicollis | Landbird | M | 106 | 2.20 | ICC | 0.2400 | N | NA | yes | Conventional | Arrival\_breed | Czechia | Europe | 17.220000 | 49.83000 | Breeding |  | text, results, last paragraph | text | The genetic regulation of avian migration timing: combining candidate genes and quantitative genetic approaches in a long-distance migrant | 2021 | Krist, M. and Munclinger, P. and Briedis, M. and Adamík, P. | Oecologia | 10.1007/s00442-021-04930-x | ft056 |
| es036 | p017 | c021 | s045 | Common murre | Uria aalge | Seabird | B | 7 | 1.40 | ICC | 0.0100 | N | NA | no | GLS | Depart\_breed | 7 seabird colonies ranging 74 to 47 N | North America | -82.970000 | 62.54000 | Breeding |  | Table 2, p. 7 | text | Individual winter movement strategies in two species of murre (Uria spp.) in the Northwest Atlantic | 2014 | McFarlane Tranquilla, L. A. and Montevecchi, W. A. and Fifield, D. A. and Hedd, A. and Gaston, A. J. and Robertson, G. J. and Phillips, R. A. | PLoS ONE | 10.1371/journal.pone.0090583 | ft081 |
| es037 | p017 | c022 | s045 | Common murre | Uria aalge | Seabird | B | 7 | 2.30 | ICC | 0.3500 | N | NA | no | GLS | Arrival\_breed | 7 seabird colonies ranging 74 to 47 N | North America | -82.970000 | 62.54000 | Breeding |  | Table 2, p. 7 | text | Individual winter movement strategies in two species of murre (Uria spp.) in the Northwest Atlantic | 2014 | McFarlane Tranquilla, L. A. and Montevecchi, W. A. and Fifield, D. A. and Hedd, A. and Gaston, A. J. and Robertson, G. J. and Phillips, R. A. | PLoS ONE | 10.1371/journal.pone.0090583 | ft081 |
| es038 | p017 | c023 | s045 | Common murre | Uria aalge | Seabird | B | 6 | 2.70 | ICC | 0.4200 | N | NA | no | GLS | Arrival\_breed | 7 seabird colonies ranging 74 to 47 N | North America | -82.970000 | 62.54000 | Breeding |  | Table 2, p. 7 | text | Individual winter movement strategies in two species of murre (Uria spp.) in the Northwest Atlantic | 2014 | McFarlane Tranquilla, L. A. and Montevecchi, W. A. and Fifield, D. A. and Hedd, A. and Gaston, A. J. and Robertson, G. J. and Phillips, R. A. | PLoS ONE | 10.1371/journal.pone.0090583 | ft081 |
| es039 | p017 | c022 | s045 | Common murre | Uria aalge | Seabird | B | 7 | 1.10 | ICC | 0.5600 | N | NA | no | GLS | Depart\_breed | 7 seabird colonies ranging 74 to 47 N | North America | -82.970000 | 62.54000 | Breeding |  | Table 2, p. 7 | text | Individual winter movement strategies in two species of murre (Uria spp.) in the Northwest Atlantic | 2014 | McFarlane Tranquilla, L. A. and Montevecchi, W. A. and Fifield, D. A. and Hedd, A. and Gaston, A. J. and Robertson, G. J. and Phillips, R. A. | PLoS ONE | 10.1371/journal.pone.0090583 | ft081 |
| es040 | p017 | c021 | s045 | Common murre | Uria aalge | Seabird | B | 7 | 1.10 | ICC | 0.6400 | N | NA | no | GLS | Arrival\_breed | 7 seabird colonies ranging 74 to 47 N | North America | -82.970000 | 62.54000 | Breeding |  | Table 2, p. 7 | text | Individual winter movement strategies in two species of murre (Uria spp.) in the Northwest Atlantic | 2014 | McFarlane Tranquilla, L. A. and Montevecchi, W. A. and Fifield, D. A. and Hedd, A. and Gaston, A. J. and Robertson, G. J. and Phillips, R. A. | PLoS ONE | 10.1371/journal.pone.0090583 | ft081 |
| es041 | p017 | c023 | s045 | Common murre | Uria aalge | Seabird | B | 6 | 3.00 | ICC | 0.8200 | N | NA | no | GLS | Depart\_breed | 7 seabird colonies ranging 74 to 47 N | North America | -82.970000 | 62.54000 | Breeding |  | Table 2, p. 7 | text | Individual winter movement strategies in two species of murre (Uria spp.) in the Northwest Atlantic | 2014 | McFarlane Tranquilla, L. A. and Montevecchi, W. A. and Fifield, D. A. and Hedd, A. and Gaston, A. J. and Robertson, G. J. and Phillips, R. A. | PLoS ONE | 10.1371/journal.pone.0090583 | ft081 |
| es042 | p018 | c027 | s003 | Common swift | Apus apus | Landbird | M | 3 | 2.00 | ICC | -0.4500 | N | NA | no | GLS | Depart\_breed | near Olpe, Germany | Europe | 7.816600 | 51.03300 | Breeding |  | Table 2, p. 900 | text | ‘Same procedure as last year?’ Repeatedly tracked swifts show individual consistency in migration pattern in successive years | 2017 | Wellbrock, A. H. J. and Bauch, C. and Rozman, J. and Witte, K. | Journal of Avian Biology | 10.1111/jav.01251 | ft123 |
| es043 | p018 | c027 | s003 | Common swift | Apus apus | Landbird | M | 3 | 2.00 | ICC | -0.0700 | N | NA | no | GLS | Arrival\_breed | near Olpe, Germany | Europe | 7.816600 | 51.03300 | Breeding |  | Table 2, p. 900 | text | ‘Same procedure as last year?’ Repeatedly tracked swifts show individual consistency in migration pattern in successive years | 2017 | Wellbrock, A. H. J. and Bauch, C. and Rozman, J. and Witte, K. | Journal of Avian Biology | 10.1111/jav.01251 | ft123 |
| es044 | p018 | c027 | s003 | Common swift | Apus apus | Landbird | M | 3 | 2.00 | ICC | 0.6700 | N | NA | no | GLS | Nonbreed\_arrival | near Olpe, Germany | Europe | 7.816600 | 51.03300 | Breeding |  | Table 2, p. 900 | text | ‘Same procedure as last year?’ Repeatedly tracked swifts show individual consistency in migration pattern in successive years | 2017 | Wellbrock, A. H. J. and Bauch, C. and Rozman, J. and Witte, K. | Journal of Avian Biology | 10.1111/jav.01251 | ft123 |
| es045 | p019 | c028 | s041 | Common tern | Sterna hirundo | Seabird | B | 1232 | 4.30 | ICC | 0.2000 | Y | Reproductive stage | yes | Conventional | Arrival\_breed | Wilhelmshaven, Germany | Europe | 8.099000 | 53.50500 | Breeding | Age includes first time-breeders, inexperiend and experienced | p. 686, text, results | text | Canalization of phenology in common terns: Genetic and phenotypic variations in spring arrival date | 2013 | Arnaud, C. M. and Becker, P. H. and Dobson F. S. and Charmantier A. | Behavioral Ecology | 10.1093/beheco/ars214 | ft162 |
| es046 | p019 | c029 | s041 | Common tern | Sterna hirundo | Seabird | B | 648 | 4.40 | ICC | 0.3500 | Y | Age, sex, breeding success in previous year | yes | Conventional | Arrival\_breed | Wilhelmshaven, Germany | Europe | 8.099000 | 53.50500 | Breeding | Experienced breeders only | p. 686, text, results | text | Canalization of phenology in common terns: Genetic and phenotypic variations in spring arrival date | 2013 | Arnaud, C. M. and Becker, P. H. and Dobson F. S. and Charmantier A. | Behavioral Ecology | 10.1093/beheco/ars214 | ft162 |
| es047 | p020 | c030 | s035 | Desertas petrel | Pterodroma deserta | Seabird | B | 4 | 2.00 | ICC | 0.4900 | N | NA | yes | GLS | Arrival\_breed | Bugio Island, Madeira | Europe | -16.480000 | 32.43000 | Breeding |  | Table 2, p. 146 | text | Conservation implications of consistent foraging and trophic ecology in a rare petrel species | 2016 | Ramirez, I. and Paiva, V. H. and Fagundes, I. and Menezes, D. and Silva, I. and Ceia, F. R. and Phillips, R. A. and Ramos, J. A. and Garthe, S. | Animal Conservation | 10.1111/acv.12227 | ft025 |
| es048 | p020 | c030 | s035 | Desertas petrel | Pterodroma deserta | Seabird | B | 4 | 2.00 | ICC | 0.5600 | N | NA | yes | GLS | Depart\_breed | Bugio Island, Madeira | Europe | -16.480000 | 32.43000 | Breeding |  | Table 2, p. 146 | text | Conservation implications of consistent foraging and trophic ecology in a rare petrel species | 2016 | Ramirez, I. and Paiva, V. H. and Fagundes, I. and Menezes, D. and Silva, I. and Ceia, F. R. and Phillips, R. A. and Ramos, J. A. and Garthe, S. | Animal Conservation | 10.1111/acv.12227 | ft025 |
| es049 | p020 | c031 | s035 | Desertas petrel | Pterodroma deserta | Seabird | B | 6 | 2.50 | ICC | 0.6200 | N | NA | yes | GLS | Depart\_breed | Bugio Island, Madeira | Europe | -16.480000 | 32.43000 | Breeding |  | Table 2, p. 146 | text | Conservation implications of consistent foraging and trophic ecology in a rare petrel species | 2016 | Ramirez, I. and Paiva, V. H. and Fagundes, I. and Menezes, D. and Silva, I. and Ceia, F. R. and Phillips, R. A. and Ramos, J. A. and Garthe, S. | Animal Conservation | 10.1111/acv.12227 | ft025 |
| es050 | p020 | c031 | s035 | Desertas petrel | Pterodroma deserta | Seabird | B | 6 | 2.50 | ICC | 0.6300 | N | NA | yes | GLS | Arrival\_breed | Bugio Island, Madeira | Europe | -16.480000 | 32.43000 | Breeding |  | Table 2, p. 146 | text | Conservation implications of consistent foraging and trophic ecology in a rare petrel species | 2016 | Ramirez, I. and Paiva, V. H. and Fagundes, I. and Menezes, D. and Silva, I. and Ceia, F. R. and Phillips, R. A. and Ramos, J. A. and Garthe, S. | Animal Conservation | 10.1111/acv.12227 | ft025 |
| es051 | p020 | c032 | s035 | Desertas petrel | Pterodroma deserta | Seabird | B | 5 | 2.20 | ICC | 0.6700 | N | NA | yes | GLS | Depart\_breed | Bugio Island, Madeira | Europe | -16.480000 | 32.43000 | Breeding |  | Table 2, p. 146 | text | Conservation implications of consistent foraging and trophic ecology in a rare petrel species | 2016 | Ramirez, I. and Paiva, V. H. and Fagundes, I. and Menezes, D. and Silva, I. and Ceia, F. R. and Phillips, R. A. and Ramos, J. A. and Garthe, S. | Animal Conservation | 10.1111/acv.12227 | ft025 |
| es052 | p020 | c032 | s035 | Desertas petrel | Pterodroma deserta | Seabird | B | 5 | 2.20 | ICC | 0.6800 | N | NA | yes | GLS | Arrival\_breed | Bugio Island, Madeira | Europe | -16.480000 | 32.43000 | Breeding |  | Table 2, p. 146 | text | Conservation implications of consistent foraging and trophic ecology in a rare petrel species | 2016 | Ramirez, I. and Paiva, V. H. and Fagundes, I. and Menezes, D. and Silva, I. and Ceia, F. R. and Phillips, R. A. and Ramos, J. A. and Garthe, S. | Animal Conservation | 10.1111/acv.12227 | ft025 |
| es053 | p020 | c033 | s035 | Desertas petrel | Pterodroma deserta | Seabird | B | 7 | 1.70 | ICC | 0.9000 | N | NA | yes | GLS | Depart\_breed | Bugio Island, Madeira | Europe | -16.480000 | 32.43000 | Breeding |  | Table 2, p. 146 | text | Conservation implications of consistent foraging and trophic ecology in a rare petrel species | 2016 | Ramirez, I. and Paiva, V. H. and Fagundes, I. and Menezes, D. and Silva, I. and Ceia, F. R. and Phillips, R. A. and Ramos, J. A. and Garthe, S. | Animal Conservation | 10.1111/acv.12227 | ft025 |
| es054 | p020 | c034 | s035 | Desertas petrel | Pterodroma deserta | Seabird | B | 4 | 2.00 | ICC | 0.9100 | N | NA | yes | GLS | Depart\_breed | Bugio Island, Madeira | Europe | -16.480000 | 32.43000 | Breeding |  | Table 2, p. 146 | text | Conservation implications of consistent foraging and trophic ecology in a rare petrel species | 2016 | Ramirez, I. and Paiva, V. H. and Fagundes, I. and Menezes, D. and Silva, I. and Ceia, F. R. and Phillips, R. A. and Ramos, J. A. and Garthe, S. | Animal Conservation | 10.1111/acv.12227 | ft025 |
| es055 | p020 | c033 | s035 | Desertas petrel | Pterodroma deserta | Seabird | B | 7 | 1.70 | ICC | 0.9200 | N | NA | yes | GLS | Arrival\_breed | Bugio Island, Madeira | Europe | -16.480000 | 32.43000 | Breeding |  | Table 2, p. 146 | text | Conservation implications of consistent foraging and trophic ecology in a rare petrel species | 2016 | Ramirez, I. and Paiva, V. H. and Fagundes, I. and Menezes, D. and Silva, I. and Ceia, F. R. and Phillips, R. A. and Ramos, J. A. and Garthe, S. | Animal Conservation | 10.1111/acv.12227 | ft025 |
| es056 | p020 | c034 | s035 | Desertas petrel | Pterodroma deserta | Seabird | B | 4 | 2.00 | ICC | 0.9500 | N | NA | yes | GLS | Arrival\_breed | Bugio Island, Madeira | Europe | -16.480000 | 32.43000 | Breeding |  | Table 2, p. 146 | text | Conservation implications of consistent foraging and trophic ecology in a rare petrel species | 2016 | Ramirez, I. and Paiva, V. H. and Fagundes, I. and Menezes, D. and Silva, I. and Ceia, F. R. and Phillips, R. A. and Ramos, J. A. and Garthe, S. | Animal Conservation | 10.1111/acv.12227 | ft025 |
| es057 | p021 | c035 | s030 | Dusky warbler | Phylloscopus fuscatus | Landbird | M | 12 | 2.00 | ICC | 0.3400 | N | NA | no | Conventional | Arrival\_breed | Malkachan river, Russian Far East | Europe | 154.230000 | 59.85000 | Breeding |  | p. 6, text, results | text | Benefits of early arrival at breeding grounds vary between males | 2002 | Forstmeier, W. | Journal of Animal Ecology | 10.1046/j.0021-8790.2001.00569.x | ft018 |
| es058 | p022 | c036 | s043 | Eastern kingbird | Tyrannus tyrannus | Landbird | M | 30 | 2.50 | ICC | 0.0500 | N | NA | no | Conventional | Arrival\_breed | Malheur National Wildlife Refuge, Oregon | North America | -118.900000 | 42.81600 | Breeding |  | p. 38, text, results | text | Age- and sex-dependent spring arrival dates of Eastern Kingbirds | 2009 | Cooper, N. W. and Murphy, M. T. and Redmond, L. J. | Journal of Field Ornithology | 10.1111/j.1557-9263.2009.00203.x | ft006 |
| es059 | p022 | c037 | s043 | Eastern kingbird | Tyrannus tyrannus | Landbird | M | 20 | 2.30 | ICC | 0.0500 | N | NA | no | Conventional | Arrival\_breed | Malheur National Wildlife Refuge, Oregon | North America | -118.900000 | 42.81600 | Breeding |  | p. 38, text, results | text | Age- and sex-dependent spring arrival dates of Eastern Kingbirds | 2009 | Cooper, N. W. and Murphy, M. T. and Redmond, L. J. | Journal of Field Ornithology | 10.1111/j.1557-9263.2009.00203.x | ft006 |
| es060 | p022 | c038 | s043 | Eastern kingbird | Tyrannus tyrannus | Landbird | F | 26 | 2.30 | ICC | 0.2300 | N | NA | no | Conventional | Arrival\_breed | Malheur National Wildlife Refuge, Oregon | North America | -118.900000 | 42.81600 | Breeding |  | p. 38, text, results | text | Age- and sex-dependent spring arrival dates of Eastern Kingbirds | 2009 | Cooper, N. W. and Murphy, M. T. and Redmond, L. J. | Journal of Field Ornithology | 10.1111/j.1557-9263.2009.00203.x | ft006 |
| es061 | p022 | c039 | s043 | Eastern kingbird | Tyrannus tyrannus | Landbird | F | 19 | 2.40 | ICC | 0.3900 | N | NA | no | Conventional | Arrival\_breed | Malheur National Wildlife Refuge, Oregon | North America | -118.900000 | 42.81600 | Breeding |  | p. 38, text, results | text | Age- and sex-dependent spring arrival dates of Eastern Kingbirds | 2009 | Cooper, N. W. and Murphy, M. T. and Redmond, L. J. | Journal of Field Ornithology | 10.1111/j.1557-9263.2009.00203.x | ft006 |
| es062 | p023 | c040 | s026 | Egyptian vulture | Neophron percnopterus | Landbird | B | 6 | 3.80 | ICC | 0.4340 | N | NA | no | Satellite | Nonbreed\_depart | Castell<f3>n and Guadalajara provinces (Spain) | Europe | -1.443056 | 40.43284 | Breeding |  | Table 2, p. 647 | text | Individual repeatability in timing and spatial flexibility of migration routes of trans-Saharan migratory raptors | 2014 | Lopez-Lopez, P. and Garcia-Ripolles, C. and Urios, V. | Current Zoology | 10.1093/czoolo/60.5.642 | ft075 |
| es063 | p023 | c040 | s026 | Egyptian vulture | Neophron percnopterus | Landbird | B | 6 | 3.80 | ICC | 0.5630 | N | NA | no | Satellite | Arrival\_breed | Castell<f3>n and Guadalajara provinces (Spain) | Europe | -1.443056 | 40.43284 | Breeding |  | Table 2, p. 647 | text | Individual repeatability in timing and spatial flexibility of migration routes of trans-Saharan migratory raptors | 2014 | Lopez-Lopez, P. and Garcia-Ripolles, C. and Urios, V. | Current Zoology | 10.1093/czoolo/60.5.642 | ft075 |
| es064 | p023 | c040 | s026 | Egyptian vulture | Neophron percnopterus | Landbird | B | 6 | 4.20 | ICC | 0.7040 | N | NA | no | Satellite | Nonbreed\_arrival | Castell<f3>n and Guadalajara provinces (Spain) | Europe | -1.443056 | 40.43284 | Breeding |  | Table 2, p. 647 | text | Individual repeatability in timing and spatial flexibility of migration routes of trans-Saharan migratory raptors | 2014 | Lopez-Lopez, P. and Garcia-Ripolles, C. and Urios, V. | Current Zoology | 10.1093/czoolo/60.5.642 | ft075 |
| es065 | p023 | c040 | s026 | Egyptian vulture | Neophron percnopterus | Landbird | B | 6 | 4.20 | ICC | 0.7050 | N | NA | no | Satellite | Depart\_breed | Castell<f3>n and Guadalajara provinces (Spain) | Europe | -1.443056 | 40.43284 | Breeding |  | Table 2, p. 647 | text | Individual repeatability in timing and spatial flexibility of migration routes of trans-Saharan migratory raptors | 2014 | Lopez-Lopez, P. and Garcia-Ripolles, C. and Urios, V. | Current Zoology | 10.1093/czoolo/60.5.642 | ft075 |
| es066 | p024 | c041 | s009 | Emperor goose | Chen canagicus | Waterbird | F | 18 | 2.00 | ICC | 0.3410 | N | NA | no | Conventional | Arrival\_breed | Kokechik Bay, YKD, Alaska | North America | -165.730000 | 61.67000 | Breeding |  | Table 2, p. 387 | text | Reproductive Ecology of Emperor Geese - Annual and Individual Variation in Nesting | 1992 | Petersen, M. R. | Condor | 10.2307/1369211 | ft122 |
| es067 | p025 | c042 | s038 | Eurasian woodcock | Scolopax rusticola | Waterbird | B | 5 | 2.80 | ICC | 0.1300 | N | NA | yes | Satellite | Nonbreed\_depart | Italian peninsula | Italy | 14.260000 | 41.53000 | Non-breeding |  | Table 5, p. 161 | text | Interindividual variation and consistency of migratory behavior in the Eurasian woodcock | 2020 | Tedeschi, A. and Sorrenti, M. and Bottazzo, M. and Spagnesi, M. and Telletxea, I. and Ibanez, R. and Tormen, N. and De Pascalis, F. and Guidolin, L. and Rubolini, D. | Current Zoology | 10.1093/cz/zoz038 | ft087 |
| es068 | p025 | c042 | s038 | Eurasian woodcock | Scolopax rusticola | Waterbird | B | 8 | 2.60 | ICC | 0.8900 | N | NA | yes | Satellite | Arrival\_breed | Italian peninsula | Italy | 14.260000 | 41.53000 | Non-breeding |  | Table 5, p. 161 | text | Interindividual variation and consistency of migratory behavior in the Eurasian woodcock | 2020 | Tedeschi, A. and Sorrenti, M. and Bottazzo, M. and Spagnesi, M. and Telletxea, I. and Ibanez, R. and Tormen, N. and De Pascalis, F. and Guidolin, L. and Rubolini, D. | Current Zoology | 10.1093/cz/zoz038 | ft087 |
| es069 | p026 | c043 | s005 | Ferruginous hawk | Buteo regalis | Landbird | B | 25 | 2.70 | ICC | 0.1700 | N | NA | no | Satellite | Depart\_breed | Pacific northwest, northern grasslands, northern plains, Mexico (winter) | North America | -121.540000 | 47.48000 | Both but mainly breeding grounds |  | Table 3, p. 566 | text | Repeatability in migration of Ferruginous Hawks (Buteo regalis) and implications for nomadism | 2019 | Watson, J. W. and Keren, I. N. | Wilson Journal of Ornithology | 10.1676/18-171 | ft118 |
| es070 | p026 | c043 | s005 | Ferruginous hawk | Buteo regalis | Landbird | B | 18 | 2.60 | ICC | 0.2900 | N | NA | no | Satellite | Nonbreed\_arrival | Pacific northwest, northern grasslands, northern plains, Mexico (winter) | North America | -121.540000 | 47.48000 | Both but mainly breeding grounds |  | Table 3, p. 566 | text | Repeatability in migration of Ferruginous Hawks (Buteo regalis) and implications for nomadism | 2019 | Watson, J. W. and Keren, I. N. | Wilson Journal of Ornithology | 10.1676/18-171 | ft118 |
| es071 | p026 | c043 | s005 | Ferruginous hawk | Buteo regalis | Landbird | B | 14 | 2.70 | ICC | 0.3400 | N | NA | no | Satellite | Arrival\_breed | Pacific northwest, northern grasslands, northern plains, Mexico (winter) | North America | -121.540000 | 47.48000 | Both but mainly breeding grounds |  | Table 3, p. 566 | text | Repeatability in migration of Ferruginous Hawks (Buteo regalis) and implications for nomadism | 2019 | Watson, J. W. and Keren, I. N. | Wilson Journal of Ornithology | 10.1676/18-171 | ft118 |
| es072 | p026 | c043 | s005 | Ferruginous hawk | Buteo regalis | Landbird | B | 10 | 2.60 | ICC | 0.5300 | N | NA | no | Satellite | Nonbreed\_depart | Pacific northwest, northern grasslands, northern plains, Mexico (winter) | North America | -121.540000 | 47.48000 | Both but mainly breeding grounds |  | Table 3, p. 566 | text | Repeatability in migration of Ferruginous Hawks (Buteo regalis) and implications for nomadism | 2019 | Watson, J. W. and Keren, I. N. | Wilson Journal of Ornithology | 10.1676/18-171 | ft118 |
| es073 | p027 | c044 | s001 | Great reed warbler | Acrocephalus arundinaceus | Landbird | M | 7 | 2.30 | ICC | -0.3600 | N | NA | no | GLS | Depart\_breed | Lake Kvismaren, Sweden | Europe | 15.400516 | 59.16956 | Breeding |  | p. 96, text, results | text | Individual consistency of long-distance migration in a songbird: significant repeatability of autumn route, stopovers and wintering sites but not in timing of migration | 2017 | Hasselquist, D. and Montras-Janer, T. and Tarka, M. and Hansson, B. | Journal of Avian Biology | 10.1111/jav.01292 | ft073 |
| es074 | p027 | c044 | s001 | Great reed warbler | Acrocephalus arundinaceus | Landbird | M | 4 | 2.30 | ICC | 0.2300 | N | NA | no | GLS | Nonbreed\_depart | Lake Kvismaren, Sweden | Europe | 15.400516 | 59.16956 | Breeding |  | p. 96, text, results | text | Individual consistency of long-distance migration in a songbird: significant repeatability of autumn route, stopovers and wintering sites but not in timing of migration | 2017 | Hasselquist, D. and Montras-Janer, T. and Tarka, M. and Hansson, B. | Journal of Avian Biology | 10.1111/jav.01292 | ft073 |
| es075 | p027 | c044 | s001 | Great reed warbler | Acrocephalus arundinaceus | Landbird | M | 4 | 2.30 | ICC | 0.2500 | N | NA | no | GLS | Arrival\_breed | Lake Kvismaren, Sweden | Europe | 15.400516 | 59.16956 | Breeding |  | p. 97, text, results | text | Individual consistency of long-distance migration in a songbird: significant repeatability of autumn route, stopovers and wintering sites but not in timing of migration | 2017 | Hasselquist, D. and Montras-Janer, T. and Tarka, M. and Hansson, B. | Journal of Avian Biology | 10.1111/jav.01292 | ft073 |
| es076 | p028 | c045 | s002 | Greater snow goose | Anser caerulescens atlanticus | Waterbird | B | 20 | 2.10 | ICC | 0.4200 | N | NA | no | Conventional | Arrival\_breed | Bylot Island migratory bird sanctuary, Canada | North America | -78.490000 | 73.21000 | both? |  | Table 2, p. 4 | text | Individual variation in timing of migration: causes and reproductive consequences in greater snow geese (Anser caerulescens atlanticus) | 2004 | Bety, J. and Giroux, J. F. and Gauthier, G. | Behavioral Ecology and Sociobiology | 10.1007/s00265-004-0840-3 | ft080 |
| es077 | p029 | c046 | s032 | Grey petrel | Procellaria cinerea | Seabird | B | 7 | 4.30 | ICC | 0.4100 | N | NA | no | GLS | Arrival\_breed | Mayes Island, Kerguelen Islands | Not | 69.940000 | -49.47000 | Breeding |  | Table 3, p. 99 | text | Individual Consistency in the Non-Breeding Behavior of a Long-Distance Migrant Seabird, the Grey Petrel Procellaria Cinerea | 2019 | Delord, K. and Barbraud, C. and Pinaud, D. and Ruault, S. and Patrick, S. C. and Weimerskirch, H. | Marine Ornithology |  | ft072 |
| es078 | p029 | c046 | s032 | Grey petrel | Procellaria cinerea | Seabird | B | 7 | 4.30 | ICC | 0.4400 | N | NA | no | GLS | Depart\_breed | Mayes Island, Kerguelen Islands | Not | 69.940000 | -49.47000 | Breeding |  | Table 3, p. 99 | text | Individual Consistency in the Non-Breeding Behavior of a Long-Distance Migrant Seabird, the Grey Petrel Procellaria Cinerea | 2019 | Delord, K. and Barbraud, C. and Pinaud, D. and Ruault, S. and Patrick, S. C. and Weimerskirch, H. | Marine Ornithology |  | ft072 |
| es079 | p029 | c046 | s032 | Grey petrel | Procellaria cinerea | Seabird | B | 7 | 4.30 | ICC | 0.5500 | N | NA | no | GLS | Nonbreed\_arrival | Mayes Island, Kerguelen Islands | Not | 69.940000 | -49.47000 | Breeding |  | Table 3, p. 99 | text | Individual Consistency in the Non-Breeding Behavior of a Long-Distance Migrant Seabird, the Grey Petrel Procellaria Cinerea | 2019 | Delord, K. and Barbraud, C. and Pinaud, D. and Ruault, S. and Patrick, S. C. and Weimerskirch, H. | Marine Ornithology |  | ft072 |
| es080 | p029 | c046 | s032 | Grey petrel | Procellaria cinerea | Seabird | B | 7 | 4.30 | ICC | 0.7000 | N | NA | no | GLS | Nonbreed\_depart | Mayes Island, Kerguelen Islands | Not | 69.940000 | -49.47000 | Breeding |  | Table 3, p. 99 | text | Individual Consistency in the Non-Breeding Behavior of a Long-Distance Migrant Seabird, the Grey Petrel Procellaria Cinerea | 2019 | Delord, K. and Barbraud, C. and Pinaud, D. and Ruault, S. and Patrick, S. C. and Weimerskirch, H. | Marine Ornithology |  | ft072 |
| es081 | p030 | c047 | s044 | Hoopoe | Upupa epops | Landbird | B | 16 | 2.00 | ICC | 0.2400 | N | NA | no | GLS | Nonbreed\_depart | southern Switzerland | Europe | 7.360000 | 46.23000 | Breeding |  | Table 2, p. 8683 | text | Repeatability of individual migration routes, wintering sites, and timing in a long-distance migrant bird | 2016 | van Wijk, R. E. and Bauer, S. and Schaub, M. | Ecology and Evolution | 10.1002/ece3.2578 | ft120 |
| es082 | p030 | c047 | s044 | Hoopoe | Upupa epops | Landbird | B | 12 | 2.00 | ICC | 0.4300 | N | NA | no | GLS | Arrival\_breed | southern Switzerland | Europe | 7.360000 | 46.23000 | Breeding |  | Table 2, p. 8683 | text | Repeatability of individual migration routes, wintering sites, and timing in a long-distance migrant bird | 2016 | van Wijk, R. E. and Bauer, S. and Schaub, M. | Ecology and Evolution | 10.1002/ece3.2578 | ft120 |
| es083 | p030 | c047 | s044 | Hoopoe | Upupa epops | Landbird | B | 16 | 2.00 | ICC | 0.7300 | N | NA | no | GLS | Nonbreed\_arrival | southern Switzerland | Europe | 7.360000 | 46.23000 | Breeding |  | Table 2, p. 8683 | text | Repeatability of individual migration routes, wintering sites, and timing in a long-distance migrant bird | 2016 | van Wijk, R. E. and Bauer, S. and Schaub, M. | Ecology and Evolution | 10.1002/ece3.2578 | ft120 |
| es084 | p030 | c047 | s044 | Hoopoe | Upupa epops | Landbird | B | 14 | 2.00 | ICC | 0.7500 | N | NA | no | GLS | Depart\_breed | southern Switzerland | Europe | 7.360000 | 46.23000 | Breeding |  | Table 2, p. 8683 | text | Repeatability of individual migration routes, wintering sites, and timing in a long-distance migrant bird | 2016 | van Wijk, R. E. and Bauer, S. and Schaub, M. | Ecology and Evolution | 10.1002/ece3.2578 | ft120 |
| es085 | p031 | c048 | s022 | Marbled godwit | Limosa fedoa | Waterbird | B | 5 | 3.20 | ICC | 0.0000 | N | NA | yes | Satellite | Arrival\_breed | Ugashik Bay, Alaska | North America | -157.390000 | 57.51000 | Breeding |  | Figure 3. C, p. 6 | figure | Flexible timing of annual movements across consistently used sites by Marbled Godwits breeding in Alaska | 2019 | Ruthrauff, D. R. and Tibbitts, T. L. and Gill, R. E. | Auk | 10.1093/auk/uky007 | ft054 |
| es086 | p031 | c048 | s022 | Marbled godwit | Limosa fedoa | Waterbird | B | 5 | 3.20 | ICC | 0.2500 | N | NA | yes | Satellite | Nonbreed\_depart | Ugashik Bay, Alaska | North America | -157.390000 | 57.51000 | Breeding |  | Figure 3. C, p. 6 | figure | Flexible timing of annual movements across consistently used sites by Marbled Godwits breeding in Alaska | 2019 | Ruthrauff, D. R. and Tibbitts, T. L. and Gill, R. E. | Auk | 10.1093/auk/uky007 | ft054 |
| es087 | p031 | c048 | s022 | Marbled godwit | Limosa fedoa | Waterbird | B | 5 | 4.00 | ICC | 0.3200 | N | NA | yes | Satellite | Depart\_breed | Ugashik Bay, Alaska | North America | -157.390000 | 57.51000 | Breeding |  | Figure 3. C, p. 6 | figure | Flexible timing of annual movements across consistently used sites by Marbled Godwits breeding in Alaska | 2019 | Ruthrauff, D. R. and Tibbitts, T. L. and Gill, R. E. | Auk | 10.1093/auk/uky007 | ft054 |
| es088 | p031 | c048 | s022 | Marbled godwit | Limosa fedoa | Waterbird | B | 5 | 4.00 | ICC | 0.4500 | N | NA | yes | Satellite | Nonbreed\_arrival | Ugashik Bay, Alaska | North America | -157.390000 | 57.51000 | Breeding |  | Figure 3. C, p. 6 | figure | Flexible timing of annual movements across consistently used sites by Marbled Godwits breeding in Alaska | 2019 | Ruthrauff, D. R. and Tibbitts, T. L. and Gill, R. E. | Auk | 10.1093/auk/uky007 | ft054 |
| es089 | p032 | c049 | s011 | Marsh harrier | Circus aeruginosus | Landbird | B | 4 | 4.80 | ICC | 0.3500 | N | NA | yes | Satellite | Depart\_breed | Bergslagen, Lindesberg, Sweden | Europe | 15.161838 | 58.79720 | Breeding |  | Table 3, p. 181 | text | Consistency in long-distance bird migration: contrasting patterns in time and space for two raptors | 2016 | Vardanis, Y. and Nilsson, J. A. and Klaassen, R. H. G. and Strandberg, R. and Alerstam, T. | Animal Behaviour | 10.1016/j.anbehav.2015.12.014 | ft026 |
| es090 | p032 | c049 | s011 | Marsh harrier | Circus aeruginosus | Landbird | B | 4 | 4.80 | ICC | 0.6000 | N | NA | yes | Satellite | Nonbreed\_arrival | Bergslagen, Lindesberg, Sweden | Europe | 15.161838 | 58.79720 | Breeding |  | Table 3, p. 181 | text | Consistency in long-distance bird migration: contrasting patterns in time and space for two raptors | 2016 | Vardanis, Y. and Nilsson, J. A. and Klaassen, R. H. G. and Strandberg, R. and Alerstam, T. | Animal Behaviour | 10.1016/j.anbehav.2015.12.014 | ft026 |
| es091 | p032 | c049 | s011 | Marsh harrier | Circus aeruginosus | Landbird | B | 3 | 5.00 | ICC | 0.6300 | N | NA | yes | Satellite | Arrival\_breed | Bergslagen, Lindesberg, Sweden | Europe | 15.161838 | 58.79720 | Breeding |  | Table 3, p. 181 | text | Consistency in long-distance bird migration: contrasting patterns in time and space for two raptors | 2016 | Vardanis, Y. and Nilsson, J. A. and Klaassen, R. H. G. and Strandberg, R. and Alerstam, T. | Animal Behaviour | 10.1016/j.anbehav.2015.12.014 | ft026 |
| es092 | p032 | c049 | s011 | Marsh harrier | Circus aeruginosus | Landbird | B | 3 | 5.00 | ICC | 0.8100 | N | NA | yes | Satellite | Nonbreed\_depart | Bergslagen, Lindesberg, Sweden | Europe | 15.161838 | 58.79720 | Breeding |  | Table 3, p. 181 | text | Consistency in long-distance bird migration: contrasting patterns in time and space for two raptors | 2016 | Vardanis, Y. and Nilsson, J. A. and Klaassen, R. H. G. and Strandberg, R. and Alerstam, T. | Animal Behaviour | 10.1016/j.anbehav.2015.12.014 | ft026 |
| es093 | p033 | c050 | s025 | Northern gannet | Morus bassanus | Seabird | B | 16 | 2.00 | ICC | 0.0100 | N | NA | no | GLS | Depart\_breed | Bonaventure Island | North America | -64.150000 | 48.48300 | Breeding |  | Table 5, p. 29 | text | Migratory tactics and wintering areas of northern gannets (morus bassanus) breeding in North America | 2014 | Fifield, D. A. and Montevecchi, W. A. and Garthe, S. and Robertson, G. J. and Kubetzki, U. and Rail, J. F. | Ornithological Monographs |  | ft109 |
| es094 | p033 | c050 | s025 | Northern gannet | Morus bassanus | Seabird | B | 14 | 2.00 | ICC | 0.0100 | N | NA | no | GLS | Arrival\_breed | Bonaventure Island | North America | -64.150000 | 48.48300 | Breeding |  | Table 5, p. 29 | text | Migratory tactics and wintering areas of northern gannets (morus bassanus) breeding in North America | 2014 | Fifield, D. A. and Montevecchi, W. A. and Garthe, S. and Robertson, G. J. and Kubetzki, U. and Rail, J. F. | Ornithological Monographs |  | ft109 |
| es095 | p033 | c050 | s025 | Northern gannet | Morus bassanus | Seabird | B | 16 | 2.00 | ICC | 0.4200 | N | NA | no | GLS | Nonbreed\_arrival | Bonaventure Island | North America | -64.150000 | 48.48300 | Breeding |  | Table 5, p. 29 | text | Migratory tactics and wintering areas of northern gannets (morus bassanus) breeding in North America | 2014 | Fifield, D. A. and Montevecchi, W. A. and Garthe, S. and Robertson, G. J. and Kubetzki, U. and Rail, J. F. | Ornithological Monographs |  | ft109 |
| es096 | p033 | c050 | s025 | Northern gannet | Morus bassanus | Seabird | B | 14 | 2.00 | ICC | 0.9000 | N | NA | no | GLS | Nonbreed\_depart | Bonaventure Island | North America | -64.150000 | 48.48300 | Breeding |  | Table 5, p. 29 | text | Migratory tactics and wintering areas of northern gannets (morus bassanus) breeding in North America | 2014 | Fifield, D. A. and Montevecchi, W. A. and Garthe, S. and Robertson, G. J. and Kubetzki, U. and Rail, J. F. | Ornithological Monographs |  | ft109 |
| es097 | p032 | c051 | s028 | Osprey | Pandion haliaetus | Landbird | B | 7 | 2.90 | ICC | 0.0400 | N | NA | yes | Satellite | Nonbreed\_arrival | Bergslagen, Lindesberg, Sweden | Europe | 15.161838 | 58.79720 | Breeding |  | Table 3, p. 181 | text | Consistency in long-distance bird migration: contrasting patterns in time and space for two raptors | 2016 | Vardanis, Y. and Nilsson, J. A. and Klaassen, R. H. G. and Strandberg, R. and Alerstam, T. | Animal Behaviour | 10.1016/j.anbehav.2015.12.014 | ft026 |
| es098 | p032 | c051 | s028 | Osprey | Pandion haliaetus | Landbird | B | 4 | 3.30 | ICC | 0.0700 | N | NA | yes | Satellite | Arrival\_breed | Bergslagen, Lindesberg, Sweden | Europe | 15.161838 | 58.79720 | Breeding |  | Table 3, p. 181 | text | Consistency in long-distance bird migration: contrasting patterns in time and space for two raptors | 2016 | Vardanis, Y. and Nilsson, J. A. and Klaassen, R. H. G. and Strandberg, R. and Alerstam, T. | Animal Behaviour | 10.1016/j.anbehav.2015.12.014 | ft026 |
| es099 | p032 | c051 | s028 | Osprey | Pandion haliaetus | Landbird | B | 7 | 2.90 | ICC | 0.1700 | N | NA | yes | Satellite | Depart\_breed | Bergslagen, Lindesberg, Sweden | Europe | 15.161838 | 58.79720 | Breeding |  | Table 3, p. 181 | text | Consistency in long-distance bird migration: contrasting patterns in time and space for two raptors | 2016 | Vardanis, Y. and Nilsson, J. A. and Klaassen, R. H. G. and Strandberg, R. and Alerstam, T. | Animal Behaviour | 10.1016/j.anbehav.2015.12.014 | ft026 |
| es100 | p032 | c051 | s028 | Osprey | Pandion haliaetus | Landbird | B | 4 | 3.30 | ICC | 0.3800 | N | NA | yes | Satellite | Nonbreed\_depart | Bergslagen, Lindesberg, Sweden | Europe | 15.161838 | 58.79720 | Breeding |  | Table 3, p. 181 | text | Consistency in long-distance bird migration: contrasting patterns in time and space for two raptors | 2016 | Vardanis, Y. and Nilsson, J. A. and Klaassen, R. H. G. and Strandberg, R. and Alerstam, T. | Animal Behaviour | 10.1016/j.anbehav.2015.12.014 | ft026 |
| es101 | p034 | c052 | s020 | Pallas gull | Larus ichthyaetus | Seabird | B | 4 | 3.00 | ICC | -0.0700 | N | NA | no | Satellite | Depart\_breed | Qinghai Lake, China | Not | 100.000000 | 36.88000 | Breeding |  | Table 2, p. 10 | text | Detours in long-distance migration across the Qinghai-Tibetan Plateau: Individual consistency and habitat associations | 2018 | Liu, D. and Zhang, G. and Jiang, H. and Lu, J. | PeerJ | 10.7717/peerj.4304 | ft035 |
| es102 | p034 | c052 | s020 | Pallas gull | Larus ichthyaetus | Seabird | B | 4 | 2.50 | ICC | 0.7800 | N | NA | no | Satellite | Arrival\_breed | Qinghai Lake, China | Not | 100.000000 | 36.88000 | Breeding |  | Table 2, p. 10 | text | Detours in long-distance migration across the Qinghai-Tibetan Plateau: Individual consistency and habitat associations | 2018 | Liu, D. and Zhang, G. and Jiang, H. and Lu, J. | PeerJ | 10.7717/peerj.4304 | ft035 |
| es103 | p034 | c052 | s020 | Pallas gull | Larus ichthyaetus | Seabird | B | 4 | 2.50 | ICC | 0.8400 | N | NA | no | Satellite | Nonbreed\_depart | Qinghai Lake, China | Not | 100.000000 | 36.88000 | Breeding |  | Table 2, p. 10 | text | Detours in long-distance migration across the Qinghai-Tibetan Plateau: Individual consistency and habitat associations | 2018 | Liu, D. and Zhang, G. and Jiang, H. and Lu, J. | PeerJ | 10.7717/peerj.4304 | ft035 |
| es104 | p034 | c052 | s020 | Pallas gull | Larus ichthyaetus | Seabird | B | 4 | 3.00 | ICC | 0.8500 | N | NA | no | Satellite | Nonbreed\_arrival | Qinghai Lake, China | Not | 100.000000 | 36.88000 | Breeding |  | Table 2, p. 10 | text | Detours in long-distance migration across the Qinghai-Tibetan Plateau: Individual consistency and habitat associations | 2018 | Liu, D. and Zhang, G. and Jiang, H. and Lu, J. | PeerJ | 10.7717/peerj.4304 | ft035 |
| es105 | p035 | c053 | s014 | Pied flycatcher | Ficedula hypoleuca | Landbird | F | 116 | 2.30 | ICC | 0.0780 | Y | Age | no | Conventional | Arrival\_breed | Sinober in S<f8>rkedalen | Europe | 10.633000 | 59.98300 | Breeding |  | p. 992, text, results | text | Advancement of spring arrival in a long-term study of a passerine bird: sex, age and environmental effects | 2017 | Cadahia, L. and Labra, A. and Knudsen, E. and Nilsson, A. and Lampe, H. M. and Slagsvold, T. and Stenseth, N. C. | Oecologia | 10.1007/s00442-017-3922-4 | ft002 |
| es106 | p036 | c056 | s014 | Pied flycatcher | Ficedula hypoleuca | Landbird | M | 39 | 2.30 | ICC | 0.0900 | N | NA |  | Conventional | Arrival\_breed | La Hiruela, Spain | Europe | -3.450000 | 41.06600 | Breeding |  | p. 704, text, results | text | Arrival time from spring migration in male Pied Flycatchers: Individual consistency and familial resemblance | 1998 | Potti, J. | Condor | 10.2307/1369752 | ft016 |
| es107 | p035 | c054 | s014 | Pied flycatcher | Ficedula hypoleuca | Landbird | M | 364 | 2.70 | ICC | 0.1670 | Y | Age | no | Conventional | Arrival\_breed | Sinober in S<f8>rkedalen | Europe | 10.633000 | 59.98300 | Breeding |  | p. 992, text, results | text | Advancement of spring arrival in a long-term study of a passerine bird: sex, age and environmental effects | 2017 | Cadahia, L. and Labra, A. and Knudsen, E. and Nilsson, A. and Lampe, H. M. and Slagsvold, T. and Stenseth, N. C. | Oecologia | 10.1007/s00442-017-3922-4 | ft002 |
| es108 | p035 | c055 | s014 | Pied flycatcher | Ficedula hypoleuca | Landbird | B | 480 | 2.60 | ICC | 0.2120 | Y | Age | no | Conventional | Arrival\_breed | Sinober in S<f8>rkedalen | Europe | 10.633000 | 59.98300 | Breeding |  | p. 992, text, results | text | Advancement of spring arrival in a long-term study of a passerine bird: sex, age and environmental effects | 2017 | Cadahia, L. and Labra, A. and Knudsen, E. and Nilsson, A. and Lampe, H. M. and Slagsvold, T. and Stenseth, N. C. | Oecologia | 10.1007/s00442-017-3922-4 | ft002 |
| es109 | p037 | c057 | s014 | Pied flycatcher | Ficedula hypoleuca | Landbird | M | 307 | 2.31 | ICC | 0.2700 | N | NA | no | Conventional | Arrival\_breed | Drenthe, The Netherlands | Europe | 6.360000 | 52.81600 | Breeding |  | Table 3, p. 14 | text | Repeatability in spring arrival dates in Pied Flycatchers varies among years and sexes | 2016 | Both, C. and Bijlsma, R. G. and Ouwehand, J. | Ardea | 10.5253/arde.v104i1.a1 | ft119 |
| es110 | p037 | c058 | s014 | Pied flycatcher | Ficedula hypoleuca | Landbird | F | 221 | 2.39 | ICC | 0.3000 | N | NA | no | Conventional | Arrival\_breed | Drenthe, The Netherlands | Europe | 6.360000 | 52.81600 | Breeding |  | Table 3, p. 14 | text | Repeatability in spring arrival dates in Pied Flycatchers varies among years and sexes | 2016 | Both, C. and Bijlsma, R. G. and Ouwehand, J. | Ardea | 10.5253/arde.v104i1.a1 | ft119 |
| es111 | p038 | c059 | s033 | Purple martin | Progne subis | Landbird | B | 33 | 2.00 | ICC | 0.0009 | Y | Sex, age (fixed), breeding colony, individual, year (random) | no | GLS | Nonbreed\_arrival |  | US | -80.090000 | 41.80000 | Breeding |  | Table 1, p. 3 | text | Individual Variability in Migration Timing Can Explain Long-Term, Population-Level Advances in a Songbird | 2019 | Fraser, K. C. and Shave, A. and de Greef, E. and Siegrist, J. and Garroway, C. J. | Frontiers in Ecology and Evolution | 10.3389/fevo.2019.00324 | ft077 |
| es112 | p038 | c059 | s033 | Purple martin | Progne subis | Landbird | B | 33 | 2.00 | ICC | 0.0010 | Y | Sex, age (fixed), breeding colony, individual, year (random) | no | GLS | Depart\_breed |  | US | -80.090000 | 41.80000 | Breeding |  | Table 1, p. 3 | text | Individual Variability in Migration Timing Can Explain Long-Term, Population-Level Advances in a Songbird | 2019 | Fraser, K. C. and Shave, A. and de Greef, E. and Siegrist, J. and Garroway, C. J. | Frontiers in Ecology and Evolution | 10.3389/fevo.2019.00324 | ft077 |
| es113 | p038 | c059 | s033 | Purple martin | Progne subis | Landbird | B | 33 | 2.00 | ICC | 0.3200 | Y | Sex, age (fixed), breeding colony, individual, year (random) | no | GLS | Arrival\_breed |  | US | -80.090000 | 41.80000 | Breeding |  | Table 1, p. 3 | text | Individual Variability in Migration Timing Can Explain Long-Term, Population-Level Advances in a Songbird | 2019 | Fraser, K. C. and Shave, A. and de Greef, E. and Siegrist, J. and Garroway, C. J. | Frontiers in Ecology and Evolution | 10.3389/fevo.2019.00324 | ft077 |
| es114 | p038 | c059 | s033 | Purple martin | Progne subis | Landbird | B | 33 | 2.00 | ICC | 0.3900 | Y | Sex, age (fixed), breeding colony, individual, year (random) | no | GLS | Nonbreed\_depart |  | US | -80.090000 | 41.80000 | Breeding |  | Table 1, p. 3 | text | Individual Variability in Migration Timing Can Explain Long-Term, Population-Level Advances in a Songbird | 2019 | Fraser, K. C. and Shave, A. and de Greef, E. and Siegrist, J. and Garroway, C. J. | Frontiers in Ecology and Evolution | 10.3389/fevo.2019.00324 | ft077 |
| es115 | p039 | c060 | s018 | Red-backed shrike | Lanius collurio | Landbird | M | 7 | 2.00 | ICC | 0.3800 | N | NA | no | GLS | Depart\_breed | Gribskov, Denmark | Europe | 12.281090 | 55.96581 | Breeding |  | Table 1, p. 139 | text | Full-year tracking suggests endogenous control of migration timing in a long-distance migratory songbird | 2018 | Pedersen, L. and Jackson, K. and Thorup, K. and Tottrup, A. P. | Behavioral Ecology and Sociobiology | 10.1007/s00265-018-2553-z | ft055 |
| es116 | p040 | c061 | s034 | Round Island petrel | Pterodroma arminjoniana | Seabird | B | 76 | 2.20 | ICC | 0.7870 | N | NA | yes | GLS | Depart\_breed | Round Island | Mauritius | 57.780000 | -19.85000 | Breeding |  | NA | NA | Individual consistency in migration strategies of a tropical seabird, the Round Island petrel | 2022 | Franklin, K. A. and Norris, K. and Gill, J. A. and Ratcliffe, N. and Bonnet-Lebrun A-S. and Butler, S. J. and Cole. N. C. and Jones, C. G. and Lisovski, S. and Ruhomaun, K. and Tatayah, V. Nicoll, M. A. C. | Movement Ecology | NA | NA |
| es117 | p040 | c061 | s034 | Round Island petrel | Pterodroma arminjoniana | Seabird | B | 62 | 2.10 | ICC | 0.8130 | N | NA | yes | GLS | Arrival\_breed | Round Island | Mauritius | 57.780000 | -19.85000 | Breeding |  | NA | NA | Individual consistency in migration strategies of a tropical seabird, the Round Island petrel | 2022 | Franklin, K. A. and Norris, K. and Gill, J. A. and Ratcliffe, N. and Bonnet-Lebrun A-S. and Butler, S. J. and Cole. N. C. and Jones, C. G. and Lisovski, S. and Ruhomaun, K. and Tatayah, V. Nicoll, M. A. C. | Movement Ecology | MA | NA |
| es118 | p041 | c062 | s006 | Scopoli’s shearwater | Calonectris diomedea | Seabird | F | 5 | 2.20 | ICC | 0.0000 | N | NA | no | GLS | Arrival\_breed | Linosa, Sicily | Europe | 12.868000 | 35.86400 | Breeding |  | Table 3, p. 635 | text | Individual consistency and sex differences in migration strategies of Scopoli’s shearwaters Calonectris diomedea despite year differences | 2014 | Muller, M. S. and Massa, B. and Phillips, R. A. and Dell’Omo, G. | Current Zoology | 10.1093/czoolo/60.5.631 | ft069 |
| es119 | p041 | c063 | s006 | Scopoli’s shearwater | Calonectris diomedea | Seabird | M | 7 | 2.10 | ICC | 0.1990 | N | NA | no | GLS | Arrival\_breed | Linosa, Sicily | Europe | 12.868000 | 35.86400 | Breeding | Unsure if they have fixed effect | Table 3, p. 635 | text | Individual consistency and sex differences in migration strategies of Scopoli’s shearwaters Calonectris diomedea despite year differences | 2014 | Muller, M. S. and Massa, B. and Phillips, R. A. and Dell’Omo, G. | Current Zoology | 10.1093/czoolo/60.5.631 | ft069 |
| es120 | p041 | c062 | s006 | Scopoli’s shearwater | Calonectris diomedea | Seabird | F | 5 | 2.20 | ICC | 0.3560 | N | NA | no | GLS | Depart\_breed | Linosa, Sicily | Europe | 12.868000 | 35.86400 | Breeding |  | Table 3, p. 635 | text | Individual consistency and sex differences in migration strategies of Scopoli’s shearwaters Calonectris diomedea despite year differences | 2014 | Muller, M. S. and Massa, B. and Phillips, R. A. and Dell’Omo, G. | Current Zoology | 10.1093/czoolo/60.5.631 | ft069 |
| es121 | p041 | c063 | s006 | Scopoli’s shearwater | Calonectris diomedea | Seabird | M | 7 | 2.10 | ICC | 0.4160 | N | NA | no | GLS | Depart\_breed | Linosa, Sicily | Europe | 12.868000 | 35.86400 | Breeding |  | Table 3, p. 635 | text | Individual consistency and sex differences in migration strategies of Scopoli’s shearwaters Calonectris diomedea despite year differences | 2014 | Muller, M. S. and Massa, B. and Phillips, R. A. and Dell’Omo, G. | Current Zoology | 10.1093/czoolo/60.5.631 | ft069 |
| es122 | p042 | c064 | s007 | Streaked shearwater | Calonectris leucomelas | Seabird | F | 10 | 2.00 | r | -0.4800 | N | NA | NA | GLS | Depart\_breed | Sangan Island, Mikura Island and Awa Island, Japan | Not | 141.980000 | 39.31000 | Breeding |  | Figure 3. A, p. 694 | text | Individual consistency in migratory behaviour of a pelagic seabird | 2014 | Yamamoto, T. and Takahashi, A. and Sato, K. and Oka, N. and Yamamoto, M. and Trathan, P. N. | Behaviour | 10.1163/1568539X-00003163 | ft070 |
| es123 | p042 | c065 | s007 | Streaked shearwater | Calonectris leucomelas | Seabird | M | 12 | 2.00 | r | -0.4100 | N | NA | NA | GLS | Depart\_breed | Sangan Island, Mikura Island and Awa Island, Japan | Not | 141.980000 | 39.31000 | Breeding |  | Figure 3. A, p. 694 | text | Individual consistency in migratory behaviour of a pelagic seabird | 2014 | Yamamoto, T. and Takahashi, A. and Sato, K. and Oka, N. and Yamamoto, M. and Trathan, P. N. | Behaviour | 10.1163/1568539X-00003163 | ft070 |
| es124 | p042 | c066 | s007 | Streaked shearwater | Calonectris leucomelas | Seabird | M | 11 | 2.00 | r | -0.3000 | N | NA | NA | GLS | Depart\_breed | Sangan Island, Mikura Island and Awa Island, Japan | Not | 141.980000 | 39.31000 | Breeding |  | Figure 3. A, p. 694 | text | Individual consistency in migratory behaviour of a pelagic seabird | 2014 | Yamamoto, T. and Takahashi, A. and Sato, K. and Oka, N. and Yamamoto, M. and Trathan, P. N. | Behaviour | 10.1163/1568539X-00003163 | ft070 |
| es125 | p042 | c065 | s007 | Streaked shearwater | Calonectris leucomelas | Seabird | M | 13 | 2.00 | r | -0.2400 | N | NA | NA | GLS | Arrival\_breed | Sangan Island, Mikura Island and Awa Island, Japan | Not | 141.980000 | 39.31000 | Breeding |  | Figure 3. D, p. 694 | text | Individual consistency in migratory behaviour of a pelagic seabird | 2014 | Yamamoto, T. and Takahashi, A. and Sato, K. and Oka, N. and Yamamoto, M. and Trathan, P. N. | Behaviour | 10.1163/1568539X-00003163 | ft070 |
| es126 | p042 | c067 | s007 | Streaked shearwater | Calonectris leucomelas | Seabird | F | 11 | 2.00 | r | -0.1500 | N | NA | NA | GLS | Depart\_breed | Sangan Island, Mikura Island and Awa Island, Japan | Not | 141.980000 | 39.31000 | Breeding |  | Figure 3. A, p. 694 | text | Individual consistency in migratory behaviour of a pelagic seabird | 2014 | Yamamoto, T. and Takahashi, A. and Sato, K. and Oka, N. and Yamamoto, M. and Trathan, P. N. | Behaviour | 10.1163/1568539X-00003163 | ft070 |
| es127 | p042 | c066 | s007 | Streaked shearwater | Calonectris leucomelas | Seabird | M | 9 | 2.00 | r | 0.0800 | N | NA | NA | GLS | Arrival\_breed | Sangan Island, Mikura Island and Awa Island, Japan | Not | 141.980000 | 39.31000 | Breeding |  | Figure 3. D, p. 694 | text | Individual consistency in migratory behaviour of a pelagic seabird | 2014 | Yamamoto, T. and Takahashi, A. and Sato, K. and Oka, N. and Yamamoto, M. and Trathan, P. N. | Behaviour | 10.1163/1568539X-00003163 | ft070 |
| es128 | p042 | c064 | s007 | Streaked shearwater | Calonectris leucomelas | Seabird | F | 10 | 2.00 | r | 0.1800 | N | NA | NA | GLS | Arrival\_breed | Sangan Island, Mikura Island and Awa Island, Japan | Not | 141.980000 | 39.31000 | Breeding |  | Figure 3. D, p. 694 | text | Individual consistency in migratory behaviour of a pelagic seabird | 2014 | Yamamoto, T. and Takahashi, A. and Sato, K. and Oka, N. and Yamamoto, M. and Trathan, P. N. | Behaviour | 10.1163/1568539X-00003163 | ft070 |
| es129 | p042 | c067 | s007 | Streaked shearwater | Calonectris leucomelas | Seabird | F | 9 | 2.00 | r | 0.9500 | N | NA | NA | GLS | Arrival\_breed | Sangan Island, Mikura Island and Awa Island, Japan | Not | 141.980000 | 39.31000 | Breeding |  | Figure 3. D, p. 694 | text | Individual consistency in migratory behaviour of a pelagic seabird | 2014 | Yamamoto, T. and Takahashi, A. and Sato, K. and Oka, N. and Yamamoto, M. and Trathan, P. N. | Behaviour | 10.1163/1568539X-00003163 | ft070 |
| es130 | p017 | c024 | s046 | Thick-billed murre | Uria lomvia | Seabird | B | 3 | 1.30 | ICC | 0.0000 | N | NA | no | GLS | Arrival\_breed | 7 seabird colonies ranging 74 to 47 N | North America | -82.970000 | 62.54000 | Breeding |  | Table 2, p. 7 | text | Individual winter movement strategies in two species of murre (Uria spp.) in the Northwest Atlantic | 2014 | McFarlane Tranquilla, L. A. and Montevecchi, W. A. and Fifield, D. A. and Hedd, A. and Gaston, A. J. and Robertson, G. J. and Phillips, R. A. | PLoS ONE | 10.1371/journal.pone.0090583 | ft081 |
| es131 | p017 | c025 | s046 | Thick-billed murre | Uria lomvia | Seabird | B | 8 | 2.10 | ICC | 0.0100 | N | NA | no | GLS | Depart\_breed | 7 seabird colonies ranging 74 to 47 N | North America | -82.970000 | 62.54000 | Breeding |  | Table 2, p. 7 | text | Individual winter movement strategies in two species of murre (Uria spp.) in the Northwest Atlantic | 2014 | McFarlane Tranquilla, L. A. and Montevecchi, W. A. and Fifield, D. A. and Hedd, A. and Gaston, A. J. and Robertson, G. J. and Phillips, R. A. | PLoS ONE | 10.1371/journal.pone.0090583 | ft081 |
| es132 | p017 | c026 | s046 | Thick-billed murre | Uria lomvia | Seabird | B | 7 | 1.40 | ICC | 0.0100 | N | NA | no | GLS | Depart\_breed | 7 seabird colonies ranging 74 to 47 N | North America | -82.970000 | 62.54000 | Breeding |  | Table 2, p. 7 | text | Individual winter movement strategies in two species of murre (Uria spp.) in the Northwest Atlantic | 2014 | McFarlane Tranquilla, L. A. and Montevecchi, W. A. and Fifield, D. A. and Hedd, A. and Gaston, A. J. and Robertson, G. J. and Phillips, R. A. | PLoS ONE | 10.1371/journal.pone.0090583 | ft081 |
| es133 | p017 | c024 | s046 | Thick-billed murre | Uria lomvia | Seabird | B | 3 | 2.00 | ICC | 0.0100 | N | NA | no | GLS | Depart\_breed | 7 seabird colonies ranging 74 to 47 N | North America | -82.970000 | 62.54000 | Breeding |  | Table 2, p. 7 | text | Individual winter movement strategies in two species of murre (Uria spp.) in the Northwest Atlantic | 2014 | McFarlane Tranquilla, L. A. and Montevecchi, W. A. and Fifield, D. A. and Hedd, A. and Gaston, A. J. and Robertson, G. J. and Phillips, R. A. | PLoS ONE | 10.1371/journal.pone.0090583 | ft081 |
| es134 | p017 | c026 | s046 | Thick-billed murre | Uria lomvia | Seabird | B | 7 | 2.00 | ICC | 0.0400 | N | NA | no | GLS | Arrival\_breed | 7 seabird colonies ranging 74 to 47 N | North America | -82.970000 | 62.54000 | Breeding |  | Table 2, p. 7 | text | Individual winter movement strategies in two species of murre (Uria spp.) in the Northwest Atlantic | 2014 | McFarlane Tranquilla, L. A. and Montevecchi, W. A. and Fifield, D. A. and Hedd, A. and Gaston, A. J. and Robertson, G. J. and Phillips, R. A. | PLoS ONE | 10.1371/journal.pone.0090583 | ft081 |
| es135 | p017 | c025 | s046 | Thick-billed murre | Uria lomvia | Seabird | B | 8 | 1.50 | ICC | 0.1800 | N | NA | no | GLS | Arrival\_breed | 7 seabird colonies ranging 74 to 47 N | North America | -82.970000 | 62.54000 | Breeding |  | Table 2, p. 7 | text | Individual winter movement strategies in two species of murre (Uria spp.) in the Northwest Atlantic | 2014 | McFarlane Tranquilla, L. A. and Montevecchi, W. A. and Fifield, D. A. and Hedd, A. and Gaston, A. J. and Robertson, G. J. and Phillips, R. A. | PLoS ONE | 10.1371/journal.pone.0090583 | ft081 |
| es136 | p043 | c068 | s027 | Whimbrel | Numenius phaeopus | Waterbird | B | 12 | 2.60 | ICC | 0.2300 | N | NA | no | GLS | Arrival\_breed | Iceland | Europe | -20.200000 | 63.80000 | Breeding |  | Table 2, p. 5 | text | Why Are Whimbrels Not Advancing Their Arrival Dates Into Iceland? Exploring Seasonal and Sex-Specific Variation in Consistency of Individual Timing During the Annual Cycle | 2019 | Carneiro, C. and Gunnarsson, T. G. and Alves, J. A. | Frontiers in Ecology and Evolution | 10.3389/fevo.2019.00248 | ft149 |
| es137 | p043 | c068 | s027 | Whimbrel | Numenius phaeopus | Waterbird | B | 16 | 2.90 | ICC | 0.2600 | N | NA | no | GLS | Nonbreed\_arrival | Iceland | Europe | -20.200000 | 63.80000 | Breeding |  | Table 2, p. 5 | text | Why Are Whimbrels Not Advancing Their Arrival Dates Into Iceland? Exploring Seasonal and Sex-Specific Variation in Consistency of Individual Timing During the Annual Cycle | 2019 | Carneiro, C. and Gunnarsson, T. G. and Alves, J. A. | Frontiers in Ecology and Evolution | 10.3389/fevo.2019.00248 | ft149 |
| es138 | p043 | c068 | s027 | Whimbrel | Numenius phaeopus | Waterbird | B | 16 | 2.90 | ICC | 0.2800 | N | NA | no | GLS | Depart\_breed | Iceland | Europe | -20.200000 | 63.80000 | Breeding |  | Table 2, p. 5 | text | Why Are Whimbrels Not Advancing Their Arrival Dates Into Iceland? Exploring Seasonal and Sex-Specific Variation in Consistency of Individual Timing During the Annual Cycle | 2019 | Carneiro, C. and Gunnarsson, T. G. and Alves, J. A. | Frontiers in Ecology and Evolution | 10.3389/fevo.2019.00248 | ft149 |
| es139 | p043 | c068 | s027 | Whimbrel | Numenius phaeopus | Waterbird | B | 12 | 2.60 | ICC | 0.7600 | N | NA | no | GLS | Nonbreed\_depart | Iceland | Europe | -20.200000 | 63.80000 | Breeding |  | Table 2, p. 5 | text | Why Are Whimbrels Not Advancing Their Arrival Dates Into Iceland? Exploring Seasonal and Sex-Specific Variation in Consistency of Individual Timing During the Annual Cycle | 2019 | Carneiro, C. and Gunnarsson, T. G. and Alves, J. A. | Frontiers in Ecology and Evolution | 10.3389/fevo.2019.00248 | ft149 |
| es140 | p044 | c069 | s010 | White stork | Ciconia ciconia | Waterbird | B | 35 | 2.60 | ICC | 0.4900 | N | NA | no | Satellite | Arrival\_breed | Saxony- Anhalt | Germany | 11.500000 | 51.90000 | Breeding |  | p. 1631, text, results | text | Early arrival at breeding grounds: Causes, costs and a trade-off with overwintering latitude | 2018 | Rotics, S. and Kaatz, M. and Turjeman, S. and Zurell, D. and Wikelski, M. and Sapir, N. and Eggers, U. and Fiedler, W. and Jeltsch, F. and Nathan, R. | Journal of Animal Ecology | 10.1111/1365-2656.12898 | ft164 |
| es141 | p044 | c069 | s010 | White stork | Ciconia ciconia | Waterbird | B | 35 | 2.60 | ICC | 0.5100 | N | NA | no | Satellite | Nonbreed\_depart | Saxony- Anhalt | Germany | 11.500000 | 51.90000 | Breeding |  | p. 1631, text, results | text | Early arrival at breeding grounds: Causes, costs and a trade-off with overwintering latitude | 2018 | Rotics, S. and Kaatz, M. and Turjeman, S. and Zurell, D. and Wikelski, M. and Sapir, N. and Eggers, U. and Fiedler, W. and Jeltsch, F. and Nathan, R. | Journal of Animal Ecology | 10.1111/1365-2656.12898 | ft164 |
| es142 | p045 | c070 | s031 | Willow warbler | Phylloscopus trochilus | Landbird | M | 39 | 2.40 | ICC | 0.2690 | N | NA | no | Conventional | Arrival\_breed | Milford Common, Surrey | Europe | -0.650000 | 51.16600 | Breeding |  | Table 2, p. 65 | text | Individual consistency in the arrival dates of territorial male Willow Warblers Phylloscopus trochilus | 2016 | Lawn, M. R. | Ringing and Migration | 10.1080/03078698.2016.1190618 | ft071 |
| es143 | p045 | c071 | s031 | Willow warbler | Phylloscopus trochilus | Landbird | M | 79 | 2.00 | ICC | 0.3380 | N | NA | no | Conventional | Arrival\_breed | Milford Common, Surrey | Europe | -0.650000 | 51.16600 | Breeding |  | Table 2, p. 65 | text | Individual consistency in the arrival dates of territorial male Willow Warblers Phylloscopus trochilus | 2016 | Lawn, M. R. | Ringing and Migration | 10.1080/03078698.2016.1190618 | ft071 |
| es144 | p045 | c072 | s031 | Willow warbler | Phylloscopus trochilus | Landbird | M | 16 | 2.00 | ICC | 0.6450 | N | NA | no | Conventional | Arrival\_breed | Milford Common, Surrey | Europe | -0.650000 | 51.16600 | Breeding |  | Table 2, p. 65 | text | Individual consistency in the arrival dates of territorial male Willow Warblers Phylloscopus trochilus | 2016 | Lawn, M. R. | Ringing and Migration | 10.1080/03078698.2016.1190618 | ft071 |
| es145 | p046 | c073 | s017 | Wood thrush | Hylocichla mustelina | Landbird | B | 8 | 2.00 | ICC | 0.0500 | N | NA | no | GLS | Depart\_breed | Pennsylvania | North America | -79.890000 | 41.79700 | Breeding |  | Table 2, p. 4 | text | Repeat tracking of individual songbirds reveals consistent migration timing but flexibility in route | 2012 | Stanley, C. Q. and MacPherson, M. and Fraser, K. C. and McKinnon, E. A. and Stutchbury, B. J. M. | PLoS ONE | 10.1371/journal.pone.0040688 | ft117 |
| es146 | p046 | c073 | s017 | Wood thrush | Hylocichla mustelina | Landbird | B | 9 | 2.00 | ICC | 0.6200 | N | NA | no | GLS | Nonbreed\_arrival | Pennsylvania | North America | -79.890000 | 41.79700 | Breeding |  | Table 2, p. 4 | text | Repeat tracking of individual songbirds reveals consistent migration timing but flexibility in route | 2012 | Stanley, C. Q. and MacPherson, M. and Fraser, K. C. and McKinnon, E. A. and Stutchbury, B. J. M. | PLoS ONE | 10.1371/journal.pone.0040688 | ft117 |
| es147 | p046 | c073 | s017 | Wood thrush | Hylocichla mustelina | Landbird | B | 9 | 2.00 | ICC | 0.6600 | N | NA | no | GLS | Arrival\_breed | Pennsylvania | North America | -79.890000 | 41.79700 | Breeding |  | Table 2, p. 4 | text | Repeat tracking of individual songbirds reveals consistent migration timing but flexibility in route | 2012 | Stanley, C. Q. and MacPherson, M. and Fraser, K. C. and McKinnon, E. A. and Stutchbury, B. J. M. | PLoS ONE | 10.1371/journal.pone.0040688 | ft117 |
| es148 | p046 | c073 | s017 | Wood thrush | Hylocichla mustelina | Landbird | B | 10 | 2.00 | ICC | 0.7100 | N | NA | no | GLS | Nonbreed\_depart | Pennsylvania | North America | -79.890000 | 41.79700 | Breeding |  | Table 2, p. 4 | text | Repeat tracking of individual songbirds reveals consistent migration timing but flexibility in route | 2012 | Stanley, C. Q. and MacPherson, M. and Fraser, K. C. and McKinnon, E. A. and Stutchbury, B. J. M. | PLoS ONE | 10.1371/journal.pone.0040688 | ft117 |
| es149 | p047 | c074 | s036 | Pied avocet | Recurvirostra avosetta | Waterbird | M | 31 | 2.94 | ICC | 0.2900 | N | NA | no | Conventional | Arrival\_breed | Wadden Sea Coast, Germany | Europe | 8.860000 | 54.63000 | Breeding |  | p. 383, text, results | text | Arrival of Pied Avocets Recurvirostra avosetta at the breeding site: Effects of winter quarters and consequences for reproductive success | 2002 | Hotker, H. | Ardea |  | ft015 |
| es150 | p047 | c075 | s036 | Pied avocet | Recurvirostra avosetta | Waterbird | M | 28 | 3.07 | ICC | 0.0900 | N | NA | no | Conventional | Arrival\_breed | Wadden Sea Coast, Germany | Europe | 8.860000 | 54.63000 | Breeding |  | p. 383, text, results | text | Arrival of Pied Avocets Recurvirostra avosetta at the breeding site: Effects of winter quarters and consequences for reproductive success | 2002 | Hotker, H. | Ardea |  | ft015 |
| es151 | p047 | c076 | s036 | Pied avocet | Recurvirostra avosetta | Waterbird | F | 27 | 2.96 | ICC | 0.0500 | N | NA | no | Conventional | Arrival\_breed | Wadden Sea Coast, Germany | Europe | 8.860000 | 54.63000 | Breeding |  | p. 383, text, results | text | Arrival of Pied Avocets Recurvirostra avosetta at the breeding site: Effects of winter quarters and consequences for reproductive success | 2002 | Hotker, H. | Ardea |  | ft015 |
| es152 | p047 | c077 | s036 | Pied avocet | Recurvirostra avosetta | Waterbird | F | 15 | 2.33 | ICC | -0.0800 | N | NA | no | Conventional | Arrival\_breed | Wadden Sea Coast, Germany | Europe | 8.860000 | 54.63000 | Breeding |  | p. 383, text, results | text | Arrival of Pied Avocets Recurvirostra avosetta at the breeding site: Effects of winter quarters and consequences for reproductive success | 2002 | Hotker, H. | Ardea |  | ft015 |
| es153 | p048 | c078 | s021 | Relict gull | Larus relictus | Seabird | B | 4 | 3.50 | ICC | 0.1300 | N | NA | no | Satellite | Depart\_breed | Hongjian Nur, China | Not | 109.930000 | 39.14000 | Breeding |  | Table 3, p. 7 | text | Seasonal dispersal and longitudinal migration in the Relict Gull Larus relictus across the Inner-Mongolian Plateau | 2017 | Liu, D. and Zhang, G. and Jiang, H. and Chen, L. and Meng, D. and Lu, J. | PeerJ | 10.7717/peerj.3380 | ft125 |
| es154 | p048 | c078 | s021 | Relict gull | Larus relictus | Seabird | B | 4 | 3.50 | ICC | 0.8000 | N | NA | no | Satellite | Nonbreed\_arrival | Hongjian Nur, China | Not | 109.930000 | 39.14000 | Breeding |  | Table 3, p. 7 | text | Seasonal dispersal and longitudinal migration in the Relict Gull Larus relictus across the Inner-Mongolian Plateau | 2017 | Liu, D. and Zhang, G. and Jiang, H. and Chen, L. and Meng, D. and Lu, J. | PeerJ | 10.7717/peerj.3380 | ft125 |
| es155 | p048 | c078 | s021 | Relict gull | Larus relictus | Seabird | B | 4 | 3.00 | ICC | -0.1800 | N | NA | no | Satellite | Nonbreed\_depart | Hongjian Nur, China | Not | 109.930000 | 39.14000 | Breeding |  | Table 3, p. 7 | text | Seasonal dispersal and longitudinal migration in the Relict Gull Larus relictus across the Inner-Mongolian Plateau | 2017 | Liu, D. and Zhang, G. and Jiang, H. and Chen, L. and Meng, D. and Lu, J. | PeerJ | 10.7717/peerj.3380 | ft125 |
| es156 | p048 | c078 | s021 | Relict gull | Larus relictus | Seabird | B | 4 | 3.00 | ICC | -0.0100 | N | NA | no | Satellite | Arrival\_breed | Hongjian Nur, China | Not | 109.930000 | 39.14000 | Breeding |  | Table 3, p. 7 | text | Seasonal dispersal and longitudinal migration in the Relict Gull Larus relictus across the Inner-Mongolian Plateau | 2017 | Liu, D. and Zhang, G. and Jiang, H. and Chen, L. and Meng, D. and Lu, J. | PeerJ | 10.7717/peerj.3380 | ft125 |
| es157 | p049 | c079 | s042 | Black-browed albatross | Thalassarche melanophrys | Seabird | B | 22 | 2.00 | r | -0.0700 | N | NA | NA | GLS | Depart\_breed | Bird Island, South Georgia | Not | -38.040000 | -54.00600 | Breeding |  | p. 2390, text, results | text | Summer distribution and migration of nonbreeding albatrosses: Individual consistencies and implications for conservation | 2005 | Phillips, R. A. and Silk, J. R. D. and Croxall, J. P. and Afanasyev, V. and Bennett, V. J. | Ecology | 10.1890/04-1885 | ft133 |
| es158 | p049 | c079 | s042 | Black-browed albatross | Thalassarche melanophrys | Seabird | B | 22 | 2.00 | r | 0.5000 | N | NA | NA | GLS | Arrival\_breed | Bird Island, South Georgia | Not | -38.040000 | -54.00600 | Breeding |  | p. 2390, text, results | text | Summer distribution and migration of nonbreeding albatrosses: Individual consistencies and implications for conservation | 2005 | Phillips, R. A. and Silk, J. R. D. and Croxall, J. P. and Afanasyev, V. and Bennett, V. J. | Ecology | 10.1890/04-1885 | ft133 |
| es159 | p049 | c079 | s042 | Black-browed albatross | Thalassarche melanophrys | Seabird | B | 21 | 2.00 | r | 0.2800 | N | NA | NA | GLS | Nonbreed\_arrival | Bird Island, South Georgia | Not | -38.040000 | -54.00600 | Breeding |  | p. 2390, text, results | text | Summer distribution and migration of nonbreeding albatrosses: Individual consistencies and implications for conservation | 2005 | Phillips, R. A. and Silk, J. R. D. and Croxall, J. P. and Afanasyev, V. and Bennett, V. J. | Ecology | 10.1890/04-1885 | ft133 |
| es160 | p049 | c079 | s042 | Black-browed albatross | Thalassarche melanophrys | Seabird | B | 21 | 2.00 | r | 0.5400 | N | NA | NA | GLS | Nonbreed\_depart | Bird Island, South Georgia | Not | -38.040000 | -54.00600 | Breeding |  | p. 2390, text, results | text | Summer distribution and migration of nonbreeding albatrosses: Individual consistencies and implications for conservation | 2005 | Phillips, R. A. and Silk, J. R. D. and Croxall, J. P. and Afanasyev, V. and Bennett, V. J. | Ecology | 10.1890/04-1885 | ft133 |
| es161 | p050 | c080 | s004 | Turnstone | Arenaria interpres | Waterbird | B | 39 | 2.00 | r | 0.7440 | N | NA | NA | Conventional | Nonbreed\_depart | Firth of Clyde | Europe | -4.770000 | 55.63000 | Non-breeding |  | p. 210, text, Figure 2 legend | text | Survival, winter population stability and site fidelity in the turnstone aren aria interpres | 1985 | Metcalfe, N. B. and Furness, R. W. | Bird Study | 10.1080/00063658509476881 | ft134 |
| es162 | p050 | c081 | s004 | Turnstone | Arenaria interpres | Waterbird | B | 32 | 2.00 | r | 0.4600 | N | NA | NA | Conventional | Nonbreed\_depart | Firth of Clyde | Europe | -4.770000 | 55.63000 | Non-breeding |  | p. 210, text, Figure 3 legend | text | Survival, winter population stability and site fidelity in the turnstone aren aria interpres | 1985 | Metcalfe, N. B. and Furness, R. W. | Bird Study | 10.1080/00063658509476881 | ft134 |
| es163 | p051 | c082 | s015 | Whooping crane | Grus americana | Waterbird | B | 23 | 4.40 | ICC | 0.4800 | Y | Age, social status | no | Satellite | Depart\_breed | Wood Buffalo National Park of Canada (breeding) and wintering sites along the Texas Gulf Coast | North America | -113.240000 | 59.30000 | both? |  | p. 6, text, results | text | Heterogeneity in migration strategies of Whooping Cranes | 2019 | Pearse, A. T. and Metzger, K. L. and Brandt, D. A. and Bidwell, M. T. and Harner, M. J. and Baasch, D. M. and Harrell, W. | Condor | 10.1093/condor/duz056 | ft063 |
| es164 | p051 | c082 | s015 | Whooping crane | Grus americana | Waterbird | B | 27 | 4.40 | ICC | 0.1700 | Y | Age, social status | no | Satellite | Nonbreed\_arrival | Wood Buffalo National Park of Canada (breeding) and wintering sites along the Texas Gulf Coast | North America | -113.240000 | 59.30000 | both? |  | p. 6, text, results | text | Heterogeneity in migration strategies of Whooping Cranes | 2019 | Pearse, A. T. and Metzger, K. L. and Brandt, D. A. and Bidwell, M. T. and Harner, M. J. and Baasch, D. M. and Harrell, W. | Condor | 10.1093/condor/duz056 | ft063 |
| es165 | p051 | c082 | s015 | Whooping crane | Grus americana | Waterbird | B | 30 | 4.10 | ICC | 0.4100 | Y | Age, social status | no | Satellite | Nonbreed\_depart | Wood Buffalo National Park of Canada (breeding) and wintering sites along the Texas Gulf Coast | North America | -113.240000 | 59.30000 | both? |  | p. 6, text, results | text | Heterogeneity in migration strategies of Whooping Cranes | 2019 | Pearse, A. T. and Metzger, K. L. and Brandt, D. A. and Bidwell, M. T. and Harner, M. J. and Baasch, D. M. and Harrell, W. | Condor | 10.1093/condor/duz056 | ft063 |
| es166 | p051 | c082 | s015 | Whooping crane | Grus americana | Waterbird | B | 27 | 4.50 | ICC | 0.2500 | Y | Age, social status | no | Satellite | Arrival\_breed | Wood Buffalo National Park of Canada (breeding) and wintering sites along the Texas Gulf Coast | North America | -113.240000 | 59.30000 | both? |  | p. 6, text, results | text | Heterogeneity in migration strategies of Whooping Cranes | 2019 | Pearse, A. T. and Metzger, K. L. and Brandt, D. A. and Bidwell, M. T. and Harner, M. J. and Baasch, D. M. and Harrell, W. | Condor | 10.1093/condor/duz056 | ft063 |
| es167 | p052 | c083 | s019 | Lesser black-backed gull | Larus fuscus | Seabird | B | 81 | 2.85 | ICC | 0.5100 | Y | Migration distance (fixed), colony and individual (random) | yes | Satellite | Depart\_breed | eight colonies in the Netherlands, Belgium and the UK | Europe | 3.180000 | 51.30000 | Breeding |  | p. 6, text, results | text | Long-distance migrants vary migratory behaviour as much as short-distance migrants: An individual-level comparison from a seabird species with diverse migration strategies | 2021 | Brown, J. M. and van Loon, E. E. and Bouten, W. and Camphuysen, K. C. J. and Lens, L. and Muller, W. and Thaxter, C. B. and Shamoun-Baranes, J. | Journal of Animal Ecology | 10.1111/1365-2656.13431 | ft095 |
| es168 | p052 | c083 | s019 | Lesser black-backed gull | Larus fuscus | Seabird | B | 80 | 2.86 | ICC | 0.7700 | Y | Migration distance (fixed), colony and individual (random) | yes | Satellite | Nonbreed\_arrival | eight colonies in the Netherlands, Belgium and the UK | Europe | 3.180000 | 51.30000 | Breeding |  | p. 6, text, results | text | Long-distance migrants vary migratory behaviour as much as short-distance migrants: An individual-level comparison from a seabird species with diverse migration strategies | 2021 | Brown, J. M. and van Loon, E. E. and Bouten, W. and Camphuysen, K. C. J. and Lens, L. and Muller, W. and Thaxter, C. B. and Shamoun-Baranes, J. | Journal of Animal Ecology | 10.1111/1365-2656.13431 | ft095 |
| es169 | p052 | c083 | s019 | Lesser black-backed gull | Larus fuscus | Seabird | B | 82 | 2.84 | ICC | 0.5800 | Y | Migration distance (fixed), colony and individual (random) | yes | Satellite | Nonbreed\_depart | eight colonies in the Netherlands, Belgium and the UK | Europe | 3.180000 | 51.30000 | Breeding |  | p. 6, text, results | text | Long-distance migrants vary migratory behaviour as much as short-distance migrants: An individual-level comparison from a seabird species with diverse migration strategies | 2021 | Brown, J. M. and van Loon, E. E. and Bouten, W. and Camphuysen, K. C. J. and Lens, L. and Muller, W. and Thaxter, C. B. and Shamoun-Baranes, J. | Journal of Animal Ecology | 10.1111/1365-2656.13431 | ft095 |
| es170 | p052 | c083 | s019 | Lesser black-backed gull | Larus fuscus | Seabird | B | 82 | 2.84 | ICC | 0.5700 | Y | Migration distance (fixed), colony and individual (random) | yes | Satellite | Arrival\_breed | eight colonies in the Netherlands, Belgium and the UK | Europe | 3.180000 | 51.30000 | Breeding |  | p. 6, text, results | text | Long-distance migrants vary migratory behaviour as much as short-distance migrants: An individual-level comparison from a seabird species with diverse migration strategies | 2021 | Brown, J. M. and van Loon, E. E. and Bouten, W. and Camphuysen, K. C. J. and Lens, L. and Muller, W. and Thaxter, C. B. and Shamoun-Baranes, J. | Journal of Animal Ecology | 10.1111/1365-2656.13431 | ft095 |
| es171 | p053 | c084 | s040 | Common eider | Somateria mollissima | Waterbird | F | 24 | 2.75 | ICC | 0.5240 | N | NA | yes | GLS | Nonbreed\_depart | Kongsfjorden in Svalbard (79<U+00B0>N, 12<U+00B0>E) | Europe | 11.880000 | 79.03000 | Breeding |  | p. 9, text, results | text | Repeatability and Flexibility in the Migration Strategies of an Arctic Seabird | 2016 | Bjorn, N. | MSc thesis |  | ft166 |
| es172 | p053 | c085 | s040 | Common eider | Somateria mollissima | Waterbird | F | 6 | 2.50 | ICC | 0.5400 | N | NA | yes | GLS | Nonbreed\_depart | Kongsfjorden in Svalbard (79<U+00B0>N, 12<U+00B0>E) | Europe | 11.880000 | 79.03000 | Breeding |  | p. 9, text, results | text | Repeatability and Flexibility in the Migration Strategies of an Arctic Seabird | 2016 | Bjorn, N. | MSc thesis |  | ft166 |
| es173 | p053 | c084 | s040 | Common eider | Somateria mollissima | Waterbird | F | 24 | 2.75 | ICC | 0.5390 | Y | Breeding success | yes | GLS | Nonbreed\_arrival | Kongsfjorden in Svalbard (79<U+00B0>N, 12<U+00B0>E) | Europe | 11.880000 | 79.03000 | Breeding |  | p. 9, text, results | text | Repeatability and Flexibility in the Migration Strategies of an Arctic Seabird | 2016 | Bjorn, N. | MSc thesis |  | ft166 |
| es174 | p053 | c085 | s040 | Common eider | Somateria mollissima | Waterbird | F | 6 | 2.50 | ICC | 0.7440 | Y | Breeding success | yes | GLS | Nonbreed\_arrival | Kongsfjorden in Svalbard (79<U+00B0>N, 12<U+00B0>E) | Europe | 11.880000 | 79.03000 | Breeding |  | p. 9, text, results | text | Repeatability and Flexibility in the Migration Strategies of an Arctic Seabird | 2016 | Bjorn, N. | MSc thesis |  | ft166 |
| es175 | p053 | c086 | s040 | Common eider | Somateria mollissima | Waterbird | F | 30 | 2.70 | ICC | 0.5350 | N | NA | yes | GLS | Nonbreed\_depart | Kongsfjorden in Svalbard (79<U+00B0>N, 12<U+00B0>E) | Europe | 11.880000 | 79.03000 | Breeding |  | p. 9, text, results | text | Repeatability and Flexibility in the Migration Strategies of an Arctic Seabird | 2016 | Bjorn, N. | MSc thesis |  | ft166 |
| es176 | p053 | c086 | s040 | Common eider | Somateria mollissima | Waterbird | F | 30 | 2.70 | ICC | 0.6440 | Y | Breeding success | yes | GLS | Nonbreed\_arrival | Kongsfjorden in Svalbard (79<U+00B0>N, 12<U+00B0>E) | Europe | 11.880000 | 79.03000 | Breeding |  | p. 9, text, results | text | Repeatability and Flexibility in the Migration Strategies of an Arctic Seabird | 2016 | Bjorn, N. | MSc thesis |  | ft166 |
| es177 | p054 | c087 | s047 | White-eyed vireo | Vireo griseus | Landbird | M | 32 | 2.00 | r | 0.6160 | N | NA | NA | Conventional | Arrival\_breed | southwestern Virginia | North America | -81.530000 | 36.84000 | Breeding |  | p. 49, text | text | Banding Returns, Arrival Pattern, and Site-Fidelity of White-Eyed Vireos | 1999 | Hopp, S. L. and Kirby, A. and Boone, C. A. | The Wilson Bulletin |  | ft017 |

A. **es\_ID**: Unique ID for each row of data (i.e. each effect size).

B. **paper\_ID**: Unique ID for each paper.

C. **cohort\_ID**: Unique ID for each cohort of birds.

D. **species\_ID**: Unique ID for each species.

E. **species\_common**: Common name of species (taken from paper).

F. **species\_latin**: Latin name of species (taken from paper).

G. **taxa**: Species split into three ecological groups: ‘waterbird’, ‘seabird’ or ‘landbird’ based on Geen et al. (2019)

H. **sex**: Male, female or both/unknown.

I. **n**: Number of individuals.

J. **k**: Number of observations per individual.

K. **est**: Method of calculating repeatability: correlation coefficient (r) or intraclass correlation coefficient (ICC).

L. **R**: Repeatability value.

M. **fixed\_yn**: Any fixed effects (or additional random effects) included in repeatability calculation: yes or no.

N. **fixed\_var**: If yes, what additional variables are included.

O. **unstandardized\_variance**: Whether unstandardized variance components are reported.

P. **method**: Tracking method: conventional (ringing, colour-ringing), geolocator (geolocation), or GPS (GPS, satellite, PTT).

Q. **annual\_event**: Period of annual cycle which repeatability is measured (arrival to, or departure from, breeding or non-breeding grounds).

R. **location**: Location of tagging (as written by paper).

S. **continent**: Continent of tagging location: North America, Europe, or Other.

T. **long**: Longitude of tagging location.

U. **lat**: Latitude of tagging location.

V. **tag\_period**: Whether individuals were tagged on the breeding or non-breeding grounds.

W. **notes**: general comments

X. **data\_location**: Where in the paper the data is located.

Y. **data\_presentation**: Text, figure, or table.

Z. **title**: Title of the paper.

AA. **pub\_year**: Publication year of paper.

AB: **authors**: Authors of the paper.

AC: **journal**: Journal the paper was published in.

AD: **DOI**: DOI of the paper.

AE: **fulltext\_ID**: An ID that is used to link the paper for data extraction to the record of screened full texts.

### Sample sizes

#### Table S2

Sample sizes for our data set in terms of effect sizes, cohorts, studies, species, and the number of effect sizes in the different levels of categorical variables (factors), split by ecological group (seabird, waterbird and landbird), and overall.

```
# making a table of sample sizes for different variables
df %>%
    group_by(taxa) %>%
    summarise(`Effect sizes (analyses)` = n(), Cohort = n_distinct(cohort_ID), Studies = n_distinct(authors),
        Species = n_distinct(species_ID), `Arrival at breeding grounds` = sum(annual_event ==
            "Arrival_breed", na.rm = T), `Departure from breeding grounds` = sum(annual_event ==
            "Depart_breed", na.rm = T), `Arrival at non-breeding grounds` = sum(annual_event ==
            "Nonbreed_arrival", na.rm = T), `Departure from non-breeding grounds` = sum(annual_event ==
            "Nonbreed_depart", na.rm = T), `Conventional method` = sum(method ==
            "Conventional", na.rm = T), `GLS method` = sum(method == "GLS", na.rm = T),
        `Satellite method` = sum(method == "Satellite", na.rm = T), Female = sum(sex ==
            "F", na.rm = T), Male = sum(sex == "M", na.rm = T), `Mixed sex` = sum(sex ==
            "B", na.rm = T)) -> n_table1

n_table2 <- t(n_table1[, -1])
colnames(n_table2) <- c("Landbird", "Seabird", "Waterbird")
n_table2 %>%
    as_tibble(rownames = "Number of") %>%
    mutate(All = Landbird + Seabird + Waterbird) %>%
    kable() %>%
    kable_styling("striped", position = "left") %>%
    pack_rows("All data", 1, 4) %>%
    pack_rows("Annual event", 5, 8) %>%
    pack_rows("Tracking method", 9, 11) %>%
    pack_rows("Sex", 12, 14)
```

| Number of | Landbird | Seabird | Waterbird | All |
| --- | --- | --- | --- | --- |
| **All data** | | | | |
| Effect sizes (analyses) | 54 | 68 | 55 | 177 |
| Cohort | 29 | 29 | 29 | 87 |
| Studies | 20 | 14 | 20 | 54 |
| Species | 18 | 15 | 14 | 47 |
| **Annual event** | | | | |
| Arrival at breeding grounds | 27 | 29 | 20 | 76 |
| Departure from breeding grounds | 10 | 27 | 7 | 44 |
| Arrival at non-breeding grounds | 8 | 6 | 11 | 25 |
| Departure from non-breeding grounds | 9 | 6 | 17 | 32 |
| **Tracking method** | | | | |
| Conventional method | 19 | 2 | 21 | 42 |
| GLS method | 19 | 54 | 18 | 91 |
| Satellite method | 16 | 12 | 16 | 44 |
| **Sex** | | | | |
| Female | 4 | 8 | 10 | 22 |
| Male | 20 | 8 | 3 | 31 |
| Mixed sex | 30 | 52 | 42 | 124 |

### Marking locations of studies

#### Figure 1

```
worldmap <- map_data("world")  # get map data

# Plot of world with all tracking study locations
world <- ggplot() + geom_polygon(data = worldmap, aes(x = long, y = lat, group = group),
    fill = "grey80", colour = NA, size = 0.1) + coord_fixed(1.3) + theme_minimal() +
    scale_colour_manual(values = c(Seabird = "#009E73", Landbird = "#D55E00", Waterbird = "#0072B2")) +
    geom_point(data = df, aes(x = long, y = lat, colour = taxa, shape = method),
        size = 1.5) + geom_rect(aes(xmin = -12, xmax = 23, ymin = 32, ymax = 64),
    fill = NA, colour = "black", size = 0.3) + theme(panel.grid = element_line(colour = "white"),
    axis.title = element_blank(), axis.text = element_blank(), axis.ticks = element_blank(),
    legend.position = "none")

## Zoomed in plot on Europe
zoom <- ggplot() + geom_polygon(data = worldmap, aes(x = long, y = lat, group = group),
    fill = "grey80", colour = NA, size = 0.2) + coord_sf(xlim = c(-11, 23), ylim = c(32,
    64)) + theme_minimal() + theme(legend.text = element_text(size = 6), legend.title = element_text(size = 7,
    face = "bold"), legend.key.size = unit(1, "mm")) + theme(panel.grid = element_line(colour = "white"),
    axis.title = element_blank(), axis.text = element_blank(), panel.border = element_rect(colour = "black",
        fill = NA, size = 0.5), axis.ticks = element_blank()) + scale_colour_manual(name = "Ecological group",
    values = c(Seabird = "#009E73", Landbird = "#D55E00", Waterbird = "#0072B2")) +
    scale_shape_discrete(name = "Method") + geom_point(data = df, aes(x = long, y = lat,
    shape = method, colour = taxa), size = 1.5)

figure1 <- plot_grid(world, zoom, nrow = 1, rel_widths = c(1.3, 1))
# ggsave('figs/Map_of_tagging_locations.pdf', dpi=600, width=180, height=60,
# units='mm')

figure1
```

**Figure 1.** The marking locations of birds for all studies with repeatability estimates collated from the literature and included in analyses, coloured by ecological group (waterbird, seabird, or landbird), and shaped by tracking method (conventional, satellite, or GLS).

## Meta-analysis

### Calculating effect sizes

We created our effect size (correlation coefficient *r* and intra-class correlation coefficient *ICC* and their Fisher’s z transformation *Zr*) from repeatability values and associated sample sizes. We followed the equations outlined in Holtmann et al. (2017). We converted the negative repeatability estimates (n=13) in our dataset to zero, as they often only reflect noise around a statistical zero (Nakagawa & Schielzeth, 2010), but you can find the meta-analytic and meta-regression models with the negative repeatability estimates included in Tables S12-19.

```
# Creating new columns for effect sizes (Zr) and sampling variance (VZr)
df$VZr <- df$Zr <- NA

# Calculate effect sizes using custom function
for (i in as.numeric(rownames(df))) {
    r <- df$R[i]
    K <- df$k[i]
    Est <- df$est[i]

    Zr <- Zr_transformation(r, K, Est)

    df$Zr[i] <- Zr

}
# Calculate sampling variances using custom function
for (i in as.numeric(rownames(df))) {
    K <- df$k[i]
    N <- df$n[i]
    Est <- df$est[i]

    VZr <- Calc_SV(K, N, Est)

    df$VZr[i] <- VZr

}

# Add a new column with all negative repeatability values set to zero
df$Zr2 <- ifelse(df$Zr < 0, 0, df$Zr + 0)
```

### Phylogenetic tree

Since multiple bird species (n = 47) are included in this dataset, we needed to consider phylogenetic non-independence in our models (Chamberlain et al., 2012, Cinar et al. 2022). To generate the phylogeny, we used a phylogenetic tree from Jetz et al. (2012) (provided by Benedikt Holtmann), which was prepared on the basis of Hackett backbone (Hackett tree; Hackett et al., 2008). We first searched the tree for the species in our dataset to confirm and correct the species names.

- *Limosa lapponica baueri* was assumed to be synonym to *Limosa lapponica*
- *Limosa limosa limosa* and *Limosa limosa islandica* were assumed to be synonym to *Limosa limosa*
- *Cygnus columbianus bewickii* was assumed to be synonym to *Cygnus columbianus*
- *Catharacta antarctica lonnbergi* was assumed to be synonym to *Catharacta antarctica*
- *Pterodroma deserta* was assumed to be synonym to *Pterodroma mollis*
- *Chen canagicus* was assumed to be synonym to *Chen canagica*
- *Anser caerulescens atlanticus* was assumed to be synonym to *Chen caerulescens*

We then trimmed the tree to include only the species names in our data set, and computed branch lengths using Grafen’s method (Grafen, 1989) in the compute.brlen function in the R package ape (Paradis & Schliep, 2019).

#### Figure S3

```
species_list <- unique(df$species_latin)  # use unique() as some names are repeated
species_list <- gsub(" ", "_", species_list)  # replace spaces with underscore so they match tree

# Load bird supertree based on Hackett's backbone
bird_tree_hackett <- read.tree(here("data", "Hackett.tre"))  # tree provided by Benedikt Holtmann

# Prune phylogentic tree for meta-analysis

# Check the tree bird_tree_hackett # 9993 tips = species str(bird_tree_hackett)
# # has edge (branch) lengths
bird_tree_hackett <- collapse.singles(bird_tree_hackett)

# Get only bird species from the supertree that are also included in collected
# data
bird_tree_species <- as.character(bird_tree_hackett$tip.label)

# Check the overlap of species names between collected data file and the
# supertree All species should be present. If not, species names may not match
# with names in the supertree intersect(bird_tree_species, species_list)
# character(39) - should be 47, need to check which species do/do not match

# gives list of species names which are not matched species_list[!species_list
# %in% bird_tree_species]
#'Limosa_lapponica_baueri'
#'Cygnus_columbianus_bewickii'
#'Limosa_limosa_limosa'
#'Limosa_limosa_islandica'
#'Catharacta_antarctica_lonnbergi'
#'Pterodroma_deserta'
#'Chen_canagicus'
#'Anser_caerulescens_atlanticus'

# making a new list so it is clear that this list of species is an updated
# version compared to the original one
species_list_hackett <- species_list

# Changing subspecies to species, or old genus names from papers to new Only
# Pterodroma deserta: 3 species in the Pterodroma feae/madeira/desertae complex
# were once believed to be subspecies of a single species: Pterodroma mollis
species_list_hackett <- replace(species_list_hackett, species_list_hackett == "Limosa_lapponica_baueri",
    "Limosa_lapponica")
species_list_hackett <- replace(species_list_hackett, species_list_hackett == "Limosa_limosa_limosa" |
    species_list_hackett == "Limosa_limosa_islandica", "Limosa_limosa")
species_list_hackett <- replace(species_list_hackett, species_list_hackett == "Cygnus_columbianus_bewickii",
    "Cygnus_columbianus")
species_list_hackett <- replace(species_list_hackett, species_list_hackett == "Catharacta_antarctica_lonnbergi",
    "Catharacta_antarctica")
species_list_hackett <- replace(species_list_hackett, species_list_hackett == "Pterodroma_deserta",
    "Pterodroma_mollis")
species_list_hackett <- replace(species_list_hackett, species_list_hackett == "Chen_canagicus",
    "Chen_canagica")
species_list_hackett <- replace(species_list_hackett, species_list_hackett == "Anser_caerulescens_atlanticus",
    "Chen_caerulescens")

# Now check and see if all species are present in supertree
# intersect(bird_tree_species, species_list_hackett) # = 47 (not 48, because L.
# l. limosa and L. i. islandica both changed to L. limosa)

# Prune supertree to the list of taxa included in the data
pruned_birds_stree <- drop.tip(bird_tree_hackett, bird_tree_hackett$tip.label[-match(species_list_hackett,
    bird_tree_hackett$tip.label)])

# Check if tree is binary and ultrametric is.binary(pruned_birds_stree) # TRUE
# is.ultrametric(pruned_birds_stree) # TRUE

# Save pruned tree to be use in the meta-analysis
write.tree(pruned_birds_stree, file = here("data", "birds_meta-analysis_tree.tre"),
    append = FALSE, digits = 10, tree.names = FALSE)

# Make phylogenetic correlation matrix

# Using metafor - correlation matrix for species in tree
varcor <- vcv(pruned_birds_stree, corr = TRUE)

# Create a column called phylogeny, which matches the tree Copied column to
# edit species names to match tree (as some sp. names are from old papers, or
# are subspecies)
df$species_latin_hackett <- as.character(df$species_latin)
df$species_latin_hackett <- replace(df$species_latin_hackett, df$species_latin_hackett ==
    "Limosa lapponica baueri", "Limosa lapponica")
df$species_latin_hackett <- replace(df$species_latin_hackett, df$species_latin_hackett ==
    "Limosa limosa limosa" | df$species_latin_hackett == "Limosa limosa islandica",
    "Limosa limosa")
df$species_latin_hackett <- replace(df$species_latin_hackett, df$species_latin_hackett ==
    "Cygnus columbianus bewickii", "Cygnus columbianus")
df$species_latin_hackett <- replace(df$species_latin_hackett, df$species_latin_hackett ==
    "Catharacta antarctica lonnbergi", "Catharacta antarctica")
df$species_latin_hackett <- replace(df$species_latin_hackett, df$species_latin_hackett ==
    "Pterodroma deserta", "Pterodroma mollis")
df$species_latin_hackett <- replace(df$species_latin_hackett, df$species_latin_hackett ==
    "Chen canagicus", "Chen canagica")
df$species_latin_hackett <- replace(df$species_latin_hackett, df$species_latin_hackett ==
    "Anser caerulescens atlanticus", "Chen caerulescens")

df$phylogeny <- gsub(" ", "_", df$species_latin_hackett)

# Plot tree to see how it looks like
plot(pruned_birds_stree, label.offset = 2, cex = 0.8, main = "'Hackett tree'", cex.main = 1,
    line = 0.5)  # with branch lengths
```

**Figure S3.** Phylogenetic tree (with Hackett backbone) used for phylogenetic meta-analysis and meta-regression on repeatability in avian migratory timings.

### Meta-analytic model

#### Running multilevel meta-analytic model with and without phylogeny

We used the `rma.mv` function from the package `metafor` (Viechtbauer, 2010) to run all meta-analytic models and meta-regressions. This function allows us to incorporate variance-covariance matrices in the V term. We assumed a correlation of 0.5 (Noble et al., 2017) that will be fitted as part of the random effect structure of our models but also test different levels of correlation (see Sensitivity analysis).

As we have a multi-species dataset, we use the model that accounts for both the non-phylogenetic and phylogenetic species-level variance in addition to the full multilevel structure of the data as any attempts to simplify this model, such as using only the phylogenetic variance component, may lead to erroneous inferences from the data (Cinar et al. 2022).

```
# Create a variance-covariance matrix at the cohort level
VCV <- impute_covariance_matrix(vi = df$VZr, cluster = df$cohort_ID, r = 0.5)
# Why 0.5 - see https://onlinelibrary.wiley.com/doi/full/10.1111/mec.14031

ma_model1 <- rma.mv(yi = Zr2, V = VCV,
                   random = list(~1 | es_ID, 
                                 ~1 | paper_ID, 
                                 ~1 | cohort_ID, 
                                 ~1 | species_ID), 
                   data = df)

ma_model2 <- rma.mv(yi = Zr2, V = VCV, 
                      random = list(~1 | es_ID, 
                                    ~1 | paper_ID, 
                                    ~1 | cohort_ID, 
                                    ~1 | species_ID, # non-phylo effect
                                    ~1 | phylogeny), # phylo effect
                      R = list(phylogeny = varcor), # phylogenetic relatedness
                      data = df)
```

#### Table S3

Overall effects (meta-analytic means) and 95% confidence intervals (CIs) both in *Zr* and back-transformed to *ICC*, and heterogeneity, *I*2, for the multilevel intercept-only meta-analysis models including and excluding phylogeny.

```
## # estimating I2 as measure of heterogeneity
i2_ma1 <- round(i2_ml(ma_model1) * 100, 1)
i2_ma2 <- round(i2_ml(ma_model2) * 100, 1)

# Back-transform to ICC
ma1 <- mod_results(ma_model1, mod = "Int")
ma1_mod_table <- ma1$mod_table

ma2 <- mod_results(ma_model2, mod = "Int")
ma2_mod_table <- ma2$mod_table

# need to calculate k for whole data set to use in formula

k_all <- mean(df$k)

for (i in names(ma1_mod_table)[2:6]) {

    ma1_mod_table[i] <- Zr_to_ICC(ma1_mod_table[i], k_all)

}

for (i in names(ma2_mod_table)[2:6]) {

    ma2_mod_table[i] <- Zr_to_ICC(ma2_mod_table[i], k_all)

}

# creating a table
tibble(Model = c("Meta-analysis (Zr)", "Meta-analysis (ICC)", "Meta-analysis phylo (Zr)",
    "Meta-analysis phylo (ICC)"), `Overall mean` = c(ma_model1$b, ma1_mod_table$estimate,
    ma_model2$b, ma2_mod_table$estimate), `Lower CI [0.025]` = c(ma_model1$ci.lb,
    ma1_mod_table$lowerCL, ma_model2$ci.lb, ma2_mod_table$lowerCL), `Upper CI [0.975]` = c(ma_model1$ci.ub,
    ma1_mod_table$upperCL, ma_model2$ci.ub, ma2_mod_table$upperCL), `I^2^~total~` = c(i2_ma1[1],
    NA, i2_ma2[1], NA), `I^2^~es~` = c(i2_ma1[2], NA, i2_ma2[2], NA), `I^2^~paper~` = c(i2_ma1[3],
    NA, i2_ma2[3], NA), `I^2^~cohort~` = c(i2_ma1[4], NA, i2_ma2[4], NA), `I^2^~species~` = c(i2_ma1[5],
    NA, i2_ma2[5], NA), `I^2^~phylo~` = c(NA, NA, i2_ma2[6], NA), ) %>%
    kable("html", digits = 3) %>%
    kable_styling("striped", position = "left")
```

| Model | Overall mean | Lower CI [0.025] | Upper CI [0.975] | I2total | I2es | I2paper | I2cohort | I2species | I2phylo |
| --- | --- | --- | --- | --- | --- | --- | --- | --- | --- |
| Meta-analysis (Zr) | 0.541 | 0.445 | 0.637 | 84.0 | 50.6 | 0 | 0 | 33.4 | NA |
| Meta-analysis (ICC) | 0.421 | 0.348 | 0.490 | NA | NA | NA | NA | NA | NA |
| Meta-analysis phylo (Zr) | 0.532 | 0.400 | 0.664 | 84.2 | 49.7 | 0 | 0 | 27.3 | 7.2 |
| Meta-analysis phylo (ICC) | 0.414 | 0.313 | 0.508 | NA | NA | NA | NA | NA | NA |

#### Figure 2a

```
ma2_data <- ma2$data

# back-transform model results to ICC
# need to calculate k for whole data set to use in formula

ma2_data$yi_ICC <- Zr_to_ICC(ma2_data$yi, k_all)

ma2_data$moderator <- factor(ma2_data$moderator, levels = ma2_mod_table$name, labels = ma2_mod_table$name)
ma2_data$scale <- (1/sqrt(ma2_data[,"vi"]))
ma2_mod_table$K <- as.vector(by(ma2_data, ma2_data[,"moderator"], function(x) length(x[,"yi"])))
ma2_group_no <- nrow(ma2_mod_table)

# colour blind friendly colours with grey
cbpl <- c("#E69F00","#009E73", "#F0E442", "#0072B2", "#D55E00", "#CC79A7", "#56B4E9", "#999999")

# creating an orchard plot
fig_ma2 <- ggplot(data = ma2_mod_table, aes(x = estimate, y = "Overall mean")) + 
     scale_x_continuous(limits = c(-0.37, 1), breaks = seq(-0.4, 1, by = 0.2), labels = c("-0.4", "-0.2", "0.0", "0.2", "0.4", "0.6", "0.8", "1.0")) +
    ggbeeswarm::geom_quasirandom(data = ma2_data, aes(x = yi_ICC, y = "Overall mean", size = scale), fill="black", groupOnX=FALSE, alpha = 0.2) +
    geom_errorbarh(aes(xmin = lowerPR, xmax = upperPR), height = 0, show.legend = F, size = 0.5, alpha = 0.6) +
    geom_errorbarh(aes(xmin = lowerCL, xmax = upperCL), height = 0, show.legend = F, size = 1.2) + 
    geom_vline(xintercept = 0, linetype = 2, colour = "black", alpha = 0.5) +
    geom_point(fill="black", size = 3, shape = 21) + 
    annotate('text', x = 0.93, y = (seq(1, ma2_group_no, 1)+0.35), label= paste("italic(k)==", ma2_mod_table$K), parse=TRUE, hjust = "left", size=3.5) +
    ggplot2::theme_bw() +
    ggplot2::guides(fill="none", colour="none") +
    ggplot2::theme(legend.position = c(1,0), legend.justification = c(1,0), legend.title = element_text(size=8), 
                   legend.text = element_text(size=8), legend.key = element_rect(colour = NA, fill = NA), 
                   legend.background = element_blank(), legend.direction = "horizontal",
                   axis.text.x = element_text(size=9),
                   axis.text.y = element_text(size=9, colour="black", hjust=0.5, angle=90), 
                   axis.title.y = element_blank(), legend.key.size = unit(0.5,'cm')) +
    ggplot2::labs(size=paste("Precision (1/SE)"), x=paste("Effect size (ICC)"))

# For Figure 2

a <- ggplot(data = ma2_mod_table, aes(x = estimate, y = "Overall mean")) + 
     scale_x_continuous(limits = c(-0.37, 1), breaks = seq(-0.4, 1, by = 0.2), labels = c("-0.4", "-0.2", "0.0", "0.2", "0.4", "0.6", "0.8", "1.0")) +
    ggbeeswarm::geom_quasirandom(data = ma2_data, aes(x = yi_ICC, y = "Overall mean", size = scale), fill="black", groupOnX=FALSE, alpha = 0.2) +
    geom_errorbarh(aes(xmin = lowerPR, xmax = upperPR), height = 0, show.legend = F, size = 0.5, alpha = 0.6) + # CI
    geom_errorbarh(aes(xmin = lowerCL, xmax = upperCL), height = 0, show.legend = F, size = 1.2) + 
    geom_vline(xintercept = 0, linetype = 2, colour = "black", alpha = 0.5) +
    geom_point(fill="black", size = 3, shape = 21) + 
    annotate('text', x = 0.93, y = (seq(1, ma2_group_no, 1)+0.35), label= paste("italic(k)==", ma2_mod_table$K), parse=TRUE, hjust = "left", size=3.5) +
    ggplot2::theme_bw() +
    ggplot2::guides(fill="none", colour="none") +
    ggplot2::theme(legend.position = c(1,0), legend.justification = c(1,0), legend.title = element_text(size=8), 
                   legend.text = element_text(size=8), legend.key = element_rect(colour = NA, fill = NA), 
                   legend.background = element_blank(), legend.direction = "horizontal", 
                   axis.text.y = element_text(size=9, colour="black", hjust=0.5, angle=90), 
                   axis.title = element_blank(), legend.key.size = unit(0.5,'cm')) +
    ggplot2::labs(size=paste("Precision (1/SE)"))

fig_ma2
```

**Figure 2a.** Repeatability of avian migration timing for all estimates together. The plot shows the phylogenetic meta-analytic mean with 95% confidence intervals (thick lines, indicating uncertainty around the overall estimate) and 95% prediction intervals (thin lines, indicating the possible range for 95% of new (or simply not included) effect sizes), observed effect sizes (back-transformed to ICC) scaled by precision (circles) and k = number of effect sizes. The graph was constructed using code adapted from the `orchard_plot` function in the orchaRd package (Nakagawa, Lagisz, O’Dea, et al., 2021).

## Meta-regression

We ran a univariate meta-regression model for each of the following moderators: 1) `annual_event`, 2) `method`, 3) `taxa`, 4) `sex` and 5) number of samples per individual (`k`).

### Univariate (uni-predictor) analyses

We first conducted a series of meta-regression models with each of the moderators introduced above.

#### Period of the annual cycle

```
# meta-regression: mutiple intercepts
meta_regression1 <- rma.mv(yi = Zr2, V = VCV, 
                           mods = ~ annual_event,
                           random = list(~1 | es_ID, 
                                         ~1 | paper_ID, 
                                         ~1 | cohort_ID, 
                                         ~1 | species_ID, 
                                         ~1 | phylogeny),
                           R = list(phylogeny = varcor), # added in phylogney
                           data = df)

meta_regression1b <- rma.mv(yi = Zr2, V = VCV, 
                            mods = ~ relevel(annual_event, ref = "Depart_breed"),
                            random = list(~1 | es_ID, 
                                          ~1 | paper_ID, 
                                          ~1 | cohort_ID, 
                                          ~1 | species_ID, 
                                          ~1 | phylogeny),
                            R = list(phylogeny = varcor), # added in phylogney
                            data = df)


meta_regression1c <- rma.mv(yi = Zr2, V = VCV, 
                            mods = ~ relevel(annual_event, ref = "Nonbreed_depart"),
                            random = list(~1 | es_ID, 
                                          ~1 | paper_ID, 
                                          ~1 | cohort_ID, 
                                          ~1 | species_ID, 
                                          ~1 | phylogeny),
                            R = list(phylogeny = varcor), # added in phylogney
                            data = df)

# meta-regression: contrast (for orchard plot)
meta_regression1d <- rma.mv(yi = Zr2, V = VCV, 
                            mods = ~ annual_event -1,
                            random = list(~1 | es_ID, 
                                          ~1 | paper_ID, 
                                          ~1 | cohort_ID, 
                                          ~1 | species_ID, 
                                          ~1 | phylogeny),
                            R = list(phylogeny = varcor), # added in phylogney
                            data = df)
```

#### Table S4

Regression coefficients (Estimate) and 95% confidence intervals (CIs) both in Zr and back-transformed to ICC from the meta-regression with `annual_event`. Note that `mu` means the group mean while `beta` represents the contrast between two groups in the Unit column. *R2marginal* = 10.5%.

```
# getting marginal R2
r2_meta_regression1 <- r2_ml(meta_regression1)

# getting estimates including back-transformation to ICC

mr1 <- mod_results(meta_regression1d, mod = "annual_event")
mr1_mod_table <- mr1$mod_table

mr1_data <- mr1$data

# calculate k for each method separately

# df %>% group_by(annual_event) %>% summarise(mean(k))

mr1_data <- mr1_data %>%
    mutate(k = case_when(moderator == "Arrival_breed" ~ 2.55, moderator == "Depart_breed" ~
        2.45, moderator == "Nonbreed_arrival" ~ 3.2, moderator == "Nonbreed_depart" ~
        2.94))

mr1_data$yi_ICC <- Zr_to_ICC(mr1_data$yi, mr1_data$k)

mr1_mod_table$k <- c(2.55, 2.45, 3.2, 2.94)

for (i in names(mr1_mod_table)[2:6]) {

    mr1_mod_table[i] <- Zr_to_ICC(mr1_mod_table[i], mr1_mod_table$k)

}

# creating a table
tibble(`Fixed effect` = c(rep(as.character(mr1_mod_table$name), 2), cont_gen(mr1_mod_table$name)),
    Unit = c(rep(c("Zr (mu)", "ICC (mu)"), each = 4), rep("Zr (beta)", 6)), Estimate = c(meta_regression1d$b,
        mr1_mod_table$estimate, meta_regression1$b[-1], meta_regression1b$b[-(1:2)],
        meta_regression1c$b[-(1:3)]), `Lower CI [0.025]` = c(meta_regression1d$ci.lb,
        mr1_mod_table$lowerCL, meta_regression1$ci.lb[-1], meta_regression1b$ci.lb[-(1:2)],
        meta_regression1c$ci.lb[-(1:3)]), `Upper CI  [0.975]` = c(meta_regression1d$ci.ub,
        mr1_mod_table$upperCL, meta_regression1$ci.ub[-1], meta_regression1b$ci.ub[-(1:2)],
        meta_regression1c$ci.ub[-(1:3)])) -> t_annual_event
# `R2` = c(r2_meta_regression1[1], rep(NA,13))) ->

t_annual_event %>%
    kable("html", digits = 3) %>%
    kable_styling("striped", position = "left")
```

| Fixed effect | Unit | Estimate | Lower CI [0.025] | Upper CI [0.975] |
| --- | --- | --- | --- | --- |
| Arrival\_breed | Zr (mu) | 0.472 | 0.308 | 0.637 |
| Depart\_breed | Zr (mu) | 0.391 | 0.206 | 0.576 |
| Nonbreed\_arrival | Zr (mu) | 0.593 | 0.396 | 0.791 |
| Nonbreed\_depart | Zr (mu) | 0.719 | 0.531 | 0.908 |
| Arrival\_breed | ICC (mu) | 0.381 | 0.250 | 0.503 |
| Depart\_breed | ICC (mu) | 0.326 | 0.172 | 0.469 |
| Nonbreed\_arrival | ICC (mu) | 0.416 | 0.274 | 0.547 |
| Nonbreed\_depart | ICC (mu) | 0.522 | 0.391 | 0.636 |
| Arrival\_breed-Depart\_breed | Zr (beta) | -0.081 | -0.225 | 0.062 |
| Arrival\_breed-Nonbreed\_arrival | Zr (beta) | 0.121 | -0.048 | 0.290 |
| Arrival\_breed-Nonbreed\_depart | Zr (beta) | 0.247 | 0.088 | 0.405 |
| Depart\_breed-Nonbreed\_arrival | Zr (beta) | 0.202 | 0.023 | 0.382 |
| Depart\_breed-Nonbreed\_depart | Zr (beta) | 0.328 | 0.157 | 0.500 |
| Nonbreed\_arrival-Nonbreed\_depart | Zr (beta) | -0.126 | -0.301 | 0.049 |

#### Figure 2b

```
# Orchard plot for annual event with ICC

mr1_data$moderator <- factor(mr1_data$moderator, levels = mr1_mod_table$name, labels = mr1_mod_table$name)
mr1_data$scale <- (1/sqrt(mr1_data[, "vi"]))
mr1_mod_table$K <- as.vector(by(mr1_data, mr1_data[, "moderator"], function(x) length(x[,
    "yi"])))
mr1_group_no <- nrow(mr1_mod_table)

image_ABegg <- readPNG(here("images/AB_eggs_icon.png"))
image_ANB <- readPNG(here("images/ANB_icon.png"))
image_DBchick <- readPNG(here("images/DB_chicks_icon.png"))
image_DNB <- readPNG(here("images/DNB_icon.png"))

# creating an orchard plot

# fig_annual_event <- ggplot(data = mr1_mod_table, aes(x = estimate, y = name))
# + scale_x_continuous(limits = c(-0.4, 1), breaks = seq(-0.4, 1, by = 0.2),
# labels = c('-0.4', '-0.2', '0.0', '0.2', # '0.4', '0.6', '0.8', '1.0')) +
# ggbeeswarm::geom_quasirandom(data = mr1_data, aes(x = yi_ICC, y = moderator,
# size = scale, colour=moderator), groupOnX=FALSE, # alpha = 0.4) +
# geom_errorbarh(aes(xmin = lowerPR, xmax = upperPR), height = 0, show.legend =
# F, size = 0.5, alpha = 0.6) + # CI geom_errorbarh(aes(xmin = lowerCL, xmax =
# upperCL), height = 0, show.legend = F, size = 1.2) + geom_vline(xintercept =
# 0, linetype = 2, colour = 'black', alpha = 0.5) + geom_point(aes(fill=name),
# size = 3, shape = 21) + annotate('text', x = 0.93, y = (seq(1, mr1_group_no,
# 1)+0.35), label= paste('italic(k)==', mr1_mod_table$K), parse=TRUE, hjust # =
# 'left', size=3.5) + ggplot2::theme_bw() + ggplot2::guides(fill='none',
# colour='none') + ggplot2::theme(axis.text.y = element_text(size=9,
# colour='black', hjust=0.5, angle=90), legend.position = 'none', axis.title.y
# = element_blank(), axis.text.x = element_text(size=9)) +
# scale_y_discrete(labels=c('Arrival_breed' = 'Arrive', 'Depart_breed' =
# 'Depart', 'Nonbreed_arrival' = 'Arrive', # 'Nonbreed_depart' = 'Depart')) +
# \t scale_fill_manual(values=cbpl) + \t scale_colour_manual(values=cbpl) +
# ggplot2::labs(size=paste('Precision (1/SE)'), x=paste('Effect size (ICC)')) +
# annotation_custom(grid::rasterGrob(image_DNB), xmin = -0.45, xmax = -0.25,
# ymin = 3.7, ymax = 4.3) + annotation_custom(grid::rasterGrob(image_ANB), xmin
# = -0.45, xmax = -0.25, ymin = 2.7, ymax = 3.3) +
# annotation_custom(grid::rasterGrob(image_DBchick), xmin = -0.45, xmax =
# -0.25, ymin = 1.7, ymax = 2.3) +
# annotation_custom(grid::rasterGrob(image_ABegg), xmin = -0.45, xmax = -0.25,
# ymin = 0.7, ymax = 1.3) # for Figure 2 b <- ggplot(data = mr1_mod_table,
# aes(x = estimate, y = name)) + scale_x_continuous(limits = c(-0.37, 1),
# breaks = seq(-0.4, 1, by = 0.2), labels = c('-0.4', '-0.2', '0.0', '0.2',
# '0.4', '0.6', '0.8', '1.0')) + ggbeeswarm::geom_quasirandom(data = mr1_data,
# aes(x = yi_ICC, y = moderator, size = scale, colour=moderator),
# groupOnX=FALSE, alpha = 0.4) + geom_errorbarh(aes(xmin = lowerPR, xmax =
# upperPR), height = 0, show.legend = F, size = 0.5, alpha = 0.6) + # CI
# geom_errorbarh(aes(xmin = lowerCL, xmax = upperCL), height = 0, show.legend =
# F, size = 1.2) + geom_vline(xintercept = 0, linetype = 2, colour = 'black',
# alpha = 0.5) + geom_point(aes(fill=name), size = 3, shape = 21) +
# annotate('text', x = 0.93, y = (seq(1, mr1_group_no, 1)+0.35), label=
# paste('italic(k)==', mr1_mod_table$K), parse=TRUE, hjust = 'left', size=3.5)
# + ggplot2::theme_bw() + ggplot2::guides(fill='none', colour='none') +
# ggplot2::theme(axis.text.y = element_text(size=9, colour='black', hjust=0.5,
# angle=90), legend.position = 'none', axis.title = element_blank()) +
# scale_y_discrete(labels=c('Arrival_breed' = 'Arrive', 'Depart_breed' =
# 'Depart', 'Nonbreed_arrival' = 'Arrive', 'Nonbreed_depart' = 'Depart')) + \t
# scale_fill_manual(values=cbpl) + \t scale_colour_manual(values=cbpl) +
# annotation_custom(grid::rasterGrob(image_DNB), xmin = -0.45, xmax = -0.25,
# ymin = 3.7, ymax = 4.3) + annotation_custom(grid::rasterGrob(image_ANB), xmin
# = -0.45, xmax = -0.25, ymin = 2.7, ymax = 3.3) +
# annotation_custom(grid::rasterGrob(image_DBchick), xmin = -0.45, xmax =
# -0.25, ymin = 1.7, ymax = 2.3) +
# annotation_custom(grid::rasterGrob(image_ABegg), xmin = -0.45, xmax = -0.25,
# ymin = 0.7, ymax = 1.3)

# fig_annual_event
```

**Figure 2b.** Repeatability of avian migration timing for annual migration events. The plot shows the group-wise means with 95% confidence intervals (thick lines) and 95% prediction intervals (thin lines), observed effect sizes (back-transformed to ICC) scaled by precision (circles) and k = number of effect sizes. Note this plot was edited in Affinity Designer after construction in R.

#### The effect of tracking method

```
meta_regression2 <- rma.mv(yi = Zr2, V = VCV, mods = ~method, random = list(~1 |
    es_ID, ~1 | paper_ID, ~1 | cohort_ID, ~1 | species_ID, ~1 | phylogeny), R = list(phylogeny = varcor),
    data = df)

# reordering df$method <- factor(df$method, levels = c('Conventional', 'GLS',
# 'Satellite'))

meta_regression2b <- rma.mv(yi = Zr2, V = VCV, mods = ~relevel(method, ref = "GLS"),
    random = list(~1 | es_ID, ~1 | paper_ID, ~1 | cohort_ID, ~1 | species_ID, ~1 |
        phylogeny), R = list(phylogeny = varcor), data = df)

# Orchard plot - need meta-regression without intercept
meta_regression2c <- rma.mv(yi = Zr2, V = VCV, mods = ~method - 1, random = list(~1 |
    es_ID, ~1 | paper_ID, ~1 | cohort_ID, ~1 | species_ID, ~1 | phylogeny), R = list(phylogeny = varcor),
    data = df)
```

#### Table S5

Regression coefficients (Estimate) and 95% confidence intervals (CIs) both in Zr and back-transformed to ICC from the meta-regression with `method`. Note that `mu` means the group mean while `beta` represents the contrast between two groups in the Unit column. *R2marginal* = 5.1%.

```
# getting marginal R2
r2_meta_regression2 <- r2_ml(meta_regression2)

# getting estimates including back-transformation to ICC

mr2 <- mod_results(meta_regression2c, mod = "method")
mr2_mod_table <- mr2$mod_table

mr2_data <- mr2$data

# calculate k for each method separately

# df %>% group_by(method) %>% summarise(mean(k))


mr2_data <- mr2_data %>%
    mutate(k = case_when(moderator == "GLS" ~ 2.2, moderator == "Conventional" ~
        3.13, moderator == "Satellite" ~ 3.28))

mr2_data$yi_ICC <- Zr_to_ICC(mr2_data$yi, mr2_data$k)

mr2_mod_table$k <- c(3.13, 2.2, 3.28)

for (i in names(mr2_mod_table)[2:6]) {

    mr2_mod_table[i] <- Zr_to_ICC(mr2_mod_table[i], mr2_mod_table$k)

}

# creating a table
tibble(`Fixed effect` = c(rep(as.character(mr2_mod_table$name), 2), cont_gen(mr2_mod_table$name)),
    Unit = c(rep(c("Zr (mu)", "ICC (mu)"), each = 3), rep("Zr (beta)", 3)), Estimate = c(meta_regression2c$b,
        mr2_mod_table$estimate, meta_regression2$b[-1], meta_regression2b$b[-(1:2)]),
    `Lower CI [0.025]` = c(meta_regression2c$ci.lb, mr2_mod_table$lowerCL, meta_regression2$ci.lb[-1],
        meta_regression2b$ci.lb[-(1:2)]), `Upper CI  [0.975]` = c(meta_regression2c$ci.ub,
        mr2_mod_table$upperCL, meta_regression2$ci.ub[-1], meta_regression2b$ci.ub[-(1:2)])) ->
    t_method
# `R2` = c(r2_meta_regression2[1], rep(NA,8))) -> t_method

t_method %>%
    kable("html", digits = 3) %>%
    kable_styling("striped", position = "left")
```

| Fixed effect | Unit | Estimate | Lower CI [0.025] | Upper CI [0.975] |
| --- | --- | --- | --- | --- |
| Conventional | Zr (mu) | 0.434 | 0.291 | 0.576 |
| GLS | Zr (mu) | 0.599 | 0.456 | 0.742 |
| Satellite | Zr (mu) | 0.638 | 0.428 | 0.847 |
| Conventional | ICC (mu) | 0.306 | 0.202 | 0.409 |
| GLS | ICC (mu) | 0.512 | 0.404 | 0.608 |
| Satellite | ICC (mu) | 0.440 | 0.292 | 0.575 |
| Conventional-GLS | Zr (beta) | 0.165 | -0.026 | 0.356 |
| Conventional-Satellite | Zr (beta) | 0.204 | -0.049 | 0.457 |
| GLS-Satellite | Zr (beta) | 0.039 | -0.215 | 0.292 |

#### Figure 2c

```
mr2_data$moderator <- factor(mr2_data$moderator, levels = mr2_mod_table$name, labels = mr2_mod_table$name)
mr2_data$scale <- (1/sqrt(mr2_data[,"vi"]))
mr2_mod_table$K <- as.vector(by(mr2_data, mr2_data[,"moderator"], function(x) length(x[,"yi"])))
mr2_group_no <- nrow(mr2_mod_table)

image_conventional <- readPNG(here("images/Conventional_icon.png"))
image_GLS <- readPNG(here("images/GLS_icon.png"))
image_satellite <- readPNG(here("images/Satellite_icon.png"))

# creating orchaRd plot
fig_method <- ggplot(data = mr2_mod_table, aes(x = estimate, y = name)) + 
    scale_x_continuous(limits = c(-0.37, 1), breaks = seq(-0.4, 1, by = 0.2), labels = c("-0.4", "-0.2", "0.0", "0.2", "0.4", "0.6", "0.8", "1.0")) + 
    ggbeeswarm::geom_quasirandom(data = mr2_data, aes(x = yi_ICC, y = moderator, size = scale, colour=moderator), groupOnX=FALSE, alpha = 0.4) +
    geom_errorbarh(aes(xmin = lowerPR, xmax = upperPR), height = 0, show.legend = F, size = 0.5, alpha = 0.6) + # CI
    geom_errorbarh(aes(xmin = lowerCL, xmax = upperCL), height = 0, show.legend = F, size = 1.2) + 
    geom_vline(xintercept = 0, linetype = 2, colour = "black", alpha = 0.5) +
    geom_point(aes(fill=name), size = 3, shape = 21) + 
    annotate('text', x = 0.93, y = (seq(1, mr2_group_no, 1)+0.35), label= paste("italic(k)==", mr2_mod_table$K), parse=TRUE, hjust = "left", size=3.5) +
    scale_color_manual(values = c("Conventional" = "#CC79A7",  "GLS" = "#D55E00",  "Satellite"= "#0072B2")) +
  scale_fill_manual(values = c("Conventional" = "#CC79A7",  "GLS" = "#D55E00",  "Satellite"= "#0072B2")) +
    ggplot2::theme_bw() +
    ggplot2::guides(fill="none", colour="none") +
    ggplot2::theme(axis.text.y = element_text(size=9, colour="black", hjust=0.5, angle=90),
                   legend.position = "none", axis.title.y = element_blank(), axis.text.x=element_text(size=9)) +
    annotation_custom(grid::rasterGrob(image_satellite), xmin = -0.45, xmax = -0.25, ymin = 2.7, ymax = 3.3) +
    annotation_custom(grid::rasterGrob(image_GLS), xmin = -0.45, xmax = -0.25, ymin = 1.7, ymax = 2.3) +
    annotation_custom(grid::rasterGrob(image_conventional), xmin = -0.45, xmax = -0.25, ymin = 0.7, ymax = 1.3) +
  labs(x="Effect size (ICC)")

# for Figure 2

#c <- ggplot(data = mr2_mod_table, aes(x = estimate, y = name)) + 
#    scale_x_continuous(limits = c(-0.37, 1), breaks = seq(-0.4, 1, by = 0.2), labels = c("-0.4", "-0.2", "0.0", "0.2", "0.4", "0.6", "0.8", "1.0")) + 
#    ggbeeswarm::geom_quasirandom(data = mr2_data, aes(x = yi_ICC, y = moderator, size = scale, colour=moderator), groupOnX=FALSE, alpha = 0.4) +
#    geom_errorbarh(aes(xmin = lowerPR, xmax = upperPR), height = 0, show.legend = F, size = 0.5, alpha = 0.6) + # CI
#    geom_errorbarh(aes(xmin = lowerCL, xmax = upperCL), height = 0, show.legend = F, size = 1.2) + 
#    geom_vline(xintercept = 0, linetype = 2, colour = "black", alpha = 0.5) +
#    geom_point(aes(fill=name), size = 3, shape = 21) + 
#    annotate('text', x = 0.93, y = (seq(1, mr2_group_no, 1)+0.35), label= paste("italic(k)==", mr2_mod_table$K), parse=TRUE, hjust = "left", size=3.5) +
#    scale_color_manual(values = c("Conventional" = "#CC79A7",  "GLS" = "#D55E00",  "Satellite"= "#0072B2")) +
#  scale_fill_manual(values = c("Conventional" = "#CC79A7",  "GLS" = "#D55E00",  "Satellite"= "#0072B2")) +
#    ggplot2::theme_bw() +
#    ggplot2::guides(fill="none", colour="none") +
#    ggplot2::theme(axis.text.y = element_text(size=9, colour="black", hjust=0.5, angle=90),
#                   legend.position = "none", axis.title = element_blank()) +
#    annotation_custom(grid::rasterGrob(image_satellite), xmin = -0.45, xmax = -0.25, ymin = 2.7, ymax = 3.3) +
#    annotation_custom(grid::rasterGrob(image_GLS), xmin = -0.45, xmax = -0.25, ymin = 1.7, ymax = 2.3) +
#    annotation_custom(grid::rasterGrob(image_conventional), xmin = -0.45, xmax = -0.25, ymin = 0.7, ymax = 1.3)

fig_method
```

**Figure 2c.** Repeatability of avian migration timing for tracking method. The plot shows the group-wise means with 95% confidence intervals (thick lines) and 95% prediction intervals (thin lines), observed effect sizes (back-transformed to ICC) scaled by precision (circles) and k = number of effect sizes.

#### The effect of sex

```
# reordering df$sex <- factor(df$sex, levels = c('F', 'M', 'B'))

meta_regression4 <- rma.mv(yi = Zr2, V = VCV, mods = ~sex, random = list(~1 | es_ID,
    ~1 | paper_ID, ~1 | cohort_ID, ~1 | species_ID, ~1 | phylogeny), R = list(phylogeny = varcor),
    data = df)

meta_regression4b <- rma.mv(yi = Zr2, V = VCV, mods = ~relevel(sex, ref = "M"), random = list(~1 |
    es_ID, ~1 | paper_ID, ~1 | cohort_ID, ~1 | species_ID, ~1 | phylogeny), R = list(phylogeny = varcor),
    data = df)

# Orchard plot - need meta-regression without intercept
meta_regression4c <- rma.mv(yi = Zr2, V = VCV, mods = ~sex - 1, random = list(~1 |
    es_ID, ~1 | paper_ID, ~1 | cohort_ID, ~1 | species_ID, ~1 | phylogeny), R = list(phylogeny = varcor),
    data = df)
```

#### Table S6

Regression coefficients (Estimate) and 95% confidence intervals (CIs) both in Zr and back-transformed to ICC from the meta-regression with `sex`. Note that `mu` means the group mean while `beta` represents the contrast between two groups in the Unit column. *R2marginal* = 6.9%. B = both sexes, M = male, and F = female.

```
# getting marginal R2
r2_meta_regression4 <- r2_ml(meta_regression4)

# getting estimates including back-transformation to ICC

mr4 <- mod_results(meta_regression4c, mod = "sex")
mr4_mod_table <- mr4$mod_table

mr4_data <- mr4$data

# calculate k for each method separately

# df %>% group_by(sex) %>% summarise(mean(k))


mr4_data <- mr4_data %>%
    mutate(k = case_when(moderator == "F" ~ 2.37, moderator == "M" ~ 2.38, moderator ==
        "B" ~ 2.82))

mr4_data$yi_ICC <- Zr_to_ICC(mr4_data$yi, mr4_data$k)

mr4_mod_table$k <- c(2.37, 2.38, 2.82)

for (i in names(mr4_mod_table)[2:6]) {

    mr4_mod_table[i] <- Zr_to_ICC(mr4_mod_table[i], mr4_mod_table$k)

}

# creating a table
tibble(`Fixed effect` = c(rep(as.character(mr4_mod_table$name), 2), cont_gen(mr4_mod_table$name)),
    Unit = c(rep(c("Zr (mu)", "ICC (mu)"), each = 3), rep("Zr (beta)", 3)), Estimate = c(meta_regression4c$b,
        mr4_mod_table$estimate, meta_regression4$b[-1], meta_regression4b$b[-(1:2)]),
    `Lower CI [0.025]` = c(meta_regression4c$ci.lb, mr4_mod_table$lowerCL, meta_regression4$ci.lb[-1],
        meta_regression4b$ci.lb[-(1:2)]), `Upper CI  [0.975]` = c(meta_regression4c$ci.ub,
        mr4_mod_table$upperCL, meta_regression4$ci.ub[-1], meta_regression4b$ci.ub[-(1:2)])) ->
    t_sex
# `R2` = c(r2_meta_regression4[1], rep(NA,8))) -> t_sex

t_sex %>%
    kable("html", digits = 3) %>%
    kable_styling("striped", position = "left")
```

| Fixed effect | Unit | Estimate | Lower CI [0.025] | Upper CI [0.975] |
| --- | --- | --- | --- | --- |
| B | Zr (mu) | 0.606 | 0.496 | 0.715 |
| F | Zr (mu) | 0.471 | 0.267 | 0.675 |
| M | Zr (mu) | 0.380 | 0.205 | 0.554 |
| B | ICC (mu) | 0.499 | 0.417 | 0.573 |
| F | ICC (mu) | 0.397 | 0.229 | 0.545 |
| M | ICC (mu) | 0.287 | 0.152 | 0.419 |
| B-F | Zr (beta) | -0.135 | -0.359 | 0.089 |
| B-M | Zr (beta) | -0.226 | -0.425 | -0.027 |
| F-M | Zr (beta) | 0.091 | -0.144 | 0.327 |

#### Figure 2e

```
mr4_data$moderator <- factor(mr4_data$moderator, levels = mr4_mod_table$name, labels = mr4_mod_table$name)
mr4_data$scale <- (1/sqrt(mr4_data[,"vi"]))
mr4_mod_table$K <- as.vector(by(mr4_data, mr4_data[,"moderator"], function(x) length(x[,"yi"])))
mr4_group_no <- nrow(mr4_mod_table)

image_male <- readPNG(here("images/Male_icon.png"))
image_female <- readPNG(here("images/Female_icon.png"))
image_both <- readPNG(here("images/Both_sex_icon.png"))

# creating orchaRd plot
# for Figure 2

e <- ggplot(data = mr4_mod_table, aes(x = estimate, y = name)) + 
    scale_x_continuous(limits = c(-0.37, 1), breaks = seq(-0.4, 1, by = 0.2), labels = c("-0.4", "-0.2", "0.0", "0.2", "0.4", "0.6", "0.8", "1.0")) +  
    ggbeeswarm::geom_quasirandom(data = mr4_data, aes(x = yi_ICC, y = moderator, size = scale, colour=moderator), groupOnX=FALSE, alpha = 0.4) +
    geom_errorbarh(aes(xmin = lowerPR, xmax = upperPR), height = 0, show.legend = F, size = 0.5, alpha = 0.6) + # CI
    geom_errorbarh(aes(xmin = lowerCL, xmax = upperCL), height = 0, show.legend = F, size = 1.2) + 
    geom_vline(xintercept = 0, linetype = 2, colour = "black", alpha = 0.5) +
    geom_point(aes(fill=name), size = 3, shape = 21) + 
    annotate('text', x = 0.93, y = (seq(1, mr4_group_no, 1)+0.35), label= paste("italic(k)==", mr4_mod_table$K), parse=TRUE, hjust = "left", size=3.5) +
    ggplot2::theme_bw() +
    ggplot2::guides(fill="none", colour="none") +
    ggplot2::theme(axis.text.y = element_text(size=9, colour="black", hjust=0.5, angle=90),
                   legend.position = "none", axis.title.y = element_blank()) +
    ggplot2::labs(x=paste("Effect size (ICC)")) +
    scale_y_discrete(labels=c("F" = "Female", "M" = "Male", "B" = "Both")) +
              scale_fill_manual(values=cbpl) +
          scale_colour_manual(values=cbpl) +
        annotation_custom(grid::rasterGrob(image_male), xmin = -0.45, xmax = -0.25, ymin = 2.7, ymax = 3.3) +
    annotation_custom(grid::rasterGrob(image_female), xmin = -0.45, xmax = -0.25, ymin = 1.7, ymax = 2.3) +
    annotation_custom(grid::rasterGrob(image_both), xmin = -0.45, xmax = -0.25, ymin = 0.7, ymax = 1.3)

e
```

**Figure 2e.** Repeatability of avian migration timing for sex. The plot shows the group-wise means with 95% confidence intervals (thick lines) and 95% prediction intervals (thin lines), observed effect sizes (back-transformed to ICC) scaled by precision (circles) and k = number of effect sizes.

#### The effect of ecological group

```
meta_regression3 <- rma.mv(yi = Zr2, V = VCV, mods = ~taxa, random = list(~1 | es_ID,
    ~1 | paper_ID, ~1 | cohort_ID, ~1 | species_ID, ~1 | phylogeny), R = list(phylogeny = varcor),
    data = df)

# reordering df$taxa <- factor(df$taxa, levels = c('Waterbird', 'Seabird',
# 'Landbird'))

meta_regression3b <- rma.mv(yi = Zr2, V = VCV, mods = ~relevel(taxa, ref = "Waterbird"),
    random = list(~1 | es_ID, ~1 | paper_ID, ~1 | cohort_ID, ~1 | species_ID, ~1 |
        phylogeny), R = list(phylogeny = varcor), data = df)

# Orchard plot - need meta-regression without intercept
meta_regression3c <- rma.mv(yi = Zr2, V = VCV, mods = ~taxa - 1, random = list(~1 |
    es_ID, ~1 | paper_ID, ~1 | cohort_ID, ~1 | species_ID, ~1 | phylogeny), R = list(phylogeny = varcor),
    data = df)
```

#### Table S7

Regression coefficients (Estimate) and 95% confidence intervals (CIs) both in Zr and back-transformed to ICC from the meta-regression with `taxa`. Note that `mu` means the group mean while `beta` represents the contrast between two groups in the Unit column. *R2marginal* = 6.7%.

```
# getting marginal R2
r2_meta_regression3 <- r2_ml(meta_regression3)

# getting estimates including back-transformation to ICC

mr3 <- mod_results(meta_regression3c, mod = "taxa")
mr3_mod_table <- mr3$mod_table

mr3_data <- mr3$data

# calculate k for each method separately

# df %>% group_by(taxa) %>% summarise(mean(k))

mr3_data <- mr3_data %>%
    mutate(k = case_when(moderator == "Landbird" ~ 2.68, moderator == "Seabird" ~
        2.38, moderator == "Waterbird" ~ 3.08))

mr3_data$yi_ICC <- Zr_to_ICC(mr3_data$yi, mr3_data$k)

mr3_mod_table$k <- c(2.68, 2.38, 3.08)

for (i in names(mr3_mod_table)[2:6]) {

    mr3_mod_table[i] <- Zr_to_ICC(mr3_mod_table[i], mr3_mod_table$k)

}

# creating a table
tibble(`Fixed effect` = c(rep(as.character(mr3_mod_table$name), 2), cont_gen(mr3_mod_table$name)),
    Unit = c(rep(c("Zr (mu)", "ICC (mu)"), each = 3), rep("Zr (beta)", 3)), Estimate = c(meta_regression3c$b,
        mr3_mod_table$estimate, meta_regression3$b[-1], meta_regression3b$b[-(1:2)]),
    `Lower CI [0.025]` = c(meta_regression3c$ci.lb, mr3_mod_table$lowerCL, meta_regression3$ci.lb[-1],
        meta_regression3b$ci.lb[-(1:2)]), `Upper CI  [0.975]` = c(meta_regression3c$ci.ub,
        mr3_mod_table$upperCL, meta_regression3$ci.ub[-1], meta_regression3b$ci.ub[-(1:2)])) ->
    t_method
# `R2` = c(r2_meta_regression3[1], rep(NA,8))) -> t_method

t_method %>%
    kable("html", digits = 3) %>%
    kable_styling("striped", position = "left")
```

| Fixed effect | Unit | Estimate | Lower CI [0.025] | Upper CI [0.975] |
| --- | --- | --- | --- | --- |
| Landbird | Zr (mu) | 0.424 | 0.263 | 0.585 |
| Seabird | Zr (mu) | 0.638 | 0.472 | 0.804 |
| Waterbird | Zr (mu) | 0.564 | 0.405 | 0.722 |
| Landbird | ICC (mu) | 0.333 | 0.205 | 0.454 |
| Seabird | ICC (mu) | 0.520 | 0.398 | 0.626 |
| Waterbird | ICC (mu) | 0.404 | 0.289 | 0.513 |
| Landbird-Seabird | Zr (beta) | 0.214 | -0.017 | 0.445 |
| Landbird-Waterbird | Zr (beta) | 0.140 | -0.086 | 0.366 |
| Seabird-Waterbird | Zr (beta) | 0.074 | -0.155 | 0.304 |

#### Figure 2d

```
mr3_data$moderator <- factor(mr3_data$moderator, levels = mr3_mod_table$name, labels = mr3_mod_table$name)
mr3_data$scale <- (1/sqrt(mr3_data[,"vi"]))
mr3_mod_table$K <- as.vector(by(mr3_data, mr3_data[,"moderator"], function(x) length(x[,"yi"])))
mr3_group_no <- nrow(mr3_mod_table)

image_waterbird <- readPNG(here("images/Waterbird_icon.png"))
image_seabird <- readPNG(here("images/Seabird_icon.png"))
image_landbird <- readPNG(here("images/Landbird_icon.png"))

# creating orchaRd plot

fig_eco_group <- ggplot(data = mr3_mod_table, aes(x = estimate, y = name)) + 
    scale_x_continuous(limits = c(-0.37, 1), breaks = seq(-0.4, 1, by = 0.2), labels = c("-0.4", "-0.2", "0.0", "0.2", "0.4", "0.6", "0.8", "1.0")) + 
    ggbeeswarm::geom_quasirandom(data = mr3_data, aes(x = yi_ICC, y = moderator, size = scale, colour=moderator), groupOnX=FALSE, alpha = 0.4) +
    geom_errorbarh(aes(xmin = lowerPR, xmax = upperPR), height = 0, show.legend = F, size = 0.5, alpha = 0.6) + # CI
    geom_errorbarh(aes(xmin = lowerCL, xmax = upperCL), height = 0, show.legend = F, size = 1.2) + 
    geom_vline(xintercept = 0, linetype = 2, colour = "black", alpha = 0.5) +
    geom_point(aes(fill=name), size = 3, shape = 21) + 
    annotate('text', x = 0.93, y = (seq(1, mr3_group_no, 1)+0.35), label= paste("italic(k)==", mr3_mod_table$K), parse=TRUE, hjust = "left", size=3.5) +
    ggplot2::theme_bw() +
    ggplot2::guides(fill="none", colour="none") +
    ggplot2::theme(axis.text.y = element_text(size=9, colour="black", hjust=0.5, angle=90),
                   legend.position = "none", axis.title.y = element_blank(), axis.text.x=element_text(size=9)) +
    # setting colours
  scale_color_manual(values = c("Seabird" = "#009E73",  "Landbird" = "#D55E00",  "Waterbird"= "#0072B2")) +
  scale_fill_manual(values = c("Seabird" = "#009E73",  "Landbird" = "#D55E00",  "Waterbird"= "#0072B2")) +
    annotation_custom(grid::rasterGrob(image_waterbird), xmin = -0.45, xmax = -0.25, ymin = 2.7, ymax = 3.3) +
    annotation_custom(grid::rasterGrob(image_seabird), xmin = -0.45, xmax = -0.25, ymin = 1.7, ymax = 2.3) +
    annotation_custom(grid::rasterGrob(image_landbird), xmin = -0.45, xmax = -0.25, ymin = 0.7, ymax = 1.3) +
  labs(x="Effect size (ICC)")

fig_eco_group
```

```
# for Figure 2

d <- ggplot(data = mr3_mod_table, aes(x = estimate, y = name)) + 
    scale_x_continuous(limits = c(-0.37,1), breaks = seq(-0.4, 1, by = 0.2), labels = c("-0.4","-0.2", "0.0", "0.2", "0.4", "0.6", "0.8", "1.0")) + 
    ggbeeswarm::geom_quasirandom(data = mr3_data, aes(x = yi_ICC, y = moderator, size = scale, colour=moderator), groupOnX=FALSE, alpha = 0.4) +
    geom_errorbarh(aes(xmin = lowerPR, xmax = upperPR), height = 0, show.legend = F, size = 0.5, alpha = 0.6) + # CI
    geom_errorbarh(aes(xmin = lowerCL, xmax = upperCL), height = 0, show.legend = F, size = 1.2) + 
    geom_vline(xintercept = 0, linetype = 2, colour = "black", alpha = 0.5) + # creating dots and different size (bee-swarm and bubbles)
    geom_point(aes(fill=name), size = 3, shape = 21) + 
    #ggplot2::annotate("text", x = max(mr2_data$yi_ICC) + (max(mr2_data$yi_ICC)*0.10), y = (seq(1, mr2_group_no, 1)+0.3),label = paste("italic(k)==", mr2_mod_table$K), parse = TRUE, hjust = "right", size = 3.5) +
    annotate('text', x = 0.93, y = (seq(1, mr3_group_no, 1)+0.35), label= paste("italic(k)==", mr3_mod_table$K), parse=TRUE, hjust = "left", size=3.5) +
    ggplot2::theme_bw() +
    ggplot2::guides(fill="none", colour="none") +
    ggplot2::theme(axis.text.y = element_text(size=9, colour="black", hjust=0.5, angle=90),
                   legend.position = "none", axis.title = element_blank()) +
    # setting colours
  scale_color_manual(values = c("Seabird" = "#009E73",  "Landbird" = "#D55E00",  "Waterbird"= "#0072B2")) +
  scale_fill_manual(values = c("Seabird" = "#009E73",  "Landbird" = "#D55E00",  "Waterbird"= "#0072B2")) +
    annotation_custom(grid::rasterGrob(image_waterbird), xmin = -0.45, xmax = -0.25, ymin = 2.7, ymax = 3.3) +
    annotation_custom(grid::rasterGrob(image_seabird), xmin = -0.45, xmax = -0.25, ymin = 1.7, ymax = 2.3) +
    annotation_custom(grid::rasterGrob(image_landbird), xmin = -0.45, xmax = -0.25, ymin = 0.7, ymax = 1.3)
```

**Figure 2d.** Repeatability of avian migration timing for ecological group. The plot shows the group-wise means with 95% confidence intervals (thick lines) and 95% prediction intervals (thin lines), observed effect sizes (back-transformed to ICC) scaled by precision (circles) and k = number of effect sizes.

#### Putting together Figure 2

```
# building fig 2 using patchwork

# Figure2 <- (a / b / c / d / e + plot_layout(heights = c(1.8, 3.7, 3, 3, 3)) +
# plot_annotation(tag_levels = 'a', tag_suffix = ')'))

# Figure2

# ggsave(here('figs/Figure_2_R_zero.pdf'), width = 180, height = 300,
# units=c('mm'), dpi=600) edited this file in Affinity Designer
```

**Figure 2.** Putting all five panels together: Figure 2a - Figure 2e (see the main text). Note this plot was edited in Affinity Designer after construction in R.

#### The effect of number of observations per individual (k)

```
meta_regression5 <- rma.mv(yi = Zr2, V = VCV, mods = ~k, random = list(~1 | es_ID,
    ~1 | paper_ID, ~1 | cohort_ID, ~1 | species_ID, ~1 | phylogeny), R = list(phylogeny = varcor),
    data = df)
```

#### Table S8

Regression coefficients (Estimate) and 95% confidence intervals (CIs) in Zr from the meta-regression with `k` (number of observations per individual). *R2marginal* = 0.2%.

```
# getting marginal R2
r2_meta_regression5 <- r2_ml(meta_regression5)

# creating a table
tibble(`Fixed effect` = c("Intercept", "k"), Estimate = c(meta_regression5$b), `Lower CI [0.025]` = c(meta_regression5$ci.lb),
    `Upper CI  [0.975]` = c(meta_regression5$ci.ub)) -> t_k
# `R2` = c(r2_meta_regression5[1], NA)) -> t_k

t_k %>%
    kable("html", digits = 3) %>%
    kable_styling("striped", position = "left")
```

| Fixed effect | Estimate | Lower CI [0.025] | Upper CI [0.975] |
| --- | --- | --- | --- |
| Intercept | 0.563 | 0.358 | 0.769 |
| k | -0.011 | -0.062 | 0.041 |

#### Figure S4

```
pred_meta_regression5 <- predict.rma(meta_regression5)

# creating bubble plot
df %>%
    mutate(fit = pred_meta_regression5$pred, ci.lb = pred_meta_regression5$ci.lb,
        ci.ub = pred_meta_regression5$ci.ub, pr.lb = pred_meta_regression5$cr.lb,
        pr.ub = pred_meta_regression5$cr.ub) %>%
    ggplot(aes(x = k, y = Zr2)) + geom_point(aes(size = (1/sqrt(VZr))), shape = 21,
    alpha = 0.5, fill = "grey85", colour = "grey60", col = "gray25", stroke = 1) +
    geom_line(aes(y = fit), size = 1.5, colour = "darkorchid4") + geom_ribbon(aes(ymin = ci.lb,
    ymax = ci.ub, color = NULL), alpha = 0.3, fill = "darkorchid4") + labs(x = "Number of observations per individual (k)",
    y = "Effect size (Zr)", size = "Precison (1/SE)") + theme_bw() + scale_size_continuous(range = c(1,
    12)) + geom_hline(yintercept = 0, linetype = 2, colour = "black", alpha = 0.5) +
    theme(text = element_text(size = 9, colour = "black", hjust = 0.5), legend.text = element_text(size = 8),
        legend.position = c(1, 1), legend.justification = c(1, 1), legend.background = element_blank(),
        legend.direction = "horizontal", legend.title = element_text(size = 8))
```

**Figure S4.** Repeatability of avian migration timing for the continuous variable `k`, where the solid line represents the model estimate and the shading shows the 95% confidence intervals, with individual data points scaled by precision (1/SE).

```
df2 <- df %>% filter(k < 10)
VCV2 <- impute_covariance_matrix(vi = df2$VZr, cluster = df2$cohort_ID, r = 0.5)

meta_regression6 <- rma.mv(yi = Zr2, V = VCV2, 
                           mods = ~ k,
                           random = list(~1 | es_ID, 
                                         ~1 | paper_ID, 
                                         ~1 | cohort_ID, 
                                         ~1 | species_ID, 
                                         ~1 | phylogeny),
                           R = list(phylogeny = varcor), # added in phylogney
                           data = df2)
```

When we remove the two points with high `k` values (k = 12.4), the results are similar. There is no significant effect (slope = 0.087, 95% CI = [-0.012, 0.187]; *R2marginal* = 4.4%) of ‘k’ on effect sizes, showing that repeatability does not vary with the number of observations per individual.

### Model selection (multi-predictor model)

Here we used the `MuMin` package to generate all possible moderator combinations (using all five variables: `annual_event`, `method`, `sex`, `taxa` & `k` (number of observations per individuals)), determine the importance of the moderators, and generate model-averaged estimates.

```
eval(metafor:::.MuMIn)

full_model_MuMIn <- rma.mv(yi = Zr2, V = VCV, 
                          mods = ~ method + 
                            taxa + 
                            sex + 
                            annual_event +
                            k, 
                          random = list(~1 | es_ID, 
                                        ~1 | paper_ID, 
                                        ~1 | cohort_ID, 
                                        ~1 | species_ID, 
                                        ~1 | phylogeny),
                          R = list(phylogeny = varcor), 
                          method = "ML", # maximum likelihood for model selection
                          data = df)

# vif.rma(full_model_MuMIn)  # No major problems of collinearity (VIF <4)

candidate_models<-dredge(full_model_MuMIn) # Generate all possible combinations of moderators

candidates_aic2 <- subset(candidate_models, delta<=2) # Display all models within 2 values of AICc

importance <- sw(model.avg(candidate_models, subset=delta<=2))# relative importance (sum of weights) of the moderators

mod.avg <- summary(model.avg(candidate_models, subset=delta<=2)) # Generate model-averaged estimates 

confidence <- confint(mod.avg, full=TRUE) # Generate confidence intervals for the estimates averaged using full-averages procedures
```

#### Table S9

The top five models within the \(\Delta\)AIC difference of 2, and which five variables: `annual_event`, `method`, `taxa`, `sex`, & `k` were included (indicated by \(+\)); model weights and the sum of weights for each of the variables are included.

```
# creating a table
tibble(`Model (variable weight)` = c("Model1", "Model2", "Model3", "Model4", "Model5",
    "(Sum of weights)"), annual_event = c(if_else(candidates_aic2$annual_event ==
    "+", "$+$", "NA"), round(importance[1], 3)), taxa = c(if_else(candidates_aic2$taxa ==
    "+", "$+$", "NA"), round(importance[2], 3)), method = c(if_else(candidates_aic2$method ==
    "+", "$+$", "NA"), round(importance[3], 3)), k = c(if_else(candidates_aic2$k <=
    0, "$+$", "NA"), round(importance[4], 3)), sex = c(if_else(candidates_aic2$sex ==
    "+", "$+$", "NA"), round(importance[5], 3)), delta_AICc = c(candidates_aic2$delta,
    NA), Weight = c(candidates_aic2$weight, NA)) %>%
    kable("html", digits = 3) %>%
    kable_styling("striped", position = "left")
```

| Model (variable weight) | annual\_event | taxa | method | k | sex | delta\_AICc | Weight |
| --- | --- | --- | --- | --- | --- | --- | --- |
| Model1 | \(+\) | \(+\) | NA | NA | NA | 0.000 | 0.288 |
| Model2 | \(+\) | NA | NA | NA | NA | 0.353 | 0.242 |
| Model3 | \(+\) | NA | \(+\) | NA | NA | 0.474 | 0.227 |
| Model4 | \(+\) | \(+\) | NA | \(+\) | NA | 1.512 | 0.135 |
| Model5 | \(+\) | NA | NA | NA | \(+\) | 1.978 | 0.107 |
| (Sum of weights) | 1 | 0.424 | 0.227 | 0.135 | 0.107 | NA | NA |

#### Model averaging

#### Table S10

The average estimates for regression coefficients (Estimate) and 95% confidence intervals (CIs) from the model averaging procedure using full-averages (assuming zero values for moderators when they do not occur).

```
# creating a table
tibble(`Fixed effect` = c("Intercept", "Depart_breed", "Nonbreed_arrival", "Nonbreed_depart",
    "Female", "Male", "Seabird", "Waterbird", "GLS", "Satellite", "k"), Estimate = mod.avg$coefmat.full[,
    1], `Lower CI [0.025]` = confidence[, 1], `Upper CI  [0.975]` = confidence[,
    2]) %>%
    kable("html", digits = 3) %>%
    kable_styling("striped", position = "left")
```

| Fixed effect | Estimate | Lower CI [0.025] | Upper CI [0.975] |
| --- | --- | --- | --- |
| Intercept | 0.437 | 0.251 | 0.624 |
| Depart\_breed | -0.090 | -0.236 | 0.056 |
| Nonbreed\_arrival | 0.110 | -0.063 | 0.282 |
| Nonbreed\_depart | 0.241 | 0.081 | 0.402 |
| Female | 0.108 | -0.177 | 0.393 |
| Male | 0.039 | -0.126 | 0.204 |
| Seabird | 0.044 | -0.139 | 0.227 |
| Waterbird | 0.044 | -0.155 | 0.244 |
| GLS | -0.003 | -0.026 | 0.020 |
| Satellite | -0.012 | -0.108 | 0.085 |
| k | -0.019 | -0.143 | 0.105 |

## Sensitivity Analysis

### Variance-covariance matrix with different levels of correlation

Multiple repeatability estimates were measured on the same animals within a paper (cohort ID) which induces a correlation between sampling error variances (Noble et al., 2017). Thus, we constructed variance-covariance matrices to model shared sampling error for effect sizes from the same cohort. We initially assumed a 0.5 correlation, but also ran the phylogenetic meta-analytic model with a 0.25 and 0.75 correlation. All three correlations yielded qualitatively similar results, thus throughout the manuscript we assume a 0.5 correlation, but the results for the other correlation values are presented below.

#### Table S11

Overall effects (meta-analytic means) and 95% confidence intervals (CIs) in *Zr* and heterogeneity, *I*2, for the phylogenetic multilevel intercept-only meta-analysis model when testing different levels of correlation (r = 0.25, 0.50, and 0.75) between sampling variances from the same cohort of birds.

```
# Create a variance-covariance matrix at the cohort level with different correlation values
VCV_25 <- impute_covariance_matrix(vi = df$VZr, cluster = df$cohort_ID, r = 0.25)
VCV_75 <- impute_covariance_matrix(vi = df$VZr, cluster = df$cohort_ID, r = 0.75)

# Run phylogenetic meta-analytic models with 0.25 and 0.75 VCV
ma_model2_VCV25 <- rma.mv(yi = Zr2, V = VCV_25, 
                      random = list(~1 | es_ID, 
                                    ~1 | paper_ID, 
                                    ~1 | cohort_ID, 
                                    ~1 | species_ID, # non-phylo effect
                                    ~1 | phylogeny), # phylo effect
                      R = list(phylogeny = varcor), # phylogenetic relatedness
                      data = df)

i2_ma2_VCV25 <- round(i2_ml(ma_model2_VCV25)*100,1)

ma_model2_VCV75 <- rma.mv(yi = Zr2, V = VCV_75, 
                      random = list(~1 | es_ID, 
                                    ~1 | paper_ID, 
                                    ~1 | cohort_ID, 
                                    ~1 | species_ID, # non-phylo effect
                                    ~1 | phylogeny), # phylo effect
                      R = list(phylogeny = varcor), # phylogenetic relatedness
                      data = df)

i2_ma2_VCV75 <- round(i2_ml(ma_model2_VCV75)*100,1)

# creating a table
tibble(
  Model = c("Meta-analysis, r=0.25", "Meta-analysis, r=0.50", "Meta-analysis, r=0.75"),
  `Overall mean` = c(ma_model2_VCV25$b, ma_model2$b, ma_model2_VCV75$b),
  `Lower CI [0.025]` = c(ma_model2_VCV25$ci.lb, ma_model2$ci.lb, ma_model2_VCV75$ci.lb),
  `Upper CI [0.975]` = c(ma_model2_VCV25$ci.ub, ma_model2$ci.ub, ma_model2_VCV75$ci.ub),
  `I^2^~total~` = c(i2_ma2_VCV25[1], i2_ma2[1], i2_ma2_VCV75[1]),
  `I^2^~es~` = c(i2_ma2_VCV25[2], i2_ma2[2], i2_ma2_VCV75[2]),
  `I^2^~paper~` = c(i2_ma2_VCV25[3], i2_ma2[3], i2_ma2_VCV75[3]),
  `I^2^~cohort~` = c(i2_ma2_VCV25[4], i2_ma2[4], i2_ma2_VCV75[4]),
  `I^2^~species~` = c(i2_ma2_VCV25[5], i2_ma2[5], i2_ma2_VCV75[5]),
  `I^2^~phylo~` = c(i2_ma2_VCV25[6], i2_ma2[6], i2_ma2_VCV75[6]),) %>% 
  kable("html",  digits = 3) %>%
  kable_styling("striped", position = "left")
```

| Model | Overall mean | Lower CI [0.025] | Upper CI [0.975] | I2total | I2es | I2paper | I2cohort | I2species | I2phylo |
| --- | --- | --- | --- | --- | --- | --- | --- | --- | --- |
| Meta-analysis, r=0.25 | 0.535 | 0.405 | 0.664 | 82.8 | 40.6 | 0 | 0 | 34.3 | 7.8 |
| Meta-analysis, r=0.50 | 0.532 | 0.400 | 0.664 | 84.2 | 49.7 | 0 | 0 | 27.3 | 7.2 |
| Meta-analysis, r=0.75 | 0.530 | 0.396 | 0.663 | 86.2 | 60.5 | 0 | 0 | 19.6 | 6.1 |

### Inclusion of negative repeatabiliy estimates

Correlation- and ANOVA-based repeatabilities can produce negative values, often reflecting noise around a statistical zero (Nakagawa & Schielzeth, 2010). For our main analyses, we set these negative estimates to zero, however here, we re-ran all the meta-analytic and meta-regression models with these negative values included.

#### Table S12

Overall effects (meta-analytic means) and 95% confidence intervals (CIs) both in *Zr* and back-transformed to *ICC*, and heterogeneity, *I*2, for the multilevel intercept-only meta-analysis models including and excluding phylogeny when negative repeatability values are included.

```
ma_model1_neg <- rma.mv(yi = Zr, V = VCV,
                    random = list(~1 | es_ID, 
                                  ~1 | paper_ID, 
                                  ~1 | cohort_ID, 
                                  ~1 | species_ID), 
                    data = df)

ma_model2_neg <- rma.mv(yi = Zr, V = VCV, 
                    random = list(~1 | es_ID, 
                                  ~1 | paper_ID, 
                                  ~1 | cohort_ID, 
                                  ~1 | species_ID, # non-phylo effect
                                  ~1 | phylogeny), # phylo effect
                    R = list(phylogeny = varcor), # phylogenetic relatedness
                    data = df)

## # estimating I2 as measure of heterogeneity
i2_ma1_neg <- round(i2_ml(ma_model1_neg)*100,1)
i2_ma2_neg <- round(i2_ml(ma_model2_neg)*100,1)

# Back-transform to ICC
ma1_neg <- mod_results(ma_model1_neg, mod="Int")
ma1_mod_table_neg <- ma1_neg$mod_table

ma2_neg <- mod_results(ma_model2_neg, mod="Int")
ma2_mod_table_neg <- ma2_neg$mod_table

# need to calculate k for whole data set to use in formula

k_all <- mean(df$k)

for(i in names(ma1_mod_table_neg)[2:6]){
  
  ma1_mod_table_neg[i] <- Zr_to_ICC(ma1_mod_table_neg[i], k_all)
  
}

for(i in names(ma2_mod_table_neg)[2:6]){
  
  ma2_mod_table_neg[i] <- Zr_to_ICC(ma2_mod_table_neg[i], k_all)
  
}

# creating a table
tibble(
  Model = c("Meta-analysis (Zr)", "Meta-analysis (ICC)", "Meta-analysis phylo (Zr)", "Meta-analysis phylo (ICC)"),
  `Overall mean` = c(ma_model1_neg$b, ma1_mod_table_neg$estimate, ma_model2_neg$b, ma2_mod_table_neg$estimate),
  `Lower CI [0.025]` = c(ma_model1_neg$ci.lb, ma1_mod_table_neg$lowerCL, ma_model2_neg$ci.lb, ma2_mod_table_neg$lowerCL),
  `Upper CI [0.975]` = c(ma_model1_neg$ci.ub, ma1_mod_table_neg$upperCL, ma_model2_neg$ci.ub, ma2_mod_table_neg$upperCL),
  `I^2^~total~` = c(i2_ma1_neg[1], NA, i2_ma2_neg[1], NA),
  `I^2^~es~` = c(i2_ma1_neg[2], NA, i2_ma2_neg[2], NA),
  `I^2^~paper~` = c(i2_ma1_neg[3], NA, i2_ma2_neg[3], NA),
  `I^2^~cohort~` = c(i2_ma1_neg[4], NA, i2_ma2_neg[4], NA),
  `I^2^~species~` = c(i2_ma1_neg[5], NA, i2_ma2_neg[5], NA),
  `I^2^~phylo~` = c(NA, NA, i2_ma2_neg[6], NA),) %>% 
  kable("html",  digits = 3) %>%
  kable_styling("striped", position = "left")
```

| Model | Overall mean | Lower CI [0.025] | Upper CI [0.975] | I2total | I2es | I2paper | I2cohort | I2species | I2phylo |
| --- | --- | --- | --- | --- | --- | --- | --- | --- | --- |
| Meta-analysis (Zr) | 0.529 | 0.429 | 0.629 | 85.3 | 51.2 | 0 | 0 | 34.2 | NA |
| Meta-analysis (ICC) | 0.412 | 0.336 | 0.484 | NA | NA | NA | NA | NA | NA |
| Meta-analysis phylo (Zr) | 0.524 | 0.397 | 0.650 | 85.5 | 50.6 | 0 | 0 | 30.2 | 4.8 |
| Meta-analysis phylo (ICC) | 0.408 | 0.311 | 0.498 | NA | NA | NA | NA | NA | NA |

```
# meta-regression: mutiple intercepts
meta_regression1_neg <- rma.mv(yi = Zr, V = VCV, 
                           mods = ~ annual_event,
                           random = list(~1 | es_ID, 
                                         ~1 | paper_ID, 
                                         ~1 | cohort_ID, 
                                         ~1 | species_ID, 
                                         ~1 | phylogeny),
                           R = list(phylogeny = varcor), # added in phylogney
                           data = df)

meta_regression1b_neg <- rma.mv(yi = Zr, V = VCV, 
                            mods = ~ relevel(annual_event, ref = "Depart_breed"),
                            random = list(~1 | es_ID, 
                                          ~1 | paper_ID, 
                                          ~1 | cohort_ID, 
                                          ~1 | species_ID, 
                                          ~1 | phylogeny),
                            R = list(phylogeny = varcor), # added in phylogney
                            data = df)


meta_regression1c_neg <- rma.mv(yi = Zr, V = VCV, 
                            mods = ~ relevel(annual_event, ref = "Nonbreed_depart"),
                            random = list(~1 | es_ID, 
                                          ~1 | paper_ID, 
                                          ~1 | cohort_ID, 
                                          ~1 | species_ID, 
                                          ~1 | phylogeny),
                            R = list(phylogeny = varcor), # added in phylogney
                            data = df)

# meta-regression: contrast (for orchard plot)
meta_regression1d_neg <- rma.mv(yi = Zr, V = VCV, 
                            mods = ~ annual_event -1,
                            random = list(~1 | es_ID, 
                                          ~1 | paper_ID, 
                                          ~1 | cohort_ID, 
                                          ~1 | species_ID, 
                                          ~1 | phylogeny),
                            R = list(phylogeny = varcor), # added in phylogney
                            data = df)
```

#### Table S13

Regression coefficients (Estimate) and 95% confidence intervals (CIs) both in Zr and back-transformed to ICC from the meta-regression with `annual_event` when negative repeatability values are included. Note that `mu` means the group mean while `beta` represents the contrast between two groups in the Unit column. *R2marginal* = 10.9%.

```
# getting marginal R2
r2_meta_regression1_neg <- r2_ml(meta_regression1_neg)

# getting estimates including back-transformation to ICC

mr1_neg <- mod_results(meta_regression1d_neg, mod = "annual_event")
mr1_mod_table_neg <- mr1_neg$mod_table

mr1_data_neg <- mr1_neg$data

# calculate k for each method separately

# df %>% group_by(annual_event) %>% summarise(mean(k))

mr1_data_neg <- mr1_data_neg %>%
    mutate(k = case_when(moderator == "Arrival_breed" ~ 2.55, moderator == "Depart_breed" ~
        2.45, moderator == "Nonbreed_arrival" ~ 3.2, moderator == "Nonbreed_depart" ~
        2.94))

mr1_data_neg$yi_ICC <- Zr_to_ICC(mr1_data_neg$yi, mr1_data_neg$k)

mr1_mod_table_neg$k <- c(2.55, 2.45, 3.2, 2.94)

for (i in names(mr1_mod_table_neg)[2:6]) {

    mr1_mod_table_neg[i] <- Zr_to_ICC(mr1_mod_table_neg[i], mr1_mod_table_neg$k)

}

# creating a table
tibble(`Fixed effect` = c(rep(as.character(mr1_mod_table_neg$name), 2), cont_gen(mr1_mod_table_neg$name)),
    Unit = c(rep(c("Zr (mu)", "ICC (mu)"), each = 4), rep("Zr (beta)", 6)), Estimate = c(meta_regression1d_neg$b,
        mr1_mod_table_neg$estimate, meta_regression1_neg$b[-1], meta_regression1b_neg$b[-(1:2)],
        meta_regression1c_neg$b[-(1:3)]), `Lower CI [0.025]` = c(meta_regression1d_neg$ci.lb,
        mr1_mod_table_neg$lowerCL, meta_regression1_neg$ci.lb[-1], meta_regression1b_neg$ci.lb[-(1:2)],
        meta_regression1c_neg$ci.lb[-(1:3)]), `Upper CI  [0.975]` = c(meta_regression1d_neg$ci.ub,
        mr1_mod_table_neg$upperCL, meta_regression1_neg$ci.ub[-1], meta_regression1b_neg$ci.ub[-(1:2)],
        meta_regression1c_neg$ci.ub[-(1:3)])) -> t_annual_event_neg
# `R2` = c(r2_meta_regression1[1], rep(NA,13))) ->

t_annual_event_neg %>%
    kable("html", digits = 3) %>%
    kable_styling("striped", position = "left")
```

| Fixed effect | Unit | Estimate | Lower CI [0.025] | Upper CI [0.975] |
| --- | --- | --- | --- | --- |
| Arrival\_breed | Zr (mu) | 0.477 | 0.314 | 0.640 |
| Depart\_breed | Zr (mu) | 0.352 | 0.168 | 0.536 |
| Nonbreed\_arrival | Zr (mu) | 0.592 | 0.394 | 0.790 |
| Nonbreed\_depart | Zr (mu) | 0.712 | 0.523 | 0.901 |
| Arrival\_breed | ICC (mu) | 0.385 | 0.256 | 0.505 |
| Depart\_breed | ICC (mu) | 0.294 | 0.140 | 0.440 |
| Nonbreed\_arrival | ICC (mu) | 0.414 | 0.272 | 0.546 |
| Nonbreed\_depart | ICC (mu) | 0.518 | 0.386 | 0.632 |
| Arrival\_breed-Depart\_breed | Zr (beta) | -0.125 | -0.273 | 0.022 |
| Arrival\_breed-Nonbreed\_arrival | Zr (beta) | 0.114 | -0.060 | 0.289 |
| Arrival\_breed-Nonbreed\_depart | Zr (beta) | 0.235 | 0.071 | 0.399 |
| Depart\_breed-Nonbreed\_arrival | Zr (beta) | 0.240 | 0.055 | 0.424 |
| Depart\_breed-Nonbreed\_depart | Zr (beta) | 0.360 | 0.183 | 0.537 |
| Nonbreed\_arrival-Nonbreed\_depart | Zr (beta) | -0.120 | -0.301 | 0.060 |

```
meta_regression2_neg <- rma.mv(yi = Zr, V = VCV, mods = ~method, random = list(~1 |
    es_ID, ~1 | paper_ID, ~1 | cohort_ID, ~1 | species_ID, ~1 | phylogeny), R = list(phylogeny = varcor),
    data = df)

# reordering df$method <- factor(df$method, levels = c('Conventional', 'GLS',
# 'Satellite'))

meta_regression2b_neg <- rma.mv(yi = Zr, V = VCV, mods = ~relevel(method, ref = "GLS"),
    random = list(~1 | es_ID, ~1 | paper_ID, ~1 | cohort_ID, ~1 | species_ID, ~1 |
        phylogeny), R = list(phylogeny = varcor), data = df)

# Orchard plot - need meta-regression without intercept
meta_regression2c_neg <- rma.mv(yi = Zr, V = VCV, mods = ~method - 1, random = list(~1 |
    es_ID, ~1 | paper_ID, ~1 | cohort_ID, ~1 | species_ID, ~1 | phylogeny), R = list(phylogeny = varcor),
    data = df)
```

#### Table S14

Regression coefficients (Estimate) and 95% confidence intervals (CIs) both in Zr and back-transformed to ICC from the meta-regression with `method` when negative repeatability values are included. Note that `mu` means the group mean while `beta` represents the contrast between two groups in the Unit column. *R2marginal* = 4.2%.

```
# getting marginal R2
r2_meta_regression2_neg <- r2_ml(meta_regression2_neg)

# getting estimates including back-transformation to ICC

mr2_neg <- mod_results(meta_regression2c_neg, mod = "method")
mr2_mod_table_neg <- mr2_neg$mod_table

mr2_data_neg <- mr2_neg$data

# calculate k for each method separately

# df %>% group_by(method) %>% summarise(mean(k))


mr2_data_neg <- mr2_data_neg %>%
    mutate(k = case_when(moderator == "GLS" ~ 2.2, moderator == "Conventional" ~
        3.13, moderator == "Satellite" ~ 3.28))

mr2_data_neg$yi_ICC <- Zr_to_ICC(mr2_data_neg$yi, mr2_data_neg$k)

mr2_mod_table_neg$k <- c(3.13, 2.2, 3.28)

for (i in names(mr2_mod_table_neg)[2:6]) {

    mr2_mod_table_neg[i] <- Zr_to_ICC(mr2_mod_table_neg[i], mr2_mod_table_neg$k)

}

# creating a table
tibble(`Fixed effect` = c(rep(as.character(mr2_mod_table_neg$name), 2), cont_gen(mr2_mod_table_neg$name)),
    Unit = c(rep(c("Zr (mu)", "ICC (mu)"), each = 3), rep("Zr (beta)", 3)), Estimate = c(meta_regression2c_neg$b,
        mr2_mod_table_neg$estimate, meta_regression2_neg$b[-1], meta_regression2b_neg$b[-(1:2)]),
    `Lower CI [0.025]` = c(meta_regression2c_neg$ci.lb, mr2_mod_table_neg$lowerCL,
        meta_regression2_neg$ci.lb[-1], meta_regression2b_neg$ci.lb[-(1:2)]), `Upper CI  [0.975]` = c(meta_regression2c_neg$ci.ub,
        mr2_mod_table_neg$upperCL, meta_regression2_neg$ci.ub[-1], meta_regression2b_neg$ci.ub[-(1:2)])) ->
    t_method_neg

t_method_neg %>%
    kable("html", digits = 3) %>%
    kable_styling("striped", position = "left")
```

| Fixed effect | Unit | Estimate | Lower CI [0.025] | Upper CI [0.975] |
| --- | --- | --- | --- | --- |
| Conventional | Zr (mu) | 0.429 | 0.278 | 0.579 |
| GLS | Zr (mu) | 0.574 | 0.425 | 0.723 |
| Satellite | Zr (mu) | 0.633 | 0.415 | 0.851 |
| Conventional | ICC (mu) | 0.302 | 0.192 | 0.411 |
| GLS | ICC (mu) | 0.494 | 0.379 | 0.596 |
| Satellite | ICC (mu) | 0.437 | 0.283 | 0.577 |
| Conventional-GLS | Zr (beta) | 0.145 | -0.054 | 0.345 |
| Conventional-Satellite | Zr (beta) | 0.204 | -0.060 | 0.469 |
| GLS-Satellite | Zr (beta) | 0.059 | -0.205 | 0.323 |

```
# reordering df$sex <- factor(df$sex, levels = c('F', 'M', 'B'))

meta_regression4_neg <- rma.mv(yi = Zr, V = VCV, mods = ~sex, random = list(~1 |
    es_ID, ~1 | paper_ID, ~1 | cohort_ID, ~1 | species_ID, ~1 | phylogeny), R = list(phylogeny = varcor),
    data = df)

meta_regression4b_neg <- rma.mv(yi = Zr, V = VCV, mods = ~relevel(sex, ref = "M"),
    random = list(~1 | es_ID, ~1 | paper_ID, ~1 | cohort_ID, ~1 | species_ID, ~1 |
        phylogeny), R = list(phylogeny = varcor), data = df)

# Orchard plot - need meta-regression without intercept
meta_regression4c_neg <- rma.mv(yi = Zr, V = VCV, mods = ~sex - 1, random = list(~1 |
    es_ID, ~1 | paper_ID, ~1 | cohort_ID, ~1 | species_ID, ~1 | phylogeny), R = list(phylogeny = varcor),
    data = df)
```

#### Table S15

Regression coefficients (Estimate) and 95% confidence intervals (CIs) both in Zr and back-transformed to ICC from the meta-regression with `sex` when negative repeatability values are included. Note that `mu` means the group mean while `beta` represents the contrast between two groups in the Unit column. *R2marginal* = 8%. B = both sexes, M = male, and F = female.

```
# getting marginal R2
r2_meta_regression4_neg <- r2_ml(meta_regression4_neg)

# getting estimates including back-transformation to ICC

mr4_neg <- mod_results(meta_regression4c_neg, mod = "sex")
mr4_mod_table_neg <- mr4_neg$mod_table

mr4_data_neg <- mr4_neg$data

# calculate k for each method separately

# df %>% group_by(sex) %>% summarise(mean(k))


mr4_data_neg <- mr4_data_neg %>%
    mutate(k = case_when(moderator == "F" ~ 2.37, moderator == "M" ~ 2.38, moderator ==
        "B" ~ 2.82))

mr4_data_neg$yi_ICC <- Zr_to_ICC(mr4_data_neg$yi, mr4_data_neg$k)

mr4_mod_table_neg$k <- c(2.37, 2.38, 2.82)

for (i in names(mr4_mod_table_neg)[2:6]) {

    mr4_mod_table_neg[i] <- Zr_to_ICC(mr4_mod_table_neg[i], mr4_mod_table_neg$k)

}

# creating a table
tibble(`Fixed effect` = c(rep(as.character(mr4_mod_table_neg$name), 2), cont_gen(mr4_mod_table_neg$name)),
    Unit = c(rep(c("Zr (mu)", "ICC (mu)"), each = 3), rep("Zr (beta)", 3)), Estimate = c(meta_regression4c_neg$b,
        mr4_mod_table_neg$estimate, meta_regression4_neg$b[-1], meta_regression4b_neg$b[-(1:2)]),
    `Lower CI [0.025]` = c(meta_regression4c_neg$ci.lb, mr4_mod_table_neg$lowerCL,
        meta_regression4_neg$ci.lb[-1], meta_regression4b_neg$ci.lb[-(1:2)]), `Upper CI  [0.975]` = c(meta_regression4c_neg$ci.ub,
        mr4_mod_table_neg$upperCL, meta_regression4_neg$ci.ub[-1], meta_regression4b_neg$ci.ub[-(1:2)])) ->
    t_sex_neg

t_sex_neg %>%
    kable("html", digits = 3) %>%
    kable_styling("striped", position = "left")
```

| Fixed effect | Unit | Estimate | Lower CI [0.025] | Upper CI [0.975] |
| --- | --- | --- | --- | --- |
| B | Zr (mu) | 0.602 | 0.489 | 0.715 |
| F | Zr (mu) | 0.450 | 0.239 | 0.661 |
| M | Zr (mu) | 0.347 | 0.166 | 0.527 |
| B | ICC (mu) | 0.496 | 0.412 | 0.573 |
| F | ICC (mu) | 0.380 | 0.205 | 0.536 |
| M | ICC (mu) | 0.262 | 0.122 | 0.399 |
| B-F | Zr (beta) | -0.153 | -0.384 | 0.079 |
| B-M | Zr (beta) | -0.256 | -0.462 | -0.050 |
| F-M | Zr (beta) | 0.103 | -0.141 | 0.347 |

```
meta_regression3_neg <- rma.mv(yi = Zr, V = VCV, mods = ~taxa, random = list(~1 |
    es_ID, ~1 | paper_ID, ~1 | cohort_ID, ~1 | species_ID, ~1 | phylogeny), R = list(phylogeny = varcor),
    data = df)

# reordering df$taxa <- factor(df$taxa, levels = c('Waterbird', 'Seabird',
# 'Landbird'))

meta_regression3b_neg <- rma.mv(yi = Zr, V = VCV, mods = ~relevel(taxa, ref = "Waterbird"),
    random = list(~1 | es_ID, ~1 | paper_ID, ~1 | cohort_ID, ~1 | species_ID, ~1 |
        phylogeny), R = list(phylogeny = varcor), data = df)

# Orchard plot - need meta-regression without intercept
meta_regression3c_neg <- rma.mv(yi = Zr, V = VCV, mods = ~taxa - 1, random = list(~1 |
    es_ID, ~1 | paper_ID, ~1 | cohort_ID, ~1 | species_ID, ~1 | phylogeny), R = list(phylogeny = varcor),
    data = df)
```

#### Table S16

Regression coefficients (Estimate) and 95% confidence intervals (CIs) both in Zr and back-transformed to ICC from the meta-regression with `taxa` when negative repeatability values are included. Note that `mu` means the group mean while `beta` represents the contrast between two groups in the Unit column. *R2marginal* = 5.1%.

```
# getting marginal R2
r2_meta_regression3_neg <- r2_ml(meta_regression3_neg)

# getting estimates including back-transformation to ICC

mr3_neg <- mod_results(meta_regression3c_neg, mod = "taxa")
mr3_mod_table_neg <- mr3_neg$mod_table

mr3_data_neg <- mr3_neg$data

# calculate k for each method separately

# df %>% group_by(taxa) %>% summarise(mean(k))

mr3_data_neg <- mr3_data_neg %>%
    mutate(k = case_when(moderator == "Landbird" ~ 2.68, moderator == "Seabird" ~
        2.38, moderator == "Waterbird" ~ 3.08))

mr3_data_neg$yi_ICC <- Zr_to_ICC(mr3_data_neg$yi, mr3_data_neg$k)

mr3_mod_table_neg$k <- c(2.68, 2.38, 3.08)

for (i in names(mr3_mod_table_neg)[2:6]) {

    mr3_mod_table_neg[i] <- Zr_to_ICC(mr3_mod_table_neg[i], mr3_mod_table_neg$k)

}

# creating a table
tibble(`Fixed effect` = c(rep(as.character(mr3_mod_table_neg$name), 2), cont_gen(mr3_mod_table_neg$name)),
    Unit = c(rep(c("Zr (mu)", "ICC (mu)"), each = 3), rep("Zr (beta)", 3)), Estimate = c(meta_regression3c_neg$b,
        mr3_mod_table_neg$estimate, meta_regression3_neg$b[-1], meta_regression3b_neg$b[-(1:2)]),
    `Lower CI [0.025]` = c(meta_regression3c_neg$ci.lb, mr3_mod_table_neg$lowerCL,
        meta_regression3_neg$ci.lb[-1], meta_regression3b_neg$ci.lb[-(1:2)]), `Upper CI  [0.975]` = c(meta_regression3c_neg$ci.ub,
        mr3_mod_table_neg$upperCL, meta_regression3_neg$ci.ub[-1], meta_regression3b_neg$ci.ub[-(1:2)])) ->
    t_method_neg

t_method_neg %>%
    kable("html", digits = 3) %>%
    kable_styling("striped", position = "left")
```

| Fixed effect | Unit | Estimate | Lower CI [0.025] | Upper CI [0.975] |
| --- | --- | --- | --- | --- |
| Landbird | Zr (mu) | 0.417 | 0.247 | 0.587 |
| Seabird | Zr (mu) | 0.612 | 0.438 | 0.786 |
| Waterbird | Zr (mu) | 0.562 | 0.394 | 0.729 |
| Landbird | ICC (mu) | 0.327 | 0.193 | 0.454 |
| Seabird | ICC (mu) | 0.502 | 0.371 | 0.616 |
| Waterbird | ICC (mu) | 0.403 | 0.280 | 0.517 |
| Landbird-Seabird | Zr (beta) | 0.195 | -0.048 | 0.438 |
| Landbird-Waterbird | Zr (beta) | 0.145 | -0.094 | 0.383 |
| Seabird-Waterbird | Zr (beta) | 0.050 | -0.191 | 0.292 |

```
meta_regression5_neg <- rma.mv(yi = Zr, V = VCV, mods = ~k, random = list(~1 | es_ID,
    ~1 | paper_ID, ~1 | cohort_ID, ~1 | species_ID, ~1 | phylogeny), R = list(phylogeny = varcor),
    data = df)
```

#### Table S17

Regression coefficients (Estimate) and 95% confidence intervals (CIs) in Zr from the meta-regression with `k` (number of observations per individual) when negative repeatability values are included. *R2marginal* = 0.1%.

```
# getting marginal R2
r2_meta_regression5_neg <- r2_ml(meta_regression5_neg)

# creating a table
tibble(`Fixed effect` = c("Intercept", "k"), Estimate = c(meta_regression5_neg$b),
    `Lower CI [0.025]` = c(meta_regression5_neg$ci.lb), `Upper CI  [0.975]` = c(meta_regression5_neg$ci.ub)) ->
    t_k_neg

t_k_neg %>%
    kable("html", digits = 3) %>%
    kable_styling("striped", position = "left")
```

| Fixed effect | Estimate | Lower CI [0.025] | Upper CI [0.975] |
| --- | --- | --- | --- |
| Intercept | 0.542 | 0.336 | 0.749 |
| k | -0.006 | -0.061 | 0.048 |

```
VCV2 <- impute_covariance_matrix(vi = df2$VZr, cluster = df2$cohort_ID, r = 0.5)

meta_regression6_neg <- rma.mv(yi = Zr, V = VCV2, 
                           mods = ~ k,
                           random = list(~1 | es_ID, 
                                         ~1 | paper_ID, 
                                         ~1 | cohort_ID, 
                                         ~1 | species_ID, 
                                         ~1 | phylogeny),
                           R = list(phylogeny = varcor), # added in phylogney
                           data = df2)
```

### Model selection with negative repeatability estimates included

Here we used the `MuMin` package to generate all possible moderator combinations (using all five variables: `annual_event`, `method`, `sex`, `taxa` & `k` (number of observations per individuals)), determine the importance of the moderators, and generate model-averaged estimates when the negative repeatability estimates are included.

```
eval(metafor:::.MuMIn)

full_model_MuMIn_neg <- rma.mv(yi = Zr, V = VCV, 
                          mods = ~ method + 
                            taxa + 
                            sex + 
                            annual_event +
                            k, 
                          random = list(~1 | es_ID, 
                                        ~1 | paper_ID, 
                                        ~1 | cohort_ID, 
                                        ~1 | species_ID, 
                                        ~1 | phylogeny),
                          R = list(phylogeny = varcor), 
                          method = "ML", # maximum likelihood for model selection
                          data = df)

# vif.rma(full_model_MuMIn)  # No major problems of collinearity (VIF <4)

candidate_models_neg <-dredge(full_model_MuMIn_neg) # Generate all possible combinations of moderators

candidates_aic2_neg <- subset(candidate_models_neg, delta<=2) # Display all models within 2 values of AICc

importance_neg <- sw(model.avg(candidate_models_neg, subset=delta<=2))# relative importance (sum of weights) of the moderators

mod.avg_neg <- summary(model.avg(candidate_models_neg, subset=delta<=2)) # Generate model-averaged estimates 

confidence_neg <- confint(mod.avg_neg, full=TRUE) # Generate confidence intervals for the estimates averaged using full-averages procedures
```

#### Table S18

The top six models (when negative repeatability values are included) within the \(\Delta\)AIC difference of 2, and which five variables: `annual_event`, `method`, `taxa`, `sex`, & `k` were included (indicated by \(+\)); model weights and the sum of weights for each of the variables are included.

```
# creating a table
tibble(`Model (variable weight)` = c("Model1", "Model2", "Model3", "Model4", "Model5",
    "Model6", "(Sum of weights)"), annual_event = c(if_else(candidates_aic2_neg$annual_event ==
    "+", "$+$", "NA"), round(importance_neg[1], 3)), sex = c(if_else(candidates_aic2_neg$sex ==
    "+", "$+$", "NA"), round(importance_neg[2], 3)), taxa = c(if_else(candidates_aic2_neg$taxa ==
    "+", "$+$", "NA"), round(importance_neg[3], 3)), method = c(if_else(candidates_aic2_neg$method ==
    "+", "$+$", "NA"), round(importance_neg[4], 3)), k = c(if_else(candidates_aic2_neg$k <=
    0, "$+$", "NA"), round(importance_neg[5], 3)), delta_AICc = c(candidates_aic2_neg$delta,
    NA), Weight = c(candidates_aic2_neg$weight, NA)) %>%
    kable("html", digits = 3) %>%
    kable_styling("striped", position = "left")
```

| Model (variable weight) | annual\_event | sex | taxa | method | k | delta\_AICc | Weight |
| --- | --- | --- | --- | --- | --- | --- | --- |
| Model1 | \(+\) | NA | NA | NA | NA | 0.000 | 0.241 |
| Model2 | \(+\) | NA | \(+\) | NA | NA | 0.428 | 0.195 |
| Model3 | \(+\) | NA | NA | \(+\) | NA | 0.434 | 0.194 |
| Model4 | \(+\) | \(+\) | NA | NA | NA | 0.515 | 0.186 |
| Model5 | \(+\) | \(+\) | NA | NA | \(+\) | 1.921 | 0.092 |
| Model6 | \(+\) | NA | NA | NA | \(+\) | 1.955 | 0.091 |
| (Sum of weights) | 1 | 0.279 | 0.195 | 0.194 | 0.183 | NA | NA |

#### Model averaging

#### Table S19

The average estimates for regression coefficients (Estimate) and 95% confidence intervals (CIs) from the model averaging procedure when negative repeatability values are included using full-averages (assuming zero values for moderators when they do not occur).

```
# creating a table
tibble(`Fixed effect` = c("Intercept", "Depart_breed", "Nonbreed_arrival", "Nonbreed_depart",
    "Seabird", "Waterbird", "GLS", "Satellite", "k", "Female", "Male"), Estimate = mod.avg_neg$coefmat.full[,
    1], `Lower CI [0.025]` = confidence_neg[, 1], `Upper CI  [0.975]` = confidence_neg[,
    2]) %>%
    kable("html", digits = 3) %>%
    kable_styling("striped", position = "left")
```

| Fixed effect | Estimate | Lower CI [0.025] | Upper CI [0.975] |
| --- | --- | --- | --- |
| Intercept | 0.486 | 0.260 | 0.713 |
| Depart\_breed | -0.134 | -0.284 | 0.017 |
| Nonbreed\_arrival | 0.098 | -0.079 | 0.276 |
| Nonbreed\_depart | 0.222 | 0.055 | 0.389 |
| Seabird | 0.047 | -0.167 | 0.262 |
| Waterbird | 0.017 | -0.104 | 0.138 |
| GLS | 0.036 | -0.133 | 0.206 |
| Satellite | 0.040 | -0.156 | 0.236 |
| k | -0.039 | -0.208 | 0.131 |
| Female | -0.060 | -0.279 | 0.158 |
| Male | -0.004 | -0.030 | 0.023 |

## Publication Bias Analysis

We followed a recently proposed method by Nakagawa, Lagisz, Jennions, et al. (2021), which involved conducting 3 publication bias models: 1) Meta-regression with SE (uni-moderator), 2) meta-regression with year of publication (uni-moderator), and 3) all-in publication bias test (multi-moderator). We ran these publication bias models using the dataset that had negative repeatability values set to zero.

### Meta-regression with SE (uni-moderator)

To test for publication bias, we first fit a phylogenetic multilevel meta-regression to explore whether there is some evidence of small-study effects in our meta-analytic dataset. To do so, we fit a uni-moderator phylogenetic multilevel meta-regression including the effect sizes’ standard errors (sei) as the only moderator.

```
# creating a variable for the standard error of each effect size (i.e. the square root of the sampling variance)
df$sei <- sqrt(df$VZr)

# Application of Equation 21 from the main text in Nakagawa et al. 2021
publication.bias.model.r.se <- rma.mv(yi = Zr2, V = VCV,
                                      mod = ~1 + sei,
                                      random = list(~1 | es_ID, 
                                                    ~1 | paper_ID, 
                                                    ~1 | cohort_ID, 
                                                    ~1 | species_ID, 
                                                    ~1 | phylogeny),
                                      R = list(phylogeny = varcor), # added in phylogney
                                      data=df)

# print(publication.bias.model.r.se,digits=3)
```

#### Table S20

Regression coefficients (Estimate) and 95% confidence intervals (CIs) from the univariate meta-regression fitted with `sei`.

```
# creating a table
t_sei <- tibble(`Fixed effect` = c("Intercept", "sei"), Estimate = c(publication.bias.model.r.se$b),
    `Lower CI [0.025]` = c(publication.bias.model.r.se$ci.lb), `Upper CI  [0.975]` = c(publication.bias.model.r.se$ci.ub))
# `R2` = c(orchaRd::r2_ml(publication.bias.model.r.se)[[1]], rep(NA,1)))

t_sei %>%
    kable("html", digits = 3) %>%
    kable_styling("striped", position = "left")
```

| Fixed effect | Estimate | Lower CI [0.025] | Upper CI [0.975] |
| --- | --- | --- | --- |
| Intercept | 0.482 | 0.303 | 0.662 |
| sei | 0.213 | -0.326 | 0.752 |

According to this uni-moderator meta-regression, there is no evidence of small-study effects since the slope of the moderator ‘sei’ is not statistically significant (slope = 0.213, 95% CI = [-0.326, 0.752]; *R2marginal* = 2.1%), showing that effect sizes with larger SE (more uncertain effect sizes) do not tend to be larger. But we will confirm this after accounting for some of the heterogeneity present in the data using the all-in publication bias test (multi-moderator meta-regression).

### Meta-regression with year of publication (uni-moderator)

To test for time-lag bias (also called decline effects) we can first fit a uni-moderator phylogenetic multilevel meta-regression including the year of publication (mean-centred) as the only moderator.

```
df$pub_year.c <- as.vector(scale(df$pub_year, scale = F))

# Application of Equation 23 from the main text in Nakagawa et al. 2021
publication.bias.model.r.timelag <- rma.mv(yi = Zr2, V = VCV,
                                           mods= ~1 + pub_year.c,
                                           random = list(~1 | es_ID, 
                                                         ~1 | paper_ID, 
                                                         ~1 | cohort_ID, 
                                                         ~1 | species_ID, 
                                                         ~1 | phylogeny),
                                           R = list(phylogeny = varcor), # added in phylogney
                                           data=df)
```

#### Table S21

Regression coefficients (Estimate) and 95% confidence intervals (CIs) from the univariate meta-regression fitted with publication year.

```
# creating a table
t_pub_year <- tibble(`Fixed effect` = c("Intercept", "pub_year.c"), Estimate = c(publication.bias.model.r.timelag$b),
    `Lower CI [0.025]` = c(publication.bias.model.r.timelag$ci.lb), `Upper CI  [0.975]` = c(publication.bias.model.r.timelag$ci.ub))
# `R2` = c(orchaRd::r2_ml(publication.bias.model.r.timelag)[[1]], rep(NA,1)))

t_pub_year %>%
    kable("html", digits = 3) %>%
    kable_styling("striped", position = "left")
```

| Fixed effect | Estimate | Lower CI [0.025] | Upper CI [0.975] |
| --- | --- | --- | --- |
| Intercept | 0.550 | 0.428 | 0.673 |
| pub\_year.c | 0.008 | -0.002 | 0.019 |

According to this uni-moderator meta-regression, there is no decline effects since the slope of the moderator ‘year of publication’ is essentially zero (slope = 0.008, 95% CI = [-0.002, 0.019]; *R2marginal* = 2.5%), showing that effect sizes have not changed linearly over time since the first effect size was published. But again, we need to confirm this pattern after accounting for some of the heterogeneity present in the data using the all-in publication bias test.

### All-in publication bias test (multi-moderator)

When heterogeneity exists (which is normally the case in ecology and evolution; Senior et al. 2016), it is best to combine the above two models with other moderators since those additional moderators will generally be expected to explain some of the heterogeneity. That is, this all-in publication bias test (multi-moderator meta-regression) would be the best test of small-study (publication bias) and decline effects (time-lag bias) in most meta-analytic datasets (see Nakagawa, Lagisz, Jennions, et al., 2021). For our data, we will run a multi-moderator phylogenetic multilevel meta-regression including the effect sizes’ standard errors, the year of publication (mean-centred) and the 5 moderators included in previous models.

```
publication.bias.model.r.all.se <- rma.mv(yi = Zr2, V = VCV,
                                         mods= ~1 + # -1 removes the intercept
                                           sei +
                                           pub_year.c +
                                         annual_event + method + taxa + sex + k, 
                                         random = list(~1 | es_ID, 
                                                       ~1 | paper_ID, 
                                                       ~1 | cohort_ID, 
                                                       ~1 | species_ID, 
                                                       ~1 | phylogeny),
                                         R = list(phylogeny = varcor), # added in phylogney
                                         data=df)
```

#### Table S22

Regression coefficients (Estimate) and 95% confidence intervals (CIs) from the multivariate meta-regression with sei, publication year, and the five moderator variables. *R2marginal* = 20.8%.

```
# creating a table
t_all_in <- tibble(`Fixed effect` = c("Intercept", "sei", "pub_year.c", "Depart_breed",
    "Nonbreed_arrival", "Nonbreed_depart", "GLS", "Satellite", "Seabird", "Waterbird",
    "Female", "Male", "k"), Estimate = c(publication.bias.model.r.all.se$b), `Lower CI [0.025]` = c(publication.bias.model.r.all.se$ci.lb),
    `Upper CI  [0.975]` = c(publication.bias.model.r.all.se$ci.ub))
# `R2` = c(r2_publication.bias.model.r.all.se[1], rep(NA,12)))

t_all_in %>%
    kable("html", digits = 3) %>%
    kable_styling("striped", position = "left")
```

| Fixed effect | Estimate | Lower CI [0.025] | Upper CI [0.975] |
| --- | --- | --- | --- |
| Intercept | 0.435 | 0.136 | 0.734 |
| sei | 0.176 | -0.459 | 0.811 |
| pub\_year.c | 0.009 | -0.004 | 0.023 |
| Depart\_breed | -0.113 | -0.266 | 0.040 |
| Nonbreed\_arrival | 0.100 | -0.082 | 0.282 |
| Nonbreed\_depart | 0.237 | 0.067 | 0.408 |
| GLS | -0.012 | -0.299 | 0.275 |
| Satellite | -0.023 | -0.369 | 0.323 |
| Seabird | 0.191 | -0.066 | 0.448 |
| Waterbird | 0.108 | -0.137 | 0.352 |
| Female | -0.072 | -0.307 | 0.163 |
| Male | -0.094 | -0.325 | 0.137 |
| k | -0.006 | -0.061 | 0.050 |

The all-in publication bias test agrees with what we observed in the uni-moderator meta-regressions above. First, the multi-moderator meta-regression shows no significant slope for the moderator ‘sei’ (slope = 0.176, 95% CI = [-0.459,0.811]; Fig. S5), showing no evidence of small-study effects. In other words, the largest effect sizes in the dataset do not tend to be those with the lowest precision (i.e. larger uncertainty). Second, the all-in publication bias test also confirms that there is no evidence of decline effects in the data since the slope of the moderator ‘year of publication’ was again indistinguishable from zero (slope = 0.009, 95% CI = [-0.004,0.023]; Fig. S6).

#### Figure S5

```
predict.publication.bias.model.r.all.se.plot.1 <- predict(publication.bias.model.r.all.se,
    newmods = cbind(seq(min(df$sei), max(df$sei), 0.005), c(0), 0, 0, 0, 0, 0, 0,
        0, 0, 0, 0))

newdat <- data.frame(sei = seq(min(df$sei), max(df$sei), 0.005), fit = predict.publication.bias.model.r.all.se.plot.1$pred,
    upper = predict.publication.bias.model.r.all.se.plot.1$ci.ub, lower = predict.publication.bias.model.r.all.se.plot.1$ci.lb,
    stringsAsFactors = FALSE)

ggplot(data = df, aes(x = sei, y = Zr2)) + geom_point(shape = 21, fill = "grey85",
    colour = "grey60", size = 3, alpha = 0.5) + geom_hline(yintercept = 0, linetype = 2,
    colour = "black", alpha = 0.5) + geom_line(data = newdat, aes(x = sei, y = fit),
    size = 1.5, colour = "darkorchid4") + geom_ribbon(data = newdat, aes(ymin = lower,
    ymax = upper, y = 0), alpha = 0.3, fill = "darkorchid4") + labs(x = "Standard error (sei)",
    y = "Effect size (Zr)") + scale_y_continuous(limits = c(-1, 2.5), breaks = seq(-1,
    2.2, by = 1)) + theme_bw() + theme(text = element_text(size = 9, colour = "black",
    hjust = 0.5), panel.grid = element_blank())
```

**Figure S5.** A bubble plot showing that effect sizes with larger standard errors do not tend to be larger, providing no evidence of small-study effects in the meta-analytic dataset. The solid line represents the model estimate and the shading shows its 95% confidence intervals.

#### Figure S6

```
predict.publication.bias.model.r.all.se.plot.2 <- predict(publication.bias.model.r.all.se,
    newmods = cbind(mean(df$sei), seq(min(df$pub_year.c), max(df$pub_year.c), 0.25),
        0, 0, 0, 0, 0, 0, 0, 0, 0, 0))


newdat2 <- data.frame(pub_year.c = seq(min(df$pub_year.c), max(df$pub_year.c), 0.25),
    fit = predict.publication.bias.model.r.all.se.plot.2$pred, upper = predict.publication.bias.model.r.all.se.plot.2$ci.ub,
    lower = predict.publication.bias.model.r.all.se.plot.2$ci.lb, stringsAsFactors = FALSE)

ggplot(data = df, aes(x = pub_year.c, y = Zr2)) + geom_point(aes(size = (1/sqrt(VZr))),
    shape = 21, fill = "grey85", colour = "grey60", alpha = 0.5) + geom_hline(yintercept = 0,
    linetype = 2, colour = "black", alpha = 0.5) + geom_line(data = newdat2, aes(x = pub_year.c,
    y = fit), size = 1.5, colour = "darkorchid4") + geom_ribbon(data = newdat2, aes(ymin = lower,
    ymax = upper, y = 0), alpha = 0.3, fill = "darkorchid4") + labs(x = "Year of publication",
    y = "Effect size (Zr)", size = "Precison (1/SE)") + scale_y_continuous(limits = c(-1,
    2.5), breaks = seq(-1, 2.2, by = 1)) + scale_x_continuous(breaks = c(-24.0862069,
    -14.0862069, -4.0862069, 5.9137931), label = c(1990, 2000, 2010, 2020)) + theme_bw() +
    theme(text = element_text(size = 9, colour = "black", hjust = 0.5), panel.grid = element_blank(),
        legend.text = element_text(size = 8), legend.position = c(0, 0), legend.justification = c(0,
            0), legend.background = element_blank(), legend.direction = "horizontal",
        legend.title = element_text(size = 8))
```

**Figure S6.** A bubble plot showing that the overall effect size has not changed over time, where the solid line represents the model estimate and the shading shows its 95% confidence intervals, with individual data points scaled by precision (1/SE).

## Acknowledgements

Many coding materials have been borrowed from these papers (Hayward et al., 2021; Pottier et al., 2021).

## R Session Information

**R version 3.6.2 (2019-12-12)**

**Platform:** x86\_64-w64-mingw32/x64 (64-bit)

**locale:** *LC\_COLLATE=English\_United Kingdom.1252*, *LC\_CTYPE=English\_United Kingdom.1252*, *LC\_MONETARY=English\_United Kingdom.1252*, *LC\_NUMERIC=C* and *LC\_TIME=English\_United Kingdom.1252*

**attached base packages:** *stats*, *graphics*, *grDevices*, *utils*, *datasets*, *methods* and *base*

**other attached packages:** *pander(v.0.6.4)*, *png(v.0.1-7)*, *patchwork(v.1.1.1)*, *kableExtra(v.1.3.4)*, *MuMIn(v.1.43.17)*, *orchaRd(v.0.0.0.9000)*, *clubSandwich(v.0.5.3)*, *here(v.1.0.1)*, *cowplot(v.1.1.1)*, *ape(v.5.5)*, *rotl(v.3.0.11)*, *metafor(v.3.0-2)*, *Matrix(v.1.2-18)*, *forcats(v.0.5.1)*, *stringr(v.1.4.0)*, *dplyr(v.1.0.7)*, *purrr(v.0.3.2)*, *readr(v.2.0.0)*, *tidyr(v.1.1.3)*, *tibble(v.3.1.2)*, *ggplot2(v.3.3.5)*, *tidyverse(v.1.3.1)* and *SciViews(v.0.9-13.1)*

**loaded via a namespace (and not attached):** *nlme(v.3.1-142)*, *fs(v.1.5.0)*, *lubridate(v.1.7.10)*, *webshot(v.0.5.2)*, *progress(v.1.2.2)*, *httr(v.1.4.2)*, *rprojroot(v.2.0.2)*, *tools(v.3.6.2)*, *backports(v.1.2.1)*, *bslib(v.0.2.5.1)*, *utf8(v.1.2.1)*, *R6(v.2.5.0)*, *vipor(v.0.4.5)*, *DBI(v.1.1.1)*, *colorspace(v.2.0-2)*, *withr(v.2.4.2)*, *tidyselect(v.1.1.1)*, *prettyunits(v.1.1.1)*, *compiler(v.3.6.2)*, *cli(v.3.0.0)*, *rvest(v.1.0.0)*, *formatR(v.1.11)*, *xml2(v.1.3.2)*, *sandwich(v.3.0-1)*, *labeling(v.0.4.2)*, *sass(v.0.4.0)*, *scales(v.1.1.1)*, *systemfonts(v.1.0.2)*, *digest(v.0.6.27)*, *rmarkdown(v.2.9)*, *svglite(v.2.0.0)*, *rentrez(v.1.2.3)*, *pkgconfig(v.2.0.3)*, *htmltools(v.0.5.1.1)*, *highr(v.0.9)*, *dbplyr(v.2.1.1)*, *rlang(v.0.4.11)*, *readxl(v.1.3.1)*, *rstudioapi(v.0.13)*, *farver(v.2.1.0)*, *jquerylib(v.0.1.4)*, *generics(v.0.1.0)*, *zoo(v.1.8-9)*, *jsonlite(v.1.7.2)*, *magrittr(v.2.0.1)*, *ggbeeswarm(v.0.6.0)*, *Rcpp(v.1.0.7)*, *munsell(v.0.5.0)*, *fansi(v.0.5.0)*, *lifecycle(v.1.0.0)*, *stringi(v.1.7.3)*, *yaml(v.2.2.1)*, *mathjaxr(v.1.4-0)*, *grid(v.3.6.2)*, *parallel(v.3.6.2)*, *crayon(v.1.4.1)*, *rncl(v.0.8.4)*, *lattice(v.0.20-40)*, *haven(v.2.4.1)*, *hms(v.1.1.0)*, *knitr(v.1.33)*, *pillar(v.1.6.4)*, *codetools(v.0.2-18)*, *stats4(v.3.6.2)*, *reprex(v.2.0.0)*, *XML(v.3.99-0.3)*, *glue(v.1.4.2)*, *evaluate(v.0.14)*, *modelr(v.0.1.8)*, *vctrs(v.0.3.8)*, *tzdb(v.0.1.1)*, *cellranger(v.1.1.0)*, *gtable(v.0.3.0)*, *assertthat(v.0.2.1)*, *xfun(v.0.24)*, *broom(v.0.7.8)*, *viridisLite(v.0.4.0)*, *beeswarm(v.0.4.0)*, *ellipse(v.0.4.2)* and *ellipsis(v.0.3.2)*

## References

Chamberlain, S. A., Dibble, C. J., Rasmussen, N. L., Allen, V., Maitner, B. S., Ahern, J. R., Roy, C. L., Meza-Lopez, M., Carrillo, J., Siemann, E., Lajeunesse, M. J., & Whitney, K. D. (2012). *Does phylogeny matter? Assessing the impact of phylogenetic information in ecological meta‐analysis*. 10.

Geen, G. R., Robinson, R. A., & Baillie, S. R. (2019). Effects of tracking devices on individual birds - a review of the evidence. *Journal of Avian Biology*, *50*(2). https://doi.org/10.1111/jav.01823

Hackett, S. J., Kimball, R. T., Reddy, S., Bowie, R. C. K., Braun, E. L., Braun, M. J., Chojnowski, J. L., Cox, W. A., Han, K.-L., Harshman, J., Huddleston, C. J., Marks, B. D., Miglia, K. J., Moore, W. S., Sheldon, F. H., Steadman, D. W., Witt, C. C., & Yuri, T. (2008). A Phylogenomic Study of Birds Reveals Their Evolutionary History. *Science*, *320*(5884), 1763–1768. https://doi.org/10.1126/science.1157704

Hayward, A., Poulin, R., & Nakagawa, S. (2021). A broadscale analysis of host‐symbiont cophylogeny reveals the drivers of phylogenetic congruence. *Ecology Letters*, *24*(8), 1681–1696. https://doi.org/10.1111/ele.13757

Holtmann, B., Lagisz, M., & Nakagawa, S. (2017). Metabolic rates, and not hormone levels, are a likely mediator of between‐individual differences in behaviour: A meta‐analysis. *Functional Ecology*, *31*(3), 685–696. https://doi.org/10.1111/1365-2435.12779

Jetz, W., Thomas, G. H., Joy, J. B., Hartmann, K., & Mooers, A. O. (2012). The global diversity of birds in space and time. *Nature*, *491*(7424), 444–448. https://doi.org/10.1038/nature11631

Nakagawa, S., Lagisz, M., Jennions, M. D., Koricheva, J., Noble, D. W. A., Parker, T. H., Sánchez‐Tójar, A., Yang, Y., & O’Dea, R. E. (2021). Methods for testing publication bias in ecological and evolutionary meta‐analyses. *Methods in Ecology and Evolution*, 2041–210X.13724. https://doi.org/10.1111/2041-210X.13724

Nakagawa, S., Lagisz, M., O’Dea, R. E., Rutkowska, J., Yang, Y., Noble, D. W. A., & Senior, A. M. (2021). The orchard plot: Cultivating a forest plot for use in ecology, evolution, and beyond. *Research Synthesis Methods*, *12*(1), 4–12. https://doi.org/10.1002/jrsm.1424

Nakagawa, S., & Schielzeth, H. (2010). Repeatability for Gaussian and non-Gaussian data: A practical guide for biologists. *Biological Reviews*, no–no. https://doi.org/10.1111/j.1469-185X.2010.00141.x

Noble, D. W. A., Lagisz, M., O’dea, R. E., & Nakagawa, S. (2017). Nonindependence and sensitivity analyses in ecological and evolutionary meta‐analyses. *Molecular Ecology*, *26*(9), 2410–2425. https://doi.org/10.1111/mec.14031

O’Dea, R. E., Lagisz, M., Jennions, M. D., Koricheva, J., Noble, D. W. A., Parker, T. H., Gurevitch, J., Page, M. J., Stewart, G., Moher, D., & Nakagawa, S. (2021). Preferred reporting items for systematic reviews and meta‐analyses in ecology and evolutionary biology: A PRISMA extension. *Biological Reviews*, *96*(5), 1695–1722. https://doi.org/10.1111/brv.12721

Paradis, E., & Schliep, K. (2019). Ape 5.0: An environment for modern phylogenetics and evolutionary analyses in R. *Bioinformatics*, *35*(3), 526–528. https://doi.org/10.1093/bioinformatics/bty633

Pottier, P., Burke, S., Drobniak, S. M., Lagisz, M., & Nakagawa, S. (2021). Sexual (in)equality? A meta‐analysis of sex differences in thermal acclimation capacity across ectotherms. *Functional Ecology*, 1365–2435.13899. https://doi.org/10.1111/1365-2435.13899

Viechtbauer, W. (2010). Conducting Meta-Analyses in R with the metafor Package. *Journal of Statistical Software*, *36*(3). https://doi.org/10.18637/jss.v036.i03

LS0tDQp0aXRsZTogJ0luZGl2aWR1YWwgcmVwZWF0YWJpbGl0eSBvZiBhdmlhbiBtaWdyYXRpb24gcGhlbm9sb2d5OiBhIHN5c3RlbWF0aWMgcmV2aWV3DQogIGFuZCBtZXRhLWFuYWx5c2lzJw0KYXV0aG9yOiBLaXJzdHkgQS4gRnJhbmtsaW4qLCBNYWxjb2xtIEEuIEMuIE5pY29sbCwgU2ltb24gSi4gQnV0bGVyLCBLZW4gTm9ycmlzLCBOb3JtYW4gUmF0Y2xpZmZlLA0KICBTaGluaWNoaSBOYWthZ2F3YSAmIEplbm5pZmVyIEEuIEdpbGwNCmRhdGU6ICJgciBmb3JtYXQoU3lzLnRpbWUoKSwgJyVkICVCICVZJylgIg0Kb3V0cHV0Og0KICBodG1sX2RvY3VtZW50Og0KICAgIGNvZGVfZG93bmxvYWQ6IHllcw0KICAgIGNvZGVfZm9sZGluZzogaGlkZQ0KICAgIGRlcHRoOiA0DQogICAgbnVtYmVyX3NlY3Rpb25zOiBubw0KICAgIHRoZW1lOiBjb3Ntbw0KICAgIHRvYzogeWVzDQogICAgdG9jX2RlcHRoOiA0DQogICAgdG9jX2Zsb2F0OiB5ZXMNCiAgcGRmX2RvY3VtZW50Og0KICAgIHRvYzogeWVzDQogIHdvcmRfZG9jdW1lbnQ6DQogICAgdG9jOiB5ZXMNCiAgICB0b2NfZGVwdGg6ICc0Jw0KbGluay1jaXRhdGlvbnM6IHllcw0KY3NsOiBqb3VybmFsLW9mLWFuaW1hbC1lY29sb2d5LmNzbA0Kc3VidGl0bGU6IEFwcGVuZGl4IFMxDQpiaWJsaW9ncmFwaHk6IE1ldGEtYW5hbHlzaXNfc3VwcF9yZWZlcmVuY2VzLmJpYg0KYWx3YXlzX2FsbG93X2h0bWw6IHRydWUNCi0tLQ0KDQpgYGB7ciBzZXR1cCwgaW5jbHVkZSA9IEZBTFNFfQ0KI2tuaXRlciBzZXR0aW5nDQprbml0cjo6b3B0c19jaHVuayRzZXQoDQptZXNzYWdlID0gRkFMU0UsDQp3YXJuaW5nID0gRkFMU0UsICMgbm8gd2FybmluZ3MNCmNhY2hlID0gVFJVRSwjIGNhY2hlaW5nIHRvIHNhdmUgdGltZSB3aGVuIGtuaXRpbmcNCnRpZHkgPSBUUlVFDQojZmlnLndpZHRoID0gOQ0KKQ0KDQojIGNsZWFuaW5nIHVwDQogcm0obGlzdD1scygpKQ0KYGBgDQoNCiMjIFNldHVwcw0KDQojIyMgTG9hZGluZyBwYWNrYWdlcyBhbmQgY3VzdG9tIGZ1bmN0aW9ucw0KDQpgYGB7ciBsb2FkLXBhY2thZ2VzfQ0KIyBsb2FkaW5nIHBhY2thZ2VzDQojZGV2dG9vbHM6Omluc3RhbGxfZ2l0aHViKCJpdGNoeXNoaW4vb3JjaGFyZF9wbG90Iiwgc3ViZGlyID0gIm9yY2hhUmQiLCBmb3JjZSA9IFRSVUUsIGJ1aWxkX3ZpZ25ldHRlcyA9IFRSVUUpDQpwYWNtYW46OnBfbG9hZChTY2lWaWV3cywNCiAgICAgICAgICAgICAgIHRpZHl2ZXJzZSwNCiAgICAgICAgICAgICAgIGRwbHlyLA0KICAgICAgICAgICAgICAgbWV0YWZvciwgIyBwYWNrYWdlIGZvciBtZXRhLWFuYWx5c2lzDQogICAgICAgICAgICAgICByb3RsLCAjIHBhY2thZ2UgZm9yIHBoeWxvZ2VueQ0KICAgICAgICAgICAgICAgYXBlLCAjIHBhY2thZ2UgZm9yIHBoeWxvZ2VueQ0KICAgICAgICAgICAgICAgY293cGxvdCwgIyBjb21iaW5pbmcgbXVsdGlwbGUgcGxvdHMNCiAgICAgICAgICAgICAgIGhlcmUsICMgbWFraW5nIGZvbGRlciBwYXRoIHVzYWJsZSBmb3IgYWxsDQogICAgICAgICAgICAgICBjbHViU2FuZHdpY2gsICMgcGFja2FnZSB0byBhc3Npc3QgbWV0YWZvcg0KICAgICAgICAgICAgICAgb3JjaGFSZCwgIyBwbG90dGluZyBvcmNoYVJkIHBsb3RzDQogICAgICAgICAgICAgICBNdU1JbiwgIyBtdWx0aS1tb2RlbCBpbmZlcmVuY2UNCiAgICAgICAgICAgICAgIGthYmxlRXh0cmEsICMgbWFraW5nIG5pY2UgdGFibGVzDQogICAgICAgICAgICAgICBwYXRjaHdvcmssICMgcHV0dGluZyBnZ3Bsb3RzIHRvZ2V0aGVyDQogICAgICAgICAgICAgICBwbmcsICMgcmVhZGluZyBwbmcgZmlsZXMNCiAgICAgICAgICAgICAgIGdyaWQsICMgZ3JhcGhpYyBsYXlvdXQgbWFuaXB1bGF0aW9uDQogICAgICAgICAgICAgICBwYW5kZXIsICMgbmljZSB0YWJsZXMNCiAgICAgICAgICAgICAgIGdncGxvdDIgIyBtYWtpbmcgZmlndXJlcw0KKQ0KYGBgDQoNCiMjIyMgQ3VzdG9tIGZ1bmN0aW9ucw0KDQpXZSBoYXZlIDQgY3VzdG9tIGZ1bmN0aW9ucyBuYW1lZCA6IGBacl90cmFuc2Zvcm1hdGlvbigpYCwgYENhbGNfU1YoKWAsIGBacl90b19JQ0MoKWAsIGFuZCBgY29udF9nZW4oKWAsIGFsbCBvZiB3aGljaCBhcmUgdXNlZCBsYXRlciAoc2VlIGJlbG93IGZvciB0aGVpciBmdW5jdGlvbmFsaXR5KSBhbmQgdGhlIGNvZGUgaXMgaW5jbHVkZWQgaGVyZS4gDQoNCmBgYHtyIGN1c3RvbS1mdW5jdGlvbnN9DQojIEN1c3RvbSBmdW5jdGlvbnMNCg0KIyBHZXR0aW5nIFpyIGFuZCBpdHMgc2FtcGxpbmcgdmFyaWFuY2UgZnJvbSByZXBlYXRhYmlsaXR5IHZhbHVlcyBhbmQgc2FtcGxlIHNpemUgaW5mb3JtYXRpb24NCiMgRnVuY3Rpb24gZm9yIEZpc2hlcidzIFogdHJhbnNmb3JtYXRpb24gKFpyKSBmb3IgY29ycmVsYXRpb24tYmFzZWQgcmVwZWF0YWJpbGl0aWVzIChyKSBhbmQgSUNDIChIb2x0bWFubiBldCBhbC4gMjAxNywgVGFibGUgMSkNClpyX3RyYW5zZm9ybWF0aW9uIDwtIGZ1bmN0aW9uKHIsSyxFc3QpIHsNCiAgDQogIGlmKEVzdCA9PSAiSUNDIikge1pyIDwtIDAuNSpsbigoKDErKEstMSkqcikvKDEtcikpKX0NCiAgaWYoRXN0ID09ICJyIikge1pyIDwtIDAuNSpsbigoKDErcikvKDEtcikpKX0NCiAgWnINCn0NCg0KIyBGdW5jdGlvbiBmb3Igc2FtcGxpbmcgdmFyaWFuY2UgZm9yIGNvcnJlbGF0aW9uLWJhc2VkIHJlcGVhdGFiaWxpdGllcyAocikgYW5kIElDQyAoSG9sdG1hbm4gZXQgYWwuIDIwMTcsIFRhYmxlIDEpDQpDYWxjX1NWIDwtIGZ1bmN0aW9uKEssTixFc3Qpew0KICANCiAgaWYoRXN0ID09ICJJQ0MiKSB7VlpyIDwtIEsvKDIqKChOLTIpKihLLTEpKSl9DQogIGlmKEVzdCA9PSAiciIpIHtWWnIgPC0gMS8oTi0zKX0NCiAgVlpyDQp9DQoNCiMgRnVuY3Rpb24gZm9yIGJhY2stdHJhbnNmb3JtaW5nIFpyIHRvIElDQw0KWnJfdG9fSUNDIDwtIGZ1bmN0aW9uKHgsayl7IA0KICAoZXhwKDIqeCktMSkvKGV4cCgyKngpK2stMSkNCn0NCg0KIyBDb250cmFzdCBuYW1lIGdlbmVyYXRvciBmb3IgdGliYmxlIHRhYmxlcyAoZnJvbSBIYXl3YXJkIGV0IGFsLiAyMDIxKQ0KY29udF9nZW4gPC0gZnVuY3Rpb24gKG5hbWUpIHsNCiAgY29tYmluYXRpb24gPC0gY29tYm4obmFtZSwyKQ0KICBuYW1lX2RhdCA8LSB0KGNvbWJpbmF0aW9uKQ0KICBuYW1lcyA8LSBwYXN0ZShuYW1lX2RhdFsgLDFdLCBuYW1lX2RhdFssIDJdLCBzZXAgPSAiLSIpDQogIHJldHVybihuYW1lcykNCn0NCg0KYGBgDQoNCiMjIFN1cHBsZW1lbnRhcnkgTWV0aG9kcw0KDQojIyMgU3VwcGxlbWVudGFyeSBpbmZvcm1hdGlvbiBmb3IgdGhlIGxpdGVyYXR1cmUgc2VhcmNoDQoNCldlIGFpbWVkIHRvIGNvbmR1Y3QgYSBjb21wcmVoZW5zaXZlIHNlYXJjaCBmb3Igc3R1ZGllcyBlc3RpbWF0aW5nIHJlcGVhdGFiaWxpdHkgb2YgdGVtcG9yYWwgcGFyYW1ldGVycyBvZiBhdmlhbiBtaWdyYXRpb24gdXNpbmcgYSBjb21iaW5hdGlvbiBvZiBhcHByb2FjaGVzLiBXZSBmb2N1c2VkIG9uIGFycml2YWwgYXQsIGFuZCBkZXBhcnR1cmUgZnJvbSwgYnJlZWRpbmcgYW5kIG5vbi1icmVlZGluZyBncm91bmRzLiBGaXJzdCwgd2UgcGVyZm9ybWVkIGEgc3lzdGVtYXRpYyBzZWFyY2ggZm9yIHB1Ymxpc2hlZCBzdHVkaWVzIHVzaW5nIHRoZSBXZWIgb2YgU2NpZW5jZSBhbmQgU2NvcHVzIG9ubGluZSBkYXRhYmFzZXMgb24gMXN0IEp1bmUgMjAyMSAoVGltZXNwYW46IGFsbCB5ZWFycykuIFNlY29uZCwgd2UgY29uc3VsdGVkIGEgcmVjZW50bHkgcHVibGlzaGVkIG1ldGEtYW5hbHlzaXMgb2YgaG9ybW9uYWwsIG1ldGFib2xpYyBhbmQgYmVoYXZpb3VyYWwgcmVwZWF0YWJpbGl0eSBpbiBiaXJkcyBbQGhvbHRtYW5uX21ldGFib2xpY18yMDE3XSwgd2hpY2ggaW5jbHVkZWQgcmVwZWF0YWJpbGl0eSBlc3RpbWF0ZXMgb2YgbWlncmF0aW9uLiBXZSBtYW51YWxseSBjaGVja2VkIGVhY2ggZW50cnkgZnJvbSB0aG9zZSBzb3VyY2VzIHRvIGNvbmZpcm0gc3VpdGFiaWxpdHkgZm9yIG91ciBwdXJwb3NlcyBhbmQgZXh0cmFjdGVkIGFkZGl0aW9uYWwgbW9kZXJhdG9yIHZhcmlhYmxlcyB0byBiZSB1c2VkIGluIG91ciBhbmFseXNlcyAoc2VlIGJlbG93KS4gRmluYWxseSwgaW4gb3JkZXIgdG8gYWRkIHRvIOKAkyBhbmQgdmFsaWRhdGUgdGhlIGFjY3VyYWN5IG9mIOKAkyB0aGUgcmVzdWx0cyBvZiB0aGUgbGl0ZXJhdHVyZSBzZWFyY2gsIHdlIHNlYXJjaGVkIHRoZSByZWZlcmVuY2UgbGlzdHMgb2YgcGFwZXJzIGFscmVhZHkgaW4gb3VyIGFjY2VwdGVkIHJlZmVyZW5jZSBsaWJyYXJ5LiBUaGUgZGV0YWlscyBvZiB0aGVzZSBzZWFyY2ggc3RyYXRlZ2llcyBhbmQgdGhlIEJvb2xlYW4gc2VhcmNoIHN0cmluZ3MgdXNlZCBhcmUgcHJlc2VudGVkIGJlbG93LCBhbG9uZyB3aXRoIGEgZmxvdyBkaWFncmFtIChvZnRlbiByZWZlcnJlZCB0byBhcyBhIFBSSVNNQSBmbG93IGNoYXJ0IOKAkyB0aGUgUHJlZmVycmVkIFJlcG9ydGluZyBJdGVtcyBpbiBTeXN0ZW1hdGljIFJldmlld3MgYW5kIE1ldGEtQW5hbHlzZXM7IEZpZ3VyZSBTMSkgd2hpY2ggc2hvd3MgdGhlIHN0YWdlcyBhdCB3aGljaCBzdHVkaWVzIHdlcmUgZGlzcXVhbGlmaWVkIG9yIGV2ZW50dWFsbHkgdXNlZCBpbiB0aGUgY3VycmVudCBzdHVkeS4NCiAgDQogICoqV2ViIG9mIFNjaWVuY2UgQ29yZSBDb2xsZWN0aW9uOioqDQogIA0KICAoVFM9KOKAnHJlcGVhdFwq4oCdIE9SICJpbnRyYWNsYXNzIGNvcnJlbGF0aW9uIiBPUiDigJxJQ0PigJ0gT1IgImluZGl2aWR1YWwgdmFyaWF0aW9uIiBPUiAiaW50cmEtaW5kaXZpZHVhbCB2YXJpYXRpb24iIE9SICJiZXR3ZWVuLWluZGl2aWR1YWwgdmFyaWF0aW9uIiBPUiAiY29uc2lzdGVuXCoiIE9SICJmbGV4aWJcKiIpIEFORCBUUz0o4oCcbWlncmF0aW9u4oCdIE9SIOKAnG1pZ3JhdG9yeeKAnSkgQU5EIFRTPSjigJxcKmJpcmRcKuKAnSBPUiDigJxhdmVz4oCdIE9SIOKAnGF2aWFu4oCdKSkgQU5EIChTVT0oQmVoYXZpb3JhbCBTY2llbmNlcyBPUiBCaW9kaXZlcnNpdHkgJiBDb25zZXJ2YXRpb24gT1IgRW52aXJvbm1lbnRhbCBTY2llbmNlcyAmIEVjb2xvZ3kgT1IgRXZvbHV0aW9uYXJ5IEJpb2xvZ3kgT1IgR2VuZXRpY3MgJiBIZXJlZGl0eSBPUiBNYXJpbmUgJiBGcmVzaHdhdGVyIEJpb2xvZ3kgT1IgT2NlYW5vZ3JhcGh5IE9SIFZldGVyaW5hcnkgU2NpZW5jZXMgT1IgWm9vbG9neSkpDQogIA0KICAqKlNjb3B1czoqKiANCiAgDQogIFRJVExFLUFCUy1LRVkgKCAicmVwZWF0XCoiIE9SICJpbnRyYWNsYXNzIGNvcnJlbGF0aW9uIiBPUiAiSUNDIiBPUiAiaW5kaXZpZHVhbCB2YXJpYXRpb24iIE9SICJpbnRyYS1pbmRpdmlkdWFsIHZhcmlhdGlvbiIgT1IgImJldHdlZW4taW5kaXZpZHVhbCB2YXJpYXRpb24iIE9SICJjb25zaXN0ZW5cKiIgT1IgImZsZXhpYlwqIiApIEFORCBUSVRMRS1BQlMtS0VZICggIm1pZ3JhdGlvbiIgT1IgIm1pZ3JhdG9yeSIgKSBBTkQgVElUTEUtQUJTLUtFWSAoICJcKmJpcmRcKiIgT1IgImF2ZXMiIE9SICJhdmlhbiIgKSBBTkQgKCBMSU1JVC1UTyAoIFNVQkpBUkVBICwgIkFHUkkiICkgT1IgTElNSVQtVE8gKCBTVUJKQVJFQSAsICJFTlZJIiApIE9SIExJTUlULVRPICggU1VCSkFSRUEgLCAiVkVURSIgKSApDQoNCg0KV2UgZm9sbG93ZWQgcmVwb3J0aW5nIGd1aWRlbGluZXMgb3V0bGluZWQgaW4gdGhlIFBSSVNNQS1FY29Fdm8gY2hlY2tsaXN0IFtAb2RlYV9wcmVmZXJyZWRfMjAyMV0uDQoNCiMjIyBQUklTTUEgZmxvdyBjaGFydA0KDQojIyMjIEZpZ3VyZSBTMQ0KIVtdKEM6L1VzZXJzL3JzdDE4dnp1L09uZURyaXZlIC0gVW5pdmVyc2l0eSBvZiBFYXN0IEFuZ2xpYS9QaEQvTGl0LiByZXZpZXcvTWV0YS1hbmFseXNpcy9Bdmlhbl9taWdyYXRpb25fbWV0YS1hbmFseXNpcy9maWdzL1BSSVNNQV9mbG93X2NoYXJ0LlBORyl7d2lkdGg9ODAlfQ0KDQoqKkZpZ3VyZSBTMS4qKiBQUklTTUEgZmxvdyBjaGFydCBzdW1tYXJpc2luZyBzZWFyY2ggbWV0aG9kcyBhbmQgc2NyZWVuaW5nIGZvciBzdHVkaWVzIGluY2x1ZGVkIGluIGFuYWx5c2VzLCBhbmQgcmVhc29ucyBmb3IgZXhjbHVkaW5nIHN0dWRpZXMuIA0KDQojIyMgRGVjaXNpb24gdHJlZQ0KDQojIyMjIEZpZ3VyZSBTMg0KIVtdKEM6L1VzZXJzL3JzdDE4dnp1L09uZURyaXZlIC0gVW5pdmVyc2l0eSBvZiBFYXN0IEFuZ2xpYS9QaEQvTGl0LiByZXZpZXcvTWV0YS1hbmFseXNpcy9Bdmlhbl9taWdyYXRpb25fbWV0YS1hbmFseXNpcy9maWdzL0RlY2lzaW9uX3RyZWVfYWJzdHJhY3QuUE5HKXt3aWR0aD02MCV9DQoNCioqRmlndXJlIFMyLioqIERlY2lzaW9uIHRyZWUgdXNlZCB0byBldmFsdWF0ZSBzdHVkaWVzIGZvciBpbmNsdXNpb24gYW5kIGV4Y2x1c2lvbiBhdCB0aGUgc3RhZ2Ugb2YgdGl0bGUgYW5kIGFic3RyYWN0IHNjcmVlbmluZy4NCg0KIyMgVGhlIFJlcGVhdGFiaWxpdHkgRGF0YXNldA0KDQojIyMgVGFibGUgb2YgdGhlIGRhdGFzZXQNCg0KQmVsb3cgaXMgdGhlIGRhdGFzZXQgdXNlZCBmb3Igb3VyIG1ldGEtYW5hbHlzaXMsIGZvbGxvd2VkIGJ5IGV4cGxhbmF0aW9ucyBvZiB0aGUgdmFyaWFibGVzIGV4dHJhY3RlZCBmcm9tIHRoZSBwYXBlcnMgaW5jbHVkZWQgKG5vdCBhbGwgdmFyaWFibGVzIHdlcmUgdXNlZCBmb3Igb3VyIGFuYWx5c2VzKS4NCg0KIyMjIyBUYWJsZSBTMQ0KVGhlIG1ldGEtYW5hbHl0aWMgZGF0YXNldCBvZiB0aGlzIHN0dWR5Lg0KDQpgYGB7ciBkYXRhLXNldH0NCiMgZ2V0dGluZyB0aGUgZGF0YSBhbmQgZm9ybWF0aW5nIHNvbWUgdmFyaWFibGVzICh0dXJuaW5nIGNocmFyYWN0ZXIgdmVjdG9ycyB0byBmYWN0b3JzKQ0KZGYgPC0gcmVhZF9jc3YoaGVyZSgiZGF0YSIsICJNZXRhLWFuYWx5c2lzX2RhdGEtMjAyMi5jc3YiKSwgbmEgPSAiTkEiLCBzaG93X2NvbF90eXBlcyA9IEYpICU+JSANCiAgIG11dGF0ZV9pZihpcy5jaGFyYWN0ZXIsIGFzLmZhY3RvcikNCg0KIyBtYWtpbmcgYSBzY3JvbGxhYmxlIHRhYmxlIG9mIGRhdGFzZXQNCmthYmxlKGRmLCAiaHRtbCIpICU+JQ0KICBrYWJsZV9zdHlsaW5nKCJzdHJpcGVkIiwgcG9zaXRpb24gPSAibGVmdCIpICU+JQ0KICBzY3JvbGxfYm94KHdpZHRoID0gIjEwMCUiLCBoZWlnaHQgPSAiNTAwcHgiKQ0KYGBgDQoNCkEuIF9fZXNfSURfXzogVW5pcXVlIElEIGZvciBlYWNoIHJvdyBvZiBkYXRhIChpLmUuIGVhY2ggZWZmZWN0IHNpemUpLg0KDQpCLiBfX3BhcGVyX0lEX186IFVuaXF1ZSBJRCBmb3IgZWFjaCBwYXBlci4NCg0KQy4gX19jb2hvcnRfSURfXzogVW5pcXVlIElEIGZvciBlYWNoIGNvaG9ydCBvZiBiaXJkcy4NCg0KRC4gX19zcGVjaWVzX0lEX186IFVuaXF1ZSBJRCBmb3IgZWFjaCBzcGVjaWVzLg0KDQpFLiBfX3NwZWNpZXNfY29tbW9uX186IENvbW1vbiBuYW1lIG9mIHNwZWNpZXMgKHRha2VuIGZyb20gcGFwZXIpLg0KDQpGLiBfX3NwZWNpZXNfbGF0aW5fXzogTGF0aW4gbmFtZSBvZiBzcGVjaWVzICh0YWtlbiBmcm9tIHBhcGVyKS4NCg0KRy4gX190YXhhX186IFNwZWNpZXMgc3BsaXQgaW50byB0aHJlZSBlY29sb2dpY2FsIGdyb3VwczogJ3dhdGVyYmlyZCcsICdzZWFiaXJkJyBvciAnbGFuZGJpcmQnIGJhc2VkIG9uIEBnZWVuX2VmZmVjdHNfMjAxOQ0KDQpILiBfX3NleF9fOiBNYWxlLCBmZW1hbGUgb3IgYm90aC91bmtub3duLg0KDQpJLiBfX25fXzogTnVtYmVyIG9mIGluZGl2aWR1YWxzLg0KDQpKLiBfX2tfXzogTnVtYmVyIG9mIG9ic2VydmF0aW9ucyBwZXIgaW5kaXZpZHVhbC4NCg0KSy4gX19lc3RfXzogTWV0aG9kIG9mIGNhbGN1bGF0aW5nIHJlcGVhdGFiaWxpdHk6IGNvcnJlbGF0aW9uIGNvZWZmaWNpZW50IChyKSBvciBpbnRyYWNsYXNzIGNvcnJlbGF0aW9uIGNvZWZmaWNpZW50IChJQ0MpLg0KDQpMLiBfX1JfXzogUmVwZWF0YWJpbGl0eSB2YWx1ZS4NCg0KTS4gX19maXhlZF95bl9fOiBBbnkgZml4ZWQgZWZmZWN0cyAob3IgYWRkaXRpb25hbCByYW5kb20gZWZmZWN0cykgaW5jbHVkZWQgaW4gcmVwZWF0YWJpbGl0eSBjYWxjdWxhdGlvbjogeWVzIG9yIG5vLg0KDQpOLiBfX2ZpeGVkX3Zhcl9fOiBJZiB5ZXMsIHdoYXQgYWRkaXRpb25hbCB2YXJpYWJsZXMgYXJlIGluY2x1ZGVkLg0KDQpPLiBfX3Vuc3RhbmRhcmRpemVkX3ZhcmlhbmNlX186IFdoZXRoZXIgdW5zdGFuZGFyZGl6ZWQgdmFyaWFuY2UgY29tcG9uZW50cyBhcmUgcmVwb3J0ZWQuDQoNClAuIF9fbWV0aG9kX186IFRyYWNraW5nIG1ldGhvZDogY29udmVudGlvbmFsIChyaW5naW5nLCBjb2xvdXItcmluZ2luZyksIGdlb2xvY2F0b3IgKGdlb2xvY2F0aW9uKSwgb3IgR1BTIChHUFMsIHNhdGVsbGl0ZSwgUFRUKS4NCg0KUS4gX19hbm51YWxfZXZlbnRfXzogUGVyaW9kIG9mIGFubnVhbCBjeWNsZSB3aGljaCByZXBlYXRhYmlsaXR5IGlzIG1lYXN1cmVkIChhcnJpdmFsIHRvLCBvciBkZXBhcnR1cmUgZnJvbSwgYnJlZWRpbmcgb3Igbm9uLWJyZWVkaW5nIGdyb3VuZHMpLg0KDQpSLiBfX2xvY2F0aW9uX186IExvY2F0aW9uIG9mIHRhZ2dpbmcgKGFzIHdyaXR0ZW4gYnkgcGFwZXIpLg0KDQpTLiBfX2NvbnRpbmVudF9fOiBDb250aW5lbnQgb2YgdGFnZ2luZyBsb2NhdGlvbjogTm9ydGggQW1lcmljYSwgRXVyb3BlLCBvciBPdGhlci4NCg0KVC4gX19sb25nX186IExvbmdpdHVkZSBvZiB0YWdnaW5nIGxvY2F0aW9uLg0KDQpVLiBfX2xhdF9fOiBMYXRpdHVkZSBvZiB0YWdnaW5nIGxvY2F0aW9uLg0KDQpWLiBfX3RhZ19wZXJpb2RfXzogV2hldGhlciBpbmRpdmlkdWFscyB3ZXJlIHRhZ2dlZCBvbiB0aGUgYnJlZWRpbmcgb3Igbm9uLWJyZWVkaW5nIGdyb3VuZHMuDQoNClcuIF9fbm90ZXNfXzogZ2VuZXJhbCBjb21tZW50cw0KDQpYLiBfX2RhdGFfbG9jYXRpb25fXzogV2hlcmUgaW4gdGhlIHBhcGVyIHRoZSBkYXRhIGlzIGxvY2F0ZWQuDQoNClkuIF9fZGF0YV9wcmVzZW50YXRpb25fXzogVGV4dCwgZmlndXJlLCBvciB0YWJsZS4NCg0KWi4gX190aXRsZV9fOiBUaXRsZSBvZiB0aGUgcGFwZXIuDQoNCkFBLiBfX3B1Yl95ZWFyX186IFB1YmxpY2F0aW9uIHllYXIgb2YgcGFwZXIuDQoNCkFCOiBfX2F1dGhvcnNfXzogQXV0aG9ycyBvZiB0aGUgcGFwZXIuDQoNCkFDOiBfX2pvdXJuYWxfXzogSm91cm5hbCB0aGUgcGFwZXIgd2FzIHB1Ymxpc2hlZCBpbi4NCg0KQUQ6IF9fRE9JX186IERPSSBvZiB0aGUgcGFwZXIuDQoNCkFFOiBfX2Z1bGx0ZXh0X0lEX186IEFuIElEIHRoYXQgaXMgdXNlZCB0byBsaW5rIHRoZSBwYXBlciBmb3IgZGF0YSBleHRyYWN0aW9uIHRvIHRoZSByZWNvcmQgb2Ygc2NyZWVuZWQgZnVsbCB0ZXh0cy4NCg0KIyMjIFNhbXBsZSBzaXplcw0KDQojIyMjIFRhYmxlIFMyDQpTYW1wbGUgc2l6ZXMgZm9yIG91ciBkYXRhIHNldCBpbiB0ZXJtcyBvZiBlZmZlY3Qgc2l6ZXMsIGNvaG9ydHMsIHN0dWRpZXMsIHNwZWNpZXMsIGFuZCB0aGUgbnVtYmVyIG9mIGVmZmVjdCBzaXplcyBpbiB0aGUgZGlmZmVyZW50IGxldmVscyBvZiBjYXRlZ29yaWNhbCB2YXJpYWJsZXMgKGZhY3RvcnMpLCBzcGxpdCBieSBlY29sb2dpY2FsIGdyb3VwIChzZWFiaXJkLCB3YXRlcmJpcmQgYW5kIGxhbmRiaXJkKSwgYW5kIG92ZXJhbGwuICAgDQoNCmBgYHtyIHNhbXBsZS1zaXplc30NCg0KIyBtYWtpbmcgYSB0YWJsZSBvZiBzYW1wbGUgc2l6ZXMgZm9yIGRpZmZlcmVudCB2YXJpYWJsZXMNCmRmICU+JSBncm91cF9ieSh0YXhhKSAlPiUgDQogIHN1bW1hcmlzZSgNCiAgICBgRWZmZWN0IHNpemVzIChhbmFseXNlcylgID0gbigpLA0KICAgIGBDb2hvcnRgID0gbl9kaXN0aW5jdChjb2hvcnRfSUQpLA0KICAgIGBTdHVkaWVzYCA9IG5fZGlzdGluY3QoYXV0aG9ycyksDQogICAgYFNwZWNpZXNgID0gbl9kaXN0aW5jdChzcGVjaWVzX0lEKSwNCiAgICBgQXJyaXZhbCBhdCBicmVlZGluZyBncm91bmRzYCA9IHN1bShhbm51YWxfZXZlbnQgPT0gIkFycml2YWxfYnJlZWQiLCBuYS5ybT1UKSwNCiAgICBgRGVwYXJ0dXJlIGZyb20gYnJlZWRpbmcgZ3JvdW5kc2AgPSBzdW0oYW5udWFsX2V2ZW50ID09ICJEZXBhcnRfYnJlZWQiLCBuYS5ybT1UKSwNCiAgICBgQXJyaXZhbCBhdCBub24tYnJlZWRpbmcgZ3JvdW5kc2AgPSBzdW0oYW5udWFsX2V2ZW50ID09ICJOb25icmVlZF9hcnJpdmFsIiwgbmEucm09VCksDQogICAgYERlcGFydHVyZSBmcm9tIG5vbi1icmVlZGluZyBncm91bmRzYCA9IHN1bShhbm51YWxfZXZlbnQgPT0gIk5vbmJyZWVkX2RlcGFydCIsIG5hLnJtPVQpLA0KICAgIGBDb252ZW50aW9uYWwgbWV0aG9kYCA9IHN1bShtZXRob2QgPT0gIkNvbnZlbnRpb25hbCIsIG5hLnJtID0gVCksDQogICAgYEdMUyBtZXRob2RgID0gc3VtKG1ldGhvZCA9PSAiR0xTIiwgbmEucm0gPSBUKSwNCiAgICBgU2F0ZWxsaXRlIG1ldGhvZGAgID0gc3VtKG1ldGhvZCA9PSAiU2F0ZWxsaXRlIiwgbmEucm0gPSBUKSwNCiAgICBgRmVtYWxlYCA9IHN1bShzZXggPT0gIkYiLCBuYS5ybT1UKSwNCiAgICBgTWFsZWAgPSBzdW0oc2V4ID09ICJNIiwgbmEucm09VCksDQogICAgYE1peGVkIHNleGAgPSBzdW0oc2V4ID09ICJCIiwgbmEucm09VCkNCiAgKSAtPiBuX3RhYmxlMQ0KDQpuX3RhYmxlMiA8LXQobl90YWJsZTFbLC0xXSkNCmNvbG5hbWVzKG5fdGFibGUyKSA8LSBjKCJMYW5kYmlyZCIsICJTZWFiaXJkIiwgIldhdGVyYmlyZCIpDQpuX3RhYmxlMiAlPiUgYXNfdGliYmxlKHJvd25hbWVzID0gIk51bWJlciBvZiIpICU+JSANCiAgbXV0YXRlKEFsbCA9IExhbmRiaXJkICsgU2VhYmlyZCArIFdhdGVyYmlyZCkgJT4lICANCiAga2FibGUoKSAlPiUga2FibGVfc3R5bGluZygic3RyaXBlZCIsIHBvc2l0aW9uID0gImxlZnQiKSAlPiUNCiAgcGFja19yb3dzKCJBbGwgZGF0YSIsIDEsIDQpICU+JQ0KICBwYWNrX3Jvd3MoIkFubnVhbCBldmVudCIsIDUsIDgpICU+JQ0KICBwYWNrX3Jvd3MoIlRyYWNraW5nIG1ldGhvZCIsIDksIDExKSAlPiUNCiAgcGFja19yb3dzKCJTZXgiLCAxMiwgMTQpDQoNCmBgYA0KDQojIyMgTWFya2luZyBsb2NhdGlvbnMgb2Ygc3R1ZGllcw0KIyMjIyBGaWd1cmUgMQ0KYGBge3IgZmlndXJlMSwgZmlnLndpZHRoPTEwLCBmaWcuaGVpZ2h0PTV9DQoNCndvcmxkbWFwIDwtIG1hcF9kYXRhKCd3b3JsZCcpICMgZ2V0IG1hcCBkYXRhDQoNCiMgUGxvdCBvZiB3b3JsZCB3aXRoIGFsbCB0cmFja2luZyBzdHVkeSBsb2NhdGlvbnMNCndvcmxkIDwtIGdncGxvdCgpICsgZ2VvbV9wb2x5Z29uKGRhdGEgPSB3b3JsZG1hcCwgYWVzKHg9bG9uZywgeSA9IGxhdCwgZ3JvdXAgPSBncm91cCksIGZpbGwgPSAiZ3JleTgwIiwgDQogICAgICAgICAgICAgICAgICAgICAgICAgICAgICAgICAgY29sb3VyID0gTkEsIHNpemUgPSAwLjEpICsgDQogICAgY29vcmRfZml4ZWQoMS4zKSArDQogICAgdGhlbWVfbWluaW1hbCgpICsNCiAgICBzY2FsZV9jb2xvdXJfbWFudWFsKHZhbHVlcyA9IGMoIlNlYWJpcmQiID0gIiMwMDlFNzMiLCAgIkxhbmRiaXJkIiA9ICIjRDU1RTAwIiwgICJXYXRlcmJpcmQiPSAiIzAwNzJCMiIpKSArDQogICAgZ2VvbV9wb2ludChkYXRhPWRmLCBhZXMoeD0gbG9uZywgeSA9bGF0LCBjb2xvdXI9dGF4YSwgc2hhcGU9bWV0aG9kKSwgc2l6ZSA9MS41KSArDQogICAgZ2VvbV9yZWN0KGFlcyh4bWluPSAtMTIsIHhtYXg9IDIzLCB5bWluPSAzMiwgeW1heD0gNjQpLCBmaWxsID0gTkEsIGNvbG91ciA9ICJibGFjayIsIHNpemUgPSAwLjMpICsNCiAgICB0aGVtZShwYW5lbC5ncmlkID0gZWxlbWVudF9saW5lKGNvbG91ciA9ICJ3aGl0ZSIpLCBheGlzLnRpdGxlID0gZWxlbWVudF9ibGFuaygpLCBheGlzLnRleHQgPSBlbGVtZW50X2JsYW5rKCksIA0KICAgICAgICAgIGF4aXMudGlja3MgPSBlbGVtZW50X2JsYW5rKCksIGxlZ2VuZC5wb3NpdGlvbiA9ICJub25lIikNCg0KIyMgWm9vbWVkIGluIHBsb3Qgb24gRXVyb3BlDQp6b29tIDwtIGdncGxvdCgpICsgZ2VvbV9wb2x5Z29uKGRhdGEgPSB3b3JsZG1hcCwgYWVzKHg9bG9uZywgeSA9IGxhdCwgZ3JvdXAgPSBncm91cCksIGZpbGwgPSAiZ3JleTgwIiwgDQogICAgICAgICAgICAgICAgICAgICAgICAgICAgICAgICBjb2xvdXIgPSBOQSwgc2l6ZSA9IDAuMikgKyANCiAgICBjb29yZF9zZih4bGltPSBjKC0xMSwyMyksIHlsaW09IGMoMzIsNjQpKSArDQogICAgdGhlbWVfbWluaW1hbCgpICsNCiAgICB0aGVtZShsZWdlbmQudGV4dD1lbGVtZW50X3RleHQoc2l6ZT02KSwgbGVnZW5kLnRpdGxlPWVsZW1lbnRfdGV4dChzaXplPTcsIGZhY2U9ImJvbGQiKSwgbGVnZW5kLmtleS5zaXplID0gdW5pdCgxLCAibW0iKSkgKw0KICAgIHRoZW1lKHBhbmVsLmdyaWQgPSBlbGVtZW50X2xpbmUoY29sb3VyPSAid2hpdGUiKSwNCiAgICAgICAgICBheGlzLnRpdGxlID0gZWxlbWVudF9ibGFuaygpLCBheGlzLnRleHQgPSBlbGVtZW50X2JsYW5rKCksDQogICAgICAgICAgcGFuZWwuYm9yZGVyID0gZWxlbWVudF9yZWN0KGNvbG91ciA9ICJibGFjayIsIGZpbGwgPSBOQSwgc2l6ZT0wLjUpLCANCiAgICAgICAgICBheGlzLnRpY2tzID0gZWxlbWVudF9ibGFuaygpKSArDQogICAgc2NhbGVfY29sb3VyX21hbnVhbChuYW1lPSJFY29sb2dpY2FsIGdyb3VwIiwgdmFsdWVzID0gYygiU2VhYmlyZCIgPSAiIzAwOUU3MyIsICAiTGFuZGJpcmQiID0gIiNENTVFMDAiLCAgIldhdGVyYmlyZCI9ICIjMDA3MkIyIikpICsNCiAgICBzY2FsZV9zaGFwZV9kaXNjcmV0ZShuYW1lID0gIk1ldGhvZCIpICsNCiAgICBnZW9tX3BvaW50KGRhdGE9ZGYsIGFlcyh4PSBsb25nLCB5ID1sYXQsIHNoYXBlPW1ldGhvZCwgY29sb3VyPXRheGEpLHNpemUgPTEuNSkNCg0KZmlndXJlMSA8LSBwbG90X2dyaWQod29ybGQsIHpvb20sIG5yb3c9MSwgcmVsX3dpZHRocyA9IGMoMS4zLDEpKQ0KIyBnZ3NhdmUoImZpZ3MvTWFwX29mX3RhZ2dpbmdfbG9jYXRpb25zLnBkZiIsIGRwaT02MDAsIHdpZHRoPTE4MCwgaGVpZ2h0PTYwLCB1bml0cz0ibW0iKQ0KDQpmaWd1cmUxDQoNCmBgYA0KDQoqKkZpZ3VyZSAxLioqIFRoZSBtYXJraW5nIGxvY2F0aW9ucyBvZiBiaXJkcyBmb3IgYWxsIHN0dWRpZXMgd2l0aCByZXBlYXRhYmlsaXR5IGVzdGltYXRlcyBjb2xsYXRlZCBmcm9tIHRoZSBsaXRlcmF0dXJlIGFuZCBpbmNsdWRlZCBpbiBhbmFseXNlcywgY29sb3VyZWQgYnkgZWNvbG9naWNhbCBncm91cCAod2F0ZXJiaXJkLCBzZWFiaXJkLCBvciBsYW5kYmlyZCksIGFuZCBzaGFwZWQgYnkgdHJhY2tpbmcgbWV0aG9kIChjb252ZW50aW9uYWwsIHNhdGVsbGl0ZSwgb3IgR0xTKS4NCg0KIyMgTWV0YS1hbmFseXNpcw0KDQojIyMgQ2FsY3VsYXRpbmcgZWZmZWN0IHNpemVzDQoNCldlIGNyZWF0ZWQgb3VyIGVmZmVjdCBzaXplIChjb3JyZWxhdGlvbiBjb2VmZmljaWVudCAqciogYW5kIGludHJhLWNsYXNzIGNvcnJlbGF0aW9uIGNvZWZmaWNpZW50ICpJQ0MqIGFuZCB0aGVpciBGaXNoZXIncyB6IHRyYW5zZm9ybWF0aW9uICpaciopIGZyb20gcmVwZWF0YWJpbGl0eSB2YWx1ZXMgYW5kIGFzc29jaWF0ZWQgc2FtcGxlIHNpemVzLiBXZSBmb2xsb3dlZCB0aGUgZXF1YXRpb25zIG91dGxpbmVkIGluIEBob2x0bWFubl9tZXRhYm9saWNfMjAxNy4gV2UgY29udmVydGVkIHRoZSBuZWdhdGl2ZSByZXBlYXRhYmlsaXR5IGVzdGltYXRlcyAobj0xMykgaW4gb3VyIGRhdGFzZXQgdG8gemVybywgYXMgdGhleSBvZnRlbiBvbmx5IHJlZmxlY3Qgbm9pc2UgYXJvdW5kIGEgc3RhdGlzdGljYWwgemVybyBbQG5ha2FnYXdhX3JlcGVhdGFiaWxpdHlfMjAxMF0sIGJ1dCB5b3UgY2FuIGZpbmQgdGhlIG1ldGEtYW5hbHl0aWMgYW5kIG1ldGEtcmVncmVzc2lvbiBtb2RlbHMgd2l0aCB0aGUgbmVnYXRpdmUgcmVwZWF0YWJpbGl0eSBlc3RpbWF0ZXMgaW5jbHVkZWQgaW4gVGFibGVzIFMxMi0xOS4gDQoNCmBgYHtyIGVmZmVjdC1zaXplc30NCiMgQ3JlYXRpbmcgbmV3IGNvbHVtbnMgZm9yIGVmZmVjdCBzaXplcyAoWnIpIGFuZCBzYW1wbGluZyB2YXJpYW5jZSAoVlpyKQ0KZGYkVlpyIDwtIGRmJFpyIDwtIE5BDQoNCiMgQ2FsY3VsYXRlIGVmZmVjdCBzaXplcyB1c2luZyBjdXN0b20gZnVuY3Rpb24NCmZvciAoaSBpbiBhcy5udW1lcmljKHJvd25hbWVzKGRmKSkpIHsNCiAgciA8LSBkZiRSW2ldDQogIEsgPC0gZGYka1tpXQ0KICBFc3QgPC0gZGYkZXN0W2ldDQogIA0KICBaciA8LSBacl90cmFuc2Zvcm1hdGlvbihyLCBLLCBFc3QpDQogIA0KICBkZiRacltpXSA8LSBacg0KICANCn0NCiMgQ2FsY3VsYXRlIHNhbXBsaW5nIHZhcmlhbmNlcyB1c2luZyBjdXN0b20gZnVuY3Rpb24NCmZvciAoaSBpbiBhcy5udW1lcmljKHJvd25hbWVzKGRmKSkpIHsNCiAgSyA8LSBkZiRrW2ldDQogIE4gPC0gZGYkbltpXQ0KICBFc3QgPC0gZGYkZXN0W2ldDQogIA0KICBWWnIgPC0gQ2FsY19TVihLLE4sRXN0KQ0KICANCiAgZGYkVlpyW2ldIDwtIFZacg0KICANCn0NCg0KIyBBZGQgYSBuZXcgY29sdW1uIHdpdGggYWxsIG5lZ2F0aXZlIHJlcGVhdGFiaWxpdHkgdmFsdWVzIHNldCB0byB6ZXJvDQpkZiRacjIgPC0gaWZlbHNlKGRmJFpyIDwgMCwgMCwgZGYkWnIrMCkNCg0KYGBgDQoNCg0KIyMjIFBoeWxvZ2VuZXRpYyB0cmVlDQpTaW5jZSBtdWx0aXBsZSBiaXJkIHNwZWNpZXMgKG4gPSBgciAoZHBseXI6Om5fZGlzdGluY3QoZGYkc3BlY2llc19JRCkpYCkgYXJlIGluY2x1ZGVkIGluIHRoaXMgZGF0YXNldCwgd2UgbmVlZGVkIHRvIGNvbnNpZGVyIHBoeWxvZ2VuZXRpYyBub24taW5kZXBlbmRlbmNlIGluIG91ciBtb2RlbHMgW0BjaGFtYmVybGFpbl9kb2VzXzIwMTIsIENpbmFyIGV0IGFsLiAyMDIyXS4gVG8gZ2VuZXJhdGUgdGhlIHBoeWxvZ2VueSwgd2UgdXNlZCBhIHBoeWxvZ2VuZXRpYyB0cmVlIGZyb20gQGpldHpfZ2xvYmFsXzIwMTIgKHByb3ZpZGVkIGJ5IEJlbmVkaWt0IEhvbHRtYW5uKSwgd2hpY2ggd2FzIHByZXBhcmVkIG9uIHRoZSBiYXNpcyBvZiBIYWNrZXR0IGJhY2tib25lIFtIYWNrZXR0IHRyZWU7IEBoYWNrZXR0X3BoeWxvZ2Vub21pY18yMDA4XS4gV2UgZmlyc3Qgc2VhcmNoZWQgdGhlIHRyZWUgZm9yIHRoZSBzcGVjaWVzIGluIG91ciBkYXRhc2V0IHRvIGNvbmZpcm0gYW5kIGNvcnJlY3QgdGhlIHNwZWNpZXMgbmFtZXMuIA0KDQotIF9MaW1vc2EgbGFwcG9uaWNhIGJhdWVyaV8gd2FzIGFzc3VtZWQgdG8gYmUgc3lub255bSB0byBfTGltb3NhIGxhcHBvbmljYV8NCi0gX0xpbW9zYSBsaW1vc2EgbGltb3NhXyBhbmQgX0xpbW9zYSBsaW1vc2EgaXNsYW5kaWNhXyB3ZXJlIGFzc3VtZWQgdG8gYmUgc3lub255bSB0byBfTGltb3NhIGxpbW9zYV8NCi0gX0N5Z251cyBjb2x1bWJpYW51cyBiZXdpY2tpaV8gd2FzIGFzc3VtZWQgdG8gYmUgc3lub255bSB0byBfQ3lnbnVzIGNvbHVtYmlhbnVzXw0KLSBfQ2F0aGFyYWN0YSBhbnRhcmN0aWNhIGxvbm5iZXJnaV8gd2FzIGFzc3VtZWQgdG8gYmUgc3lub255bSB0byBfQ2F0aGFyYWN0YSBhbnRhcmN0aWNhXw0KLSBfUHRlcm9kcm9tYSBkZXNlcnRhXyB3YXMgYXNzdW1lZCB0byBiZSBzeW5vbnltIHRvIF9QdGVyb2Ryb21hIG1vbGxpc18NCi0gX0NoZW4gY2FuYWdpY3VzXyB3YXMgYXNzdW1lZCB0byBiZSBzeW5vbnltIHRvIF9DaGVuIGNhbmFnaWNhXw0KLSBfQW5zZXIgY2FlcnVsZXNjZW5zIGF0bGFudGljdXNfIHdhcyBhc3N1bWVkIHRvIGJlIHN5bm9ueW0gdG8gX0NoZW4gY2FlcnVsZXNjZW5zXw0KDQpXZSB0aGVuIHRyaW1tZWQgdGhlIHRyZWUgdG8gaW5jbHVkZSBvbmx5IHRoZSBzcGVjaWVzIG5hbWVzIGluIG91ciBkYXRhIHNldCwgYW5kIGNvbXB1dGVkIGJyYW5jaCBsZW5ndGhzIHVzaW5nIEdyYWZlbidzIG1ldGhvZCAoR3JhZmVuLCAxOTg5KSBpbiB0aGUgY29tcHV0ZS5icmxlbiBmdW5jdGlvbiBpbiB0aGUgUiBwYWNrYWdlIGFwZSBbQHBhcmFkaXNfYXBlXzIwMTldLg0KDQojIyMjIEZpZ3VyZSBTMw0KYGBge3IgcGh5bG8tdHJlZSwgZmlnLndpZHRoPTEwLCBmaWcuaGVpZ2h0PTh9DQoNCnNwZWNpZXNfbGlzdCA8LSB1bmlxdWUoZGYkc3BlY2llc19sYXRpbikgIyB1c2UgdW5pcXVlKCkgYXMgc29tZSBuYW1lcyBhcmUgcmVwZWF0ZWQNCnNwZWNpZXNfbGlzdDwtZ3N1YigiICIsICJfIiwgc3BlY2llc19saXN0KSAjIHJlcGxhY2Ugc3BhY2VzIHdpdGggdW5kZXJzY29yZSBzbyB0aGV5IG1hdGNoIHRyZWUNCg0KIyBMb2FkIGJpcmQgc3VwZXJ0cmVlIGJhc2VkIG9uIEhhY2tldHQncyBiYWNrYm9uZSANCmJpcmRfdHJlZV9oYWNrZXR0IDwtIHJlYWQudHJlZShoZXJlKCJkYXRhIiwgIkhhY2tldHQudHJlIikpICMgdHJlZSBwcm92aWRlZCBieSBCZW5lZGlrdCBIb2x0bWFubg0KDQojIFBydW5lIHBoeWxvZ2VudGljIHRyZWUgZm9yIG1ldGEtYW5hbHlzaXMNCg0KIyBDaGVjayB0aGUgdHJlZQ0KIyBiaXJkX3RyZWVfaGFja2V0dCAjIDk5OTMgdGlwcyA9IHNwZWNpZXMNCiMgc3RyKGJpcmRfdHJlZV9oYWNrZXR0KSAjIGhhcyBlZGdlIChicmFuY2gpIGxlbmd0aHMNCmJpcmRfdHJlZV9oYWNrZXR0IDwtIGNvbGxhcHNlLnNpbmdsZXMoYmlyZF90cmVlX2hhY2tldHQpDQoNCiMgR2V0IG9ubHkgYmlyZCBzcGVjaWVzIGZyb20gdGhlIHN1cGVydHJlZSB0aGF0IGFyZSBhbHNvIGluY2x1ZGVkIGluIGNvbGxlY3RlZCBkYXRhDQpiaXJkX3RyZWVfc3BlY2llcyA8LSBhcy5jaGFyYWN0ZXIoYmlyZF90cmVlX2hhY2tldHQkdGlwLmxhYmVsKQ0KDQojIENoZWNrIHRoZSBvdmVybGFwIG9mIHNwZWNpZXMgbmFtZXMgYmV0d2VlbiBjb2xsZWN0ZWQgZGF0YSBmaWxlIGFuZCB0aGUgc3VwZXJ0cmVlDQojIEFsbCBzcGVjaWVzIHNob3VsZCBiZSBwcmVzZW50LiBJZiBub3QsIHNwZWNpZXMgbmFtZXMgbWF5IG5vdCBtYXRjaCB3aXRoIG5hbWVzIGluIHRoZSBzdXBlcnRyZWUNCiMgaW50ZXJzZWN0KGJpcmRfdHJlZV9zcGVjaWVzLCBzcGVjaWVzX2xpc3QpIA0KIyBjaGFyYWN0ZXIoMzkpIC0gc2hvdWxkIGJlIDQ3LCBuZWVkIHRvIGNoZWNrIHdoaWNoIHNwZWNpZXMgZG8vZG8gbm90IG1hdGNoDQoNCiMgZ2l2ZXMgbGlzdCBvZiBzcGVjaWVzIG5hbWVzIHdoaWNoIGFyZSBub3QgbWF0Y2hlZA0KIyBzcGVjaWVzX2xpc3RbIXNwZWNpZXNfbGlzdCAlaW4lIGJpcmRfdHJlZV9zcGVjaWVzXQ0KIyJMaW1vc2FfbGFwcG9uaWNhX2JhdWVyaSINCiMiQ3lnbnVzX2NvbHVtYmlhbnVzX2Jld2lja2lpIg0KIyJMaW1vc2FfbGltb3NhX2xpbW9zYSINCiMiTGltb3NhX2xpbW9zYV9pc2xhbmRpY2EiDQojIkNhdGhhcmFjdGFfYW50YXJjdGljYV9sb25uYmVyZ2kiDQojIlB0ZXJvZHJvbWFfZGVzZXJ0YSINCiMiQ2hlbl9jYW5hZ2ljdXMiDQojIkFuc2VyX2NhZXJ1bGVzY2Vuc19hdGxhbnRpY3VzIg0KDQojIG1ha2luZyBhIG5ldyBsaXN0IHNvIGl0IGlzIGNsZWFyIHRoYXQgdGhpcyBsaXN0IG9mIHNwZWNpZXMgaXMgYW4gdXBkYXRlZCB2ZXJzaW9uIGNvbXBhcmVkIHRvIHRoZSBvcmlnaW5hbCBvbmUNCnNwZWNpZXNfbGlzdF9oYWNrZXR0IDwtIHNwZWNpZXNfbGlzdA0KDQojIENoYW5naW5nIHN1YnNwZWNpZXMgdG8gc3BlY2llcywgb3Igb2xkIGdlbnVzIG5hbWVzIGZyb20gcGFwZXJzIHRvIG5ldyANCiMgT25seSBQdGVyb2Ryb21hIGRlc2VydGE6IDMgc3BlY2llcyBpbiB0aGUgUHRlcm9kcm9tYSBmZWFlL21hZGVpcmEvZGVzZXJ0YWUgY29tcGxleCB3ZXJlIG9uY2UgYmVsaWV2ZWQgdG8gYmUgc3Vic3BlY2llcyBvZiBhIHNpbmdsZSBzcGVjaWVzOiBQdGVyb2Ryb21hIG1vbGxpcw0Kc3BlY2llc19saXN0X2hhY2tldHQgPC0gcmVwbGFjZShzcGVjaWVzX2xpc3RfaGFja2V0dCwgc3BlY2llc19saXN0X2hhY2tldHQ9PSJMaW1vc2FfbGFwcG9uaWNhX2JhdWVyaSIsICJMaW1vc2FfbGFwcG9uaWNhIikNCnNwZWNpZXNfbGlzdF9oYWNrZXR0IDwtIHJlcGxhY2Uoc3BlY2llc19saXN0X2hhY2tldHQsIHNwZWNpZXNfbGlzdF9oYWNrZXR0PT0iTGltb3NhX2xpbW9zYV9saW1vc2EiIHwgc3BlY2llc19saXN0X2hhY2tldHQ9PSJMaW1vc2FfbGltb3NhX2lzbGFuZGljYSIgLCAiTGltb3NhX2xpbW9zYSIpDQpzcGVjaWVzX2xpc3RfaGFja2V0dCA8LSByZXBsYWNlKHNwZWNpZXNfbGlzdF9oYWNrZXR0LCBzcGVjaWVzX2xpc3RfaGFja2V0dD09IkN5Z251c19jb2x1bWJpYW51c19iZXdpY2tpaSIsICJDeWdudXNfY29sdW1iaWFudXMiKQ0Kc3BlY2llc19saXN0X2hhY2tldHQgPC0gcmVwbGFjZShzcGVjaWVzX2xpc3RfaGFja2V0dCwgc3BlY2llc19saXN0X2hhY2tldHQ9PSJDYXRoYXJhY3RhX2FudGFyY3RpY2FfbG9ubmJlcmdpIiwgIkNhdGhhcmFjdGFfYW50YXJjdGljYSIpDQpzcGVjaWVzX2xpc3RfaGFja2V0dCA8LSByZXBsYWNlKHNwZWNpZXNfbGlzdF9oYWNrZXR0LCBzcGVjaWVzX2xpc3RfaGFja2V0dD09IlB0ZXJvZHJvbWFfZGVzZXJ0YSIsICJQdGVyb2Ryb21hX21vbGxpcyIpDQpzcGVjaWVzX2xpc3RfaGFja2V0dCA8LSByZXBsYWNlKHNwZWNpZXNfbGlzdF9oYWNrZXR0LCBzcGVjaWVzX2xpc3RfaGFja2V0dD09IkNoZW5fY2FuYWdpY3VzIiwgIkNoZW5fY2FuYWdpY2EiKQ0Kc3BlY2llc19saXN0X2hhY2tldHQgPC0gcmVwbGFjZShzcGVjaWVzX2xpc3RfaGFja2V0dCwgc3BlY2llc19saXN0X2hhY2tldHQ9PSJBbnNlcl9jYWVydWxlc2NlbnNfYXRsYW50aWN1cyIsICJDaGVuX2NhZXJ1bGVzY2VucyIpDQoNCiMgTm93IGNoZWNrIGFuZCBzZWUgaWYgYWxsIHNwZWNpZXMgYXJlIHByZXNlbnQgaW4gc3VwZXJ0cmVlDQojIGludGVyc2VjdChiaXJkX3RyZWVfc3BlY2llcywgc3BlY2llc19saXN0X2hhY2tldHQpICMgPSA0NyAobm90IDQ4LCBiZWNhdXNlIEwuIGwuIGxpbW9zYSBhbmQgTC4gaS4gaXNsYW5kaWNhIGJvdGggY2hhbmdlZCB0byBMLiBsaW1vc2EpDQoNCiMgUHJ1bmUgc3VwZXJ0cmVlIHRvIHRoZSBsaXN0IG9mIHRheGEgaW5jbHVkZWQgaW4gdGhlIGRhdGENCnBydW5lZF9iaXJkc19zdHJlZSA8LSBkcm9wLnRpcChiaXJkX3RyZWVfaGFja2V0dCwgYmlyZF90cmVlX2hhY2tldHQkdGlwLmxhYmVsWy1tYXRjaChzcGVjaWVzX2xpc3RfaGFja2V0dCwgYmlyZF90cmVlX2hhY2tldHQkdGlwLmxhYmVsKV0pDQoNCiMgQ2hlY2sgaWYgdHJlZSBpcyBiaW5hcnkgYW5kIHVsdHJhbWV0cmljDQojIGlzLmJpbmFyeShwcnVuZWRfYmlyZHNfc3RyZWUpICMgVFJVRQ0KIyBpcy51bHRyYW1ldHJpYyhwcnVuZWRfYmlyZHNfc3RyZWUpICMgVFJVRQ0KDQojIFNhdmUgcHJ1bmVkIHRyZWUgdG8gYmUgdXNlIGluIHRoZSBtZXRhLWFuYWx5c2lzDQp3cml0ZS50cmVlKHBydW5lZF9iaXJkc19zdHJlZSwNCiAgICAgICAgICAgZmlsZSA9IGhlcmUoImRhdGEiLCAiYmlyZHNfbWV0YS1hbmFseXNpc190cmVlLnRyZSIpLCBhcHBlbmQgPSBGQUxTRSwNCiAgICAgICAgICAgZGlnaXRzID0gMTAsIHRyZWUubmFtZXMgPSBGQUxTRQ0KKQ0KDQojIE1ha2UgcGh5bG9nZW5ldGljIGNvcnJlbGF0aW9uIG1hdHJpeA0KDQojIFVzaW5nIG1ldGFmb3IgLSBjb3JyZWxhdGlvbiBtYXRyaXggZm9yIHNwZWNpZXMgaW4gdHJlZQ0KdmFyY29yIDwtIHZjdihwcnVuZWRfYmlyZHNfc3RyZWUsIGNvcnIgPSBUUlVFKQ0KDQojIENyZWF0ZSBhIGNvbHVtbiBjYWxsZWQgcGh5bG9nZW55LCB3aGljaCBtYXRjaGVzIHRoZSB0cmVlDQojIENvcGllZCBjb2x1bW4gdG8gZWRpdCBzcGVjaWVzIG5hbWVzIHRvIG1hdGNoIHRyZWUgKGFzIHNvbWUgc3AuIG5hbWVzIGFyZSBmcm9tIG9sZCBwYXBlcnMsIG9yIGFyZSBzdWJzcGVjaWVzKQ0KZGYkc3BlY2llc19sYXRpbl9oYWNrZXR0IDwtIGFzLmNoYXJhY3RlcihkZiRzcGVjaWVzX2xhdGluKSANCmRmJHNwZWNpZXNfbGF0aW5faGFja2V0dCA8LXJlcGxhY2UoZGYkc3BlY2llc19sYXRpbl9oYWNrZXR0LCBkZiRzcGVjaWVzX2xhdGluX2hhY2tldHQgPT0gIkxpbW9zYSBsYXBwb25pY2EgYmF1ZXJpIiwgIkxpbW9zYSBsYXBwb25pY2EiKQ0KZGYkc3BlY2llc19sYXRpbl9oYWNrZXR0IDwtIHJlcGxhY2UoZGYkc3BlY2llc19sYXRpbl9oYWNrZXR0LCBkZiRzcGVjaWVzX2xhdGluX2hhY2tldHQ9PSJMaW1vc2EgbGltb3NhIGxpbW9zYSIgfCBkZiRzcGVjaWVzX2xhdGluX2hhY2tldHQ9PSJMaW1vc2EgbGltb3NhIGlzbGFuZGljYSIgLCAiTGltb3NhIGxpbW9zYSIpDQpkZiRzcGVjaWVzX2xhdGluX2hhY2tldHQgPC1yZXBsYWNlKGRmJHNwZWNpZXNfbGF0aW5faGFja2V0dCwgZGYkc3BlY2llc19sYXRpbl9oYWNrZXR0ID09ICJDeWdudXMgY29sdW1iaWFudXMgYmV3aWNraWkiLCAiQ3lnbnVzIGNvbHVtYmlhbnVzIikNCmRmJHNwZWNpZXNfbGF0aW5faGFja2V0dCA8LXJlcGxhY2UoZGYkc3BlY2llc19sYXRpbl9oYWNrZXR0LCBkZiRzcGVjaWVzX2xhdGluX2hhY2tldHQgPT0gIkNhdGhhcmFjdGEgYW50YXJjdGljYSBsb25uYmVyZ2kiLCAiQ2F0aGFyYWN0YSBhbnRhcmN0aWNhIikNCmRmJHNwZWNpZXNfbGF0aW5faGFja2V0dCA8LXJlcGxhY2UoZGYkc3BlY2llc19sYXRpbl9oYWNrZXR0LCBkZiRzcGVjaWVzX2xhdGluX2hhY2tldHQgPT0gIlB0ZXJvZHJvbWEgZGVzZXJ0YSIsICJQdGVyb2Ryb21hIG1vbGxpcyIpDQpkZiRzcGVjaWVzX2xhdGluX2hhY2tldHQgPC1yZXBsYWNlKGRmJHNwZWNpZXNfbGF0aW5faGFja2V0dCwgZGYkc3BlY2llc19sYXRpbl9oYWNrZXR0ID09ICJDaGVuIGNhbmFnaWN1cyIsICJDaGVuIGNhbmFnaWNhIikNCmRmJHNwZWNpZXNfbGF0aW5faGFja2V0dCA8LXJlcGxhY2UoZGYkc3BlY2llc19sYXRpbl9oYWNrZXR0LCBkZiRzcGVjaWVzX2xhdGluX2hhY2tldHQgPT0gIkFuc2VyIGNhZXJ1bGVzY2VucyBhdGxhbnRpY3VzIiwgIkNoZW4gY2FlcnVsZXNjZW5zIikNCg0KZGYkcGh5bG9nZW55PC1nc3ViKCIgIiwgIl8iLCBkZiRzcGVjaWVzX2xhdGluX2hhY2tldHQpDQoNCiMgUGxvdCB0cmVlIHRvIHNlZSBob3cgaXQgbG9va3MgbGlrZQ0KcGxvdChwcnVuZWRfYmlyZHNfc3RyZWUsIGxhYmVsLm9mZnNldCA9IDIsIGNleCA9IDAuOCwgbWFpbiA9ICInSGFja2V0dCB0cmVlJyIsIGNleC5tYWluID0gMSwgbGluZSA9IDAuNSkgIyB3aXRoIGJyYW5jaCBsZW5ndGhzDQpgYGANCg0KKipGaWd1cmUgUzMuKiogUGh5bG9nZW5ldGljIHRyZWUgKHdpdGggSGFja2V0dCBiYWNrYm9uZSkgdXNlZCBmb3IgcGh5bG9nZW5ldGljIG1ldGEtYW5hbHlzaXMgYW5kIG1ldGEtcmVncmVzc2lvbiBvbiByZXBlYXRhYmlsaXR5IGluIGF2aWFuIG1pZ3JhdG9yeSB0aW1pbmdzLg0KDQojIyMgTWV0YS1hbmFseXRpYyBtb2RlbA0KDQojIyMjIFJ1bm5pbmcgbXVsdGlsZXZlbCBtZXRhLWFuYWx5dGljIG1vZGVsIHdpdGggYW5kIHdpdGhvdXQgcGh5bG9nZW55DQoNCldlIHVzZWQgdGhlIGBybWEubXZgIGZ1bmN0aW9uIGZyb20gdGhlIHBhY2thZ2UgYG1ldGFmb3JgIFtAdmllY2h0YmF1ZXJfY29uZHVjdGluZ18yMDEwXSB0byBydW4gYWxsIG1ldGEtYW5hbHl0aWMgbW9kZWxzIGFuZCBtZXRhLXJlZ3Jlc3Npb25zLiBUaGlzIGZ1bmN0aW9uIGFsbG93cyB1cyB0byBpbmNvcnBvcmF0ZSB2YXJpYW5jZS1jb3ZhcmlhbmNlIG1hdHJpY2VzIGluIHRoZSBWIHRlcm0uIFdlIGFzc3VtZWQgYSBjb3JyZWxhdGlvbiBvZiAwLjUgDQpbQG5vYmxlX25vbmluZGVwZW5kZW5jZV8yMDE3XSB0aGF0IHdpbGwgYmUgZml0dGVkIGFzIHBhcnQgb2YgdGhlIHJhbmRvbSBlZmZlY3Qgc3RydWN0dXJlIG9mIG91ciBtb2RlbHMgYnV0IGFsc28gdGVzdCBkaWZmZXJlbnQgbGV2ZWxzIG9mIGNvcnJlbGF0aW9uIChzZWUgU2Vuc2l0aXZpdHkgYW5hbHlzaXMpLg0KDQpBcyB3ZSBoYXZlIGEgbXVsdGktc3BlY2llcyBkYXRhc2V0LCB3ZSB1c2UgdGhlIG1vZGVsIHRoYXQgYWNjb3VudHMgZm9yIGJvdGggdGhlIG5vbi1waHlsb2dlbmV0aWMgYW5kIHBoeWxvZ2VuZXRpYyBzcGVjaWVzLWxldmVsIHZhcmlhbmNlIGluIGFkZGl0aW9uIHRvIHRoZSBmdWxsIG11bHRpbGV2ZWwgc3RydWN0dXJlIG9mIHRoZSBkYXRhIGFzIGFueSBhdHRlbXB0cyB0byBzaW1wbGlmeSB0aGlzIG1vZGVsLCBzdWNoIGFzIHVzaW5nIG9ubHkgdGhlIHBoeWxvZ2VuZXRpYyB2YXJpYW5jZSBjb21wb25lbnQsIG1heSBsZWFkIHRvIGVycm9uZW91cyBpbmZlcmVuY2VzIGZyb20gdGhlIGRhdGEgKENpbmFyIGV0IGFsLiAyMDIyKS4NCg0KYGBge3IgbWV0YS1hbmFseXRpYy1tb2RlbHN9DQojIENyZWF0ZSBhIHZhcmlhbmNlLWNvdmFyaWFuY2UgbWF0cml4IGF0IHRoZSBjb2hvcnQgbGV2ZWwNClZDViA8LSBpbXB1dGVfY292YXJpYW5jZV9tYXRyaXgodmkgPSBkZiRWWnIsIGNsdXN0ZXIgPSBkZiRjb2hvcnRfSUQsIHIgPSAwLjUpDQojIFdoeSAwLjUgLSBzZWUgaHR0cHM6Ly9vbmxpbmVsaWJyYXJ5LndpbGV5LmNvbS9kb2kvZnVsbC8xMC4xMTExL21lYy4xNDAzMQ0KDQptYV9tb2RlbDEgPC0gcm1hLm12KHlpID0gWnIyLCBWID0gVkNWLA0KICAgICAgICAgICAgICAgICAgIHJhbmRvbSA9IGxpc3QofjEgfCBlc19JRCwgDQogICAgICAgICAgICAgICAgICAgICAgICAgICAgICAgICB+MSB8IHBhcGVyX0lELCANCiAgICAgICAgICAgICAgICAgICAgICAgICAgICAgICAgIH4xIHwgY29ob3J0X0lELCANCiAgICAgICAgICAgICAgICAgICAgICAgICAgICAgICAgIH4xIHwgc3BlY2llc19JRCksIA0KICAgICAgICAgICAgICAgICAgIGRhdGEgPSBkZikNCg0KbWFfbW9kZWwyIDwtIHJtYS5tdih5aSA9IFpyMiwgViA9IFZDViwgDQogICAgICAgICAgICAgICAgICAgICAgcmFuZG9tID0gbGlzdCh+MSB8IGVzX0lELCANCiAgICAgICAgICAgICAgICAgICAgICAgICAgICAgICAgICAgIH4xIHwgcGFwZXJfSUQsIA0KICAgICAgICAgICAgICAgICAgICAgICAgICAgICAgICAgICAgfjEgfCBjb2hvcnRfSUQsIA0KICAgICAgICAgICAgICAgICAgICAgICAgICAgICAgICAgICAgfjEgfCBzcGVjaWVzX0lELCAjIG5vbi1waHlsbyBlZmZlY3QNCiAgICAgICAgICAgICAgICAgICAgICAgICAgICAgICAgICAgIH4xIHwgcGh5bG9nZW55KSwgIyBwaHlsbyBlZmZlY3QNCiAgICAgICAgICAgICAgICAgICAgICBSID0gbGlzdChwaHlsb2dlbnkgPSB2YXJjb3IpLCAjIHBoeWxvZ2VuZXRpYyByZWxhdGVkbmVzcw0KICAgICAgICAgICAgICAgICAgICAgIGRhdGEgPSBkZikNCg0KYGBgDQoNCiMjIyMgVGFibGUgUzMNCk92ZXJhbGwgZWZmZWN0cyAobWV0YS1hbmFseXRpYyBtZWFucykgYW5kIDk1JSBjb25maWRlbmNlIGludGVydmFscyAoQ0lzKSBib3RoIGluICpaciogYW5kIGJhY2stdHJhbnNmb3JtZWQgdG8gKklDQyosIGFuZCBoZXRlcm9nZW5laXR5LCAqSSpeMl4sIGZvciB0aGUgbXVsdGlsZXZlbCBpbnRlcmNlcHQtb25seSBtZXRhLWFuYWx5c2lzIG1vZGVscyBpbmNsdWRpbmcgYW5kIGV4Y2x1ZGluZyBwaHlsb2dlbnkuDQoNCmBgYHtyIG1ldGEtYW5hbHl0aWMtdGFibGV9DQojIyAjIGVzdGltYXRpbmcgSTIgYXMgbWVhc3VyZSBvZiBoZXRlcm9nZW5laXR5DQppMl9tYTEgPC0gcm91bmQoaTJfbWwobWFfbW9kZWwxKSoxMDAsMSkNCmkyX21hMiA8LSByb3VuZChpMl9tbChtYV9tb2RlbDIpKjEwMCwxKQ0KDQojIEJhY2stdHJhbnNmb3JtIHRvIElDQw0KbWExIDwtIG1vZF9yZXN1bHRzKG1hX21vZGVsMSwgbW9kPSJJbnQiKQ0KbWExX21vZF90YWJsZSA8LSBtYTEkbW9kX3RhYmxlDQoNCm1hMiA8LSBtb2RfcmVzdWx0cyhtYV9tb2RlbDIsIG1vZD0iSW50IikNCm1hMl9tb2RfdGFibGUgPC0gbWEyJG1vZF90YWJsZQ0KDQojIG5lZWQgdG8gY2FsY3VsYXRlIGsgZm9yIHdob2xlIGRhdGEgc2V0IHRvIHVzZSBpbiBmb3JtdWxhDQoNCmtfYWxsIDwtIG1lYW4oZGYkaykNCg0KZm9yKGkgaW4gbmFtZXMobWExX21vZF90YWJsZSlbMjo2XSl7DQogIA0KICBtYTFfbW9kX3RhYmxlW2ldIDwtIFpyX3RvX0lDQyhtYTFfbW9kX3RhYmxlW2ldLCBrX2FsbCkNCiAgDQp9DQoNCmZvcihpIGluIG5hbWVzKG1hMl9tb2RfdGFibGUpWzI6Nl0pew0KICANCiAgbWEyX21vZF90YWJsZVtpXSA8LSBacl90b19JQ0MobWEyX21vZF90YWJsZVtpXSwga19hbGwpDQogIA0KfQ0KDQojIGNyZWF0aW5nIGEgdGFibGUNCnRpYmJsZSgNCiAgTW9kZWwgPSBjKCJNZXRhLWFuYWx5c2lzIChacikiLCAiTWV0YS1hbmFseXNpcyAoSUNDKSIsICJNZXRhLWFuYWx5c2lzIHBoeWxvIChacikiLCAiTWV0YS1hbmFseXNpcyBwaHlsbyAoSUNDKSIpLA0KICBgT3ZlcmFsbCBtZWFuYCA9IGMobWFfbW9kZWwxJGIsIG1hMV9tb2RfdGFibGUkZXN0aW1hdGUsIG1hX21vZGVsMiRiLCBtYTJfbW9kX3RhYmxlJGVzdGltYXRlKSwNCiAgYExvd2VyIENJIFswLjAyNV1gID0gYyhtYV9tb2RlbDEkY2kubGIsIG1hMV9tb2RfdGFibGUkbG93ZXJDTCwgbWFfbW9kZWwyJGNpLmxiLCBtYTJfbW9kX3RhYmxlJGxvd2VyQ0wpLA0KICBgVXBwZXIgQ0kgWzAuOTc1XWAgPSBjKG1hX21vZGVsMSRjaS51YiwgbWExX21vZF90YWJsZSR1cHBlckNMLCBtYV9tb2RlbDIkY2kudWIsIG1hMl9tb2RfdGFibGUkdXBwZXJDTCksDQogIGBJXjJefnRvdGFsfmAgPSBjKGkyX21hMVsxXSwgTkEsIGkyX21hMlsxXSwgTkEpLA0KICBgSV4yXn5lc35gID0gYyhpMl9tYTFbMl0sIE5BLCBpMl9tYTJbMl0sIE5BKSwNCiAgYEleMl5+cGFwZXJ+YCA9IGMoaTJfbWExWzNdLCBOQSwgaTJfbWEyWzNdLCBOQSksDQogIGBJXjJefmNvaG9ydH5gID0gYyhpMl9tYTFbNF0sIE5BLCBpMl9tYTJbNF0sIE5BKSwNCiAgYEleMl5+c3BlY2llc35gID0gYyhpMl9tYTFbNV0sIE5BLCBpMl9tYTJbNV0sIE5BKSwNCiAgYEleMl5+cGh5bG9+YCA9IGMoTkEsIE5BLCBpMl9tYTJbNl0sIE5BKSwpICU+JSANCiAga2FibGUoImh0bWwiLCAgZGlnaXRzID0gMykgJT4lDQogIGthYmxlX3N0eWxpbmcoInN0cmlwZWQiLCBwb3NpdGlvbiA9ICJsZWZ0IikNCmBgYA0KDQojIyMjIEZpZ3VyZSAyYQ0KDQpgYGB7ciBmaWd1cmUyYSwgIGZpZy53aWR0aD03LCBmaWcuaGVpZ2h0PTJ9DQoNCm1hMl9kYXRhIDwtIG1hMiRkYXRhDQoNCiMgYmFjay10cmFuc2Zvcm0gbW9kZWwgcmVzdWx0cyB0byBJQ0MNCiMgbmVlZCB0byBjYWxjdWxhdGUgayBmb3Igd2hvbGUgZGF0YSBzZXQgdG8gdXNlIGluIGZvcm11bGENCg0KbWEyX2RhdGEkeWlfSUNDIDwtIFpyX3RvX0lDQyhtYTJfZGF0YSR5aSwga19hbGwpDQoNCm1hMl9kYXRhJG1vZGVyYXRvciA8LSBmYWN0b3IobWEyX2RhdGEkbW9kZXJhdG9yLCBsZXZlbHMgPSBtYTJfbW9kX3RhYmxlJG5hbWUsIGxhYmVscyA9IG1hMl9tb2RfdGFibGUkbmFtZSkNCm1hMl9kYXRhJHNjYWxlIDwtICgxL3NxcnQobWEyX2RhdGFbLCJ2aSJdKSkNCm1hMl9tb2RfdGFibGUkSyA8LSBhcy52ZWN0b3IoYnkobWEyX2RhdGEsIG1hMl9kYXRhWywibW9kZXJhdG9yIl0sIGZ1bmN0aW9uKHgpIGxlbmd0aCh4WywieWkiXSkpKQ0KbWEyX2dyb3VwX25vIDwtIG5yb3cobWEyX21vZF90YWJsZSkNCg0KIyBjb2xvdXIgYmxpbmQgZnJpZW5kbHkgY29sb3VycyB3aXRoIGdyZXkNCmNicGwgPC0gYygiI0U2OUYwMCIsIiMwMDlFNzMiLCAiI0YwRTQ0MiIsICIjMDA3MkIyIiwgIiNENTVFMDAiLCAiI0NDNzlBNyIsICIjNTZCNEU5IiwgIiM5OTk5OTkiKQ0KDQojIGNyZWF0aW5nIGFuIG9yY2hhcmQgcGxvdA0KZmlnX21hMiA8LSBnZ3Bsb3QoZGF0YSA9IG1hMl9tb2RfdGFibGUsIGFlcyh4ID0gZXN0aW1hdGUsIHkgPSAiT3ZlcmFsbCBtZWFuIikpICsgDQogICAgIHNjYWxlX3hfY29udGludW91cyhsaW1pdHMgPSBjKC0wLjM3LCAxKSwgYnJlYWtzID0gc2VxKC0wLjQsIDEsIGJ5ID0gMC4yKSwgbGFiZWxzID0gYygiLTAuNCIsICItMC4yIiwgIjAuMCIsICIwLjIiLCAiMC40IiwgIjAuNiIsICIwLjgiLCAiMS4wIikpICsNCiAgICBnZ2JlZXN3YXJtOjpnZW9tX3F1YXNpcmFuZG9tKGRhdGEgPSBtYTJfZGF0YSwgYWVzKHggPSB5aV9JQ0MsIHkgPSAiT3ZlcmFsbCBtZWFuIiwgc2l6ZSA9IHNjYWxlKSwgZmlsbD0iYmxhY2siLCBncm91cE9uWD1GQUxTRSwgYWxwaGEgPSAwLjIpICsNCiAgICBnZW9tX2Vycm9yYmFyaChhZXMoeG1pbiA9IGxvd2VyUFIsIHhtYXggPSB1cHBlclBSKSwgaGVpZ2h0ID0gMCwgc2hvdy5sZWdlbmQgPSBGLCBzaXplID0gMC41LCBhbHBoYSA9IDAuNikgKw0KICAgIGdlb21fZXJyb3JiYXJoKGFlcyh4bWluID0gbG93ZXJDTCwgeG1heCA9IHVwcGVyQ0wpLCBoZWlnaHQgPSAwLCBzaG93LmxlZ2VuZCA9IEYsIHNpemUgPSAxLjIpICsgDQogICAgZ2VvbV92bGluZSh4aW50ZXJjZXB0ID0gMCwgbGluZXR5cGUgPSAyLCBjb2xvdXIgPSAiYmxhY2siLCBhbHBoYSA9IDAuNSkgKw0KICAgIGdlb21fcG9pbnQoZmlsbD0iYmxhY2siLCBzaXplID0gMywgc2hhcGUgPSAyMSkgKyANCiAgICBhbm5vdGF0ZSgndGV4dCcsIHggPSAwLjkzLCB5ID0gKHNlcSgxLCBtYTJfZ3JvdXBfbm8sIDEpKzAuMzUpLCBsYWJlbD0gcGFzdGUoIml0YWxpYyhrKT09IiwgbWEyX21vZF90YWJsZSRLKSwgcGFyc2U9VFJVRSwgaGp1c3QgPSAibGVmdCIsIHNpemU9My41KSArDQogICAgZ2dwbG90Mjo6dGhlbWVfYncoKSArDQogICAgZ2dwbG90Mjo6Z3VpZGVzKGZpbGw9Im5vbmUiLCBjb2xvdXI9Im5vbmUiKSArDQogICAgZ2dwbG90Mjo6dGhlbWUobGVnZW5kLnBvc2l0aW9uID0gYygxLDApLCBsZWdlbmQuanVzdGlmaWNhdGlvbiA9IGMoMSwwKSwgbGVnZW5kLnRpdGxlID0gZWxlbWVudF90ZXh0KHNpemU9OCksIA0KICAgICAgICAgICAgICAgICAgIGxlZ2VuZC50ZXh0ID0gZWxlbWVudF90ZXh0KHNpemU9OCksIGxlZ2VuZC5rZXkgPSBlbGVtZW50X3JlY3QoY29sb3VyID0gTkEsIGZpbGwgPSBOQSksIA0KICAgICAgICAgICAgICAgICAgIGxlZ2VuZC5iYWNrZ3JvdW5kID0gZWxlbWVudF9ibGFuaygpLCBsZWdlbmQuZGlyZWN0aW9uID0gImhvcml6b250YWwiLA0KICAgICAgICAgICAgICAgICAgIGF4aXMudGV4dC54ID0gZWxlbWVudF90ZXh0KHNpemU9OSksDQogICAgICAgICAgICAgICAgICAgYXhpcy50ZXh0LnkgPSBlbGVtZW50X3RleHQoc2l6ZT05LCBjb2xvdXI9ImJsYWNrIiwgaGp1c3Q9MC41LCBhbmdsZT05MCksIA0KICAgICAgICAgICAgICAgICAgIGF4aXMudGl0bGUueSA9IGVsZW1lbnRfYmxhbmsoKSwgbGVnZW5kLmtleS5zaXplID0gdW5pdCgwLjUsJ2NtJykpICsNCiAgICBnZ3Bsb3QyOjpsYWJzKHNpemU9cGFzdGUoIlByZWNpc2lvbiAoMS9TRSkiKSwgeD1wYXN0ZSgiRWZmZWN0IHNpemUgKElDQykiKSkNCg0KIyBGb3IgRmlndXJlIDINCg0KYSA8LSBnZ3Bsb3QoZGF0YSA9IG1hMl9tb2RfdGFibGUsIGFlcyh4ID0gZXN0aW1hdGUsIHkgPSAiT3ZlcmFsbCBtZWFuIikpICsgDQogICAgIHNjYWxlX3hfY29udGludW91cyhsaW1pdHMgPSBjKC0wLjM3LCAxKSwgYnJlYWtzID0gc2VxKC0wLjQsIDEsIGJ5ID0gMC4yKSwgbGFiZWxzID0gYygiLTAuNCIsICItMC4yIiwgIjAuMCIsICIwLjIiLCAiMC40IiwgIjAuNiIsICIwLjgiLCAiMS4wIikpICsNCiAgICBnZ2JlZXN3YXJtOjpnZW9tX3F1YXNpcmFuZG9tKGRhdGEgPSBtYTJfZGF0YSwgYWVzKHggPSB5aV9JQ0MsIHkgPSAiT3ZlcmFsbCBtZWFuIiwgc2l6ZSA9IHNjYWxlKSwgZmlsbD0iYmxhY2siLCBncm91cE9uWD1GQUxTRSwgYWxwaGEgPSAwLjIpICsNCiAgICBnZW9tX2Vycm9yYmFyaChhZXMoeG1pbiA9IGxvd2VyUFIsIHhtYXggPSB1cHBlclBSKSwgaGVpZ2h0ID0gMCwgc2hvdy5sZWdlbmQgPSBGLCBzaXplID0gMC41LCBhbHBoYSA9IDAuNikgKyAjIENJDQogICAgZ2VvbV9lcnJvcmJhcmgoYWVzKHhtaW4gPSBsb3dlckNMLCB4bWF4ID0gdXBwZXJDTCksIGhlaWdodCA9IDAsIHNob3cubGVnZW5kID0gRiwgc2l6ZSA9IDEuMikgKyANCiAgICBnZW9tX3ZsaW5lKHhpbnRlcmNlcHQgPSAwLCBsaW5ldHlwZSA9IDIsIGNvbG91ciA9ICJibGFjayIsIGFscGhhID0gMC41KSArDQogICAgZ2VvbV9wb2ludChmaWxsPSJibGFjayIsIHNpemUgPSAzLCBzaGFwZSA9IDIxKSArIA0KICAgIGFubm90YXRlKCd0ZXh0JywgeCA9IDAuOTMsIHkgPSAoc2VxKDEsIG1hMl9ncm91cF9ubywgMSkrMC4zNSksIGxhYmVsPSBwYXN0ZSgiaXRhbGljKGspPT0iLCBtYTJfbW9kX3RhYmxlJEspLCBwYXJzZT1UUlVFLCBoanVzdCA9ICJsZWZ0Iiwgc2l6ZT0zLjUpICsNCiAgICBnZ3Bsb3QyOjp0aGVtZV9idygpICsNCiAgICBnZ3Bsb3QyOjpndWlkZXMoZmlsbD0ibm9uZSIsIGNvbG91cj0ibm9uZSIpICsNCiAgICBnZ3Bsb3QyOjp0aGVtZShsZWdlbmQucG9zaXRpb24gPSBjKDEsMCksIGxlZ2VuZC5qdXN0aWZpY2F0aW9uID0gYygxLDApLCBsZWdlbmQudGl0bGUgPSBlbGVtZW50X3RleHQoc2l6ZT04KSwgDQogICAgICAgICAgICAgICAgICAgbGVnZW5kLnRleHQgPSBlbGVtZW50X3RleHQoc2l6ZT04KSwgbGVnZW5kLmtleSA9IGVsZW1lbnRfcmVjdChjb2xvdXIgPSBOQSwgZmlsbCA9IE5BKSwgDQogICAgICAgICAgICAgICAgICAgbGVnZW5kLmJhY2tncm91bmQgPSBlbGVtZW50X2JsYW5rKCksIGxlZ2VuZC5kaXJlY3Rpb24gPSAiaG9yaXpvbnRhbCIsIA0KICAgICAgICAgICAgICAgICAgIGF4aXMudGV4dC55ID0gZWxlbWVudF90ZXh0KHNpemU9OSwgY29sb3VyPSJibGFjayIsIGhqdXN0PTAuNSwgYW5nbGU9OTApLCANCiAgICAgICAgICAgICAgICAgICBheGlzLnRpdGxlID0gZWxlbWVudF9ibGFuaygpLCBsZWdlbmQua2V5LnNpemUgPSB1bml0KDAuNSwnY20nKSkgKw0KICAgIGdncGxvdDI6OmxhYnMoc2l6ZT1wYXN0ZSgiUHJlY2lzaW9uICgxL1NFKSIpKQ0KDQpmaWdfbWEyDQoNCmBgYA0KDQoqKkZpZ3VyZSAyYS4qKiBSZXBlYXRhYmlsaXR5IG9mIGF2aWFuIG1pZ3JhdGlvbiB0aW1pbmcgZm9yIGFsbCBlc3RpbWF0ZXMgdG9nZXRoZXIuIFRoZSBwbG90IHNob3dzIHRoZSBwaHlsb2dlbmV0aWMgbWV0YS1hbmFseXRpYyBtZWFuIHdpdGggOTUlIGNvbmZpZGVuY2UgaW50ZXJ2YWxzICh0aGljayBsaW5lcywgaW5kaWNhdGluZyB1bmNlcnRhaW50eSBhcm91bmQgdGhlIG92ZXJhbGwgZXN0aW1hdGUpIGFuZCA5NSUgcHJlZGljdGlvbiBpbnRlcnZhbHMgKHRoaW4gbGluZXMsIGluZGljYXRpbmcgdGhlIHBvc3NpYmxlIHJhbmdlIGZvciA5NSUgb2YgbmV3IChvciBzaW1wbHkgbm90IGluY2x1ZGVkKSBlZmZlY3Qgc2l6ZXMpLCBvYnNlcnZlZCBlZmZlY3Qgc2l6ZXMgKGJhY2stdHJhbnNmb3JtZWQgdG8gSUNDKSBzY2FsZWQgYnkgcHJlY2lzaW9uIChjaXJjbGVzKSBhbmQgayA9IG51bWJlciBvZiBlZmZlY3Qgc2l6ZXMuIFRoZSBncmFwaCB3YXMgY29uc3RydWN0ZWQgdXNpbmcgY29kZSBhZGFwdGVkIGZyb20gdGhlIGBvcmNoYXJkX3Bsb3RgIGZ1bmN0aW9uIGluIHRoZSBvcmNoYVJkIHBhY2thZ2UgW0BuYWthZ2F3YV9vcmNoYXJkXzIwMjFdLg0KDQojIyBNZXRhLXJlZ3Jlc3Npb24NCg0KV2UgcmFuIGEgdW5pdmFyaWF0ZSBtZXRhLXJlZ3Jlc3Npb24gbW9kZWwgZm9yIGVhY2ggb2YgdGhlIGZvbGxvd2luZyBtb2RlcmF0b3JzOiAxKSBgYW5udWFsX2V2ZW50YCwgMikgYG1ldGhvZGAsIDMpIGB0YXhhYCwgNCkgYHNleGAgYW5kIDUpIG51bWJlciBvZiBzYW1wbGVzIHBlciBpbmRpdmlkdWFsIChga2ApLg0KDQojIyMgVW5pdmFyaWF0ZSAodW5pLXByZWRpY3RvcikgYW5hbHlzZXMNCg0KV2UgZmlyc3QgY29uZHVjdGVkIGEgc2VyaWVzIG9mIG1ldGEtcmVncmVzc2lvbiBtb2RlbHMgd2l0aCBlYWNoIG9mIHRoZSBtb2RlcmF0b3JzIGludHJvZHVjZWQgYWJvdmUuIA0KDQojIyMjIFBlcmlvZCBvZiB0aGUgYW5udWFsIGN5Y2xlDQoNCmBgYHtyIG1ldGEtcmVncmVzc2lvbi1hbm51YWwtZXZlbnR9DQojIG1ldGEtcmVncmVzc2lvbjogbXV0aXBsZSBpbnRlcmNlcHRzDQptZXRhX3JlZ3Jlc3Npb24xIDwtIHJtYS5tdih5aSA9IFpyMiwgViA9IFZDViwgDQogICAgICAgICAgICAgICAgICAgICAgICAgICBtb2RzID0gfiBhbm51YWxfZXZlbnQsDQogICAgICAgICAgICAgICAgICAgICAgICAgICByYW5kb20gPSBsaXN0KH4xIHwgZXNfSUQsIA0KICAgICAgICAgICAgICAgICAgICAgICAgICAgICAgICAgICAgICAgICB+MSB8IHBhcGVyX0lELCANCiAgICAgICAgICAgICAgICAgICAgICAgICAgICAgICAgICAgICAgICAgfjEgfCBjb2hvcnRfSUQsIA0KICAgICAgICAgICAgICAgICAgICAgICAgICAgICAgICAgICAgICAgICB+MSB8IHNwZWNpZXNfSUQsIA0KICAgICAgICAgICAgICAgICAgICAgICAgICAgICAgICAgICAgICAgICB+MSB8IHBoeWxvZ2VueSksDQogICAgICAgICAgICAgICAgICAgICAgICAgICBSID0gbGlzdChwaHlsb2dlbnkgPSB2YXJjb3IpLCAjIGFkZGVkIGluIHBoeWxvZ25leQ0KICAgICAgICAgICAgICAgICAgICAgICAgICAgZGF0YSA9IGRmKQ0KDQptZXRhX3JlZ3Jlc3Npb24xYiA8LSBybWEubXYoeWkgPSBacjIsIFYgPSBWQ1YsIA0KICAgICAgICAgICAgICAgICAgICAgICAgICAgIG1vZHMgPSB+IHJlbGV2ZWwoYW5udWFsX2V2ZW50LCByZWYgPSAiRGVwYXJ0X2JyZWVkIiksDQogICAgICAgICAgICAgICAgICAgICAgICAgICAgcmFuZG9tID0gbGlzdCh+MSB8IGVzX0lELCANCiAgICAgICAgICAgICAgICAgICAgICAgICAgICAgICAgICAgICAgICAgIH4xIHwgcGFwZXJfSUQsIA0KICAgICAgICAgICAgICAgICAgICAgICAgICAgICAgICAgICAgICAgICAgfjEgfCBjb2hvcnRfSUQsIA0KICAgICAgICAgICAgICAgICAgICAgICAgICAgICAgICAgICAgICAgICAgfjEgfCBzcGVjaWVzX0lELCANCiAgICAgICAgICAgICAgICAgICAgICAgICAgICAgICAgICAgICAgICAgIH4xIHwgcGh5bG9nZW55KSwNCiAgICAgICAgICAgICAgICAgICAgICAgICAgICBSID0gbGlzdChwaHlsb2dlbnkgPSB2YXJjb3IpLCAjIGFkZGVkIGluIHBoeWxvZ25leQ0KICAgICAgICAgICAgICAgICAgICAgICAgICAgIGRhdGEgPSBkZikNCg0KDQptZXRhX3JlZ3Jlc3Npb24xYyA8LSBybWEubXYoeWkgPSBacjIsIFYgPSBWQ1YsIA0KICAgICAgICAgICAgICAgICAgICAgICAgICAgIG1vZHMgPSB+IHJlbGV2ZWwoYW5udWFsX2V2ZW50LCByZWYgPSAiTm9uYnJlZWRfZGVwYXJ0IiksDQogICAgICAgICAgICAgICAgICAgICAgICAgICAgcmFuZG9tID0gbGlzdCh+MSB8IGVzX0lELCANCiAgICAgICAgICAgICAgICAgICAgICAgICAgICAgICAgICAgICAgICAgIH4xIHwgcGFwZXJfSUQsIA0KICAgICAgICAgICAgICAgICAgICAgICAgICAgICAgICAgICAgICAgICAgfjEgfCBjb2hvcnRfSUQsIA0KICAgICAgICAgICAgICAgICAgICAgICAgICAgICAgICAgICAgICAgICAgfjEgfCBzcGVjaWVzX0lELCANCiAgICAgICAgICAgICAgICAgICAgICAgICAgICAgICAgICAgICAgICAgIH4xIHwgcGh5bG9nZW55KSwNCiAgICAgICAgICAgICAgICAgICAgICAgICAgICBSID0gbGlzdChwaHlsb2dlbnkgPSB2YXJjb3IpLCAjIGFkZGVkIGluIHBoeWxvZ25leQ0KICAgICAgICAgICAgICAgICAgICAgICAgICAgIGRhdGEgPSBkZikNCg0KIyBtZXRhLXJlZ3Jlc3Npb246IGNvbnRyYXN0IChmb3Igb3JjaGFyZCBwbG90KQ0KbWV0YV9yZWdyZXNzaW9uMWQgPC0gcm1hLm12KHlpID0gWnIyLCBWID0gVkNWLCANCiAgICAgICAgICAgICAgICAgICAgICAgICAgICBtb2RzID0gfiBhbm51YWxfZXZlbnQgLTEsDQogICAgICAgICAgICAgICAgICAgICAgICAgICAgcmFuZG9tID0gbGlzdCh+MSB8IGVzX0lELCANCiAgICAgICAgICAgICAgICAgICAgICAgICAgICAgICAgICAgICAgICAgIH4xIHwgcGFwZXJfSUQsIA0KICAgICAgICAgICAgICAgICAgICAgICAgICAgICAgICAgICAgICAgICAgfjEgfCBjb2hvcnRfSUQsIA0KICAgICAgICAgICAgICAgICAgICAgICAgICAgICAgICAgICAgICAgICAgfjEgfCBzcGVjaWVzX0lELCANCiAgICAgICAgICAgICAgICAgICAgICAgICAgICAgICAgICAgICAgICAgIH4xIHwgcGh5bG9nZW55KSwNCiAgICAgICAgICAgICAgICAgICAgICAgICAgICBSID0gbGlzdChwaHlsb2dlbnkgPSB2YXJjb3IpLCAjIGFkZGVkIGluIHBoeWxvZ25leQ0KICAgICAgICAgICAgICAgICAgICAgICAgICAgIGRhdGEgPSBkZikNCmBgYA0KDQojIyMjIFRhYmxlIFM0DQpSZWdyZXNzaW9uIGNvZWZmaWNpZW50cyAoRXN0aW1hdGUpIGFuZCA5NSUgY29uZmlkZW5jZSBpbnRlcnZhbHMgKENJcykgYm90aCBpbiBaciBhbmQgYmFjay10cmFuc2Zvcm1lZCB0byBJQ0MgZnJvbSB0aGUgbWV0YS1yZWdyZXNzaW9uIHdpdGggYGFubnVhbF9ldmVudGAuIE5vdGUgdGhhdCBgbXVgIG1lYW5zIHRoZSBncm91cCBtZWFuIHdoaWxlIGBiZXRhYCByZXByZXNlbnRzIHRoZSBjb250cmFzdCBiZXR3ZWVuIHR3byBncm91cHMgaW4gdGhlIFVuaXQgY29sdW1uLiAqUjxzdXA+Mjwvc3VwPjxzdWI+bWFyZ2luYWw8L3N1Yj4qID0gYHIgcm91bmQob3JjaGFSZDo6cjJfbWwobWV0YV9yZWdyZXNzaW9uMSlbWzFdXSoxMDAsMSlgJS4NCg0KYGBge3IgYW5udWFsLWV2ZW50LXRhYmxlfQ0KIyBnZXR0aW5nIG1hcmdpbmFsIFIyDQpyMl9tZXRhX3JlZ3Jlc3Npb24xIDwtIHIyX21sKG1ldGFfcmVncmVzc2lvbjEpDQoNCiMgZ2V0dGluZyBlc3RpbWF0ZXMNCiMgaW5jbHVkaW5nIGJhY2stdHJhbnNmb3JtYXRpb24gdG8gSUNDDQoNCm1yMSA8LSBtb2RfcmVzdWx0cyhtZXRhX3JlZ3Jlc3Npb24xZCwgbW9kPSJhbm51YWxfZXZlbnQiKQ0KbXIxX21vZF90YWJsZSA8LSBtcjEkbW9kX3RhYmxlDQoNCm1yMV9kYXRhIDwtIG1yMSRkYXRhDQoNCiMgY2FsY3VsYXRlIGsgZm9yIGVhY2ggbWV0aG9kIHNlcGFyYXRlbHkNCg0KIyBkZiAlPiUgZ3JvdXBfYnkoYW5udWFsX2V2ZW50KSAlPiUgc3VtbWFyaXNlKG1lYW4oaykpDQoNCm1yMV9kYXRhIDwtIG1yMV9kYXRhICU+JSBtdXRhdGUoayA9IGNhc2Vfd2hlbihtb2RlcmF0b3IgPT0gIkFycml2YWxfYnJlZWQiIH4gMi41NSwgDQogICAgICAgICAgICAgICAgICAgICAgICAgICAgICAgICAgICAgICAgICAgICAgbW9kZXJhdG9yID09ICJEZXBhcnRfYnJlZWQiIH4gMi40NSwNCiAgICAgICAgICAgICAgICAgICAgICAgICAgICAgICAgICAgICAgICAgICAgICBtb2RlcmF0b3IgPT0gIk5vbmJyZWVkX2Fycml2YWwiIH4gMy4yMCwNCiAgICAgICAgICAgICAgICAgICAgICAgICAgICAgICAgICAgICAgICAgICAgICBtb2RlcmF0b3IgPT0gIk5vbmJyZWVkX2RlcGFydCIgfiAyLjk0KSkNCg0KbXIxX2RhdGEkeWlfSUNDIDwtIFpyX3RvX0lDQyhtcjFfZGF0YSR5aSwgbXIxX2RhdGEkaykNCg0KbXIxX21vZF90YWJsZSRrIDwtIGMoMi41NSwgMi40NSwgMy4yMCwgMi45NCkNCg0KZm9yKGkgaW4gbmFtZXMobXIxX21vZF90YWJsZSlbMjo2XSl7DQogIA0KICBtcjFfbW9kX3RhYmxlW2ldIDwtIFpyX3RvX0lDQyhtcjFfbW9kX3RhYmxlW2ldLCBtcjFfbW9kX3RhYmxlJGspDQogIA0KfQ0KDQojIGNyZWF0aW5nIGEgdGFibGUNCnRpYmJsZSgNCiAgYEZpeGVkIGVmZmVjdGAgPSBjKHJlcChhcy5jaGFyYWN0ZXIobXIxX21vZF90YWJsZSRuYW1lKSwyKSwgY29udF9nZW4obXIxX21vZF90YWJsZSRuYW1lKSksDQogIGBVbml0YCA9IGMocmVwKGMoIlpyIChtdSkiLCAiSUNDIChtdSkiKSxlYWNoID0gNCksIHJlcCgiWnIgKGJldGEpIiwgNikpLA0KICBgRXN0aW1hdGVgID0gYyhtZXRhX3JlZ3Jlc3Npb24xZCRiLCBtcjFfbW9kX3RhYmxlJGVzdGltYXRlLCANCiAgICAgICAgICAgICAgICAgbWV0YV9yZWdyZXNzaW9uMSRiWy0xXSwgbWV0YV9yZWdyZXNzaW9uMWIkYlstKDE6MildLCBtZXRhX3JlZ3Jlc3Npb24xYyRiWy0oMTozKV0pLA0KICBgTG93ZXIgQ0kgWzAuMDI1XWAgPSBjKG1ldGFfcmVncmVzc2lvbjFkJGNpLmxiLCBtcjFfbW9kX3RhYmxlJGxvd2VyQ0wsDQogICAgICAgICAgICAgICAgICAgICAgICAgbWV0YV9yZWdyZXNzaW9uMSRjaS5sYlstMV0sIG1ldGFfcmVncmVzc2lvbjFiJGNpLmxiWy0oMToyKV0sIG1ldGFfcmVncmVzc2lvbjFjJGNpLmxiWy0oMTozKV0pLA0KICBgVXBwZXIgQ0kgIFswLjk3NV1gID0gYyhtZXRhX3JlZ3Jlc3Npb24xZCRjaS51YiwgbXIxX21vZF90YWJsZSR1cHBlckNMLA0KICAgICAgICAgICAgICAgICAgICAgICAgIG1ldGFfcmVncmVzc2lvbjEkY2kudWJbLTFdLCBtZXRhX3JlZ3Jlc3Npb24xYiRjaS51YlstKDE6MildLCBtZXRhX3JlZ3Jlc3Npb24xYyRjaS51YlstKDE6MyldKSkgLT4gdF9hbm51YWxfZXZlbnQNCiAjIGBSMmAgPSBjKHIyX21ldGFfcmVncmVzc2lvbjFbMV0sIHJlcChOQSwxMykpKSAtPiANCg0KdF9hbm51YWxfZXZlbnQgJT4lIGthYmxlKCJodG1sIiwgZGlnaXRzID0gMykgJT4lDQogIGthYmxlX3N0eWxpbmcoInN0cmlwZWQiLCBwb3NpdGlvbiA9ICJsZWZ0IikNCiAgICAgICAgICAgICAgIA0KYGBgDQoNCiMjIyMgRmlndXJlIDJiIA0KDQpgYGB7ciBmaWd1cmUyYiwgZmlnLndpZHRoPTcsIGZpZy5oZWlnaHQ9NH0NCiMgT3JjaGFyZCBwbG90IGZvciBhbm51YWwgZXZlbnQgd2l0aCBJQ0MNCg0KbXIxX2RhdGEkbW9kZXJhdG9yIDwtIGZhY3RvcihtcjFfZGF0YSRtb2RlcmF0b3IsIGxldmVscyA9IG1yMV9tb2RfdGFibGUkbmFtZSwgbGFiZWxzID0gbXIxX21vZF90YWJsZSRuYW1lKQ0KbXIxX2RhdGEkc2NhbGUgPC0gKDEvc3FydChtcjFfZGF0YVssInZpIl0pKQ0KbXIxX21vZF90YWJsZSRLIDwtIGFzLnZlY3RvcihieShtcjFfZGF0YSwgbXIxX2RhdGFbLCJtb2RlcmF0b3IiXSwgZnVuY3Rpb24oeCkgbGVuZ3RoKHhbLCJ5aSJdKSkpDQptcjFfZ3JvdXBfbm8gPC0gbnJvdyhtcjFfbW9kX3RhYmxlKQ0KDQppbWFnZV9BQmVnZyA8LSByZWFkUE5HKGhlcmUoImltYWdlcy9BQl9lZ2dzX2ljb24ucG5nIikpDQppbWFnZV9BTkIgPC0gcmVhZFBORyhoZXJlKCJpbWFnZXMvQU5CX2ljb24ucG5nIikpDQppbWFnZV9EQmNoaWNrIDwtIHJlYWRQTkcoaGVyZSgiaW1hZ2VzL0RCX2NoaWNrc19pY29uLnBuZyIpKQ0KaW1hZ2VfRE5CIDwtIHJlYWRQTkcoaGVyZSgiaW1hZ2VzL0ROQl9pY29uLnBuZyIpKQ0KDQojIGNyZWF0aW5nIGFuIG9yY2hhcmQgcGxvdA0KDQojIGZpZ19hbm51YWxfZXZlbnQgPC0gZ2dwbG90KGRhdGEgPSBtcjFfbW9kX3RhYmxlLCBhZXMoeCA9IGVzdGltYXRlLCB5ID0gbmFtZSkpICsgDQojICAgICBzY2FsZV94X2NvbnRpbnVvdXMobGltaXRzID0gYygtMC40LCAxKSwgYnJlYWtzID0gc2VxKC0wLjQsIDEsIGJ5ID0gMC4yKSwgbGFiZWxzID0gYygiLTAuNCIsICItMC4yIiwgIjAuMCIsICIwLjIiLCAjICIwLjQiLCAiMC42IiwgIjAuOCIsICIxLjAiKSkgKyANCiMgICAgIGdnYmVlc3dhcm06Omdlb21fcXVhc2lyYW5kb20oZGF0YSA9IG1yMV9kYXRhLCBhZXMoeCA9IHlpX0lDQywgeSA9IG1vZGVyYXRvciwgc2l6ZSA9IHNjYWxlLCBjb2xvdXI9bW9kZXJhdG9yKSwgZ3JvdXBPblg9RkFMU0UsICMgYWxwaGEgPSAwLjQpICsNCiMgICAgIGdlb21fZXJyb3JiYXJoKGFlcyh4bWluID0gbG93ZXJQUiwgeG1heCA9IHVwcGVyUFIpLCBoZWlnaHQgPSAwLCBzaG93LmxlZ2VuZCA9IEYsIHNpemUgPSAwLjUsIGFscGhhID0gMC42KSArICMgQ0kNCiMgICAgIGdlb21fZXJyb3JiYXJoKGFlcyh4bWluID0gbG93ZXJDTCwgeG1heCA9IHVwcGVyQ0wpLCBoZWlnaHQgPSAwLCBzaG93LmxlZ2VuZCA9IEYsIHNpemUgPSAxLjIpICsgDQojICAgICBnZW9tX3ZsaW5lKHhpbnRlcmNlcHQgPSAwLCBsaW5ldHlwZSA9IDIsIGNvbG91ciA9ICJibGFjayIsIGFscGhhID0gMC41KSArDQojICAgICBnZW9tX3BvaW50KGFlcyhmaWxsPW5hbWUpLCBzaXplID0gMywgc2hhcGUgPSAyMSkgKyANCiMgICAgIGFubm90YXRlKCd0ZXh0JywgeCA9IDAuOTMsIHkgPSAoc2VxKDEsIG1yMV9ncm91cF9ubywgMSkrMC4zNSksIGxhYmVsPSBwYXN0ZSgiaXRhbGljKGspPT0iLCBtcjFfbW9kX3RhYmxlJEspLCBwYXJzZT1UUlVFLCBoanVzdCAjID0gImxlZnQiLCBzaXplPTMuNSkgKw0KIyAgICAgZ2dwbG90Mjo6dGhlbWVfYncoKSArDQojICAgICBnZ3Bsb3QyOjpndWlkZXMoZmlsbD0ibm9uZSIsIGNvbG91cj0ibm9uZSIpICsNCiMgICAgIGdncGxvdDI6OnRoZW1lKGF4aXMudGV4dC55ID0gZWxlbWVudF90ZXh0KHNpemU9OSwgY29sb3VyPSJibGFjayIsIGhqdXN0PTAuNSwgYW5nbGU9OTApLA0KIyAgICAgICAgICAgICAgICAgICAgbGVnZW5kLnBvc2l0aW9uID0gIm5vbmUiLCBheGlzLnRpdGxlLnkgPSBlbGVtZW50X2JsYW5rKCksIGF4aXMudGV4dC54ID0gZWxlbWVudF90ZXh0KHNpemU9OSkpICsNCiMgICAgIHNjYWxlX3lfZGlzY3JldGUobGFiZWxzPWMoIkFycml2YWxfYnJlZWQiID0gIkFycml2ZSIsICJEZXBhcnRfYnJlZWQiID0gIkRlcGFydCIsICJOb25icmVlZF9hcnJpdmFsIiA9ICJBcnJpdmUiLCAjICJOb25icmVlZF9kZXBhcnQiID0gIkRlcGFydCIpKSArDQojIAkgICAgICBzY2FsZV9maWxsX21hbnVhbCh2YWx1ZXM9Y2JwbCkgKw0KIyAJICAgICAgc2NhbGVfY29sb3VyX21hbnVhbCh2YWx1ZXM9Y2JwbCkgKw0KIyAgICAgZ2dwbG90Mjo6bGFicyhzaXplPXBhc3RlKCJQcmVjaXNpb24gKDEvU0UpIiksIHg9cGFzdGUoIkVmZmVjdCBzaXplIChJQ0MpIikpICsNCiMgICAgIGFubm90YXRpb25fY3VzdG9tKGdyaWQ6OnJhc3Rlckdyb2IoaW1hZ2VfRE5CKSwgeG1pbiA9IC0wLjQ1LCB4bWF4ID0gLTAuMjUsIHltaW4gPSAzLjcsIHltYXggPSA0LjMpICsNCiMgICAgIGFubm90YXRpb25fY3VzdG9tKGdyaWQ6OnJhc3Rlckdyb2IoaW1hZ2VfQU5CKSwgeG1pbiA9IC0wLjQ1LCB4bWF4ID0gLTAuMjUsIHltaW4gPSAyLjcsIHltYXggPSAzLjMpICsNCiMgICAgIGFubm90YXRpb25fY3VzdG9tKGdyaWQ6OnJhc3Rlckdyb2IoaW1hZ2VfREJjaGljayksIHhtaW4gPSAtMC40NSwgeG1heCA9IC0wLjI1LCB5bWluID0gMS43LCB5bWF4ID0gMi4zKSArDQojICAgICBhbm5vdGF0aW9uX2N1c3RvbShncmlkOjpyYXN0ZXJHcm9iKGltYWdlX0FCZWdnKSwgeG1pbiA9IC0wLjQ1LCB4bWF4ID0gLTAuMjUsIHltaW4gPSAwLjcsIHltYXggPSAxLjMpDQojIA0KIyAjIGZvciBGaWd1cmUgMg0KIyANCiMgYiA8LSBnZ3Bsb3QoZGF0YSA9IG1yMV9tb2RfdGFibGUsIGFlcyh4ID0gZXN0aW1hdGUsIHkgPSBuYW1lKSkgKyANCiMgICAgIHNjYWxlX3hfY29udGludW91cyhsaW1pdHMgPSBjKC0wLjM3LCAxKSwgYnJlYWtzID0gc2VxKC0wLjQsIDEsIGJ5ID0gMC4yKSwgbGFiZWxzID0gYygiLTAuNCIsICItMC4yIiwgIjAuMCIsICIwLjIiLCAiMC40IiwgIjAuNiIsICIwLjgiLCAiMS4wIikpICsgDQojICAgICBnZ2JlZXN3YXJtOjpnZW9tX3F1YXNpcmFuZG9tKGRhdGEgPSBtcjFfZGF0YSwgYWVzKHggPSB5aV9JQ0MsIHkgPSBtb2RlcmF0b3IsIHNpemUgPSBzY2FsZSwgY29sb3VyPW1vZGVyYXRvciksIGdyb3VwT25YPUZBTFNFLCBhbHBoYSA9IDAuNCkgKw0KIyAgICAgZ2VvbV9lcnJvcmJhcmgoYWVzKHhtaW4gPSBsb3dlclBSLCB4bWF4ID0gdXBwZXJQUiksIGhlaWdodCA9IDAsIHNob3cubGVnZW5kID0gRiwgc2l6ZSA9IDAuNSwgYWxwaGEgPSAwLjYpICsgIyBDSQ0KIyAgICAgZ2VvbV9lcnJvcmJhcmgoYWVzKHhtaW4gPSBsb3dlckNMLCB4bWF4ID0gdXBwZXJDTCksIGhlaWdodCA9IDAsIHNob3cubGVnZW5kID0gRiwgc2l6ZSA9IDEuMikgKyANCiMgICAgIGdlb21fdmxpbmUoeGludGVyY2VwdCA9IDAsIGxpbmV0eXBlID0gMiwgY29sb3VyID0gImJsYWNrIiwgYWxwaGEgPSAwLjUpICsNCiMgICAgIGdlb21fcG9pbnQoYWVzKGZpbGw9bmFtZSksIHNpemUgPSAzLCBzaGFwZSA9IDIxKSArIA0KIyAgICAgYW5ub3RhdGUoJ3RleHQnLCB4ID0gMC45MywgeSA9IChzZXEoMSwgbXIxX2dyb3VwX25vLCAxKSswLjM1KSwgbGFiZWw9IHBhc3RlKCJpdGFsaWMoayk9PSIsIG1yMV9tb2RfdGFibGUkSyksIHBhcnNlPVRSVUUsIGhqdXN0ID0gImxlZnQiLCBzaXplPTMuNSkgKw0KIyAgICAgZ2dwbG90Mjo6dGhlbWVfYncoKSArDQojICAgICBnZ3Bsb3QyOjpndWlkZXMoZmlsbD0ibm9uZSIsIGNvbG91cj0ibm9uZSIpICsNCiMgICAgIGdncGxvdDI6OnRoZW1lKGF4aXMudGV4dC55ID0gZWxlbWVudF90ZXh0KHNpemU9OSwgY29sb3VyPSJibGFjayIsIGhqdXN0PTAuNSwgYW5nbGU9OTApLA0KIyAgICAgICAgICAgICAgICAgICAgbGVnZW5kLnBvc2l0aW9uID0gIm5vbmUiLCBheGlzLnRpdGxlID0gZWxlbWVudF9ibGFuaygpKSArDQojICAgICBzY2FsZV95X2Rpc2NyZXRlKGxhYmVscz1jKCJBcnJpdmFsX2JyZWVkIiA9ICJBcnJpdmUiLCAiRGVwYXJ0X2JyZWVkIiA9ICJEZXBhcnQiLCAiTm9uYnJlZWRfYXJyaXZhbCIgPSAiQXJyaXZlIiwgIk5vbmJyZWVkX2RlcGFydCIgPSAiRGVwYXJ0IikpICsNCiMgCSAgICAgIHNjYWxlX2ZpbGxfbWFudWFsKHZhbHVlcz1jYnBsKSArDQojIAkgICAgICBzY2FsZV9jb2xvdXJfbWFudWFsKHZhbHVlcz1jYnBsKSArDQojICAgICBhbm5vdGF0aW9uX2N1c3RvbShncmlkOjpyYXN0ZXJHcm9iKGltYWdlX0ROQiksIHhtaW4gPSAtMC40NSwgeG1heCA9IC0wLjI1LCB5bWluID0gMy43LCB5bWF4ID0gNC4zKSArDQojICAgICBhbm5vdGF0aW9uX2N1c3RvbShncmlkOjpyYXN0ZXJHcm9iKGltYWdlX0FOQiksIHhtaW4gPSAtMC40NSwgeG1heCA9IC0wLjI1LCB5bWluID0gMi43LCB5bWF4ID0gMy4zKSArDQojICAgICBhbm5vdGF0aW9uX2N1c3RvbShncmlkOjpyYXN0ZXJHcm9iKGltYWdlX0RCY2hpY2spLCB4bWluID0gLTAuNDUsIHhtYXggPSAtMC4yNSwgeW1pbiA9IDEuNywgeW1heCA9IDIuMykgKw0KIyAgICAgYW5ub3RhdGlvbl9jdXN0b20oZ3JpZDo6cmFzdGVyR3JvYihpbWFnZV9BQmVnZyksIHhtaW4gPSAtMC40NSwgeG1heCA9IC0wLjI1LCB5bWluID0gMC43LCB5bWF4ID0gMS4zKQ0KDQojIGZpZ19hbm51YWxfZXZlbnQNCg0KYGBgDQoNCiFbXShDOi9Vc2Vycy9yc3QxOHZ6dS9PbmVEcml2ZSAtIFVuaXZlcnNpdHkgb2YgRWFzdCBBbmdsaWEvUGhEL0xpdC4gcmV2aWV3L01ldGEtYW5hbHlzaXMvQXZpYW5fbWlncmF0aW9uX21ldGEtYW5hbHlzaXMvZmlncy9GaWcyYl96ZXJvX2Zvcl9SbWQucG5nKXt3aWR0aD04NSV9DQoNCioqRmlndXJlIDJiLioqIFJlcGVhdGFiaWxpdHkgb2YgYXZpYW4gbWlncmF0aW9uIHRpbWluZyBmb3IgYW5udWFsIG1pZ3JhdGlvbiBldmVudHMuIFRoZSBwbG90IHNob3dzIHRoZSBncm91cC13aXNlIG1lYW5zIHdpdGggOTUlIGNvbmZpZGVuY2UgaW50ZXJ2YWxzICh0aGljayBsaW5lcykgYW5kIDk1JSBwcmVkaWN0aW9uIGludGVydmFscyAodGhpbiBsaW5lcyksIG9ic2VydmVkIGVmZmVjdCBzaXplcyAoYmFjay10cmFuc2Zvcm1lZCB0byBJQ0MpIHNjYWxlZCBieSBwcmVjaXNpb24gKGNpcmNsZXMpIGFuZCBrID0gbnVtYmVyIG9mIGVmZmVjdCBzaXplcy4gTm90ZSB0aGlzIHBsb3Qgd2FzIGVkaXRlZCBpbiBBZmZpbml0eSBEZXNpZ25lciBhZnRlciBjb25zdHJ1Y3Rpb24gaW4gUi4NCg0KIyMjIyBUaGUgZWZmZWN0IG9mIHRyYWNraW5nIG1ldGhvZA0KDQpgYGB7ciBtZXRhLXJlZ3Jlc3Npb24tbWV0aG9kfQ0KbWV0YV9yZWdyZXNzaW9uMiA8LSBybWEubXYoeWkgPSBacjIsIFYgPSBWQ1YsIA0KICAgICAgICAgICAgICAgICAgICAgIG1vZHMgPSB+IG1ldGhvZCwNCiAgICAgICAgICAgICAgICAgICAgICByYW5kb20gPSBsaXN0KH4xIHwgZXNfSUQsIA0KICAgICAgICAgICAgICAgICAgICAgICAgICAgICAgICAgICAgfjEgfCBwYXBlcl9JRCwgDQogICAgICAgICAgICAgICAgICAgICAgICAgICAgICAgICAgICB+MSB8IGNvaG9ydF9JRCwgDQogICAgICAgICAgICAgICAgICAgICAgICAgICAgICAgICAgICB+MSB8IHNwZWNpZXNfSUQsIA0KICAgICAgICAgICAgICAgICAgICAgICAgICAgICAgICAgICAgfjEgfCBwaHlsb2dlbnkpLA0KICAgICAgICAgICAgICAgICAgICAgIFIgPSBsaXN0KHBoeWxvZ2VueSA9IHZhcmNvciksDQogICAgICAgICAgICAgICAgICAgICAgZGF0YSA9IGRmKQ0KDQojIHJlb3JkZXJpbmcNCiMgZGYkbWV0aG9kIDwtIGZhY3RvcihkZiRtZXRob2QsIGxldmVscyA9IGMoIkNvbnZlbnRpb25hbCIsICJHTFMiLCAiU2F0ZWxsaXRlIikpDQoNCm1ldGFfcmVncmVzc2lvbjJiIDwtIHJtYS5tdih5aSA9IFpyMiwgViA9IFZDViwgDQogICAgICAgICAgICAgICAgICAgICAgICAgICAgbW9kcyA9IH4gcmVsZXZlbChtZXRob2QsIHJlZiA9ICJHTFMiKSwNCiAgICAgICAgICAgICAgICAgICAgICAgICAgICByYW5kb20gPSBsaXN0KH4xIHwgZXNfSUQsIA0KICAgICAgICAgICAgICAgICAgICAgICAgICAgICAgICAgICAgICAgICAgfjEgfCBwYXBlcl9JRCwgDQogICAgICAgICAgICAgICAgICAgICAgICAgICAgICAgICAgICAgICAgICB+MSB8IGNvaG9ydF9JRCwgDQogICAgICAgICAgICAgICAgICAgICAgICAgICAgICAgICAgICAgICAgICB+MSB8IHNwZWNpZXNfSUQsIA0KICAgICAgICAgICAgICAgICAgICAgICAgICAgICAgICAgICAgICAgICAgfjEgfCBwaHlsb2dlbnkpLA0KICAgICAgICAgICAgICAgICAgICAgICAgICAgIFIgPSBsaXN0KHBoeWxvZ2VueSA9IHZhcmNvciksDQogICAgICAgICAgICAgICAgICAgICAgICAgICAgZGF0YSA9IGRmKQ0KDQojIE9yY2hhcmQgcGxvdCAtIG5lZWQgbWV0YS1yZWdyZXNzaW9uIHdpdGhvdXQgaW50ZXJjZXB0DQptZXRhX3JlZ3Jlc3Npb24yYyA8LSBybWEubXYoeWkgPSBacjIsIFYgPSBWQ1YsIA0KICAgICAgICAgICAgICAgICAgICAgICAgICAgIG1vZHMgPSB+IG1ldGhvZCAtMSwNCiAgICAgICAgICAgICAgICAgICAgICAgICAgICByYW5kb20gPSBsaXN0KH4xIHwgZXNfSUQsIA0KICAgICAgICAgICAgICAgICAgICAgICAgICAgICAgICAgICAgICAgICAgfjEgfCBwYXBlcl9JRCwgDQogICAgICAgICAgICAgICAgICAgICAgICAgICAgICAgICAgICAgICAgICB+MSB8IGNvaG9ydF9JRCwgDQogICAgICAgICAgICAgICAgICAgICAgICAgICAgICAgICAgICAgICAgICB+MSB8IHNwZWNpZXNfSUQsIA0KICAgICAgICAgICAgICAgICAgICAgICAgICAgICAgICAgICAgICAgICAgfjEgfCBwaHlsb2dlbnkpLA0KICAgICAgICAgICAgICAgICAgICAgICAgICAgIFIgPSBsaXN0KHBoeWxvZ2VueSA9IHZhcmNvciksDQogICAgICAgICAgICAgICAgICAgICAgICAgICAgZGF0YSA9IGRmKQ0KDQpgYGANCg0KIyMjIyBUYWJsZSBTNQ0KUmVncmVzc2lvbiBjb2VmZmljaWVudHMgKEVzdGltYXRlKSBhbmQgOTUlIGNvbmZpZGVuY2UgaW50ZXJ2YWxzIChDSXMpIGJvdGggaW4gWnIgYW5kIGJhY2stdHJhbnNmb3JtZWQgdG8gSUNDIGZyb20gdGhlIG1ldGEtcmVncmVzc2lvbiB3aXRoIGBtZXRob2RgLiBOb3RlIHRoYXQgYG11YCBtZWFucyB0aGUgZ3JvdXAgbWVhbiB3aGlsZSBgYmV0YWAgcmVwcmVzZW50cyB0aGUgY29udHJhc3QgYmV0d2VlbiB0d28gZ3JvdXBzIGluIHRoZSBVbml0IGNvbHVtbi4gKlI8c3VwPjI8L3N1cD48c3ViPm1hcmdpbmFsPC9zdWI+KiA9IGByIHJvdW5kKG9yY2hhUmQ6OnIyX21sKG1ldGFfcmVncmVzc2lvbjIpW1sxXV0qMTAwLDEpYCUuDQoNCmBgYHtyIG1ldGhvZC10YWJsZX0NCiMgZ2V0dGluZyBtYXJnaW5hbCBSMg0KcjJfbWV0YV9yZWdyZXNzaW9uMiA8LSByMl9tbChtZXRhX3JlZ3Jlc3Npb24yKQ0KDQojIGdldHRpbmcgZXN0aW1hdGVzDQojIGluY2x1ZGluZyBiYWNrLXRyYW5zZm9ybWF0aW9uIHRvIElDQw0KDQptcjIgPC0gbW9kX3Jlc3VsdHMobWV0YV9yZWdyZXNzaW9uMmMsIG1vZD0ibWV0aG9kIikNCm1yMl9tb2RfdGFibGUgPC0gbXIyJG1vZF90YWJsZQ0KDQptcjJfZGF0YSA8LSBtcjIkZGF0YQ0KDQojIGNhbGN1bGF0ZSBrIGZvciBlYWNoIG1ldGhvZCBzZXBhcmF0ZWx5DQoNCiNkZiAlPiUgZ3JvdXBfYnkobWV0aG9kKSAlPiUgc3VtbWFyaXNlKG1lYW4oaykpDQoNCg0KbXIyX2RhdGEgPC0gbXIyX2RhdGEgJT4lIG11dGF0ZShrID0gY2FzZV93aGVuKG1vZGVyYXRvciA9PSAiR0xTIiB+IDIuMjAsIA0KICAgICAgICAgICAgICAgICAgICAgICAgICAgICAgICAgICAgICAgICAgICAgIG1vZGVyYXRvciA9PSAiQ29udmVudGlvbmFsIiB+IDMuMTMsDQogICAgICAgICAgICAgICAgICAgICAgICAgICAgICAgICAgICAgICAgICAgICAgbW9kZXJhdG9yID09ICJTYXRlbGxpdGUiIH4gMy4yOCkpDQoNCm1yMl9kYXRhJHlpX0lDQyA8LSBacl90b19JQ0MobXIyX2RhdGEkeWksIG1yMl9kYXRhJGspDQoNCm1yMl9tb2RfdGFibGUkayA8LSBjKDMuMTMsIDIuMjAsIDMuMjgpDQoNCmZvcihpIGluIG5hbWVzKG1yMl9tb2RfdGFibGUpWzI6Nl0pew0KICANCiAgbXIyX21vZF90YWJsZVtpXSA8LSBacl90b19JQ0MobXIyX21vZF90YWJsZVtpXSwgbXIyX21vZF90YWJsZSRrKQ0KICANCn0NCg0KIyBjcmVhdGluZyBhIHRhYmxlDQp0aWJibGUoDQogIGBGaXhlZCBlZmZlY3RgID0gYyhyZXAoYXMuY2hhcmFjdGVyKG1yMl9tb2RfdGFibGUkbmFtZSksMiksIGNvbnRfZ2VuKG1yMl9tb2RfdGFibGUkbmFtZSkpLA0KICBgVW5pdGAgPSBjKHJlcChjKCJaciAobXUpIiwgIklDQyAobXUpIiksZWFjaCA9IDMpLCByZXAoIlpyIChiZXRhKSIsIDMpKSwNCiAgYEVzdGltYXRlYCA9IGMobWV0YV9yZWdyZXNzaW9uMmMkYiwgbXIyX21vZF90YWJsZSRlc3RpbWF0ZSwgDQogICAgICAgICAgICAgICAgIG1ldGFfcmVncmVzc2lvbjIkYlstMV0sIG1ldGFfcmVncmVzc2lvbjJiJGJbLSgxOjIpXSksDQogIGBMb3dlciBDSSBbMC4wMjVdYCA9IGMobWV0YV9yZWdyZXNzaW9uMmMkY2kubGIsIG1yMl9tb2RfdGFibGUkbG93ZXJDTCwNCiAgICAgICAgICAgICAgICAgICAgICAgICBtZXRhX3JlZ3Jlc3Npb24yJGNpLmxiWy0xXSwgbWV0YV9yZWdyZXNzaW9uMmIkY2kubGJbLSgxOjIpXSksDQogIGBVcHBlciBDSSAgWzAuOTc1XWAgPSBjKG1ldGFfcmVncmVzc2lvbjJjJGNpLnViLCBtcjJfbW9kX3RhYmxlJHVwcGVyQ0wsDQogICAgICAgICAgICAgICAgICAgICAgICAgbWV0YV9yZWdyZXNzaW9uMiRjaS51YlstMV0sIG1ldGFfcmVncmVzc2lvbjJiJGNpLnViWy0oMToyKV0pKSAtPiB0X21ldGhvZA0KICMgYFIyYCA9IGMocjJfbWV0YV9yZWdyZXNzaW9uMlsxXSwgcmVwKE5BLDgpKSkgLT4gdF9tZXRob2QNCg0KdF9tZXRob2QgJT4lIGthYmxlKCJodG1sIiwgZGlnaXRzID0gMykgJT4lDQogIGthYmxlX3N0eWxpbmcoInN0cmlwZWQiLCBwb3NpdGlvbiA9ICJsZWZ0IikgDQpgYGANCg0KIyMjIyBGaWd1cmUgMmMNCmBgYHtyIGZpZ3VyZTJjLCBmaWcud2lkdGg9NywgZmlnLmhlaWdodD0gM30NCg0KbXIyX2RhdGEkbW9kZXJhdG9yIDwtIGZhY3RvcihtcjJfZGF0YSRtb2RlcmF0b3IsIGxldmVscyA9IG1yMl9tb2RfdGFibGUkbmFtZSwgbGFiZWxzID0gbXIyX21vZF90YWJsZSRuYW1lKQ0KbXIyX2RhdGEkc2NhbGUgPC0gKDEvc3FydChtcjJfZGF0YVssInZpIl0pKQ0KbXIyX21vZF90YWJsZSRLIDwtIGFzLnZlY3RvcihieShtcjJfZGF0YSwgbXIyX2RhdGFbLCJtb2RlcmF0b3IiXSwgZnVuY3Rpb24oeCkgbGVuZ3RoKHhbLCJ5aSJdKSkpDQptcjJfZ3JvdXBfbm8gPC0gbnJvdyhtcjJfbW9kX3RhYmxlKQ0KDQppbWFnZV9jb252ZW50aW9uYWwgPC0gcmVhZFBORyhoZXJlKCJpbWFnZXMvQ29udmVudGlvbmFsX2ljb24ucG5nIikpDQppbWFnZV9HTFMgPC0gcmVhZFBORyhoZXJlKCJpbWFnZXMvR0xTX2ljb24ucG5nIikpDQppbWFnZV9zYXRlbGxpdGUgPC0gcmVhZFBORyhoZXJlKCJpbWFnZXMvU2F0ZWxsaXRlX2ljb24ucG5nIikpDQoNCiMgY3JlYXRpbmcgb3JjaGFSZCBwbG90DQpmaWdfbWV0aG9kIDwtIGdncGxvdChkYXRhID0gbXIyX21vZF90YWJsZSwgYWVzKHggPSBlc3RpbWF0ZSwgeSA9IG5hbWUpKSArIA0KICAgIHNjYWxlX3hfY29udGludW91cyhsaW1pdHMgPSBjKC0wLjM3LCAxKSwgYnJlYWtzID0gc2VxKC0wLjQsIDEsIGJ5ID0gMC4yKSwgbGFiZWxzID0gYygiLTAuNCIsICItMC4yIiwgIjAuMCIsICIwLjIiLCAiMC40IiwgIjAuNiIsICIwLjgiLCAiMS4wIikpICsgDQogICAgZ2diZWVzd2FybTo6Z2VvbV9xdWFzaXJhbmRvbShkYXRhID0gbXIyX2RhdGEsIGFlcyh4ID0geWlfSUNDLCB5ID0gbW9kZXJhdG9yLCBzaXplID0gc2NhbGUsIGNvbG91cj1tb2RlcmF0b3IpLCBncm91cE9uWD1GQUxTRSwgYWxwaGEgPSAwLjQpICsNCiAgICBnZW9tX2Vycm9yYmFyaChhZXMoeG1pbiA9IGxvd2VyUFIsIHhtYXggPSB1cHBlclBSKSwgaGVpZ2h0ID0gMCwgc2hvdy5sZWdlbmQgPSBGLCBzaXplID0gMC41LCBhbHBoYSA9IDAuNikgKyAjIENJDQogICAgZ2VvbV9lcnJvcmJhcmgoYWVzKHhtaW4gPSBsb3dlckNMLCB4bWF4ID0gdXBwZXJDTCksIGhlaWdodCA9IDAsIHNob3cubGVnZW5kID0gRiwgc2l6ZSA9IDEuMikgKyANCiAgICBnZW9tX3ZsaW5lKHhpbnRlcmNlcHQgPSAwLCBsaW5ldHlwZSA9IDIsIGNvbG91ciA9ICJibGFjayIsIGFscGhhID0gMC41KSArDQogICAgZ2VvbV9wb2ludChhZXMoZmlsbD1uYW1lKSwgc2l6ZSA9IDMsIHNoYXBlID0gMjEpICsgDQogICAgYW5ub3RhdGUoJ3RleHQnLCB4ID0gMC45MywgeSA9IChzZXEoMSwgbXIyX2dyb3VwX25vLCAxKSswLjM1KSwgbGFiZWw9IHBhc3RlKCJpdGFsaWMoayk9PSIsIG1yMl9tb2RfdGFibGUkSyksIHBhcnNlPVRSVUUsIGhqdXN0ID0gImxlZnQiLCBzaXplPTMuNSkgKw0KICAgIHNjYWxlX2NvbG9yX21hbnVhbCh2YWx1ZXMgPSBjKCJDb252ZW50aW9uYWwiID0gIiNDQzc5QTciLCAgIkdMUyIgPSAiI0Q1NUUwMCIsICAiU2F0ZWxsaXRlIj0gIiMwMDcyQjIiKSkgKw0KICBzY2FsZV9maWxsX21hbnVhbCh2YWx1ZXMgPSBjKCJDb252ZW50aW9uYWwiID0gIiNDQzc5QTciLCAgIkdMUyIgPSAiI0Q1NUUwMCIsICAiU2F0ZWxsaXRlIj0gIiMwMDcyQjIiKSkgKw0KICAgIGdncGxvdDI6OnRoZW1lX2J3KCkgKw0KICAgIGdncGxvdDI6Omd1aWRlcyhmaWxsPSJub25lIiwgY29sb3VyPSJub25lIikgKw0KICAgIGdncGxvdDI6OnRoZW1lKGF4aXMudGV4dC55ID0gZWxlbWVudF90ZXh0KHNpemU9OSwgY29sb3VyPSJibGFjayIsIGhqdXN0PTAuNSwgYW5nbGU9OTApLA0KICAgICAgICAgICAgICAgICAgIGxlZ2VuZC5wb3NpdGlvbiA9ICJub25lIiwgYXhpcy50aXRsZS55ID0gZWxlbWVudF9ibGFuaygpLCBheGlzLnRleHQueD1lbGVtZW50X3RleHQoc2l6ZT05KSkgKw0KICAgIGFubm90YXRpb25fY3VzdG9tKGdyaWQ6OnJhc3Rlckdyb2IoaW1hZ2Vfc2F0ZWxsaXRlKSwgeG1pbiA9IC0wLjQ1LCB4bWF4ID0gLTAuMjUsIHltaW4gPSAyLjcsIHltYXggPSAzLjMpICsNCiAgICBhbm5vdGF0aW9uX2N1c3RvbShncmlkOjpyYXN0ZXJHcm9iKGltYWdlX0dMUyksIHhtaW4gPSAtMC40NSwgeG1heCA9IC0wLjI1LCB5bWluID0gMS43LCB5bWF4ID0gMi4zKSArDQogICAgYW5ub3RhdGlvbl9jdXN0b20oZ3JpZDo6cmFzdGVyR3JvYihpbWFnZV9jb252ZW50aW9uYWwpLCB4bWluID0gLTAuNDUsIHhtYXggPSAtMC4yNSwgeW1pbiA9IDAuNywgeW1heCA9IDEuMykgKw0KICBsYWJzKHg9IkVmZmVjdCBzaXplIChJQ0MpIikNCg0KIyBmb3IgRmlndXJlIDINCg0KI2MgPC0gZ2dwbG90KGRhdGEgPSBtcjJfbW9kX3RhYmxlLCBhZXMoeCA9IGVzdGltYXRlLCB5ID0gbmFtZSkpICsgDQojICAgIHNjYWxlX3hfY29udGludW91cyhsaW1pdHMgPSBjKC0wLjM3LCAxKSwgYnJlYWtzID0gc2VxKC0wLjQsIDEsIGJ5ID0gMC4yKSwgbGFiZWxzID0gYygiLTAuNCIsICItMC4yIiwgIjAuMCIsICIwLjIiLCAiMC40IiwgIjAuNiIsICIwLjgiLCAiMS4wIikpICsgDQojICAgIGdnYmVlc3dhcm06Omdlb21fcXVhc2lyYW5kb20oZGF0YSA9IG1yMl9kYXRhLCBhZXMoeCA9IHlpX0lDQywgeSA9IG1vZGVyYXRvciwgc2l6ZSA9IHNjYWxlLCBjb2xvdXI9bW9kZXJhdG9yKSwgZ3JvdXBPblg9RkFMU0UsIGFscGhhID0gMC40KSArDQojICAgIGdlb21fZXJyb3JiYXJoKGFlcyh4bWluID0gbG93ZXJQUiwgeG1heCA9IHVwcGVyUFIpLCBoZWlnaHQgPSAwLCBzaG93LmxlZ2VuZCA9IEYsIHNpemUgPSAwLjUsIGFscGhhID0gMC42KSArICMgQ0kNCiMgICAgZ2VvbV9lcnJvcmJhcmgoYWVzKHhtaW4gPSBsb3dlckNMLCB4bWF4ID0gdXBwZXJDTCksIGhlaWdodCA9IDAsIHNob3cubGVnZW5kID0gRiwgc2l6ZSA9IDEuMikgKyANCiMgICAgZ2VvbV92bGluZSh4aW50ZXJjZXB0ID0gMCwgbGluZXR5cGUgPSAyLCBjb2xvdXIgPSAiYmxhY2siLCBhbHBoYSA9IDAuNSkgKw0KIyAgICBnZW9tX3BvaW50KGFlcyhmaWxsPW5hbWUpLCBzaXplID0gMywgc2hhcGUgPSAyMSkgKyANCiMgICAgYW5ub3RhdGUoJ3RleHQnLCB4ID0gMC45MywgeSA9IChzZXEoMSwgbXIyX2dyb3VwX25vLCAxKSswLjM1KSwgbGFiZWw9IHBhc3RlKCJpdGFsaWMoayk9PSIsIG1yMl9tb2RfdGFibGUkSyksIHBhcnNlPVRSVUUsIGhqdXN0ID0gImxlZnQiLCBzaXplPTMuNSkgKw0KIyAgICBzY2FsZV9jb2xvcl9tYW51YWwodmFsdWVzID0gYygiQ29udmVudGlvbmFsIiA9ICIjQ0M3OUE3IiwgICJHTFMiID0gIiNENTVFMDAiLCAgIlNhdGVsbGl0ZSI9ICIjMDA3MkIyIikpICsNCiMgIHNjYWxlX2ZpbGxfbWFudWFsKHZhbHVlcyA9IGMoIkNvbnZlbnRpb25hbCIgPSAiI0NDNzlBNyIsICAiR0xTIiA9ICIjRDU1RTAwIiwgICJTYXRlbGxpdGUiPSAiIzAwNzJCMiIpKSArDQojICAgIGdncGxvdDI6OnRoZW1lX2J3KCkgKw0KIyAgICBnZ3Bsb3QyOjpndWlkZXMoZmlsbD0ibm9uZSIsIGNvbG91cj0ibm9uZSIpICsNCiMgICAgZ2dwbG90Mjo6dGhlbWUoYXhpcy50ZXh0LnkgPSBlbGVtZW50X3RleHQoc2l6ZT05LCBjb2xvdXI9ImJsYWNrIiwgaGp1c3Q9MC41LCBhbmdsZT05MCksDQojICAgICAgICAgICAgICAgICAgIGxlZ2VuZC5wb3NpdGlvbiA9ICJub25lIiwgYXhpcy50aXRsZSA9IGVsZW1lbnRfYmxhbmsoKSkgKw0KIyAgICBhbm5vdGF0aW9uX2N1c3RvbShncmlkOjpyYXN0ZXJHcm9iKGltYWdlX3NhdGVsbGl0ZSksIHhtaW4gPSAtMC40NSwgeG1heCA9IC0wLjI1LCB5bWluID0gMi43LCB5bWF4ID0gMy4zKSArDQojICAgIGFubm90YXRpb25fY3VzdG9tKGdyaWQ6OnJhc3Rlckdyb2IoaW1hZ2VfR0xTKSwgeG1pbiA9IC0wLjQ1LCB4bWF4ID0gLTAuMjUsIHltaW4gPSAxLjcsIHltYXggPSAyLjMpICsNCiMgICAgYW5ub3RhdGlvbl9jdXN0b20oZ3JpZDo6cmFzdGVyR3JvYihpbWFnZV9jb252ZW50aW9uYWwpLCB4bWluID0gLTAuNDUsIHhtYXggPSAtMC4yNSwgeW1pbiA9IDAuNywgeW1heCA9IDEuMykNCg0KZmlnX21ldGhvZA0KDQpgYGANCg0KKipGaWd1cmUgMmMuKiogUmVwZWF0YWJpbGl0eSBvZiBhdmlhbiBtaWdyYXRpb24gdGltaW5nIGZvciB0cmFja2luZyBtZXRob2QuIFRoZSBwbG90IHNob3dzIHRoZSBncm91cC13aXNlIG1lYW5zIHdpdGggOTUlIGNvbmZpZGVuY2UgaW50ZXJ2YWxzICh0aGljayBsaW5lcykgYW5kIDk1JSBwcmVkaWN0aW9uIGludGVydmFscyAodGhpbiBsaW5lcyksIG9ic2VydmVkIGVmZmVjdCBzaXplcyAoYmFjay10cmFuc2Zvcm1lZCB0byBJQ0MpIHNjYWxlZCBieSBwcmVjaXNpb24gKGNpcmNsZXMpIGFuZCBrID0gbnVtYmVyIG9mIGVmZmVjdCBzaXplcy4NCg0KIyMjIyBUaGUgZWZmZWN0IG9mIHNleA0KDQpgYGB7ciBtZXRhLXJlZ3Jlc3Npb24tc2V4fQ0KIyByZW9yZGVyaW5nDQojZGYkc2V4IDwtIGZhY3RvcihkZiRzZXgsIGxldmVscyA9IGMoIkYiLCAiTSIsICJCIikpDQoNCm1ldGFfcmVncmVzc2lvbjQgPC0gcm1hLm12KHlpID0gWnIyLCBWID0gVkNWLCANCiAgICAgICAgICAgICAgICAgICAgICAgICAgIG1vZHMgPSB+IHNleCwNCiAgICAgICAgICAgICAgICAgICAgICAgICAgIHJhbmRvbSA9IGxpc3QofjEgfCBlc19JRCwgDQogICAgICAgICAgICAgICAgICAgICAgICAgICAgICAgICAgICAgICAgIH4xIHwgcGFwZXJfSUQsIA0KICAgICAgICAgICAgICAgICAgICAgICAgICAgICAgICAgICAgICAgICB+MSB8IGNvaG9ydF9JRCwgDQogICAgICAgICAgICAgICAgICAgICAgICAgICAgICAgICAgICAgICAgIH4xIHwgc3BlY2llc19JRCwgDQogICAgICAgICAgICAgICAgICAgICAgICAgICAgICAgICAgICAgICAgIH4xIHwgcGh5bG9nZW55KSwNCiAgICAgICAgICAgICAgICAgICAgICAgICAgIFIgPSBsaXN0KHBoeWxvZ2VueSA9IHZhcmNvciksDQogICAgICAgICAgICAgICAgICAgICAgICAgICBkYXRhID0gZGYpDQoNCm1ldGFfcmVncmVzc2lvbjRiIDwtIHJtYS5tdih5aSA9IFpyMiwgViA9IFZDViwgDQogICAgICAgICAgICAgICAgICAgICAgICAgICAgbW9kcyA9IH4gcmVsZXZlbChzZXgsIHJlZiA9ICJNIiksDQogICAgICAgICAgICAgICAgICAgICAgICAgICAgcmFuZG9tID0gbGlzdCh+MSB8IGVzX0lELCANCiAgICAgICAgICAgICAgICAgICAgICAgICAgICAgICAgICAgICAgICAgIH4xIHwgcGFwZXJfSUQsIA0KICAgICAgICAgICAgICAgICAgICAgICAgICAgICAgICAgICAgICAgICAgfjEgfCBjb2hvcnRfSUQsIA0KICAgICAgICAgICAgICAgICAgICAgICAgICAgICAgICAgICAgICAgICAgfjEgfCBzcGVjaWVzX0lELCANCiAgICAgICAgICAgICAgICAgICAgICAgICAgICAgICAgICAgICAgICAgIH4xIHwgcGh5bG9nZW55KSwNCiAgICAgICAgICAgICAgICAgICAgICAgICAgICBSID0gbGlzdChwaHlsb2dlbnkgPSB2YXJjb3IpLCANCiAgICAgICAgICAgICAgICAgICAgICAgICAgICBkYXRhID0gZGYpDQoNCiMgT3JjaGFyZCBwbG90IC0gbmVlZCBtZXRhLXJlZ3Jlc3Npb24gd2l0aG91dCBpbnRlcmNlcHQNCm1ldGFfcmVncmVzc2lvbjRjIDwtIHJtYS5tdih5aSA9IFpyMiwgViA9IFZDViwgDQogICAgICAgICAgICAgICAgICAgICAgICAgICAgbW9kcyA9IH4gc2V4IC0xLA0KICAgICAgICAgICAgICAgICAgICAgICAgICAgIHJhbmRvbSA9IGxpc3QofjEgfCBlc19JRCwgDQogICAgICAgICAgICAgICAgICAgICAgICAgICAgICAgICAgICAgICAgICB+MSB8IHBhcGVyX0lELCANCiAgICAgICAgICAgICAgICAgICAgICAgICAgICAgICAgICAgICAgICAgIH4xIHwgY29ob3J0X0lELCANCiAgICAgICAgICAgICAgICAgICAgICAgICAgICAgICAgICAgICAgICAgIH4xIHwgc3BlY2llc19JRCwgDQogICAgICAgICAgICAgICAgICAgICAgICAgICAgICAgICAgICAgICAgICB+MSB8IHBoeWxvZ2VueSksDQogICAgICAgICAgICAgICAgICAgICAgICAgICAgUiA9IGxpc3QocGh5bG9nZW55ID0gdmFyY29yKSwNCiAgICAgICAgICAgICAgICAgICAgICAgICAgICBkYXRhID0gZGYpDQoNCg0KYGBgDQoNCiMjIyMgVGFibGUgUzYNClJlZ3Jlc3Npb24gY29lZmZpY2llbnRzIChFc3RpbWF0ZSkgYW5kIDk1JSBjb25maWRlbmNlIGludGVydmFscyAoQ0lzKSBib3RoIGluIFpyIGFuZCBiYWNrLXRyYW5zZm9ybWVkIHRvIElDQyBmcm9tIHRoZSBtZXRhLXJlZ3Jlc3Npb24gd2l0aCBgc2V4YC4gTm90ZSB0aGF0IGBtdWAgbWVhbnMgdGhlIGdyb3VwIG1lYW4gd2hpbGUgYGJldGFgIHJlcHJlc2VudHMgdGhlIGNvbnRyYXN0IGJldHdlZW4gdHdvIGdyb3VwcyBpbiB0aGUgVW5pdCBjb2x1bW4uICpSPHN1cD4yPC9zdXA+PHN1Yj5tYXJnaW5hbDwvc3ViPiogPSBgciByb3VuZChvcmNoYVJkOjpyMl9tbChtZXRhX3JlZ3Jlc3Npb240KVtbMV1dKjEwMCwxKWAlLiBCID0gYm90aCBzZXhlcywgTSA9IG1hbGUsIGFuZCBGID0gZmVtYWxlLg0KDQpgYGB7ciBzZXgtdGFibGV9DQojIGdldHRpbmcgbWFyZ2luYWwgUjINCnIyX21ldGFfcmVncmVzc2lvbjQgPC0gcjJfbWwobWV0YV9yZWdyZXNzaW9uNCkNCg0KIyBnZXR0aW5nIGVzdGltYXRlcw0KIyBpbmNsdWRpbmcgYmFjay10cmFuc2Zvcm1hdGlvbiB0byBJQ0MNCg0KbXI0IDwtIG1vZF9yZXN1bHRzKG1ldGFfcmVncmVzc2lvbjRjLCBtb2Q9InNleCIpDQptcjRfbW9kX3RhYmxlIDwtIG1yNCRtb2RfdGFibGUNCg0KbXI0X2RhdGEgPC0gbXI0JGRhdGENCg0KIyBjYWxjdWxhdGUgayBmb3IgZWFjaCBtZXRob2Qgc2VwYXJhdGVseQ0KDQojZGYgJT4lIGdyb3VwX2J5KHNleCkgJT4lIHN1bW1hcmlzZShtZWFuKGspKQ0KDQoNCm1yNF9kYXRhIDwtIG1yNF9kYXRhICU+JSBtdXRhdGUoayA9IGNhc2Vfd2hlbihtb2RlcmF0b3IgPT0gIkYiIH4gMi4zNywgDQogICAgICAgICAgICAgICAgICAgICAgICAgICAgICAgICAgICAgICAgICAgICAgbW9kZXJhdG9yID09ICJNIiB+IDIuMzgsDQogICAgICAgICAgICAgICAgICAgICAgICAgICAgICAgICAgICAgICAgICAgICAgbW9kZXJhdG9yID09ICJCIiB+IDIuODIpKQ0KDQptcjRfZGF0YSR5aV9JQ0MgPC0gWnJfdG9fSUNDKG1yNF9kYXRhJHlpLCBtcjRfZGF0YSRrKQ0KDQptcjRfbW9kX3RhYmxlJGsgPC0gYygyLjM3LCAyLjM4LCAyLjgyKQ0KDQpmb3IoaSBpbiBuYW1lcyhtcjRfbW9kX3RhYmxlKVsyOjZdKXsNCiAgDQogIG1yNF9tb2RfdGFibGVbaV0gPC0gWnJfdG9fSUNDKG1yNF9tb2RfdGFibGVbaV0sIG1yNF9tb2RfdGFibGUkaykNCiAgDQp9DQoNCiMgY3JlYXRpbmcgYSB0YWJsZQ0KdGliYmxlKA0KICBgRml4ZWQgZWZmZWN0YCA9IGMocmVwKGFzLmNoYXJhY3RlcihtcjRfbW9kX3RhYmxlJG5hbWUpLDIpLCBjb250X2dlbihtcjRfbW9kX3RhYmxlJG5hbWUpKSwNCiAgYFVuaXRgID0gYyhyZXAoYygiWnIgKG11KSIsICJJQ0MgKG11KSIpLGVhY2ggPSAzKSwgcmVwKCJaciAoYmV0YSkiLCAzKSksDQogIGBFc3RpbWF0ZWAgPSBjKG1ldGFfcmVncmVzc2lvbjRjJGIsIG1yNF9tb2RfdGFibGUkZXN0aW1hdGUsIA0KICAgICAgICAgICAgICAgICBtZXRhX3JlZ3Jlc3Npb240JGJbLTFdLCBtZXRhX3JlZ3Jlc3Npb240YiRiWy0oMToyKV0pLA0KICBgTG93ZXIgQ0kgWzAuMDI1XWAgPSBjKG1ldGFfcmVncmVzc2lvbjRjJGNpLmxiLCBtcjRfbW9kX3RhYmxlJGxvd2VyQ0wsDQogICAgICAgICAgICAgICAgICAgICAgICAgbWV0YV9yZWdyZXNzaW9uNCRjaS5sYlstMV0sIG1ldGFfcmVncmVzc2lvbjRiJGNpLmxiWy0oMToyKV0pLA0KICBgVXBwZXIgQ0kgIFswLjk3NV1gID0gYyhtZXRhX3JlZ3Jlc3Npb240YyRjaS51YiwgbXI0X21vZF90YWJsZSR1cHBlckNMLA0KICAgICAgICAgICAgICAgICAgICAgICAgIG1ldGFfcmVncmVzc2lvbjQkY2kudWJbLTFdLCBtZXRhX3JlZ3Jlc3Npb240YiRjaS51YlstKDE6MildKSkgLT4gdF9zZXgNCiAjIGBSMmAgPSBjKHIyX21ldGFfcmVncmVzc2lvbjRbMV0sIHJlcChOQSw4KSkpIC0+IHRfc2V4DQoNCnRfc2V4ICU+JSBrYWJsZSgiaHRtbCIsIGRpZ2l0cyA9IDMpICU+JQ0KICBrYWJsZV9zdHlsaW5nKCJzdHJpcGVkIiwgcG9zaXRpb24gPSAibGVmdCIpDQoNCmBgYA0KDQojIyMjIEZpZ3VyZSAyZQ0KYGBge3IgZmlndXJlMmUsIGZpZy53aWR0aD03LCBmaWcuaGVpZ2h0PSAzfQ0KDQptcjRfZGF0YSRtb2RlcmF0b3IgPC0gZmFjdG9yKG1yNF9kYXRhJG1vZGVyYXRvciwgbGV2ZWxzID0gbXI0X21vZF90YWJsZSRuYW1lLCBsYWJlbHMgPSBtcjRfbW9kX3RhYmxlJG5hbWUpDQptcjRfZGF0YSRzY2FsZSA8LSAoMS9zcXJ0KG1yNF9kYXRhWywidmkiXSkpDQptcjRfbW9kX3RhYmxlJEsgPC0gYXMudmVjdG9yKGJ5KG1yNF9kYXRhLCBtcjRfZGF0YVssIm1vZGVyYXRvciJdLCBmdW5jdGlvbih4KSBsZW5ndGgoeFssInlpIl0pKSkNCm1yNF9ncm91cF9ubyA8LSBucm93KG1yNF9tb2RfdGFibGUpDQoNCmltYWdlX21hbGUgPC0gcmVhZFBORyhoZXJlKCJpbWFnZXMvTWFsZV9pY29uLnBuZyIpKQ0KaW1hZ2VfZmVtYWxlIDwtIHJlYWRQTkcoaGVyZSgiaW1hZ2VzL0ZlbWFsZV9pY29uLnBuZyIpKQ0KaW1hZ2VfYm90aCA8LSByZWFkUE5HKGhlcmUoImltYWdlcy9Cb3RoX3NleF9pY29uLnBuZyIpKQ0KDQojIGNyZWF0aW5nIG9yY2hhUmQgcGxvdA0KIyBmb3IgRmlndXJlIDINCg0KZSA8LSBnZ3Bsb3QoZGF0YSA9IG1yNF9tb2RfdGFibGUsIGFlcyh4ID0gZXN0aW1hdGUsIHkgPSBuYW1lKSkgKyANCiAgICBzY2FsZV94X2NvbnRpbnVvdXMobGltaXRzID0gYygtMC4zNywgMSksIGJyZWFrcyA9IHNlcSgtMC40LCAxLCBieSA9IDAuMiksIGxhYmVscyA9IGMoIi0wLjQiLCAiLTAuMiIsICIwLjAiLCAiMC4yIiwgIjAuNCIsICIwLjYiLCAiMC44IiwgIjEuMCIpKSArICANCiAgICBnZ2JlZXN3YXJtOjpnZW9tX3F1YXNpcmFuZG9tKGRhdGEgPSBtcjRfZGF0YSwgYWVzKHggPSB5aV9JQ0MsIHkgPSBtb2RlcmF0b3IsIHNpemUgPSBzY2FsZSwgY29sb3VyPW1vZGVyYXRvciksIGdyb3VwT25YPUZBTFNFLCBhbHBoYSA9IDAuNCkgKw0KICAgIGdlb21fZXJyb3JiYXJoKGFlcyh4bWluID0gbG93ZXJQUiwgeG1heCA9IHVwcGVyUFIpLCBoZWlnaHQgPSAwLCBzaG93LmxlZ2VuZCA9IEYsIHNpemUgPSAwLjUsIGFscGhhID0gMC42KSArICMgQ0kNCiAgICBnZW9tX2Vycm9yYmFyaChhZXMoeG1pbiA9IGxvd2VyQ0wsIHhtYXggPSB1cHBlckNMKSwgaGVpZ2h0ID0gMCwgc2hvdy5sZWdlbmQgPSBGLCBzaXplID0gMS4yKSArIA0KICAgIGdlb21fdmxpbmUoeGludGVyY2VwdCA9IDAsIGxpbmV0eXBlID0gMiwgY29sb3VyID0gImJsYWNrIiwgYWxwaGEgPSAwLjUpICsNCiAgICBnZW9tX3BvaW50KGFlcyhmaWxsPW5hbWUpLCBzaXplID0gMywgc2hhcGUgPSAyMSkgKyANCiAgICBhbm5vdGF0ZSgndGV4dCcsIHggPSAwLjkzLCB5ID0gKHNlcSgxLCBtcjRfZ3JvdXBfbm8sIDEpKzAuMzUpLCBsYWJlbD0gcGFzdGUoIml0YWxpYyhrKT09IiwgbXI0X21vZF90YWJsZSRLKSwgcGFyc2U9VFJVRSwgaGp1c3QgPSAibGVmdCIsIHNpemU9My41KSArDQogICAgZ2dwbG90Mjo6dGhlbWVfYncoKSArDQogICAgZ2dwbG90Mjo6Z3VpZGVzKGZpbGw9Im5vbmUiLCBjb2xvdXI9Im5vbmUiKSArDQogICAgZ2dwbG90Mjo6dGhlbWUoYXhpcy50ZXh0LnkgPSBlbGVtZW50X3RleHQoc2l6ZT05LCBjb2xvdXI9ImJsYWNrIiwgaGp1c3Q9MC41LCBhbmdsZT05MCksDQogICAgICAgICAgICAgICAgICAgbGVnZW5kLnBvc2l0aW9uID0gIm5vbmUiLCBheGlzLnRpdGxlLnkgPSBlbGVtZW50X2JsYW5rKCkpICsNCiAgICBnZ3Bsb3QyOjpsYWJzKHg9cGFzdGUoIkVmZmVjdCBzaXplIChJQ0MpIikpICsNCiAgICBzY2FsZV95X2Rpc2NyZXRlKGxhYmVscz1jKCJGIiA9ICJGZW1hbGUiLCAiTSIgPSAiTWFsZSIsICJCIiA9ICJCb3RoIikpICsNCiAgICAJICAgICAgc2NhbGVfZmlsbF9tYW51YWwodmFsdWVzPWNicGwpICsNCgkgICAgICBzY2FsZV9jb2xvdXJfbWFudWFsKHZhbHVlcz1jYnBsKSArDQogICAgICAgIGFubm90YXRpb25fY3VzdG9tKGdyaWQ6OnJhc3Rlckdyb2IoaW1hZ2VfbWFsZSksIHhtaW4gPSAtMC40NSwgeG1heCA9IC0wLjI1LCB5bWluID0gMi43LCB5bWF4ID0gMy4zKSArDQogICAgYW5ub3RhdGlvbl9jdXN0b20oZ3JpZDo6cmFzdGVyR3JvYihpbWFnZV9mZW1hbGUpLCB4bWluID0gLTAuNDUsIHhtYXggPSAtMC4yNSwgeW1pbiA9IDEuNywgeW1heCA9IDIuMykgKw0KICAgIGFubm90YXRpb25fY3VzdG9tKGdyaWQ6OnJhc3Rlckdyb2IoaW1hZ2VfYm90aCksIHhtaW4gPSAtMC40NSwgeG1heCA9IC0wLjI1LCB5bWluID0gMC43LCB5bWF4ID0gMS4zKQ0KDQplDQoNCmBgYA0KDQoqKkZpZ3VyZSAyZS4qKiBSZXBlYXRhYmlsaXR5IG9mIGF2aWFuIG1pZ3JhdGlvbiB0aW1pbmcgZm9yIHNleC4gVGhlIHBsb3Qgc2hvd3MgdGhlIGdyb3VwLXdpc2UgbWVhbnMgd2l0aCA5NSUgY29uZmlkZW5jZSBpbnRlcnZhbHMgKHRoaWNrIGxpbmVzKSBhbmQgOTUlIHByZWRpY3Rpb24gaW50ZXJ2YWxzICh0aGluIGxpbmVzKSwgb2JzZXJ2ZWQgZWZmZWN0IHNpemVzIChiYWNrLXRyYW5zZm9ybWVkIHRvIElDQykgc2NhbGVkIGJ5IHByZWNpc2lvbiAoY2lyY2xlcykgYW5kIGsgPSBudW1iZXIgb2YgZWZmZWN0IHNpemVzLg0KDQoNCiMjIyMgVGhlIGVmZmVjdCBvZiBlY29sb2dpY2FsIGdyb3VwDQoNCmBgYHtyIG1ldGEtcmVncmVzc2lvbi10YXhhfQ0KbWV0YV9yZWdyZXNzaW9uMyA8LSBybWEubXYoeWkgPSBacjIsIFYgPSBWQ1YsIA0KICAgICAgICAgICAgICAgICAgICAgICAgICAgbW9kcyA9IH4gdGF4YSwNCiAgICAgICAgICAgICAgICAgICAgICAgICAgIHJhbmRvbSA9IGxpc3QofjEgfCBlc19JRCwgDQogICAgICAgICAgICAgICAgICAgICAgICAgICAgICAgICAgICAgICAgIH4xIHwgcGFwZXJfSUQsIA0KICAgICAgICAgICAgICAgICAgICAgICAgICAgICAgICAgICAgICAgICB+MSB8IGNvaG9ydF9JRCwgDQogICAgICAgICAgICAgICAgICAgICAgICAgICAgICAgICAgICAgICAgIH4xIHwgc3BlY2llc19JRCwgDQogICAgICAgICAgICAgICAgICAgICAgICAgICAgICAgICAgICAgICAgIH4xIHwgcGh5bG9nZW55KSwNCiAgICAgICAgICAgICAgICAgICAgICAgICAgIFIgPSBsaXN0KHBoeWxvZ2VueSA9IHZhcmNvciksIA0KICAgICAgICAgICAgICAgICAgICAgICAgICAgZGF0YSA9IGRmKQ0KDQojIHJlb3JkZXJpbmcNCiNkZiR0YXhhIDwtIGZhY3RvcihkZiR0YXhhLCBsZXZlbHMgPSBjKCJXYXRlcmJpcmQiLCAiU2VhYmlyZCIsICJMYW5kYmlyZCIpKQ0KDQptZXRhX3JlZ3Jlc3Npb24zYiA8LSBybWEubXYoeWkgPSBacjIsIFYgPSBWQ1YsIA0KICAgICAgICAgICAgICAgICAgICAgICAgICAgbW9kcyA9IH4gcmVsZXZlbCh0YXhhLCByZWYgPSAiV2F0ZXJiaXJkIiksDQogICAgICAgICAgICAgICAgICAgICAgICAgICByYW5kb20gPSBsaXN0KH4xIHwgZXNfSUQsIA0KICAgICAgICAgICAgICAgICAgICAgICAgICAgICAgICAgICAgICAgICB+MSB8IHBhcGVyX0lELCANCiAgICAgICAgICAgICAgICAgICAgICAgICAgICAgICAgICAgICAgICAgfjEgfCBjb2hvcnRfSUQsIA0KICAgICAgICAgICAgICAgICAgICAgICAgICAgICAgICAgICAgICAgICB+MSB8IHNwZWNpZXNfSUQsIA0KICAgICAgICAgICAgICAgICAgICAgICAgICAgICAgICAgICAgICAgICB+MSB8IHBoeWxvZ2VueSksDQogICAgICAgICAgICAgICAgICAgICAgICAgICBSID0gbGlzdChwaHlsb2dlbnkgPSB2YXJjb3IpLCANCiAgICAgICAgICAgICAgICAgICAgICAgICAgIGRhdGEgPSBkZikNCg0KIyBPcmNoYXJkIHBsb3QgLSBuZWVkIG1ldGEtcmVncmVzc2lvbiB3aXRob3V0IGludGVyY2VwdA0KbWV0YV9yZWdyZXNzaW9uM2MgPC0gcm1hLm12KHlpID0gWnIyLCBWID0gVkNWLCANCiAgICAgICAgICAgICAgICAgICAgICAgICAgICBtb2RzID0gfiB0YXhhIC0xLA0KICAgICAgICAgICAgICAgICAgICAgICAgICAgIHJhbmRvbSA9IGxpc3QofjEgfCBlc19JRCwgDQogICAgICAgICAgICAgICAgICAgICAgICAgICAgICAgICAgICAgICAgICB+MSB8IHBhcGVyX0lELCANCiAgICAgICAgICAgICAgICAgICAgICAgICAgICAgICAgICAgICAgICAgIH4xIHwgY29ob3J0X0lELCANCiAgICAgICAgICAgICAgICAgICAgICAgICAgICAgICAgICAgICAgICAgIH4xIHwgc3BlY2llc19JRCwgDQogICAgICAgICAgICAgICAgICAgICAgICAgICAgICAgICAgICAgICAgICB+MSB8IHBoeWxvZ2VueSksDQogICAgICAgICAgICAgICAgICAgICAgICAgICAgUiA9IGxpc3QocGh5bG9nZW55ID0gdmFyY29yKSwNCiAgICAgICAgICAgICAgICAgICAgICAgICAgICBkYXRhID0gZGYpDQoNCmBgYA0KDQojIyMjIFRhYmxlIFM3DQpSZWdyZXNzaW9uIGNvZWZmaWNpZW50cyAoRXN0aW1hdGUpIGFuZCA5NSUgY29uZmlkZW5jZSBpbnRlcnZhbHMgKENJcykgYm90aCBpbiBaciBhbmQgYmFjay10cmFuc2Zvcm1lZCB0byBJQ0MgZnJvbSB0aGUgbWV0YS1yZWdyZXNzaW9uIHdpdGggYHRheGFgLiBOb3RlIHRoYXQgYG11YCBtZWFucyB0aGUgZ3JvdXAgbWVhbiB3aGlsZSBgYmV0YWAgcmVwcmVzZW50cyB0aGUgY29udHJhc3QgYmV0d2VlbiB0d28gZ3JvdXBzIGluIHRoZSBVbml0IGNvbHVtbi4gKlI8c3VwPjI8L3N1cD48c3ViPm1hcmdpbmFsPC9zdWI+KiA9IGByIHJvdW5kKG9yY2hhUmQ6OnIyX21sKG1ldGFfcmVncmVzc2lvbjMpW1sxXV0qMTAwLDEpYCUuIA0KDQpgYGB7ciB0YXhhLXRhYmxlfQ0KIyBnZXR0aW5nIG1hcmdpbmFsIFIyDQpyMl9tZXRhX3JlZ3Jlc3Npb24zIDwtIHIyX21sKG1ldGFfcmVncmVzc2lvbjMpDQoNCiMgZ2V0dGluZyBlc3RpbWF0ZXMNCiMgaW5jbHVkaW5nIGJhY2stdHJhbnNmb3JtYXRpb24gdG8gSUNDDQoNCm1yMyA8LSBtb2RfcmVzdWx0cyhtZXRhX3JlZ3Jlc3Npb24zYywgbW9kPSJ0YXhhIikNCm1yM19tb2RfdGFibGUgPC0gbXIzJG1vZF90YWJsZQ0KDQptcjNfZGF0YSA8LSBtcjMkZGF0YQ0KDQojIGNhbGN1bGF0ZSBrIGZvciBlYWNoIG1ldGhvZCBzZXBhcmF0ZWx5DQoNCiNkZiAlPiUgZ3JvdXBfYnkodGF4YSkgJT4lIHN1bW1hcmlzZShtZWFuKGspKQ0KDQptcjNfZGF0YSA8LSBtcjNfZGF0YSAlPiUgbXV0YXRlKGsgPSBjYXNlX3doZW4obW9kZXJhdG9yID09ICJMYW5kYmlyZCIgfiAyLjY4LCANCiAgICAgICAgICAgICAgICAgICAgICAgICAgICAgICAgICAgICAgICAgICAgICBtb2RlcmF0b3IgPT0gIlNlYWJpcmQiIH4gMi4zOCwNCiAgICAgICAgICAgICAgICAgICAgICAgICAgICAgICAgICAgICAgICAgICAgICBtb2RlcmF0b3IgPT0gIldhdGVyYmlyZCIgfiAzLjA4KSkNCg0KbXIzX2RhdGEkeWlfSUNDIDwtIFpyX3RvX0lDQyhtcjNfZGF0YSR5aSwgbXIzX2RhdGEkaykNCg0KbXIzX21vZF90YWJsZSRrIDwtIGMoMi42OCwgMi4zOCwgMy4wOCkNCg0KZm9yKGkgaW4gbmFtZXMobXIzX21vZF90YWJsZSlbMjo2XSl7DQogIA0KICBtcjNfbW9kX3RhYmxlW2ldIDwtIFpyX3RvX0lDQyhtcjNfbW9kX3RhYmxlW2ldLCBtcjNfbW9kX3RhYmxlJGspDQogIA0KfQ0KDQojIGNyZWF0aW5nIGEgdGFibGUNCnRpYmJsZSgNCiAgYEZpeGVkIGVmZmVjdGAgPSBjKHJlcChhcy5jaGFyYWN0ZXIobXIzX21vZF90YWJsZSRuYW1lKSwyKSwgY29udF9nZW4obXIzX21vZF90YWJsZSRuYW1lKSksDQogIGBVbml0YCA9IGMocmVwKGMoIlpyIChtdSkiLCAiSUNDIChtdSkiKSxlYWNoID0gMyksIHJlcCgiWnIgKGJldGEpIiwgMykpLA0KICBgRXN0aW1hdGVgID0gYyhtZXRhX3JlZ3Jlc3Npb24zYyRiLCBtcjNfbW9kX3RhYmxlJGVzdGltYXRlLCANCiAgICAgICAgICAgICAgICAgbWV0YV9yZWdyZXNzaW9uMyRiWy0xXSwgbWV0YV9yZWdyZXNzaW9uM2IkYlstKDE6MildKSwNCiAgYExvd2VyIENJIFswLjAyNV1gID0gYyhtZXRhX3JlZ3Jlc3Npb24zYyRjaS5sYiwgbXIzX21vZF90YWJsZSRsb3dlckNMLA0KICAgICAgICAgICAgICAgICAgICAgICAgIG1ldGFfcmVncmVzc2lvbjMkY2kubGJbLTFdLCBtZXRhX3JlZ3Jlc3Npb24zYiRjaS5sYlstKDE6MildKSwNCiAgYFVwcGVyIENJICBbMC45NzVdYCA9IGMobWV0YV9yZWdyZXNzaW9uM2MkY2kudWIsIG1yM19tb2RfdGFibGUkdXBwZXJDTCwNCiAgICAgICAgICAgICAgICAgICAgICAgICBtZXRhX3JlZ3Jlc3Npb24zJGNpLnViWy0xXSwgbWV0YV9yZWdyZXNzaW9uM2IkY2kudWJbLSgxOjIpXSkpIC0+IHRfbWV0aG9kDQogIyBgUjJgID0gYyhyMl9tZXRhX3JlZ3Jlc3Npb24zWzFdLCByZXAoTkEsOCkpKSAtPiB0X21ldGhvZA0KDQp0X21ldGhvZCAlPiUga2FibGUoImh0bWwiLCBkaWdpdHMgPSAzKSAlPiUNCiAga2FibGVfc3R5bGluZygic3RyaXBlZCIsIHBvc2l0aW9uID0gImxlZnQiKSANCg0KYGBgDQoNCiMjIyMgRmlndXJlIDJkDQpgYGB7ciBmaWd1cmUyZCwgZmlnLndpZHRoPTcsIGZpZy5oZWlnaHQ9IDN9DQoNCm1yM19kYXRhJG1vZGVyYXRvciA8LSBmYWN0b3IobXIzX2RhdGEkbW9kZXJhdG9yLCBsZXZlbHMgPSBtcjNfbW9kX3RhYmxlJG5hbWUsIGxhYmVscyA9IG1yM19tb2RfdGFibGUkbmFtZSkNCm1yM19kYXRhJHNjYWxlIDwtICgxL3NxcnQobXIzX2RhdGFbLCJ2aSJdKSkNCm1yM19tb2RfdGFibGUkSyA8LSBhcy52ZWN0b3IoYnkobXIzX2RhdGEsIG1yM19kYXRhWywibW9kZXJhdG9yIl0sIGZ1bmN0aW9uKHgpIGxlbmd0aCh4WywieWkiXSkpKQ0KbXIzX2dyb3VwX25vIDwtIG5yb3cobXIzX21vZF90YWJsZSkNCg0KaW1hZ2Vfd2F0ZXJiaXJkIDwtIHJlYWRQTkcoaGVyZSgiaW1hZ2VzL1dhdGVyYmlyZF9pY29uLnBuZyIpKQ0KaW1hZ2Vfc2VhYmlyZCA8LSByZWFkUE5HKGhlcmUoImltYWdlcy9TZWFiaXJkX2ljb24ucG5nIikpDQppbWFnZV9sYW5kYmlyZCA8LSByZWFkUE5HKGhlcmUoImltYWdlcy9MYW5kYmlyZF9pY29uLnBuZyIpKQ0KDQojIGNyZWF0aW5nIG9yY2hhUmQgcGxvdA0KDQpmaWdfZWNvX2dyb3VwIDwtIGdncGxvdChkYXRhID0gbXIzX21vZF90YWJsZSwgYWVzKHggPSBlc3RpbWF0ZSwgeSA9IG5hbWUpKSArIA0KICAgIHNjYWxlX3hfY29udGludW91cyhsaW1pdHMgPSBjKC0wLjM3LCAxKSwgYnJlYWtzID0gc2VxKC0wLjQsIDEsIGJ5ID0gMC4yKSwgbGFiZWxzID0gYygiLTAuNCIsICItMC4yIiwgIjAuMCIsICIwLjIiLCAiMC40IiwgIjAuNiIsICIwLjgiLCAiMS4wIikpICsgDQogICAgZ2diZWVzd2FybTo6Z2VvbV9xdWFzaXJhbmRvbShkYXRhID0gbXIzX2RhdGEsIGFlcyh4ID0geWlfSUNDLCB5ID0gbW9kZXJhdG9yLCBzaXplID0gc2NhbGUsIGNvbG91cj1tb2RlcmF0b3IpLCBncm91cE9uWD1GQUxTRSwgYWxwaGEgPSAwLjQpICsNCiAgICBnZW9tX2Vycm9yYmFyaChhZXMoeG1pbiA9IGxvd2VyUFIsIHhtYXggPSB1cHBlclBSKSwgaGVpZ2h0ID0gMCwgc2hvdy5sZWdlbmQgPSBGLCBzaXplID0gMC41LCBhbHBoYSA9IDAuNikgKyAjIENJDQogICAgZ2VvbV9lcnJvcmJhcmgoYWVzKHhtaW4gPSBsb3dlckNMLCB4bWF4ID0gdXBwZXJDTCksIGhlaWdodCA9IDAsIHNob3cubGVnZW5kID0gRiwgc2l6ZSA9IDEuMikgKyANCiAgICBnZW9tX3ZsaW5lKHhpbnRlcmNlcHQgPSAwLCBsaW5ldHlwZSA9IDIsIGNvbG91ciA9ICJibGFjayIsIGFscGhhID0gMC41KSArDQogICAgZ2VvbV9wb2ludChhZXMoZmlsbD1uYW1lKSwgc2l6ZSA9IDMsIHNoYXBlID0gMjEpICsgDQogICAgYW5ub3RhdGUoJ3RleHQnLCB4ID0gMC45MywgeSA9IChzZXEoMSwgbXIzX2dyb3VwX25vLCAxKSswLjM1KSwgbGFiZWw9IHBhc3RlKCJpdGFsaWMoayk9PSIsIG1yM19tb2RfdGFibGUkSyksIHBhcnNlPVRSVUUsIGhqdXN0ID0gImxlZnQiLCBzaXplPTMuNSkgKw0KICAgIGdncGxvdDI6OnRoZW1lX2J3KCkgKw0KICAgIGdncGxvdDI6Omd1aWRlcyhmaWxsPSJub25lIiwgY29sb3VyPSJub25lIikgKw0KICAgIGdncGxvdDI6OnRoZW1lKGF4aXMudGV4dC55ID0gZWxlbWVudF90ZXh0KHNpemU9OSwgY29sb3VyPSJibGFjayIsIGhqdXN0PTAuNSwgYW5nbGU9OTApLA0KICAgICAgICAgICAgICAgICAgIGxlZ2VuZC5wb3NpdGlvbiA9ICJub25lIiwgYXhpcy50aXRsZS55ID0gZWxlbWVudF9ibGFuaygpLCBheGlzLnRleHQueD1lbGVtZW50X3RleHQoc2l6ZT05KSkgKw0KICAgICMgc2V0dGluZyBjb2xvdXJzDQogIHNjYWxlX2NvbG9yX21hbnVhbCh2YWx1ZXMgPSBjKCJTZWFiaXJkIiA9ICIjMDA5RTczIiwgICJMYW5kYmlyZCIgPSAiI0Q1NUUwMCIsICAiV2F0ZXJiaXJkIj0gIiMwMDcyQjIiKSkgKw0KICBzY2FsZV9maWxsX21hbnVhbCh2YWx1ZXMgPSBjKCJTZWFiaXJkIiA9ICIjMDA5RTczIiwgICJMYW5kYmlyZCIgPSAiI0Q1NUUwMCIsICAiV2F0ZXJiaXJkIj0gIiMwMDcyQjIiKSkgKw0KICAgIGFubm90YXRpb25fY3VzdG9tKGdyaWQ6OnJhc3Rlckdyb2IoaW1hZ2Vfd2F0ZXJiaXJkKSwgeG1pbiA9IC0wLjQ1LCB4bWF4ID0gLTAuMjUsIHltaW4gPSAyLjcsIHltYXggPSAzLjMpICsNCiAgICBhbm5vdGF0aW9uX2N1c3RvbShncmlkOjpyYXN0ZXJHcm9iKGltYWdlX3NlYWJpcmQpLCB4bWluID0gLTAuNDUsIHhtYXggPSAtMC4yNSwgeW1pbiA9IDEuNywgeW1heCA9IDIuMykgKw0KICAgIGFubm90YXRpb25fY3VzdG9tKGdyaWQ6OnJhc3Rlckdyb2IoaW1hZ2VfbGFuZGJpcmQpLCB4bWluID0gLTAuNDUsIHhtYXggPSAtMC4yNSwgeW1pbiA9IDAuNywgeW1heCA9IDEuMykgKw0KICBsYWJzKHg9IkVmZmVjdCBzaXplIChJQ0MpIikNCg0KZmlnX2Vjb19ncm91cA0KDQojIGZvciBGaWd1cmUgMg0KDQpkIDwtIGdncGxvdChkYXRhID0gbXIzX21vZF90YWJsZSwgYWVzKHggPSBlc3RpbWF0ZSwgeSA9IG5hbWUpKSArIA0KICAgIHNjYWxlX3hfY29udGludW91cyhsaW1pdHMgPSBjKC0wLjM3LDEpLCBicmVha3MgPSBzZXEoLTAuNCwgMSwgYnkgPSAwLjIpLCBsYWJlbHMgPSBjKCItMC40IiwiLTAuMiIsICIwLjAiLCAiMC4yIiwgIjAuNCIsICIwLjYiLCAiMC44IiwgIjEuMCIpKSArIA0KICAgIGdnYmVlc3dhcm06Omdlb21fcXVhc2lyYW5kb20oZGF0YSA9IG1yM19kYXRhLCBhZXMoeCA9IHlpX0lDQywgeSA9IG1vZGVyYXRvciwgc2l6ZSA9IHNjYWxlLCBjb2xvdXI9bW9kZXJhdG9yKSwgZ3JvdXBPblg9RkFMU0UsIGFscGhhID0gMC40KSArDQogICAgZ2VvbV9lcnJvcmJhcmgoYWVzKHhtaW4gPSBsb3dlclBSLCB4bWF4ID0gdXBwZXJQUiksIGhlaWdodCA9IDAsIHNob3cubGVnZW5kID0gRiwgc2l6ZSA9IDAuNSwgYWxwaGEgPSAwLjYpICsgIyBDSQ0KICAgIGdlb21fZXJyb3JiYXJoKGFlcyh4bWluID0gbG93ZXJDTCwgeG1heCA9IHVwcGVyQ0wpLCBoZWlnaHQgPSAwLCBzaG93LmxlZ2VuZCA9IEYsIHNpemUgPSAxLjIpICsgDQogICAgZ2VvbV92bGluZSh4aW50ZXJjZXB0ID0gMCwgbGluZXR5cGUgPSAyLCBjb2xvdXIgPSAiYmxhY2siLCBhbHBoYSA9IDAuNSkgKyAjIGNyZWF0aW5nIGRvdHMgYW5kIGRpZmZlcmVudCBzaXplIChiZWUtc3dhcm0gYW5kIGJ1YmJsZXMpDQogICAgZ2VvbV9wb2ludChhZXMoZmlsbD1uYW1lKSwgc2l6ZSA9IDMsIHNoYXBlID0gMjEpICsgDQogICAgI2dncGxvdDI6OmFubm90YXRlKCJ0ZXh0IiwgeCA9IG1heChtcjJfZGF0YSR5aV9JQ0MpICsgKG1heChtcjJfZGF0YSR5aV9JQ0MpKjAuMTApLCB5ID0gKHNlcSgxLCBtcjJfZ3JvdXBfbm8sIDEpKzAuMyksbGFiZWwgPSBwYXN0ZSgiaXRhbGljKGspPT0iLCBtcjJfbW9kX3RhYmxlJEspLCBwYXJzZSA9IFRSVUUsIGhqdXN0ID0gInJpZ2h0Iiwgc2l6ZSA9IDMuNSkgKw0KICAgIGFubm90YXRlKCd0ZXh0JywgeCA9IDAuOTMsIHkgPSAoc2VxKDEsIG1yM19ncm91cF9ubywgMSkrMC4zNSksIGxhYmVsPSBwYXN0ZSgiaXRhbGljKGspPT0iLCBtcjNfbW9kX3RhYmxlJEspLCBwYXJzZT1UUlVFLCBoanVzdCA9ICJsZWZ0Iiwgc2l6ZT0zLjUpICsNCiAgICBnZ3Bsb3QyOjp0aGVtZV9idygpICsNCiAgICBnZ3Bsb3QyOjpndWlkZXMoZmlsbD0ibm9uZSIsIGNvbG91cj0ibm9uZSIpICsNCiAgICBnZ3Bsb3QyOjp0aGVtZShheGlzLnRleHQueSA9IGVsZW1lbnRfdGV4dChzaXplPTksIGNvbG91cj0iYmxhY2siLCBoanVzdD0wLjUsIGFuZ2xlPTkwKSwNCiAgICAgICAgICAgICAgICAgICBsZWdlbmQucG9zaXRpb24gPSAibm9uZSIsIGF4aXMudGl0bGUgPSBlbGVtZW50X2JsYW5rKCkpICsNCiAgICAjIHNldHRpbmcgY29sb3Vycw0KICBzY2FsZV9jb2xvcl9tYW51YWwodmFsdWVzID0gYygiU2VhYmlyZCIgPSAiIzAwOUU3MyIsICAiTGFuZGJpcmQiID0gIiNENTVFMDAiLCAgIldhdGVyYmlyZCI9ICIjMDA3MkIyIikpICsNCiAgc2NhbGVfZmlsbF9tYW51YWwodmFsdWVzID0gYygiU2VhYmlyZCIgPSAiIzAwOUU3MyIsICAiTGFuZGJpcmQiID0gIiNENTVFMDAiLCAgIldhdGVyYmlyZCI9ICIjMDA3MkIyIikpICsNCiAgICBhbm5vdGF0aW9uX2N1c3RvbShncmlkOjpyYXN0ZXJHcm9iKGltYWdlX3dhdGVyYmlyZCksIHhtaW4gPSAtMC40NSwgeG1heCA9IC0wLjI1LCB5bWluID0gMi43LCB5bWF4ID0gMy4zKSArDQogICAgYW5ub3RhdGlvbl9jdXN0b20oZ3JpZDo6cmFzdGVyR3JvYihpbWFnZV9zZWFiaXJkKSwgeG1pbiA9IC0wLjQ1LCB4bWF4ID0gLTAuMjUsIHltaW4gPSAxLjcsIHltYXggPSAyLjMpICsNCiAgICBhbm5vdGF0aW9uX2N1c3RvbShncmlkOjpyYXN0ZXJHcm9iKGltYWdlX2xhbmRiaXJkKSwgeG1pbiA9IC0wLjQ1LCB4bWF4ID0gLTAuMjUsIHltaW4gPSAwLjcsIHltYXggPSAxLjMpDQoNCmBgYA0KDQoqKkZpZ3VyZSAyZC4qKiBSZXBlYXRhYmlsaXR5IG9mIGF2aWFuIG1pZ3JhdGlvbiB0aW1pbmcgZm9yIGVjb2xvZ2ljYWwgZ3JvdXAuIFRoZSBwbG90IHNob3dzIHRoZSBncm91cC13aXNlIG1lYW5zIHdpdGggOTUlIGNvbmZpZGVuY2UgaW50ZXJ2YWxzICh0aGljayBsaW5lcykgYW5kIDk1JSBwcmVkaWN0aW9uIGludGVydmFscyAodGhpbiBsaW5lcyksIG9ic2VydmVkIGVmZmVjdCBzaXplcyAoYmFjay10cmFuc2Zvcm1lZCB0byBJQ0MpIHNjYWxlZCBieSBwcmVjaXNpb24gKGNpcmNsZXMpIGFuZCBrID0gbnVtYmVyIG9mIGVmZmVjdCBzaXplcy4NCg0KIyMjIyBQdXR0aW5nIHRvZ2V0aGVyIEZpZ3VyZSAyDQoNCmBgYHtyIGZpZzJ9DQoNCiMgYnVpbGRpbmcgZmlnIDIgdXNpbmcgcGF0Y2h3b3JrDQoNCiNGaWd1cmUyIDwtIChhIC8gYiAvIGMgLyBkIC8gZSArIHBsb3RfbGF5b3V0KGhlaWdodHMgPSBjKDEuOCwgMy43LCAzLCAzLCAzKSkgKyBwbG90X2Fubm90YXRpb24odGFnX2xldmVscyA9ICdhJywgdGFnX3N1ZmZpeCA9ICcpJykpDQoNCiNGaWd1cmUyDQoNCiNnZ3NhdmUoaGVyZSgnZmlncy9GaWd1cmVfMl9SX3plcm8ucGRmJyksIHdpZHRoID0gMTgwLCBoZWlnaHQgPSAzMDAsIHVuaXRzPWMoJ21tJyksIGRwaT02MDApIA0KI2VkaXRlZCB0aGlzIGZpbGUgaW4gQWZmaW5pdHkgRGVzaWduZXINCg0KYGBgDQoNCiFbXShDOi9Vc2Vycy9yc3QxOHZ6dS9PbmVEcml2ZSAtIFVuaXZlcnNpdHkgb2YgRWFzdCBBbmdsaWEvUGhEL0xpdC4gcmV2aWV3L01ldGEtYW5hbHlzaXMvQXZpYW5fbWlncmF0aW9uX21ldGEtYW5hbHlzaXMvZmlncy9GaWd1cmVfMl9SX3plcm9fZmluYWwuanBnKXt3aWR0aD04MCV9DQoNCioqRmlndXJlIDIuKiogUHV0dGluZyBhbGwgZml2ZSBwYW5lbHMgdG9nZXRoZXI6IEZpZ3VyZSAyYSAtIEZpZ3VyZSAyZSAoc2VlIHRoZSBtYWluIHRleHQpLiBOb3RlIHRoaXMgcGxvdCB3YXMgZWRpdGVkIGluIEFmZmluaXR5IERlc2lnbmVyIGFmdGVyIGNvbnN0cnVjdGlvbiBpbiBSLg0KDQojIyMjIFRoZSBlZmZlY3Qgb2YgbnVtYmVyIG9mIG9ic2VydmF0aW9ucyBwZXIgaW5kaXZpZHVhbCAoaykNCg0KYGBge3IgbWV0YS1yZWdyZXNzaW9uLWt9DQoNCm1ldGFfcmVncmVzc2lvbjUgPC0gcm1hLm12KHlpID0gWnIyLCBWID0gVkNWLCANCiAgICAgICAgICAgICAgICAgICAgICAgICAgICBtb2RzID0gfiBrLA0KICAgICAgICAgICAgICAgICAgICAgICAgICAgIHJhbmRvbSA9IGxpc3QofjEgfCBlc19JRCwgDQogICAgICAgICAgICAgICAgICAgICAgICAgICAgICAgICAgICAgICAgICB+MSB8IHBhcGVyX0lELCANCiAgICAgICAgICAgICAgICAgICAgICAgICAgICAgICAgICAgICAgICAgIH4xIHwgY29ob3J0X0lELCANCiAgICAgICAgICAgICAgICAgICAgICAgICAgICAgICAgICAgICAgICAgIH4xIHwgc3BlY2llc19JRCwgDQogICAgICAgICAgICAgICAgICAgICAgICAgICAgICAgICAgICAgICAgICB+MSB8IHBoeWxvZ2VueSksDQogICAgICAgICAgICAgICAgICAgICAgICAgICAgUiA9IGxpc3QocGh5bG9nZW55ID0gdmFyY29yKSwNCiAgICAgICAgICAgICAgICAgICAgICAgICAgICBkYXRhID0gZGYpDQpgYGANCg0KIyMjIyBUYWJsZSBTOA0KUmVncmVzc2lvbiBjb2VmZmljaWVudHMgKEVzdGltYXRlKSBhbmQgOTUlIGNvbmZpZGVuY2UgaW50ZXJ2YWxzIChDSXMpIGluIFpyIGZyb20gdGhlIG1ldGEtcmVncmVzc2lvbiB3aXRoIGBrYCAobnVtYmVyIG9mIG9ic2VydmF0aW9ucyBwZXIgaW5kaXZpZHVhbCkuICpSPHN1cD4yPC9zdXA+PHN1Yj5tYXJnaW5hbDwvc3ViPiogPSBgciByb3VuZChvcmNoYVJkOjpyMl9tbChtZXRhX3JlZ3Jlc3Npb241KVtbMV1dKjEwMCwxKWAlLg0KDQpgYGB7ciBrLXRhYmxlfQ0KIyBnZXR0aW5nIG1hcmdpbmFsIFIyDQpyMl9tZXRhX3JlZ3Jlc3Npb241IDwtIHIyX21sKG1ldGFfcmVncmVzc2lvbjUpDQoNCiMgY3JlYXRpbmcgYSB0YWJsZQ0KdGliYmxlKA0KICBgRml4ZWQgZWZmZWN0YCA9IGMoIkludGVyY2VwdCIsICJrIiksDQogIGBFc3RpbWF0ZWAgPSBjKG1ldGFfcmVncmVzc2lvbjUkYiksDQogIGBMb3dlciBDSSBbMC4wMjVdYCA9IGMobWV0YV9yZWdyZXNzaW9uNSRjaS5sYiksDQogIGBVcHBlciBDSSAgWzAuOTc1XWAgPSBjKG1ldGFfcmVncmVzc2lvbjUkY2kudWIpKSAtPiB0X2sNCiMgIGBSMmAgPSBjKHIyX21ldGFfcmVncmVzc2lvbjVbMV0sIE5BKSkgLT4gdF9rDQoNCnRfayAlPiUga2FibGUoImh0bWwiLCBkaWdpdHMgPSAzKSAlPiUNCiAga2FibGVfc3R5bGluZygic3RyaXBlZCIsIHBvc2l0aW9uID0gImxlZnQiKSANCg0KYGBgDQoNCiMjIyMgRmlndXJlIFM0DQpgYGB7ciBmaWd1cmVTNCwgZmlnLndpZHRoPTcsIGZpZy5oZWlnaHQ9IDR9DQpwcmVkX21ldGFfcmVncmVzc2lvbjUgPC0gcHJlZGljdC5ybWEobWV0YV9yZWdyZXNzaW9uNSkNCg0KIyBjcmVhdGluZyBidWJibGUgcGxvdA0KZGYgJT4lIG11dGF0ZShmaXQ9cHJlZF9tZXRhX3JlZ3Jlc3Npb241JHByZWQsIA0KICAgICAgICAgICAgICAgY2kubGI9cHJlZF9tZXRhX3JlZ3Jlc3Npb241JGNpLmxiLA0KICAgICAgICAgICAgICAgY2kudWI9cHJlZF9tZXRhX3JlZ3Jlc3Npb241JGNpLnViLA0KICAgICAgICAgICAgICAgcHIubGI9cHJlZF9tZXRhX3JlZ3Jlc3Npb241JGNyLmxiLA0KICAgICAgICAgICAgICAgcHIudWI9cHJlZF9tZXRhX3JlZ3Jlc3Npb241JGNyLnViKSAlPiUgDQpnZ3Bsb3QoYWVzKHggPSBrLCB5ID0gWnIyKSkgKw0KICAgICBnZW9tX3BvaW50KGFlcyhzaXplPSgxL3NxcnQoVlpyKSkpLCBzaGFwZT0yMSwgYWxwaGE9MC41LCBmaWxsID0gImdyZXk4NSIsIGNvbG91cj0iZ3JleTYwIiwgY29sPSJncmF5MjUiLHN0cm9rZT0xKSArDQogICAgIGdlb21fbGluZShhZXMoeSA9IGZpdCksIHNpemUgPSAxLjUsIGNvbG91cj0iZGFya29yY2hpZDQiKSsNCiAgZ2VvbV9yaWJib24oYWVzKHltaW4gPSBjaS5sYiwgeW1heCA9IGNpLnViLCBjb2xvciA9IE5VTEwpLCBhbHBoYSA9IC4zLCBmaWxsPSJkYXJrb3JjaGlkNCIpICsNCiAgbGFicyh4ID0gIk51bWJlciBvZiBvYnNlcnZhdGlvbnMgcGVyIGluZGl2aWR1YWwgKGspIiwgeSA9ICJFZmZlY3Qgc2l6ZSAoWnIpIiwgc2l6ZSA9ICJQcmVjaXNvbiAoMS9TRSkiKSArDQogIHRoZW1lX2J3KCkgKw0KICBzY2FsZV9zaXplX2NvbnRpbnVvdXMocmFuZ2U9YygxLDEyKSkrDQogIGdlb21faGxpbmUoeWludGVyY2VwdCA9IDAsbGluZXR5cGUgPSAyLCBjb2xvdXIgPSAiYmxhY2siLGFscGhhPTAuNSkgKw0KICB0aGVtZSh0ZXh0ID0gZWxlbWVudF90ZXh0KHNpemUgPSA5LCBjb2xvdXIgPSAiYmxhY2siLCBoanVzdCA9IDAuNSksDQogICAgICAgICAgbGVnZW5kLnRleHQ9ZWxlbWVudF90ZXh0KHNpemU9OCksDQogICAgICAgICAgbGVnZW5kLnBvc2l0aW9uPWMoMSwxKSwgDQogICAgICAgICAgbGVnZW5kLmp1c3RpZmljYXRpb24gPSBjKDEsMSksDQogICAgICAgICAgbGVnZW5kLmJhY2tncm91bmQgPSBlbGVtZW50X2JsYW5rKCksIA0KICAgICAgICAgIGxlZ2VuZC5kaXJlY3Rpb249Imhvcml6b250YWwiLA0KICAgICAgICAgIGxlZ2VuZC50aXRsZSA9IGVsZW1lbnRfdGV4dChzaXplPTgpDQogICAgICAgICkNCg0KYGBgDQoNCioqRmlndXJlIFM0LioqIFJlcGVhdGFiaWxpdHkgb2YgYXZpYW4gbWlncmF0aW9uIHRpbWluZyBmb3IgdGhlIGNvbnRpbnVvdXMgdmFyaWFibGUgYGtgLCB3aGVyZSB0aGUgc29saWQgbGluZSByZXByZXNlbnRzIHRoZSBtb2RlbCBlc3RpbWF0ZSBhbmQgdGhlIHNoYWRpbmcgc2hvd3MgdGhlIDk1JSBjb25maWRlbmNlIGludGVydmFscywgd2l0aCBpbmRpdmlkdWFsIGRhdGEgcG9pbnRzIHNjYWxlZCBieSBwcmVjaXNpb24gKDEvU0UpLg0KDQpgYGB7ciBtZXRhLXJlZ3Jlc3Npb24tb3V0bGllci1rfQ0KZGYyIDwtIGRmICU+JSBmaWx0ZXIoayA8IDEwKQ0KVkNWMiA8LSBpbXB1dGVfY292YXJpYW5jZV9tYXRyaXgodmkgPSBkZjIkVlpyLCBjbHVzdGVyID0gZGYyJGNvaG9ydF9JRCwgciA9IDAuNSkNCg0KbWV0YV9yZWdyZXNzaW9uNiA8LSBybWEubXYoeWkgPSBacjIsIFYgPSBWQ1YyLCANCiAgICAgICAgICAgICAgICAgICAgICAgICAgIG1vZHMgPSB+IGssDQogICAgICAgICAgICAgICAgICAgICAgICAgICByYW5kb20gPSBsaXN0KH4xIHwgZXNfSUQsIA0KICAgICAgICAgICAgICAgICAgICAgICAgICAgICAgICAgICAgICAgICB+MSB8IHBhcGVyX0lELCANCiAgICAgICAgICAgICAgICAgICAgICAgICAgICAgICAgICAgICAgICAgfjEgfCBjb2hvcnRfSUQsIA0KICAgICAgICAgICAgICAgICAgICAgICAgICAgICAgICAgICAgICAgICB+MSB8IHNwZWNpZXNfSUQsIA0KICAgICAgICAgICAgICAgICAgICAgICAgICAgICAgICAgICAgICAgICB+MSB8IHBoeWxvZ2VueSksDQogICAgICAgICAgICAgICAgICAgICAgICAgICBSID0gbGlzdChwaHlsb2dlbnkgPSB2YXJjb3IpLCAjIGFkZGVkIGluIHBoeWxvZ25leQ0KICAgICAgICAgICAgICAgICAgICAgICAgICAgZGF0YSA9IGRmMikNCmBgYA0KDQpXaGVuIHdlIHJlbW92ZSB0aGUgdHdvIHBvaW50cyB3aXRoIGhpZ2ggYGtgIHZhbHVlcyAoayA9IDEyLjQpLCB0aGUgcmVzdWx0cyBhcmUgc2ltaWxhci4gVGhlcmUgaXMgbm8gc2lnbmlmaWNhbnQgZWZmZWN0IChzbG9wZSA9IGByIHJvdW5kKG1ldGFfcmVncmVzc2lvbjYkYlsyLDFdLDMpYCwgOTUlIENJID0gW2ByIHJvdW5kKG1ldGFfcmVncmVzc2lvbjYkY2kubGJbMl0sMylgLCBgciByb3VuZChtZXRhX3JlZ3Jlc3Npb242JGNpLnViWzJdLDMpYF07ICpSPHN1cD4yPC9zdXA+PHN1Yj5tYXJnaW5hbDwvc3ViPiogPSBgciByb3VuZChvcmNoYVJkOjpyMl9tbChtZXRhX3JlZ3Jlc3Npb242KVtbMV1dKjEwMCwxKWAlKSBvZiAnaycgb24gZWZmZWN0IHNpemVzLCBzaG93aW5nIHRoYXQgcmVwZWF0YWJpbGl0eSBkb2VzIG5vdCB2YXJ5IHdpdGggdGhlIG51bWJlciBvZiBvYnNlcnZhdGlvbnMgcGVyIGluZGl2aWR1YWwuDQoNCg0KIyMjIE1vZGVsIHNlbGVjdGlvbiAobXVsdGktcHJlZGljdG9yIG1vZGVsKQ0KDQpIZXJlIHdlIHVzZWQgdGhlIGBNdU1pbmAgcGFja2FnZSB0byBnZW5lcmF0ZSBhbGwgcG9zc2libGUgbW9kZXJhdG9yIGNvbWJpbmF0aW9ucyAodXNpbmcgYWxsIGZpdmUgdmFyaWFibGVzOiBgYW5udWFsX2V2ZW50YCwgYG1ldGhvZGAsIGBzZXhgLCBgdGF4YWAgJiBga2AgKG51bWJlciBvZiBvYnNlcnZhdGlvbnMgcGVyIGluZGl2aWR1YWxzKSksIGRldGVybWluZSB0aGUgaW1wb3J0YW5jZSBvZiB0aGUgbW9kZXJhdG9ycywgYW5kIGdlbmVyYXRlIG1vZGVsLWF2ZXJhZ2VkIGVzdGltYXRlcy4NCg0KYGBge3IgbW9kZWwtc2VsZWN0aW9ufQ0KZXZhbChtZXRhZm9yOjo6Lk11TUluKQ0KDQpmdWxsX21vZGVsX011TUluIDwtIHJtYS5tdih5aSA9IFpyMiwgViA9IFZDViwgDQogICAgICAgICAgICAgICAgICAgICAgICAgIG1vZHMgPSB+IG1ldGhvZCArIA0KICAgICAgICAgICAgICAgICAgICAgICAgICAgIHRheGEgKyANCiAgICAgICAgICAgICAgICAgICAgICAgICAgICBzZXggKyANCiAgICAgICAgICAgICAgICAgICAgICAgICAgICBhbm51YWxfZXZlbnQgKw0KICAgICAgICAgICAgICAgICAgICAgICAgICAgIGssIA0KICAgICAgICAgICAgICAgICAgICAgICAgICByYW5kb20gPSBsaXN0KH4xIHwgZXNfSUQsIA0KICAgICAgICAgICAgICAgICAgICAgICAgICAgICAgICAgICAgICAgIH4xIHwgcGFwZXJfSUQsIA0KICAgICAgICAgICAgICAgICAgICAgICAgICAgICAgICAgICAgICAgIH4xIHwgY29ob3J0X0lELCANCiAgICAgICAgICAgICAgICAgICAgICAgICAgICAgICAgICAgICAgICB+MSB8IHNwZWNpZXNfSUQsIA0KICAgICAgICAgICAgICAgICAgICAgICAgICAgICAgICAgICAgICAgIH4xIHwgcGh5bG9nZW55KSwNCiAgICAgICAgICAgICAgICAgICAgICAgICAgUiA9IGxpc3QocGh5bG9nZW55ID0gdmFyY29yKSwgDQogICAgICAgICAgICAgICAgICAgICAgICAgIG1ldGhvZCA9ICJNTCIsICMgbWF4aW11bSBsaWtlbGlob29kIGZvciBtb2RlbCBzZWxlY3Rpb24NCiAgICAgICAgICAgICAgICAgICAgICAgICAgZGF0YSA9IGRmKQ0KDQojIHZpZi5ybWEoZnVsbF9tb2RlbF9NdU1JbikgICMgTm8gbWFqb3IgcHJvYmxlbXMgb2YgY29sbGluZWFyaXR5IChWSUYgPDQpDQoNCmNhbmRpZGF0ZV9tb2RlbHM8LWRyZWRnZShmdWxsX21vZGVsX011TUluKSAjIEdlbmVyYXRlIGFsbCBwb3NzaWJsZSBjb21iaW5hdGlvbnMgb2YgbW9kZXJhdG9ycw0KDQpjYW5kaWRhdGVzX2FpYzIgPC0gc3Vic2V0KGNhbmRpZGF0ZV9tb2RlbHMsIGRlbHRhPD0yKSAjIERpc3BsYXkgYWxsIG1vZGVscyB3aXRoaW4gMiB2YWx1ZXMgb2YgQUlDYw0KDQppbXBvcnRhbmNlIDwtIHN3KG1vZGVsLmF2ZyhjYW5kaWRhdGVfbW9kZWxzLCBzdWJzZXQ9ZGVsdGE8PTIpKSMgcmVsYXRpdmUgaW1wb3J0YW5jZSAoc3VtIG9mIHdlaWdodHMpIG9mIHRoZSBtb2RlcmF0b3JzDQoNCm1vZC5hdmcgPC0gc3VtbWFyeShtb2RlbC5hdmcoY2FuZGlkYXRlX21vZGVscywgc3Vic2V0PWRlbHRhPD0yKSkgIyBHZW5lcmF0ZSBtb2RlbC1hdmVyYWdlZCBlc3RpbWF0ZXMgDQoNCmNvbmZpZGVuY2UgPC0gY29uZmludChtb2QuYXZnLCBmdWxsPVRSVUUpICMgR2VuZXJhdGUgY29uZmlkZW5jZSBpbnRlcnZhbHMgZm9yIHRoZSBlc3RpbWF0ZXMgYXZlcmFnZWQgdXNpbmcgZnVsbC1hdmVyYWdlcyBwcm9jZWR1cmVzDQoNCmBgYA0KDQojIyMjIFRhYmxlIFM5DQpUaGUgdG9wIGZpdmUgbW9kZWxzIHdpdGhpbiB0aGUgJFxEZWx0YSRBSUMgZGlmZmVyZW5jZSBvZiAyLCBhbmQgd2hpY2ggZml2ZSB2YXJpYWJsZXM6IGBhbm51YWxfZXZlbnRgLCBgbWV0aG9kYCwgYHRheGFgLCBgc2V4YCwgJiBga2Agd2VyZSBpbmNsdWRlZCAoaW5kaWNhdGVkIGJ5ICQrJCk7IG1vZGVsIHdlaWdodHMgYW5kIHRoZSBzdW0gb2Ygd2VpZ2h0cyBmb3IgZWFjaCBvZiB0aGUgdmFyaWFibGVzIGFyZSBpbmNsdWRlZC4gDQoNCmBgYHtyIG1vZGVsLXNlbGVjdGlvbi10YWJsZX0NCiMgY3JlYXRpbmcgYSB0YWJsZQ0KdGliYmxlKA0KICBgTW9kZWwgKHZhcmlhYmxlIHdlaWdodClgID0gYygiTW9kZWwxIiwgIk1vZGVsMiIsICJNb2RlbDMiLCAiTW9kZWw0IiwgIk1vZGVsNSIsICIoU3VtIG9mIHdlaWdodHMpIiksDQogIGFubnVhbF9ldmVudCA9IGMoaWZfZWxzZShjYW5kaWRhdGVzX2FpYzIkYW5udWFsX2V2ZW50ID09ICIrIiwgIiQrJCIsICJOQSIpLHJvdW5kKGltcG9ydGFuY2VbMV0sMykgKSwNCiAgdGF4YSA9ICBjKGlmX2Vsc2UoY2FuZGlkYXRlc19haWMyJHRheGE9PSAiKyIsICIkKyQiLCAiTkEiKSxyb3VuZChpbXBvcnRhbmNlWzJdLCAzKSksDQogIG1ldGhvZCA9IGMoaWZfZWxzZShjYW5kaWRhdGVzX2FpYzIkbWV0aG9kPT0gIisiLCAiJCskIiwgIk5BIikscm91bmQoaW1wb3J0YW5jZVszXSwgMykpLA0KICBrID0gYyhpZl9lbHNlKGNhbmRpZGF0ZXNfYWljMiRrIDw9IDAsICIkKyQiLCAiTkEiKSxyb3VuZChpbXBvcnRhbmNlWzRdLCAzKSksDQogIHNleCA9IGMoaWZfZWxzZShjYW5kaWRhdGVzX2FpYzIkc2V4PT0gIisiLCAiJCskIiwgIk5BIikscm91bmQoaW1wb3J0YW5jZVs1XSwgMykpLA0KICBkZWx0YV9BSUNjID0gYyhjYW5kaWRhdGVzX2FpYzIkZGVsdGEsIE5BKSwNCiAgV2VpZ2h0ID0gYyhjYW5kaWRhdGVzX2FpYzIkd2VpZ2h0LCBOQSkpICU+JSANCiAga2FibGUoImh0bWwiLCBkaWdpdHMgPSAzKSAlPiUNCiAga2FibGVfc3R5bGluZygic3RyaXBlZCIsIHBvc2l0aW9uID0gImxlZnQiKQ0KYGBgDQoNCiMjIyMgTW9kZWwgYXZlcmFnaW5nDQoNCiMjIyMgVGFibGUgUzEwDQpUaGUgYXZlcmFnZSBlc3RpbWF0ZXMgZm9yIHJlZ3Jlc3Npb24gY29lZmZpY2llbnRzIChFc3RpbWF0ZSkgYW5kIDk1JSBjb25maWRlbmNlIGludGVydmFscyAoQ0lzKSBmcm9tIHRoZSBtb2RlbCBhdmVyYWdpbmcgcHJvY2VkdXJlIHVzaW5nIGZ1bGwtYXZlcmFnZXMgKGFzc3VtaW5nIHplcm8gdmFsdWVzIGZvciBtb2RlcmF0b3JzIHdoZW4gdGhleSBkbyBub3Qgb2NjdXIpLg0KYGBge3IgbW9kZWwtYXZlcmFnaW5nfQ0KIyBjcmVhdGluZyBhIHRhYmxlDQp0aWJibGUoDQogIGBGaXhlZCBlZmZlY3RgID0gYygiSW50ZXJjZXB0IiwNCiAgICAgICAgICAgICAgICAgICAgICJEZXBhcnRfYnJlZWQiLCAiTm9uYnJlZWRfYXJyaXZhbCIsICJOb25icmVlZF9kZXBhcnQiLCANCiAgICAgICAgICAgICAgICAgICAgICJGZW1hbGUiLCJNYWxlIiwgIlNlYWJpcmQiLCAiV2F0ZXJiaXJkIiwgIkdMUyIsICJTYXRlbGxpdGUiLA0KICAgICAgICAgICAgICAgICAgICAgImsiKSwNCiAgRXN0aW1hdGUgPSBtb2QuYXZnJGNvZWZtYXQuZnVsbFssMV0sDQogIGBMb3dlciBDSSBbMC4wMjVdYCA9IGNvbmZpZGVuY2VbLDFdLA0KICBgVXBwZXIgQ0kgIFswLjk3NV1gID0gIGNvbmZpZGVuY2VbLDJdKSAlPiUgDQogIGthYmxlKCJodG1sIiwgZGlnaXRzID0gMykgJT4lDQogIGthYmxlX3N0eWxpbmcoInN0cmlwZWQiLCBwb3NpdGlvbiA9ICJsZWZ0IikNCmBgYA0KDQojIyBTZW5zaXRpdml0eSBBbmFseXNpcw0KDQojIyMgVmFyaWFuY2UtY292YXJpYW5jZSBtYXRyaXggd2l0aCBkaWZmZXJlbnQgbGV2ZWxzIG9mIGNvcnJlbGF0aW9uDQpNdWx0aXBsZSByZXBlYXRhYmlsaXR5IGVzdGltYXRlcyB3ZXJlIG1lYXN1cmVkIG9uIHRoZSBzYW1lIGFuaW1hbHMgd2l0aGluIGEgcGFwZXIgKGNvaG9ydCBJRCkgd2hpY2ggaW5kdWNlcyBhIGNvcnJlbGF0aW9uIGJldHdlZW4gc2FtcGxpbmcgZXJyb3IgdmFyaWFuY2VzIFtAbm9ibGVfbm9uaW5kZXBlbmRlbmNlXzIwMTddLiBUaHVzLCB3ZSBjb25zdHJ1Y3RlZCB2YXJpYW5jZS1jb3ZhcmlhbmNlIG1hdHJpY2VzIHRvIG1vZGVsIHNoYXJlZCBzYW1wbGluZyBlcnJvciBmb3IgZWZmZWN0IHNpemVzIGZyb20gdGhlIHNhbWUgY29ob3J0LiBXZSBpbml0aWFsbHkgYXNzdW1lZCBhIDAuNSBjb3JyZWxhdGlvbiwgYnV0IGFsc28gcmFuIHRoZSBwaHlsb2dlbmV0aWMgbWV0YS1hbmFseXRpYyBtb2RlbCB3aXRoIGEgMC4yNSBhbmQgMC43NSBjb3JyZWxhdGlvbi4gQWxsIHRocmVlIGNvcnJlbGF0aW9ucyB5aWVsZGVkIHF1YWxpdGF0aXZlbHkgc2ltaWxhciByZXN1bHRzLCB0aHVzIHRocm91Z2hvdXQgdGhlIG1hbnVzY3JpcHQgd2UgYXNzdW1lIGEgMC41IGNvcnJlbGF0aW9uLCBidXQgdGhlIHJlc3VsdHMgZm9yIHRoZSBvdGhlciBjb3JyZWxhdGlvbiB2YWx1ZXMgYXJlIHByZXNlbnRlZCBiZWxvdy4gDQoNCiMjIyMgVGFibGUgUzExDQpPdmVyYWxsIGVmZmVjdHMgKG1ldGEtYW5hbHl0aWMgbWVhbnMpIGFuZCA5NSUgY29uZmlkZW5jZSBpbnRlcnZhbHMgKENJcykgaW4gKlpyKiBhbmQgaGV0ZXJvZ2VuZWl0eSwgKkkqXjJeLCBmb3IgdGhlIHBoeWxvZ2VuZXRpYyBtdWx0aWxldmVsIGludGVyY2VwdC1vbmx5IG1ldGEtYW5hbHlzaXMgbW9kZWwgd2hlbiB0ZXN0aW5nIGRpZmZlcmVudCBsZXZlbHMgb2YgY29ycmVsYXRpb24gKHIgPSAwLjI1LCAwLjUwLCBhbmQgMC43NSkgYmV0d2VlbiBzYW1wbGluZyB2YXJpYW5jZXMgZnJvbSB0aGUgc2FtZSBjb2hvcnQgb2YgYmlyZHMuDQoNCmBgYHtyIFZDVi1jb3J9DQojIENyZWF0ZSBhIHZhcmlhbmNlLWNvdmFyaWFuY2UgbWF0cml4IGF0IHRoZSBjb2hvcnQgbGV2ZWwgd2l0aCBkaWZmZXJlbnQgY29ycmVsYXRpb24gdmFsdWVzDQpWQ1ZfMjUgPC0gaW1wdXRlX2NvdmFyaWFuY2VfbWF0cml4KHZpID0gZGYkVlpyLCBjbHVzdGVyID0gZGYkY29ob3J0X0lELCByID0gMC4yNSkNClZDVl83NSA8LSBpbXB1dGVfY292YXJpYW5jZV9tYXRyaXgodmkgPSBkZiRWWnIsIGNsdXN0ZXIgPSBkZiRjb2hvcnRfSUQsIHIgPSAwLjc1KQ0KDQojIFJ1biBwaHlsb2dlbmV0aWMgbWV0YS1hbmFseXRpYyBtb2RlbHMgd2l0aCAwLjI1IGFuZCAwLjc1IFZDVg0KbWFfbW9kZWwyX1ZDVjI1IDwtIHJtYS5tdih5aSA9IFpyMiwgViA9IFZDVl8yNSwgDQogICAgICAgICAgICAgICAgICAgICAgcmFuZG9tID0gbGlzdCh+MSB8IGVzX0lELCANCiAgICAgICAgICAgICAgICAgICAgICAgICAgICAgICAgICAgIH4xIHwgcGFwZXJfSUQsIA0KICAgICAgICAgICAgICAgICAgICAgICAgICAgICAgICAgICAgfjEgfCBjb2hvcnRfSUQsIA0KICAgICAgICAgICAgICAgICAgICAgICAgICAgICAgICAgICAgfjEgfCBzcGVjaWVzX0lELCAjIG5vbi1waHlsbyBlZmZlY3QNCiAgICAgICAgICAgICAgICAgICAgICAgICAgICAgICAgICAgIH4xIHwgcGh5bG9nZW55KSwgIyBwaHlsbyBlZmZlY3QNCiAgICAgICAgICAgICAgICAgICAgICBSID0gbGlzdChwaHlsb2dlbnkgPSB2YXJjb3IpLCAjIHBoeWxvZ2VuZXRpYyByZWxhdGVkbmVzcw0KICAgICAgICAgICAgICAgICAgICAgIGRhdGEgPSBkZikNCg0KaTJfbWEyX1ZDVjI1IDwtIHJvdW5kKGkyX21sKG1hX21vZGVsMl9WQ1YyNSkqMTAwLDEpDQoNCm1hX21vZGVsMl9WQ1Y3NSA8LSBybWEubXYoeWkgPSBacjIsIFYgPSBWQ1ZfNzUsIA0KICAgICAgICAgICAgICAgICAgICAgIHJhbmRvbSA9IGxpc3QofjEgfCBlc19JRCwgDQogICAgICAgICAgICAgICAgICAgICAgICAgICAgICAgICAgICB+MSB8IHBhcGVyX0lELCANCiAgICAgICAgICAgICAgICAgICAgICAgICAgICAgICAgICAgIH4xIHwgY29ob3J0X0lELCANCiAgICAgICAgICAgICAgICAgICAgICAgICAgICAgICAgICAgIH4xIHwgc3BlY2llc19JRCwgIyBub24tcGh5bG8gZWZmZWN0DQogICAgICAgICAgICAgICAgICAgICAgICAgICAgICAgICAgICB+MSB8IHBoeWxvZ2VueSksICMgcGh5bG8gZWZmZWN0DQogICAgICAgICAgICAgICAgICAgICAgUiA9IGxpc3QocGh5bG9nZW55ID0gdmFyY29yKSwgIyBwaHlsb2dlbmV0aWMgcmVsYXRlZG5lc3MNCiAgICAgICAgICAgICAgICAgICAgICBkYXRhID0gZGYpDQoNCmkyX21hMl9WQ1Y3NSA8LSByb3VuZChpMl9tbChtYV9tb2RlbDJfVkNWNzUpKjEwMCwxKQ0KDQojIGNyZWF0aW5nIGEgdGFibGUNCnRpYmJsZSgNCiAgTW9kZWwgPSBjKCJNZXRhLWFuYWx5c2lzLCByPTAuMjUiLCAiTWV0YS1hbmFseXNpcywgcj0wLjUwIiwgIk1ldGEtYW5hbHlzaXMsIHI9MC43NSIpLA0KICBgT3ZlcmFsbCBtZWFuYCA9IGMobWFfbW9kZWwyX1ZDVjI1JGIsIG1hX21vZGVsMiRiLCBtYV9tb2RlbDJfVkNWNzUkYiksDQogIGBMb3dlciBDSSBbMC4wMjVdYCA9IGMobWFfbW9kZWwyX1ZDVjI1JGNpLmxiLCBtYV9tb2RlbDIkY2kubGIsIG1hX21vZGVsMl9WQ1Y3NSRjaS5sYiksDQogIGBVcHBlciBDSSBbMC45NzVdYCA9IGMobWFfbW9kZWwyX1ZDVjI1JGNpLnViLCBtYV9tb2RlbDIkY2kudWIsIG1hX21vZGVsMl9WQ1Y3NSRjaS51YiksDQogIGBJXjJefnRvdGFsfmAgPSBjKGkyX21hMl9WQ1YyNVsxXSwgaTJfbWEyWzFdLCBpMl9tYTJfVkNWNzVbMV0pLA0KICBgSV4yXn5lc35gID0gYyhpMl9tYTJfVkNWMjVbMl0sIGkyX21hMlsyXSwgaTJfbWEyX1ZDVjc1WzJdKSwNCiAgYEleMl5+cGFwZXJ+YCA9IGMoaTJfbWEyX1ZDVjI1WzNdLCBpMl9tYTJbM10sIGkyX21hMl9WQ1Y3NVszXSksDQogIGBJXjJefmNvaG9ydH5gID0gYyhpMl9tYTJfVkNWMjVbNF0sIGkyX21hMls0XSwgaTJfbWEyX1ZDVjc1WzRdKSwNCiAgYEleMl5+c3BlY2llc35gID0gYyhpMl9tYTJfVkNWMjVbNV0sIGkyX21hMls1XSwgaTJfbWEyX1ZDVjc1WzVdKSwNCiAgYEleMl5+cGh5bG9+YCA9IGMoaTJfbWEyX1ZDVjI1WzZdLCBpMl9tYTJbNl0sIGkyX21hMl9WQ1Y3NVs2XSksKSAlPiUgDQogIGthYmxlKCJodG1sIiwgIGRpZ2l0cyA9IDMpICU+JQ0KICBrYWJsZV9zdHlsaW5nKCJzdHJpcGVkIiwgcG9zaXRpb24gPSAibGVmdCIpDQoNCmBgYA0KDQojIyMgSW5jbHVzaW9uIG9mIG5lZ2F0aXZlIHJlcGVhdGFiaWxpeSBlc3RpbWF0ZXMNCkNvcnJlbGF0aW9uLSBhbmQgQU5PVkEtYmFzZWQgcmVwZWF0YWJpbGl0aWVzIGNhbiBwcm9kdWNlIG5lZ2F0aXZlIHZhbHVlcywgb2Z0ZW4gcmVmbGVjdGluZyBub2lzZSBhcm91bmQgYSBzdGF0aXN0aWNhbCB6ZXJvIFtAbmFrYWdhd2FfcmVwZWF0YWJpbGl0eV8yMDEwXS4gRm9yIG91ciBtYWluIGFuYWx5c2VzLCB3ZSBzZXQgdGhlc2UgbmVnYXRpdmUgZXN0aW1hdGVzIHRvIHplcm8sIGhvd2V2ZXIgaGVyZSwgd2UgcmUtcmFuIGFsbCB0aGUgbWV0YS1hbmFseXRpYyBhbmQgbWV0YS1yZWdyZXNzaW9uIG1vZGVscyB3aXRoIHRoZXNlIG5lZ2F0aXZlIHZhbHVlcyBpbmNsdWRlZC4NCg0KIyMjIyBUYWJsZSBTMTINCk92ZXJhbGwgZWZmZWN0cyAobWV0YS1hbmFseXRpYyBtZWFucykgYW5kIDk1JSBjb25maWRlbmNlIGludGVydmFscyAoQ0lzKSBib3RoIGluICpaciogYW5kIGJhY2stdHJhbnNmb3JtZWQgdG8gKklDQyosIGFuZCBoZXRlcm9nZW5laXR5LCAqSSpeMl4sIGZvciB0aGUgbXVsdGlsZXZlbCBpbnRlcmNlcHQtb25seSBtZXRhLWFuYWx5c2lzIG1vZGVscyBpbmNsdWRpbmcgYW5kIGV4Y2x1ZGluZyBwaHlsb2dlbnkgd2hlbiBuZWdhdGl2ZSByZXBlYXRhYmlsaXR5IHZhbHVlcyBhcmUgaW5jbHVkZWQuDQoNCmBgYHtyIG1ldGEtYW5hbHl0aWMtbW9kcy1uZWd9DQoNCm1hX21vZGVsMV9uZWcgPC0gcm1hLm12KHlpID0gWnIsIFYgPSBWQ1YsDQogICAgICAgICAgICAgICAgICAgIHJhbmRvbSA9IGxpc3QofjEgfCBlc19JRCwgDQogICAgICAgICAgICAgICAgICAgICAgICAgICAgICAgICAgfjEgfCBwYXBlcl9JRCwgDQogICAgICAgICAgICAgICAgICAgICAgICAgICAgICAgICAgfjEgfCBjb2hvcnRfSUQsIA0KICAgICAgICAgICAgICAgICAgICAgICAgICAgICAgICAgIH4xIHwgc3BlY2llc19JRCksIA0KICAgICAgICAgICAgICAgICAgICBkYXRhID0gZGYpDQoNCm1hX21vZGVsMl9uZWcgPC0gcm1hLm12KHlpID0gWnIsIFYgPSBWQ1YsIA0KICAgICAgICAgICAgICAgICAgICByYW5kb20gPSBsaXN0KH4xIHwgZXNfSUQsIA0KICAgICAgICAgICAgICAgICAgICAgICAgICAgICAgICAgIH4xIHwgcGFwZXJfSUQsIA0KICAgICAgICAgICAgICAgICAgICAgICAgICAgICAgICAgIH4xIHwgY29ob3J0X0lELCANCiAgICAgICAgICAgICAgICAgICAgICAgICAgICAgICAgICB+MSB8IHNwZWNpZXNfSUQsICMgbm9uLXBoeWxvIGVmZmVjdA0KICAgICAgICAgICAgICAgICAgICAgICAgICAgICAgICAgIH4xIHwgcGh5bG9nZW55KSwgIyBwaHlsbyBlZmZlY3QNCiAgICAgICAgICAgICAgICAgICAgUiA9IGxpc3QocGh5bG9nZW55ID0gdmFyY29yKSwgIyBwaHlsb2dlbmV0aWMgcmVsYXRlZG5lc3MNCiAgICAgICAgICAgICAgICAgICAgZGF0YSA9IGRmKQ0KDQojIyAjIGVzdGltYXRpbmcgSTIgYXMgbWVhc3VyZSBvZiBoZXRlcm9nZW5laXR5DQppMl9tYTFfbmVnIDwtIHJvdW5kKGkyX21sKG1hX21vZGVsMV9uZWcpKjEwMCwxKQ0KaTJfbWEyX25lZyA8LSByb3VuZChpMl9tbChtYV9tb2RlbDJfbmVnKSoxMDAsMSkNCg0KIyBCYWNrLXRyYW5zZm9ybSB0byBJQ0MNCm1hMV9uZWcgPC0gbW9kX3Jlc3VsdHMobWFfbW9kZWwxX25lZywgbW9kPSJJbnQiKQ0KbWExX21vZF90YWJsZV9uZWcgPC0gbWExX25lZyRtb2RfdGFibGUNCg0KbWEyX25lZyA8LSBtb2RfcmVzdWx0cyhtYV9tb2RlbDJfbmVnLCBtb2Q9IkludCIpDQptYTJfbW9kX3RhYmxlX25lZyA8LSBtYTJfbmVnJG1vZF90YWJsZQ0KDQojIG5lZWQgdG8gY2FsY3VsYXRlIGsgZm9yIHdob2xlIGRhdGEgc2V0IHRvIHVzZSBpbiBmb3JtdWxhDQoNCmtfYWxsIDwtIG1lYW4oZGYkaykNCg0KZm9yKGkgaW4gbmFtZXMobWExX21vZF90YWJsZV9uZWcpWzI6Nl0pew0KICANCiAgbWExX21vZF90YWJsZV9uZWdbaV0gPC0gWnJfdG9fSUNDKG1hMV9tb2RfdGFibGVfbmVnW2ldLCBrX2FsbCkNCiAgDQp9DQoNCmZvcihpIGluIG5hbWVzKG1hMl9tb2RfdGFibGVfbmVnKVsyOjZdKXsNCiAgDQogIG1hMl9tb2RfdGFibGVfbmVnW2ldIDwtIFpyX3RvX0lDQyhtYTJfbW9kX3RhYmxlX25lZ1tpXSwga19hbGwpDQogIA0KfQ0KDQojIGNyZWF0aW5nIGEgdGFibGUNCnRpYmJsZSgNCiAgTW9kZWwgPSBjKCJNZXRhLWFuYWx5c2lzIChacikiLCAiTWV0YS1hbmFseXNpcyAoSUNDKSIsICJNZXRhLWFuYWx5c2lzIHBoeWxvIChacikiLCAiTWV0YS1hbmFseXNpcyBwaHlsbyAoSUNDKSIpLA0KICBgT3ZlcmFsbCBtZWFuYCA9IGMobWFfbW9kZWwxX25lZyRiLCBtYTFfbW9kX3RhYmxlX25lZyRlc3RpbWF0ZSwgbWFfbW9kZWwyX25lZyRiLCBtYTJfbW9kX3RhYmxlX25lZyRlc3RpbWF0ZSksDQogIGBMb3dlciBDSSBbMC4wMjVdYCA9IGMobWFfbW9kZWwxX25lZyRjaS5sYiwgbWExX21vZF90YWJsZV9uZWckbG93ZXJDTCwgbWFfbW9kZWwyX25lZyRjaS5sYiwgbWEyX21vZF90YWJsZV9uZWckbG93ZXJDTCksDQogIGBVcHBlciBDSSBbMC45NzVdYCA9IGMobWFfbW9kZWwxX25lZyRjaS51YiwgbWExX21vZF90YWJsZV9uZWckdXBwZXJDTCwgbWFfbW9kZWwyX25lZyRjaS51YiwgbWEyX21vZF90YWJsZV9uZWckdXBwZXJDTCksDQogIGBJXjJefnRvdGFsfmAgPSBjKGkyX21hMV9uZWdbMV0sIE5BLCBpMl9tYTJfbmVnWzFdLCBOQSksDQogIGBJXjJefmVzfmAgPSBjKGkyX21hMV9uZWdbMl0sIE5BLCBpMl9tYTJfbmVnWzJdLCBOQSksDQogIGBJXjJefnBhcGVyfmAgPSBjKGkyX21hMV9uZWdbM10sIE5BLCBpMl9tYTJfbmVnWzNdLCBOQSksDQogIGBJXjJefmNvaG9ydH5gID0gYyhpMl9tYTFfbmVnWzRdLCBOQSwgaTJfbWEyX25lZ1s0XSwgTkEpLA0KICBgSV4yXn5zcGVjaWVzfmAgPSBjKGkyX21hMV9uZWdbNV0sIE5BLCBpMl9tYTJfbmVnWzVdLCBOQSksDQogIGBJXjJefnBoeWxvfmAgPSBjKE5BLCBOQSwgaTJfbWEyX25lZ1s2XSwgTkEpLCkgJT4lIA0KICBrYWJsZSgiaHRtbCIsICBkaWdpdHMgPSAzKSAlPiUNCiAga2FibGVfc3R5bGluZygic3RyaXBlZCIsIHBvc2l0aW9uID0gImxlZnQiKQ0KYGBgDQoNCmBgYHtyIG1ldGEtcmVncmVzc2lvbi1hbm51YWwtZXZlbnQtbmVnfQ0KIyBtZXRhLXJlZ3Jlc3Npb246IG11dGlwbGUgaW50ZXJjZXB0cw0KbWV0YV9yZWdyZXNzaW9uMV9uZWcgPC0gcm1hLm12KHlpID0gWnIsIFYgPSBWQ1YsIA0KICAgICAgICAgICAgICAgICAgICAgICAgICAgbW9kcyA9IH4gYW5udWFsX2V2ZW50LA0KICAgICAgICAgICAgICAgICAgICAgICAgICAgcmFuZG9tID0gbGlzdCh+MSB8IGVzX0lELCANCiAgICAgICAgICAgICAgICAgICAgICAgICAgICAgICAgICAgICAgICAgfjEgfCBwYXBlcl9JRCwgDQogICAgICAgICAgICAgICAgICAgICAgICAgICAgICAgICAgICAgICAgIH4xIHwgY29ob3J0X0lELCANCiAgICAgICAgICAgICAgICAgICAgICAgICAgICAgICAgICAgICAgICAgfjEgfCBzcGVjaWVzX0lELCANCiAgICAgICAgICAgICAgICAgICAgICAgICAgICAgICAgICAgICAgICAgfjEgfCBwaHlsb2dlbnkpLA0KICAgICAgICAgICAgICAgICAgICAgICAgICAgUiA9IGxpc3QocGh5bG9nZW55ID0gdmFyY29yKSwgIyBhZGRlZCBpbiBwaHlsb2duZXkNCiAgICAgICAgICAgICAgICAgICAgICAgICAgIGRhdGEgPSBkZikNCg0KbWV0YV9yZWdyZXNzaW9uMWJfbmVnIDwtIHJtYS5tdih5aSA9IFpyLCBWID0gVkNWLCANCiAgICAgICAgICAgICAgICAgICAgICAgICAgICBtb2RzID0gfiByZWxldmVsKGFubnVhbF9ldmVudCwgcmVmID0gIkRlcGFydF9icmVlZCIpLA0KICAgICAgICAgICAgICAgICAgICAgICAgICAgIHJhbmRvbSA9IGxpc3QofjEgfCBlc19JRCwgDQogICAgICAgICAgICAgICAgICAgICAgICAgICAgICAgICAgICAgICAgICB+MSB8IHBhcGVyX0lELCANCiAgICAgICAgICAgICAgICAgICAgICAgICAgICAgICAgICAgICAgICAgIH4xIHwgY29ob3J0X0lELCANCiAgICAgICAgICAgICAgICAgICAgICAgICAgICAgICAgICAgICAgICAgIH4xIHwgc3BlY2llc19JRCwgDQogICAgICAgICAgICAgICAgICAgICAgICAgICAgICAgICAgICAgICAgICB+MSB8IHBoeWxvZ2VueSksDQogICAgICAgICAgICAgICAgICAgICAgICAgICAgUiA9IGxpc3QocGh5bG9nZW55ID0gdmFyY29yKSwgIyBhZGRlZCBpbiBwaHlsb2duZXkNCiAgICAgICAgICAgICAgICAgICAgICAgICAgICBkYXRhID0gZGYpDQoNCg0KbWV0YV9yZWdyZXNzaW9uMWNfbmVnIDwtIHJtYS5tdih5aSA9IFpyLCBWID0gVkNWLCANCiAgICAgICAgICAgICAgICAgICAgICAgICAgICBtb2RzID0gfiByZWxldmVsKGFubnVhbF9ldmVudCwgcmVmID0gIk5vbmJyZWVkX2RlcGFydCIpLA0KICAgICAgICAgICAgICAgICAgICAgICAgICAgIHJhbmRvbSA9IGxpc3QofjEgfCBlc19JRCwgDQogICAgICAgICAgICAgICAgICAgICAgICAgICAgICAgICAgICAgICAgICB+MSB8IHBhcGVyX0lELCANCiAgICAgICAgICAgICAgICAgICAgICAgICAgICAgICAgICAgICAgICAgIH4xIHwgY29ob3J0X0lELCANCiAgICAgICAgICAgICAgICAgICAgICAgICAgICAgICAgICAgICAgICAgIH4xIHwgc3BlY2llc19JRCwgDQogICAgICAgICAgICAgICAgICAgICAgICAgICAgICAgICAgICAgICAgICB+MSB8IHBoeWxvZ2VueSksDQogICAgICAgICAgICAgICAgICAgICAgICAgICAgUiA9IGxpc3QocGh5bG9nZW55ID0gdmFyY29yKSwgIyBhZGRlZCBpbiBwaHlsb2duZXkNCiAgICAgICAgICAgICAgICAgICAgICAgICAgICBkYXRhID0gZGYpDQoNCiMgbWV0YS1yZWdyZXNzaW9uOiBjb250cmFzdCAoZm9yIG9yY2hhcmQgcGxvdCkNCm1ldGFfcmVncmVzc2lvbjFkX25lZyA8LSBybWEubXYoeWkgPSBaciwgViA9IFZDViwgDQogICAgICAgICAgICAgICAgICAgICAgICAgICAgbW9kcyA9IH4gYW5udWFsX2V2ZW50IC0xLA0KICAgICAgICAgICAgICAgICAgICAgICAgICAgIHJhbmRvbSA9IGxpc3QofjEgfCBlc19JRCwgDQogICAgICAgICAgICAgICAgICAgICAgICAgICAgICAgICAgICAgICAgICB+MSB8IHBhcGVyX0lELCANCiAgICAgICAgICAgICAgICAgICAgICAgICAgICAgICAgICAgICAgICAgIH4xIHwgY29ob3J0X0lELCANCiAgICAgICAgICAgICAgICAgICAgICAgICAgICAgICAgICAgICAgICAgIH4xIHwgc3BlY2llc19JRCwgDQogICAgICAgICAgICAgICAgICAgICAgICAgICAgICAgICAgICAgICAgICB+MSB8IHBoeWxvZ2VueSksDQogICAgICAgICAgICAgICAgICAgICAgICAgICAgUiA9IGxpc3QocGh5bG9nZW55ID0gdmFyY29yKSwgIyBhZGRlZCBpbiBwaHlsb2duZXkNCiAgICAgICAgICAgICAgICAgICAgICAgICAgICBkYXRhID0gZGYpDQpgYGANCg0KIyMjIyBUYWJsZSBTMTMNClJlZ3Jlc3Npb24gY29lZmZpY2llbnRzIChFc3RpbWF0ZSkgYW5kIDk1JSBjb25maWRlbmNlIGludGVydmFscyAoQ0lzKSBib3RoIGluIFpyIGFuZCBiYWNrLXRyYW5zZm9ybWVkIHRvIElDQyBmcm9tIHRoZSBtZXRhLXJlZ3Jlc3Npb24gd2l0aCBgYW5udWFsX2V2ZW50YCB3aGVuIG5lZ2F0aXZlIHJlcGVhdGFiaWxpdHkgdmFsdWVzIGFyZSBpbmNsdWRlZC4gTm90ZSB0aGF0IGBtdWAgbWVhbnMgdGhlIGdyb3VwIG1lYW4gd2hpbGUgYGJldGFgIHJlcHJlc2VudHMgdGhlIGNvbnRyYXN0IGJldHdlZW4gdHdvIGdyb3VwcyBpbiB0aGUgVW5pdCBjb2x1bW4uICpSPHN1cD4yPC9zdXA+PHN1Yj5tYXJnaW5hbDwvc3ViPiogPSBgciByb3VuZChvcmNoYVJkOjpyMl9tbChtZXRhX3JlZ3Jlc3Npb24xX25lZylbWzFdXSoxMDAsMSlgJS4NCg0KYGBge3IgYW5udWFsLWV2ZW50LXRhYmxlLW5lZ30NCg0KIyBnZXR0aW5nIG1hcmdpbmFsIFIyDQpyMl9tZXRhX3JlZ3Jlc3Npb24xX25lZyA8LSByMl9tbChtZXRhX3JlZ3Jlc3Npb24xX25lZykNCg0KIyBnZXR0aW5nIGVzdGltYXRlcw0KIyBpbmNsdWRpbmcgYmFjay10cmFuc2Zvcm1hdGlvbiB0byBJQ0MNCg0KbXIxX25lZyA8LSBtb2RfcmVzdWx0cyhtZXRhX3JlZ3Jlc3Npb24xZF9uZWcsIG1vZD0iYW5udWFsX2V2ZW50IikNCm1yMV9tb2RfdGFibGVfbmVnIDwtIG1yMV9uZWckbW9kX3RhYmxlDQoNCm1yMV9kYXRhX25lZyA8LSBtcjFfbmVnJGRhdGENCg0KIyBjYWxjdWxhdGUgayBmb3IgZWFjaCBtZXRob2Qgc2VwYXJhdGVseQ0KDQojIGRmICU+JSBncm91cF9ieShhbm51YWxfZXZlbnQpICU+JSBzdW1tYXJpc2UobWVhbihrKSkNCg0KbXIxX2RhdGFfbmVnIDwtIG1yMV9kYXRhX25lZyAlPiUgbXV0YXRlKGsgPSBjYXNlX3doZW4obW9kZXJhdG9yID09ICJBcnJpdmFsX2JyZWVkIiB+IDIuNTUsIA0KICAgICAgICAgICAgICAgICAgICAgICAgICAgICAgICAgICAgICAgICAgICAgIG1vZGVyYXRvciA9PSAiRGVwYXJ0X2JyZWVkIiB+IDIuNDUsDQogICAgICAgICAgICAgICAgICAgICAgICAgICAgICAgICAgICAgICAgICAgICAgbW9kZXJhdG9yID09ICJOb25icmVlZF9hcnJpdmFsIiB+IDMuMjAsDQogICAgICAgICAgICAgICAgICAgICAgICAgICAgICAgICAgICAgICAgICAgICAgbW9kZXJhdG9yID09ICJOb25icmVlZF9kZXBhcnQiIH4gMi45NCkpDQoNCm1yMV9kYXRhX25lZyR5aV9JQ0MgPC0gWnJfdG9fSUNDKG1yMV9kYXRhX25lZyR5aSwgbXIxX2RhdGFfbmVnJGspDQoNCm1yMV9tb2RfdGFibGVfbmVnJGsgPC0gYygyLjU1LCAyLjQ1LCAzLjIwLCAyLjk0KQ0KDQpmb3IoaSBpbiBuYW1lcyhtcjFfbW9kX3RhYmxlX25lZylbMjo2XSl7DQogIA0KICBtcjFfbW9kX3RhYmxlX25lZ1tpXSA8LSBacl90b19JQ0MobXIxX21vZF90YWJsZV9uZWdbaV0sIG1yMV9tb2RfdGFibGVfbmVnJGspDQogIA0KfQ0KDQojIGNyZWF0aW5nIGEgdGFibGUNCnRpYmJsZSgNCiAgYEZpeGVkIGVmZmVjdGAgPSBjKHJlcChhcy5jaGFyYWN0ZXIobXIxX21vZF90YWJsZV9uZWckbmFtZSksMiksIGNvbnRfZ2VuKG1yMV9tb2RfdGFibGVfbmVnJG5hbWUpKSwNCiAgYFVuaXRgID0gYyhyZXAoYygiWnIgKG11KSIsICJJQ0MgKG11KSIpLGVhY2ggPSA0KSwgcmVwKCJaciAoYmV0YSkiLCA2KSksDQogIGBFc3RpbWF0ZWAgPSBjKG1ldGFfcmVncmVzc2lvbjFkX25lZyRiLCBtcjFfbW9kX3RhYmxlX25lZyRlc3RpbWF0ZSwgDQogICAgICAgICAgICAgICAgIG1ldGFfcmVncmVzc2lvbjFfbmVnJGJbLTFdLCBtZXRhX3JlZ3Jlc3Npb24xYl9uZWckYlstKDE6MildLCBtZXRhX3JlZ3Jlc3Npb24xY19uZWckYlstKDE6MyldKSwNCiAgYExvd2VyIENJIFswLjAyNV1gID0gYyhtZXRhX3JlZ3Jlc3Npb24xZF9uZWckY2kubGIsIG1yMV9tb2RfdGFibGVfbmVnJGxvd2VyQ0wsDQogICAgICAgICAgICAgICAgICAgICAgICAgbWV0YV9yZWdyZXNzaW9uMV9uZWckY2kubGJbLTFdLCBtZXRhX3JlZ3Jlc3Npb24xYl9uZWckY2kubGJbLSgxOjIpXSwgbWV0YV9yZWdyZXNzaW9uMWNfbmVnJGNpLmxiWy0oMTozKV0pLA0KICBgVXBwZXIgQ0kgIFswLjk3NV1gID0gYyhtZXRhX3JlZ3Jlc3Npb24xZF9uZWckY2kudWIsIG1yMV9tb2RfdGFibGVfbmVnJHVwcGVyQ0wsDQogICAgICAgICAgICAgICAgICAgICAgICAgbWV0YV9yZWdyZXNzaW9uMV9uZWckY2kudWJbLTFdLCBtZXRhX3JlZ3Jlc3Npb24xYl9uZWckY2kudWJbLSgxOjIpXSwgbWV0YV9yZWdyZXNzaW9uMWNfbmVnJGNpLnViWy0oMTozKV0pKSAtPiB0X2FubnVhbF9ldmVudF9uZWcNCiAjIGBSMmAgPSBjKHIyX21ldGFfcmVncmVzc2lvbjFbMV0sIHJlcChOQSwxMykpKSAtPiANCg0KdF9hbm51YWxfZXZlbnRfbmVnICU+JSBrYWJsZSgiaHRtbCIsIGRpZ2l0cyA9IDMpICU+JQ0KICBrYWJsZV9zdHlsaW5nKCJzdHJpcGVkIiwgcG9zaXRpb24gPSAibGVmdCIpDQogICAgICAgICAgICAgICANCmBgYA0KDQpgYGB7ciBtZXRhLXJlZ3Jlc3Npb24tbWV0aG9kLW5lZ30NCm1ldGFfcmVncmVzc2lvbjJfbmVnIDwtIHJtYS5tdih5aSA9IFpyLCBWID0gVkNWLCANCiAgICAgICAgICAgICAgICAgICAgICBtb2RzID0gfiBtZXRob2QsDQogICAgICAgICAgICAgICAgICAgICAgcmFuZG9tID0gbGlzdCh+MSB8IGVzX0lELCANCiAgICAgICAgICAgICAgICAgICAgICAgICAgICAgICAgICAgIH4xIHwgcGFwZXJfSUQsIA0KICAgICAgICAgICAgICAgICAgICAgICAgICAgICAgICAgICAgfjEgfCBjb2hvcnRfSUQsIA0KICAgICAgICAgICAgICAgICAgICAgICAgICAgICAgICAgICAgfjEgfCBzcGVjaWVzX0lELCANCiAgICAgICAgICAgICAgICAgICAgICAgICAgICAgICAgICAgIH4xIHwgcGh5bG9nZW55KSwNCiAgICAgICAgICAgICAgICAgICAgICBSID0gbGlzdChwaHlsb2dlbnkgPSB2YXJjb3IpLA0KICAgICAgICAgICAgICAgICAgICAgIGRhdGEgPSBkZikNCg0KIyByZW9yZGVyaW5nDQojIGRmJG1ldGhvZCA8LSBmYWN0b3IoZGYkbWV0aG9kLCBsZXZlbHMgPSBjKCJDb252ZW50aW9uYWwiLCAiR0xTIiwgIlNhdGVsbGl0ZSIpKQ0KDQptZXRhX3JlZ3Jlc3Npb24yYl9uZWcgPC0gcm1hLm12KHlpID0gWnIsIFYgPSBWQ1YsIA0KICAgICAgICAgICAgICAgICAgICAgICAgICAgIG1vZHMgPSB+IHJlbGV2ZWwobWV0aG9kLCByZWYgPSAiR0xTIiksDQogICAgICAgICAgICAgICAgICAgICAgICAgICAgcmFuZG9tID0gbGlzdCh+MSB8IGVzX0lELCANCiAgICAgICAgICAgICAgICAgICAgICAgICAgICAgICAgICAgICAgICAgIH4xIHwgcGFwZXJfSUQsIA0KICAgICAgICAgICAgICAgICAgICAgICAgICAgICAgICAgICAgICAgICAgfjEgfCBjb2hvcnRfSUQsIA0KICAgICAgICAgICAgICAgICAgICAgICAgICAgICAgICAgICAgICAgICAgfjEgfCBzcGVjaWVzX0lELCANCiAgICAgICAgICAgICAgICAgICAgICAgICAgICAgICAgICAgICAgICAgIH4xIHwgcGh5bG9nZW55KSwNCiAgICAgICAgICAgICAgICAgICAgICAgICAgICBSID0gbGlzdChwaHlsb2dlbnkgPSB2YXJjb3IpLA0KICAgICAgICAgICAgICAgICAgICAgICAgICAgIGRhdGEgPSBkZikNCg0KIyBPcmNoYXJkIHBsb3QgLSBuZWVkIG1ldGEtcmVncmVzc2lvbiB3aXRob3V0IGludGVyY2VwdA0KbWV0YV9yZWdyZXNzaW9uMmNfbmVnIDwtIHJtYS5tdih5aSA9IFpyLCBWID0gVkNWLCANCiAgICAgICAgICAgICAgICAgICAgICAgICAgICBtb2RzID0gfiBtZXRob2QgLTEsDQogICAgICAgICAgICAgICAgICAgICAgICAgICAgcmFuZG9tID0gbGlzdCh+MSB8IGVzX0lELCANCiAgICAgICAgICAgICAgICAgICAgICAgICAgICAgICAgICAgICAgICAgIH4xIHwgcGFwZXJfSUQsIA0KICAgICAgICAgICAgICAgICAgICAgICAgICAgICAgICAgICAgICAgICAgfjEgfCBjb2hvcnRfSUQsIA0KICAgICAgICAgICAgICAgICAgICAgICAgICAgICAgICAgICAgICAgICAgfjEgfCBzcGVjaWVzX0lELCANCiAgICAgICAgICAgICAgICAgICAgICAgICAgICAgICAgICAgICAgICAgIH4xIHwgcGh5bG9nZW55KSwNCiAgICAgICAgICAgICAgICAgICAgICAgICAgICBSID0gbGlzdChwaHlsb2dlbnkgPSB2YXJjb3IpLA0KICAgICAgICAgICAgICAgICAgICAgICAgICAgIGRhdGEgPSBkZikNCmBgYA0KDQojIyMjIFRhYmxlIFMxNA0KUmVncmVzc2lvbiBjb2VmZmljaWVudHMgKEVzdGltYXRlKSBhbmQgOTUlIGNvbmZpZGVuY2UgaW50ZXJ2YWxzIChDSXMpIGJvdGggaW4gWnIgYW5kIGJhY2stdHJhbnNmb3JtZWQgdG8gSUNDIGZyb20gdGhlIG1ldGEtcmVncmVzc2lvbiB3aXRoIGBtZXRob2RgIHdoZW4gbmVnYXRpdmUgcmVwZWF0YWJpbGl0eSB2YWx1ZXMgYXJlIGluY2x1ZGVkLiBOb3RlIHRoYXQgYG11YCBtZWFucyB0aGUgZ3JvdXAgbWVhbiB3aGlsZSBgYmV0YWAgcmVwcmVzZW50cyB0aGUgY29udHJhc3QgYmV0d2VlbiB0d28gZ3JvdXBzIGluIHRoZSBVbml0IGNvbHVtbi4gKlI8c3VwPjI8L3N1cD48c3ViPm1hcmdpbmFsPC9zdWI+KiA9IGByIHJvdW5kKG9yY2hhUmQ6OnIyX21sKG1ldGFfcmVncmVzc2lvbjJfbmVnKVtbMV1dKjEwMCwxKWAlLg0KDQpgYGB7ciBtZXRob2QtdGFibGUtbmVnfQ0KIyBnZXR0aW5nIG1hcmdpbmFsIFIyDQpyMl9tZXRhX3JlZ3Jlc3Npb24yX25lZyA8LSByMl9tbChtZXRhX3JlZ3Jlc3Npb24yX25lZykNCg0KIyBnZXR0aW5nIGVzdGltYXRlcw0KIyBpbmNsdWRpbmcgYmFjay10cmFuc2Zvcm1hdGlvbiB0byBJQ0MNCg0KbXIyX25lZyA8LSBtb2RfcmVzdWx0cyhtZXRhX3JlZ3Jlc3Npb24yY19uZWcsIG1vZD0ibWV0aG9kIikNCm1yMl9tb2RfdGFibGVfbmVnIDwtIG1yMl9uZWckbW9kX3RhYmxlDQoNCm1yMl9kYXRhX25lZyA8LSBtcjJfbmVnJGRhdGENCg0KIyBjYWxjdWxhdGUgayBmb3IgZWFjaCBtZXRob2Qgc2VwYXJhdGVseQ0KDQojZGYgJT4lIGdyb3VwX2J5KG1ldGhvZCkgJT4lIHN1bW1hcmlzZShtZWFuKGspKQ0KDQoNCm1yMl9kYXRhX25lZyA8LSBtcjJfZGF0YV9uZWcgJT4lIG11dGF0ZShrID0gY2FzZV93aGVuKG1vZGVyYXRvciA9PSAiR0xTIiB+IDIuMjAsIA0KICAgICAgICAgICAgICAgICAgICAgICAgICAgICAgICAgICAgICAgICAgICAgIG1vZGVyYXRvciA9PSAiQ29udmVudGlvbmFsIiB+IDMuMTMsDQogICAgICAgICAgICAgICAgICAgICAgICAgICAgICAgICAgICAgICAgICAgICAgbW9kZXJhdG9yID09ICJTYXRlbGxpdGUiIH4gMy4yOCkpDQoNCm1yMl9kYXRhX25lZyR5aV9JQ0MgPC0gWnJfdG9fSUNDKG1yMl9kYXRhX25lZyR5aSwgbXIyX2RhdGFfbmVnJGspDQoNCm1yMl9tb2RfdGFibGVfbmVnJGsgPC0gYygzLjEzLCAyLjIwLCAzLjI4KQ0KDQpmb3IoaSBpbiBuYW1lcyhtcjJfbW9kX3RhYmxlX25lZylbMjo2XSl7DQogIA0KICBtcjJfbW9kX3RhYmxlX25lZ1tpXSA8LSBacl90b19JQ0MobXIyX21vZF90YWJsZV9uZWdbaV0sIG1yMl9tb2RfdGFibGVfbmVnJGspDQogIA0KfQ0KDQojIGNyZWF0aW5nIGEgdGFibGUNCnRpYmJsZSgNCiAgYEZpeGVkIGVmZmVjdGAgPSBjKHJlcChhcy5jaGFyYWN0ZXIobXIyX21vZF90YWJsZV9uZWckbmFtZSksMiksIGNvbnRfZ2VuKG1yMl9tb2RfdGFibGVfbmVnJG5hbWUpKSwNCiAgYFVuaXRgID0gYyhyZXAoYygiWnIgKG11KSIsICJJQ0MgKG11KSIpLGVhY2ggPSAzKSwgcmVwKCJaciAoYmV0YSkiLCAzKSksDQogIGBFc3RpbWF0ZWAgPSBjKG1ldGFfcmVncmVzc2lvbjJjX25lZyRiLCBtcjJfbW9kX3RhYmxlX25lZyRlc3RpbWF0ZSwgDQogICAgICAgICAgICAgICAgIG1ldGFfcmVncmVzc2lvbjJfbmVnJGJbLTFdLCBtZXRhX3JlZ3Jlc3Npb24yYl9uZWckYlstKDE6MildKSwNCiAgYExvd2VyIENJIFswLjAyNV1gID0gYyhtZXRhX3JlZ3Jlc3Npb24yY19uZWckY2kubGIsIG1yMl9tb2RfdGFibGVfbmVnJGxvd2VyQ0wsDQogICAgICAgICAgICAgICAgICAgICAgICAgbWV0YV9yZWdyZXNzaW9uMl9uZWckY2kubGJbLTFdLCBtZXRhX3JlZ3Jlc3Npb24yYl9uZWckY2kubGJbLSgxOjIpXSksDQogIGBVcHBlciBDSSAgWzAuOTc1XWAgPSBjKG1ldGFfcmVncmVzc2lvbjJjX25lZyRjaS51YiwgbXIyX21vZF90YWJsZV9uZWckdXBwZXJDTCwNCiAgICAgICAgICAgICAgICAgICAgICAgICBtZXRhX3JlZ3Jlc3Npb24yX25lZyRjaS51YlstMV0sIG1ldGFfcmVncmVzc2lvbjJiX25lZyRjaS51YlstKDE6MildKSkgLT4gdF9tZXRob2RfbmVnDQoNCnRfbWV0aG9kX25lZyAlPiUga2FibGUoImh0bWwiLCBkaWdpdHMgPSAzKSAlPiUNCiAga2FibGVfc3R5bGluZygic3RyaXBlZCIsIHBvc2l0aW9uID0gImxlZnQiKSANCmBgYA0KDQpgYGB7ciBtZXRhLXJlZ3Jlc3Npb24tc2V4LW5lZ30NCiMgcmVvcmRlcmluZw0KI2RmJHNleCA8LSBmYWN0b3IoZGYkc2V4LCBsZXZlbHMgPSBjKCJGIiwgIk0iLCAiQiIpKQ0KDQptZXRhX3JlZ3Jlc3Npb240X25lZyA8LSBybWEubXYoeWkgPSBaciwgViA9IFZDViwgDQogICAgICAgICAgICAgICAgICAgICAgICAgICBtb2RzID0gfiBzZXgsDQogICAgICAgICAgICAgICAgICAgICAgICAgICByYW5kb20gPSBsaXN0KH4xIHwgZXNfSUQsIA0KICAgICAgICAgICAgICAgICAgICAgICAgICAgICAgICAgICAgICAgICB+MSB8IHBhcGVyX0lELCANCiAgICAgICAgICAgICAgICAgICAgICAgICAgICAgICAgICAgICAgICAgfjEgfCBjb2hvcnRfSUQsIA0KICAgICAgICAgICAgICAgICAgICAgICAgICAgICAgICAgICAgICAgICB+MSB8IHNwZWNpZXNfSUQsIA0KICAgICAgICAgICAgICAgICAgICAgICAgICAgICAgICAgICAgICAgICB+MSB8IHBoeWxvZ2VueSksDQogICAgICAgICAgICAgICAgICAgICAgICAgICBSID0gbGlzdChwaHlsb2dlbnkgPSB2YXJjb3IpLA0KICAgICAgICAgICAgICAgICAgICAgICAgICAgZGF0YSA9IGRmKQ0KDQptZXRhX3JlZ3Jlc3Npb240Yl9uZWcgPC0gcm1hLm12KHlpID0gWnIsIFYgPSBWQ1YsIA0KICAgICAgICAgICAgICAgICAgICAgICAgICAgIG1vZHMgPSB+IHJlbGV2ZWwoc2V4LCByZWYgPSAiTSIpLA0KICAgICAgICAgICAgICAgICAgICAgICAgICAgIHJhbmRvbSA9IGxpc3QofjEgfCBlc19JRCwgDQogICAgICAgICAgICAgICAgICAgICAgICAgICAgICAgICAgICAgICAgICB+MSB8IHBhcGVyX0lELCANCiAgICAgICAgICAgICAgICAgICAgICAgICAgICAgICAgICAgICAgICAgIH4xIHwgY29ob3J0X0lELCANCiAgICAgICAgICAgICAgICAgICAgICAgICAgICAgICAgICAgICAgICAgIH4xIHwgc3BlY2llc19JRCwgDQogICAgICAgICAgICAgICAgICAgICAgICAgICAgICAgICAgICAgICAgICB+MSB8IHBoeWxvZ2VueSksDQogICAgICAgICAgICAgICAgICAgICAgICAgICAgUiA9IGxpc3QocGh5bG9nZW55ID0gdmFyY29yKSwgDQogICAgICAgICAgICAgICAgICAgICAgICAgICAgZGF0YSA9IGRmKQ0KDQojIE9yY2hhcmQgcGxvdCAtIG5lZWQgbWV0YS1yZWdyZXNzaW9uIHdpdGhvdXQgaW50ZXJjZXB0DQptZXRhX3JlZ3Jlc3Npb240Y19uZWcgPC0gcm1hLm12KHlpID0gWnIsIFYgPSBWQ1YsIA0KICAgICAgICAgICAgICAgICAgICAgICAgICAgIG1vZHMgPSB+IHNleCAtMSwNCiAgICAgICAgICAgICAgICAgICAgICAgICAgICByYW5kb20gPSBsaXN0KH4xIHwgZXNfSUQsIA0KICAgICAgICAgICAgICAgICAgICAgICAgICAgICAgICAgICAgICAgICAgfjEgfCBwYXBlcl9JRCwgDQogICAgICAgICAgICAgICAgICAgICAgICAgICAgICAgICAgICAgICAgICB+MSB8IGNvaG9ydF9JRCwgDQogICAgICAgICAgICAgICAgICAgICAgICAgICAgICAgICAgICAgICAgICB+MSB8IHNwZWNpZXNfSUQsIA0KICAgICAgICAgICAgICAgICAgICAgICAgICAgICAgICAgICAgICAgICAgfjEgfCBwaHlsb2dlbnkpLA0KICAgICAgICAgICAgICAgICAgICAgICAgICAgIFIgPSBsaXN0KHBoeWxvZ2VueSA9IHZhcmNvciksDQogICAgICAgICAgICAgICAgICAgICAgICAgICAgZGF0YSA9IGRmKQ0KYGBgDQoNCiMjIyMgVGFibGUgUzE1DQpSZWdyZXNzaW9uIGNvZWZmaWNpZW50cyAoRXN0aW1hdGUpIGFuZCA5NSUgY29uZmlkZW5jZSBpbnRlcnZhbHMgKENJcykgYm90aCBpbiBaciBhbmQgYmFjay10cmFuc2Zvcm1lZCB0byBJQ0MgZnJvbSB0aGUgbWV0YS1yZWdyZXNzaW9uIHdpdGggYHNleGAgd2hlbiBuZWdhdGl2ZSByZXBlYXRhYmlsaXR5IHZhbHVlcyBhcmUgaW5jbHVkZWQuIE5vdGUgdGhhdCBgbXVgIG1lYW5zIHRoZSBncm91cCBtZWFuIHdoaWxlIGBiZXRhYCByZXByZXNlbnRzIHRoZSBjb250cmFzdCBiZXR3ZWVuIHR3byBncm91cHMgaW4gdGhlIFVuaXQgY29sdW1uLiAqUjxzdXA+Mjwvc3VwPjxzdWI+bWFyZ2luYWw8L3N1Yj4qID0gYHIgcm91bmQob3JjaGFSZDo6cjJfbWwobWV0YV9yZWdyZXNzaW9uNF9uZWcpW1sxXV0qMTAwLDEpYCUuIEIgPSBib3RoIHNleGVzLCBNID0gbWFsZSwgYW5kIEYgPSBmZW1hbGUuDQoNCmBgYHtyIHNleC10YWJsZS1uZWd9DQoNCiMgZ2V0dGluZyBtYXJnaW5hbCBSMg0KcjJfbWV0YV9yZWdyZXNzaW9uNF9uZWcgPC0gcjJfbWwobWV0YV9yZWdyZXNzaW9uNF9uZWcpDQoNCiMgZ2V0dGluZyBlc3RpbWF0ZXMNCiMgaW5jbHVkaW5nIGJhY2stdHJhbnNmb3JtYXRpb24gdG8gSUNDDQoNCm1yNF9uZWcgPC0gbW9kX3Jlc3VsdHMobWV0YV9yZWdyZXNzaW9uNGNfbmVnLCBtb2Q9InNleCIpDQptcjRfbW9kX3RhYmxlX25lZyA8LSBtcjRfbmVnJG1vZF90YWJsZQ0KDQptcjRfZGF0YV9uZWcgPC0gbXI0X25lZyRkYXRhDQoNCiMgY2FsY3VsYXRlIGsgZm9yIGVhY2ggbWV0aG9kIHNlcGFyYXRlbHkNCg0KI2RmICU+JSBncm91cF9ieShzZXgpICU+JSBzdW1tYXJpc2UobWVhbihrKSkNCg0KDQptcjRfZGF0YV9uZWcgPC0gbXI0X2RhdGFfbmVnICU+JSBtdXRhdGUoayA9IGNhc2Vfd2hlbihtb2RlcmF0b3IgPT0gIkYiIH4gMi4zNywgDQogICAgICAgICAgICAgICAgICAgICAgICAgICAgICAgICAgICAgICAgICAgICAgbW9kZXJhdG9yID09ICJNIiB+IDIuMzgsDQogICAgICAgICAgICAgICAgICAgICAgICAgICAgICAgICAgICAgICAgICAgICAgbW9kZXJhdG9yID09ICJCIiB+IDIuODIpKQ0KDQptcjRfZGF0YV9uZWckeWlfSUNDIDwtIFpyX3RvX0lDQyhtcjRfZGF0YV9uZWckeWksIG1yNF9kYXRhX25lZyRrKQ0KDQptcjRfbW9kX3RhYmxlX25lZyRrIDwtIGMoMi4zNywgMi4zOCwgMi44MikNCg0KZm9yKGkgaW4gbmFtZXMobXI0X21vZF90YWJsZV9uZWcpWzI6Nl0pew0KICANCiAgbXI0X21vZF90YWJsZV9uZWdbaV0gPC0gWnJfdG9fSUNDKG1yNF9tb2RfdGFibGVfbmVnW2ldLCBtcjRfbW9kX3RhYmxlX25lZyRrKQ0KICANCn0NCg0KIyBjcmVhdGluZyBhIHRhYmxlDQp0aWJibGUoDQogIGBGaXhlZCBlZmZlY3RgID0gYyhyZXAoYXMuY2hhcmFjdGVyKG1yNF9tb2RfdGFibGVfbmVnJG5hbWUpLDIpLCBjb250X2dlbihtcjRfbW9kX3RhYmxlX25lZyRuYW1lKSksDQogIGBVbml0YCA9IGMocmVwKGMoIlpyIChtdSkiLCAiSUNDIChtdSkiKSxlYWNoID0gMyksIHJlcCgiWnIgKGJldGEpIiwgMykpLA0KICBgRXN0aW1hdGVgID0gYyhtZXRhX3JlZ3Jlc3Npb240Y19uZWckYiwgbXI0X21vZF90YWJsZV9uZWckZXN0aW1hdGUsIA0KICAgICAgICAgICAgICAgICBtZXRhX3JlZ3Jlc3Npb240X25lZyRiWy0xXSwgbWV0YV9yZWdyZXNzaW9uNGJfbmVnJGJbLSgxOjIpXSksDQogIGBMb3dlciBDSSBbMC4wMjVdYCA9IGMobWV0YV9yZWdyZXNzaW9uNGNfbmVnJGNpLmxiLCBtcjRfbW9kX3RhYmxlX25lZyRsb3dlckNMLA0KICAgICAgICAgICAgICAgICAgICAgICAgIG1ldGFfcmVncmVzc2lvbjRfbmVnJGNpLmxiWy0xXSwgbWV0YV9yZWdyZXNzaW9uNGJfbmVnJGNpLmxiWy0oMToyKV0pLA0KICBgVXBwZXIgQ0kgIFswLjk3NV1gID0gYyhtZXRhX3JlZ3Jlc3Npb240Y19uZWckY2kudWIsIG1yNF9tb2RfdGFibGVfbmVnJHVwcGVyQ0wsDQogICAgICAgICAgICAgICAgICAgICAgICAgbWV0YV9yZWdyZXNzaW9uNF9uZWckY2kudWJbLTFdLCBtZXRhX3JlZ3Jlc3Npb240Yl9uZWckY2kudWJbLSgxOjIpXSkpIC0+IHRfc2V4X25lZw0KDQp0X3NleF9uZWcgJT4lIGthYmxlKCJodG1sIiwgZGlnaXRzID0gMykgJT4lDQogIGthYmxlX3N0eWxpbmcoInN0cmlwZWQiLCBwb3NpdGlvbiA9ICJsZWZ0IikNCg0KYGBgDQoNCmBgYHtyIG1ldGEtcmVncmVzc2lvbi10YXhhLW5lZ30NCm1ldGFfcmVncmVzc2lvbjNfbmVnIDwtIHJtYS5tdih5aSA9IFpyLCBWID0gVkNWLCANCiAgICAgICAgICAgICAgICAgICAgICAgICAgIG1vZHMgPSB+IHRheGEsDQogICAgICAgICAgICAgICAgICAgICAgICAgICByYW5kb20gPSBsaXN0KH4xIHwgZXNfSUQsIA0KICAgICAgICAgICAgICAgICAgICAgICAgICAgICAgICAgICAgICAgICB+MSB8IHBhcGVyX0lELCANCiAgICAgICAgICAgICAgICAgICAgICAgICAgICAgICAgICAgICAgICAgfjEgfCBjb2hvcnRfSUQsIA0KICAgICAgICAgICAgICAgICAgICAgICAgICAgICAgICAgICAgICAgICB+MSB8IHNwZWNpZXNfSUQsIA0KICAgICAgICAgICAgICAgICAgICAgICAgICAgICAgICAgICAgICAgICB+MSB8IHBoeWxvZ2VueSksDQogICAgICAgICAgICAgICAgICAgICAgICAgICBSID0gbGlzdChwaHlsb2dlbnkgPSB2YXJjb3IpLCANCiAgICAgICAgICAgICAgICAgICAgICAgICAgIGRhdGEgPSBkZikNCg0KIyByZW9yZGVyaW5nDQojZGYkdGF4YSA8LSBmYWN0b3IoZGYkdGF4YSwgbGV2ZWxzID0gYygiV2F0ZXJiaXJkIiwgIlNlYWJpcmQiLCAiTGFuZGJpcmQiKSkNCg0KbWV0YV9yZWdyZXNzaW9uM2JfbmVnIDwtIHJtYS5tdih5aSA9IFpyLCBWID0gVkNWLCANCiAgICAgICAgICAgICAgICAgICAgICAgICAgIG1vZHMgPSB+IHJlbGV2ZWwodGF4YSwgcmVmID0gIldhdGVyYmlyZCIpLA0KICAgICAgICAgICAgICAgICAgICAgICAgICAgcmFuZG9tID0gbGlzdCh+MSB8IGVzX0lELCANCiAgICAgICAgICAgICAgICAgICAgICAgICAgICAgICAgICAgICAgICAgfjEgfCBwYXBlcl9JRCwgDQogICAgICAgICAgICAgICAgICAgICAgICAgICAgICAgICAgICAgICAgIH4xIHwgY29ob3J0X0lELCANCiAgICAgICAgICAgICAgICAgICAgICAgICAgICAgICAgICAgICAgICAgfjEgfCBzcGVjaWVzX0lELCANCiAgICAgICAgICAgICAgICAgICAgICAgICAgICAgICAgICAgICAgICAgfjEgfCBwaHlsb2dlbnkpLA0KICAgICAgICAgICAgICAgICAgICAgICAgICAgUiA9IGxpc3QocGh5bG9nZW55ID0gdmFyY29yKSwgDQogICAgICAgICAgICAgICAgICAgICAgICAgICBkYXRhID0gZGYpDQoNCiMgT3JjaGFyZCBwbG90IC0gbmVlZCBtZXRhLXJlZ3Jlc3Npb24gd2l0aG91dCBpbnRlcmNlcHQNCm1ldGFfcmVncmVzc2lvbjNjX25lZyA8LSBybWEubXYoeWkgPSBaciwgViA9IFZDViwgDQogICAgICAgICAgICAgICAgICAgICAgICAgICAgbW9kcyA9IH4gdGF4YSAtMSwNCiAgICAgICAgICAgICAgICAgICAgICAgICAgICByYW5kb20gPSBsaXN0KH4xIHwgZXNfSUQsIA0KICAgICAgICAgICAgICAgICAgICAgICAgICAgICAgICAgICAgICAgICAgfjEgfCBwYXBlcl9JRCwgDQogICAgICAgICAgICAgICAgICAgICAgICAgICAgICAgICAgICAgICAgICB+MSB8IGNvaG9ydF9JRCwgDQogICAgICAgICAgICAgICAgICAgICAgICAgICAgICAgICAgICAgICAgICB+MSB8IHNwZWNpZXNfSUQsIA0KICAgICAgICAgICAgICAgICAgICAgICAgICAgICAgICAgICAgICAgICAgfjEgfCBwaHlsb2dlbnkpLA0KICAgICAgICAgICAgICAgICAgICAgICAgICAgIFIgPSBsaXN0KHBoeWxvZ2VueSA9IHZhcmNvciksDQogICAgICAgICAgICAgICAgICAgICAgICAgICAgZGF0YSA9IGRmKQ0KYGBgDQoNCiMjIyMgVGFibGUgUzE2DQpSZWdyZXNzaW9uIGNvZWZmaWNpZW50cyAoRXN0aW1hdGUpIGFuZCA5NSUgY29uZmlkZW5jZSBpbnRlcnZhbHMgKENJcykgYm90aCBpbiBaciBhbmQgYmFjay10cmFuc2Zvcm1lZCB0byBJQ0MgZnJvbSB0aGUgbWV0YS1yZWdyZXNzaW9uIHdpdGggYHRheGFgIHdoZW4gbmVnYXRpdmUgcmVwZWF0YWJpbGl0eSB2YWx1ZXMgYXJlIGluY2x1ZGVkLiBOb3RlIHRoYXQgYG11YCBtZWFucyB0aGUgZ3JvdXAgbWVhbiB3aGlsZSBgYmV0YWAgcmVwcmVzZW50cyB0aGUgY29udHJhc3QgYmV0d2VlbiB0d28gZ3JvdXBzIGluIHRoZSBVbml0IGNvbHVtbi4gKlI8c3VwPjI8L3N1cD48c3ViPm1hcmdpbmFsPC9zdWI+KiA9IGByIHJvdW5kKG9yY2hhUmQ6OnIyX21sKG1ldGFfcmVncmVzc2lvbjNfbmVnKVtbMV1dKjEwMCwxKWAlLg0KDQpgYGB7ciB0YXhhLXRhYmxlLW5lZ30NCg0KIyBnZXR0aW5nIG1hcmdpbmFsIFIyDQpyMl9tZXRhX3JlZ3Jlc3Npb24zX25lZyA8LSByMl9tbChtZXRhX3JlZ3Jlc3Npb24zX25lZykNCg0KIyBnZXR0aW5nIGVzdGltYXRlcw0KIyBpbmNsdWRpbmcgYmFjay10cmFuc2Zvcm1hdGlvbiB0byBJQ0MNCg0KbXIzX25lZyA8LSBtb2RfcmVzdWx0cyhtZXRhX3JlZ3Jlc3Npb24zY19uZWcsIG1vZD0idGF4YSIpDQptcjNfbW9kX3RhYmxlX25lZyA8LSBtcjNfbmVnJG1vZF90YWJsZQ0KDQptcjNfZGF0YV9uZWcgPC0gbXIzX25lZyRkYXRhDQoNCiMgY2FsY3VsYXRlIGsgZm9yIGVhY2ggbWV0aG9kIHNlcGFyYXRlbHkNCg0KI2RmICU+JSBncm91cF9ieSh0YXhhKSAlPiUgc3VtbWFyaXNlKG1lYW4oaykpDQoNCm1yM19kYXRhX25lZyA8LSBtcjNfZGF0YV9uZWcgJT4lIG11dGF0ZShrID0gY2FzZV93aGVuKG1vZGVyYXRvciA9PSAiTGFuZGJpcmQiIH4gMi42OCwgDQogICAgICAgICAgICAgICAgICAgICAgICAgICAgICAgICAgICAgICAgICAgICAgbW9kZXJhdG9yID09ICJTZWFiaXJkIiB+IDIuMzgsDQogICAgICAgICAgICAgICAgICAgICAgICAgICAgICAgICAgICAgICAgICAgICAgbW9kZXJhdG9yID09ICJXYXRlcmJpcmQiIH4gMy4wOCkpDQoNCm1yM19kYXRhX25lZyR5aV9JQ0MgPC0gWnJfdG9fSUNDKG1yM19kYXRhX25lZyR5aSwgbXIzX2RhdGFfbmVnJGspDQoNCm1yM19tb2RfdGFibGVfbmVnJGsgPC0gYygyLjY4LCAyLjM4LCAzLjA4KQ0KDQpmb3IoaSBpbiBuYW1lcyhtcjNfbW9kX3RhYmxlX25lZylbMjo2XSl7DQogIA0KICBtcjNfbW9kX3RhYmxlX25lZ1tpXSA8LSBacl90b19JQ0MobXIzX21vZF90YWJsZV9uZWdbaV0sIG1yM19tb2RfdGFibGVfbmVnJGspDQogIA0KfQ0KDQojIGNyZWF0aW5nIGEgdGFibGUNCnRpYmJsZSgNCiAgYEZpeGVkIGVmZmVjdGAgPSBjKHJlcChhcy5jaGFyYWN0ZXIobXIzX21vZF90YWJsZV9uZWckbmFtZSksMiksIGNvbnRfZ2VuKG1yM19tb2RfdGFibGVfbmVnJG5hbWUpKSwNCiAgYFVuaXRgID0gYyhyZXAoYygiWnIgKG11KSIsICJJQ0MgKG11KSIpLGVhY2ggPSAzKSwgcmVwKCJaciAoYmV0YSkiLCAzKSksDQogIGBFc3RpbWF0ZWAgPSBjKG1ldGFfcmVncmVzc2lvbjNjX25lZyRiLCBtcjNfbW9kX3RhYmxlX25lZyRlc3RpbWF0ZSwgDQogICAgICAgICAgICAgICAgIG1ldGFfcmVncmVzc2lvbjNfbmVnJGJbLTFdLCBtZXRhX3JlZ3Jlc3Npb24zYl9uZWckYlstKDE6MildKSwNCiAgYExvd2VyIENJIFswLjAyNV1gID0gYyhtZXRhX3JlZ3Jlc3Npb24zY19uZWckY2kubGIsIG1yM19tb2RfdGFibGVfbmVnJGxvd2VyQ0wsDQogICAgICAgICAgICAgICAgICAgICAgICAgbWV0YV9yZWdyZXNzaW9uM19uZWckY2kubGJbLTFdLCBtZXRhX3JlZ3Jlc3Npb24zYl9uZWckY2kubGJbLSgxOjIpXSksDQogIGBVcHBlciBDSSAgWzAuOTc1XWAgPSBjKG1ldGFfcmVncmVzc2lvbjNjX25lZyRjaS51YiwgbXIzX21vZF90YWJsZV9uZWckdXBwZXJDTCwNCiAgICAgICAgICAgICAgICAgICAgICAgICBtZXRhX3JlZ3Jlc3Npb24zX25lZyRjaS51YlstMV0sIG1ldGFfcmVncmVzc2lvbjNiX25lZyRjaS51YlstKDE6MildKSkgLT4gdF9tZXRob2RfbmVnDQoNCnRfbWV0aG9kX25lZyAlPiUga2FibGUoImh0bWwiLCBkaWdpdHMgPSAzKSAlPiUNCiAga2FibGVfc3R5bGluZygic3RyaXBlZCIsIHBvc2l0aW9uID0gImxlZnQiKSANCg0KYGBgDQoNCmBgYHtyIG1ldGEtcmVncmVzc2lvbi1rLW5lZ30NCg0KbWV0YV9yZWdyZXNzaW9uNV9uZWcgPC0gcm1hLm12KHlpID0gWnIsIFYgPSBWQ1YsIA0KICAgICAgICAgICAgICAgICAgICAgICAgICAgIG1vZHMgPSB+IGssDQogICAgICAgICAgICAgICAgICAgICAgICAgICAgcmFuZG9tID0gbGlzdCh+MSB8IGVzX0lELCANCiAgICAgICAgICAgICAgICAgICAgICAgICAgICAgICAgICAgICAgICAgIH4xIHwgcGFwZXJfSUQsIA0KICAgICAgICAgICAgICAgICAgICAgICAgICAgICAgICAgICAgICAgICAgfjEgfCBjb2hvcnRfSUQsIA0KICAgICAgICAgICAgICAgICAgICAgICAgICAgICAgICAgICAgICAgICAgfjEgfCBzcGVjaWVzX0lELCANCiAgICAgICAgICAgICAgICAgICAgICAgICAgICAgICAgICAgICAgICAgIH4xIHwgcGh5bG9nZW55KSwNCiAgICAgICAgICAgICAgICAgICAgICAgICAgICBSID0gbGlzdChwaHlsb2dlbnkgPSB2YXJjb3IpLA0KICAgICAgICAgICAgICAgICAgICAgICAgICAgIGRhdGEgPSBkZikNCmBgYA0KDQojIyMjIFRhYmxlIFMxNw0KUmVncmVzc2lvbiBjb2VmZmljaWVudHMgKEVzdGltYXRlKSBhbmQgOTUlIGNvbmZpZGVuY2UgaW50ZXJ2YWxzIChDSXMpIGluIFpyIGZyb20gdGhlIG1ldGEtcmVncmVzc2lvbiB3aXRoIGBrYCAobnVtYmVyIG9mIG9ic2VydmF0aW9ucyBwZXIgaW5kaXZpZHVhbCkgd2hlbiBuZWdhdGl2ZSByZXBlYXRhYmlsaXR5IHZhbHVlcyBhcmUgaW5jbHVkZWQuICpSPHN1cD4yPC9zdXA+PHN1Yj5tYXJnaW5hbDwvc3ViPiogPSBgciByb3VuZChvcmNoYVJkOjpyMl9tbChtZXRhX3JlZ3Jlc3Npb241X25lZylbWzFdXSoxMDAsMSlgJS4NCg0KYGBge3Igay10YWJsZS1uZWd9DQojIGdldHRpbmcgbWFyZ2luYWwgUjINCnIyX21ldGFfcmVncmVzc2lvbjVfbmVnIDwtIHIyX21sKG1ldGFfcmVncmVzc2lvbjVfbmVnKQ0KDQojIGNyZWF0aW5nIGEgdGFibGUNCnRpYmJsZSgNCiAgYEZpeGVkIGVmZmVjdGAgPSBjKCJJbnRlcmNlcHQiLCAiayIpLA0KICBgRXN0aW1hdGVgID0gYyhtZXRhX3JlZ3Jlc3Npb241X25lZyRiKSwNCiAgYExvd2VyIENJIFswLjAyNV1gID0gYyhtZXRhX3JlZ3Jlc3Npb241X25lZyRjaS5sYiksDQogIGBVcHBlciBDSSAgWzAuOTc1XWAgPSBjKG1ldGFfcmVncmVzc2lvbjVfbmVnJGNpLnViKSkgLT4gdF9rX25lZw0KDQp0X2tfbmVnICU+JSBrYWJsZSgiaHRtbCIsIGRpZ2l0cyA9IDMpICU+JQ0KICBrYWJsZV9zdHlsaW5nKCJzdHJpcGVkIiwgcG9zaXRpb24gPSAibGVmdCIpIA0KDQpgYGANCg0KYGBge3IgbWV0YS1yZWdyZXNzaW9uLW91dGxpZXItay1uZWd9DQpWQ1YyIDwtIGltcHV0ZV9jb3ZhcmlhbmNlX21hdHJpeCh2aSA9IGRmMiRWWnIsIGNsdXN0ZXIgPSBkZjIkY29ob3J0X0lELCByID0gMC41KQ0KDQptZXRhX3JlZ3Jlc3Npb242X25lZyA8LSBybWEubXYoeWkgPSBaciwgViA9IFZDVjIsIA0KICAgICAgICAgICAgICAgICAgICAgICAgICAgbW9kcyA9IH4gaywNCiAgICAgICAgICAgICAgICAgICAgICAgICAgIHJhbmRvbSA9IGxpc3QofjEgfCBlc19JRCwgDQogICAgICAgICAgICAgICAgICAgICAgICAgICAgICAgICAgICAgICAgIH4xIHwgcGFwZXJfSUQsIA0KICAgICAgICAgICAgICAgICAgICAgICAgICAgICAgICAgICAgICAgICB+MSB8IGNvaG9ydF9JRCwgDQogICAgICAgICAgICAgICAgICAgICAgICAgICAgICAgICAgICAgICAgIH4xIHwgc3BlY2llc19JRCwgDQogICAgICAgICAgICAgICAgICAgICAgICAgICAgICAgICAgICAgICAgIH4xIHwgcGh5bG9nZW55KSwNCiAgICAgICAgICAgICAgICAgICAgICAgICAgIFIgPSBsaXN0KHBoeWxvZ2VueSA9IHZhcmNvciksICMgYWRkZWQgaW4gcGh5bG9nbmV5DQogICAgICAgICAgICAgICAgICAgICAgICAgICBkYXRhID0gZGYyKQ0KYGBgDQoNCiMjIyBNb2RlbCBzZWxlY3Rpb24gd2l0aCBuZWdhdGl2ZSByZXBlYXRhYmlsaXR5IGVzdGltYXRlcyBpbmNsdWRlZA0KDQpIZXJlIHdlIHVzZWQgdGhlIGBNdU1pbmAgcGFja2FnZSB0byBnZW5lcmF0ZSBhbGwgcG9zc2libGUgbW9kZXJhdG9yIGNvbWJpbmF0aW9ucyAodXNpbmcgYWxsIGZpdmUgdmFyaWFibGVzOiBgYW5udWFsX2V2ZW50YCwgYG1ldGhvZGAsIGBzZXhgLCBgdGF4YWAgJiBga2AgKG51bWJlciBvZiBvYnNlcnZhdGlvbnMgcGVyIGluZGl2aWR1YWxzKSksIGRldGVybWluZSB0aGUgaW1wb3J0YW5jZSBvZiB0aGUgbW9kZXJhdG9ycywgYW5kIGdlbmVyYXRlIG1vZGVsLWF2ZXJhZ2VkIGVzdGltYXRlcyB3aGVuIHRoZSBuZWdhdGl2ZSByZXBlYXRhYmlsaXR5IGVzdGltYXRlcyBhcmUgaW5jbHVkZWQuDQoNCmBgYHtyIG1vZGVsLXNlbGVjdGlvbi1uZWd9DQpldmFsKG1ldGFmb3I6OjouTXVNSW4pDQoNCmZ1bGxfbW9kZWxfTXVNSW5fbmVnIDwtIHJtYS5tdih5aSA9IFpyLCBWID0gVkNWLCANCiAgICAgICAgICAgICAgICAgICAgICAgICAgbW9kcyA9IH4gbWV0aG9kICsgDQogICAgICAgICAgICAgICAgICAgICAgICAgICAgdGF4YSArIA0KICAgICAgICAgICAgICAgICAgICAgICAgICAgIHNleCArIA0KICAgICAgICAgICAgICAgICAgICAgICAgICAgIGFubnVhbF9ldmVudCArDQogICAgICAgICAgICAgICAgICAgICAgICAgICAgaywgDQogICAgICAgICAgICAgICAgICAgICAgICAgIHJhbmRvbSA9IGxpc3QofjEgfCBlc19JRCwgDQogICAgICAgICAgICAgICAgICAgICAgICAgICAgICAgICAgICAgICAgfjEgfCBwYXBlcl9JRCwgDQogICAgICAgICAgICAgICAgICAgICAgICAgICAgICAgICAgICAgICAgfjEgfCBjb2hvcnRfSUQsIA0KICAgICAgICAgICAgICAgICAgICAgICAgICAgICAgICAgICAgICAgIH4xIHwgc3BlY2llc19JRCwgDQogICAgICAgICAgICAgICAgICAgICAgICAgICAgICAgICAgICAgICAgfjEgfCBwaHlsb2dlbnkpLA0KICAgICAgICAgICAgICAgICAgICAgICAgICBSID0gbGlzdChwaHlsb2dlbnkgPSB2YXJjb3IpLCANCiAgICAgICAgICAgICAgICAgICAgICAgICAgbWV0aG9kID0gIk1MIiwgIyBtYXhpbXVtIGxpa2VsaWhvb2QgZm9yIG1vZGVsIHNlbGVjdGlvbg0KICAgICAgICAgICAgICAgICAgICAgICAgICBkYXRhID0gZGYpDQoNCiMgdmlmLnJtYShmdWxsX21vZGVsX011TUluKSAgIyBObyBtYWpvciBwcm9ibGVtcyBvZiBjb2xsaW5lYXJpdHkgKFZJRiA8NCkNCg0KY2FuZGlkYXRlX21vZGVsc19uZWcgPC1kcmVkZ2UoZnVsbF9tb2RlbF9NdU1Jbl9uZWcpICMgR2VuZXJhdGUgYWxsIHBvc3NpYmxlIGNvbWJpbmF0aW9ucyBvZiBtb2RlcmF0b3JzDQoNCmNhbmRpZGF0ZXNfYWljMl9uZWcgPC0gc3Vic2V0KGNhbmRpZGF0ZV9tb2RlbHNfbmVnLCBkZWx0YTw9MikgIyBEaXNwbGF5IGFsbCBtb2RlbHMgd2l0aGluIDIgdmFsdWVzIG9mIEFJQ2MNCg0KaW1wb3J0YW5jZV9uZWcgPC0gc3cobW9kZWwuYXZnKGNhbmRpZGF0ZV9tb2RlbHNfbmVnLCBzdWJzZXQ9ZGVsdGE8PTIpKSMgcmVsYXRpdmUgaW1wb3J0YW5jZSAoc3VtIG9mIHdlaWdodHMpIG9mIHRoZSBtb2RlcmF0b3JzDQoNCm1vZC5hdmdfbmVnIDwtIHN1bW1hcnkobW9kZWwuYXZnKGNhbmRpZGF0ZV9tb2RlbHNfbmVnLCBzdWJzZXQ9ZGVsdGE8PTIpKSAjIEdlbmVyYXRlIG1vZGVsLWF2ZXJhZ2VkIGVzdGltYXRlcyANCg0KY29uZmlkZW5jZV9uZWcgPC0gY29uZmludChtb2QuYXZnX25lZywgZnVsbD1UUlVFKSAjIEdlbmVyYXRlIGNvbmZpZGVuY2UgaW50ZXJ2YWxzIGZvciB0aGUgZXN0aW1hdGVzIGF2ZXJhZ2VkIHVzaW5nIGZ1bGwtYXZlcmFnZXMgcHJvY2VkdXJlcw0KDQpgYGANCg0KIyMjIyBUYWJsZSBTMTgNClRoZSB0b3Agc2l4IG1vZGVscyAod2hlbiBuZWdhdGl2ZSByZXBlYXRhYmlsaXR5IHZhbHVlcyBhcmUgaW5jbHVkZWQpIHdpdGhpbiB0aGUgJFxEZWx0YSRBSUMgZGlmZmVyZW5jZSBvZiAyLCBhbmQgd2hpY2ggZml2ZSB2YXJpYWJsZXM6IGBhbm51YWxfZXZlbnRgLCBgbWV0aG9kYCwgYHRheGFgLCBgc2V4YCwgJiBga2Agd2VyZSBpbmNsdWRlZCAoaW5kaWNhdGVkIGJ5ICQrJCk7IG1vZGVsIHdlaWdodHMgYW5kIHRoZSBzdW0gb2Ygd2VpZ2h0cyBmb3IgZWFjaCBvZiB0aGUgdmFyaWFibGVzIGFyZSBpbmNsdWRlZC4gDQoNCmBgYHtyIG1vZGVsLXNlbGVjdGlvbi10YWJsZS1uZWd9DQojIGNyZWF0aW5nIGEgdGFibGUNCnRpYmJsZSgNCiAgYE1vZGVsICh2YXJpYWJsZSB3ZWlnaHQpYCA9IGMoIk1vZGVsMSIsICJNb2RlbDIiLCAiTW9kZWwzIiwgIk1vZGVsNCIsICJNb2RlbDUiLCAiTW9kZWw2IiwgIihTdW0gb2Ygd2VpZ2h0cykiKSwNCiAgYW5udWFsX2V2ZW50ID0gYyhpZl9lbHNlKGNhbmRpZGF0ZXNfYWljMl9uZWckYW5udWFsX2V2ZW50ID09ICIrIiwgIiQrJCIsICJOQSIpLHJvdW5kKGltcG9ydGFuY2VfbmVnWzFdLDMpICksDQogIHNleCA9IGMoaWZfZWxzZShjYW5kaWRhdGVzX2FpYzJfbmVnJHNleD09ICIrIiwgIiQrJCIsICJOQSIpLHJvdW5kKGltcG9ydGFuY2VfbmVnWzJdLCAzKSkgLA0KICB0YXhhID0gIGMoaWZfZWxzZShjYW5kaWRhdGVzX2FpYzJfbmVnJHRheGE9PSAiKyIsICIkKyQiLCAiTkEiKSxyb3VuZChpbXBvcnRhbmNlX25lZ1szXSwgMykpLA0KICBgbWV0aG9kYCA9IGMoaWZfZWxzZShjYW5kaWRhdGVzX2FpYzJfbmVnJG1ldGhvZD09ICIrIiwgIiQrJCIsICJOQSIpLHJvdW5kKGltcG9ydGFuY2VfbmVnWzRdLCAzKSksDQogIGsgPSBjKGlmX2Vsc2UoY2FuZGlkYXRlc19haWMyX25lZyRrIDw9IDAsICIkKyQiLCAiTkEiKSxyb3VuZChpbXBvcnRhbmNlX25lZ1s1XSwgMykpLA0KICBkZWx0YV9BSUNjID0gYyhjYW5kaWRhdGVzX2FpYzJfbmVnJGRlbHRhLCBOQSksDQogIFdlaWdodCA9IGMoY2FuZGlkYXRlc19haWMyX25lZyR3ZWlnaHQsIE5BKSkgJT4lIA0KICBrYWJsZSgiaHRtbCIsIGRpZ2l0cyA9IDMpICU+JQ0KICBrYWJsZV9zdHlsaW5nKCJzdHJpcGVkIiwgcG9zaXRpb24gPSAibGVmdCIpDQpgYGANCg0KIyMjIyBNb2RlbCBhdmVyYWdpbmcNCg0KIyMjIyBUYWJsZSBTMTkNClRoZSBhdmVyYWdlIGVzdGltYXRlcyBmb3IgcmVncmVzc2lvbiBjb2VmZmljaWVudHMgKEVzdGltYXRlKSBhbmQgOTUlIGNvbmZpZGVuY2UgaW50ZXJ2YWxzIChDSXMpIGZyb20gdGhlIG1vZGVsIGF2ZXJhZ2luZyBwcm9jZWR1cmUgd2hlbiBuZWdhdGl2ZSByZXBlYXRhYmlsaXR5IHZhbHVlcyBhcmUgaW5jbHVkZWQgdXNpbmcgZnVsbC1hdmVyYWdlcyAoYXNzdW1pbmcgemVybyB2YWx1ZXMgZm9yIG1vZGVyYXRvcnMgd2hlbiB0aGV5IGRvIG5vdCBvY2N1cikuDQpgYGB7ciBtb2RlbC1hdmVyYWdpbmctbmVnfQ0KIyBjcmVhdGluZyBhIHRhYmxlDQp0aWJibGUoDQogIGBGaXhlZCBlZmZlY3RgID0gYygiSW50ZXJjZXB0IiwNCiAgICAgICAgICAgICAgICAgICAgICJEZXBhcnRfYnJlZWQiLCAiTm9uYnJlZWRfYXJyaXZhbCIsICJOb25icmVlZF9kZXBhcnQiLCANCiAgICAgICAgICAgICAgICAgICAgICJTZWFiaXJkIiwgIldhdGVyYmlyZCIsICJHTFMiLCAiU2F0ZWxsaXRlIiwNCiAgICAgICAgICAgICAgICAgICAgICJrIiwiRmVtYWxlIiwiTWFsZSIpLA0KICBFc3RpbWF0ZSA9IG1vZC5hdmdfbmVnJGNvZWZtYXQuZnVsbFssMV0sDQogIGBMb3dlciBDSSBbMC4wMjVdYCA9IGNvbmZpZGVuY2VfbmVnWywxXSwNCiAgYFVwcGVyIENJICBbMC45NzVdYCA9ICBjb25maWRlbmNlX25lZ1ssMl0pICU+JSANCiAga2FibGUoImh0bWwiLCBkaWdpdHMgPSAzKSAlPiUNCiAga2FibGVfc3R5bGluZygic3RyaXBlZCIsIHBvc2l0aW9uID0gImxlZnQiKQ0KYGBgDQoNCiMjIFB1YmxpY2F0aW9uIEJpYXMgQW5hbHlzaXMNCg0KV2UgZm9sbG93ZWQgYSByZWNlbnRseSBwcm9wb3NlZCBtZXRob2QgYnkgQG5ha2FnYXdhX21ldGhvZHNfMjAyMSwgd2hpY2ggaW52b2x2ZWQgY29uZHVjdGluZyAzIHB1YmxpY2F0aW9uIGJpYXMgbW9kZWxzOiAxKSBNZXRhLXJlZ3Jlc3Npb24gd2l0aCBTRSAodW5pLW1vZGVyYXRvciksIDIpIG1ldGEtcmVncmVzc2lvbiB3aXRoIHllYXIgb2YgcHVibGljYXRpb24gKHVuaS1tb2RlcmF0b3IpLCBhbmQgMykgYWxsLWluIHB1YmxpY2F0aW9uIGJpYXMgdGVzdCAobXVsdGktbW9kZXJhdG9yKS4gV2UgcmFuIHRoZXNlIHB1YmxpY2F0aW9uIGJpYXMgbW9kZWxzIHVzaW5nIHRoZSBkYXRhc2V0IHRoYXQgaGFkIG5lZ2F0aXZlIHJlcGVhdGFiaWxpdHkgdmFsdWVzIHNldCB0byB6ZXJvLg0KDQojIyMgTWV0YS1yZWdyZXNzaW9uIHdpdGggU0UgKHVuaS1tb2RlcmF0b3IpDQoNClRvIHRlc3QgZm9yIHB1YmxpY2F0aW9uIGJpYXMsIHdlIGZpcnN0IGZpdCBhIHBoeWxvZ2VuZXRpYyBtdWx0aWxldmVsIG1ldGEtcmVncmVzc2lvbiB0byBleHBsb3JlIHdoZXRoZXIgdGhlcmUgaXMgc29tZSBldmlkZW5jZSBvZiBzbWFsbC1zdHVkeSBlZmZlY3RzIGluIG91ciBtZXRhLWFuYWx5dGljIGRhdGFzZXQuIFRvIGRvIHNvLCB3ZSBmaXQgYSB1bmktbW9kZXJhdG9yIHBoeWxvZ2VuZXRpYyBtdWx0aWxldmVsIG1ldGEtcmVncmVzc2lvbiBpbmNsdWRpbmcgdGhlIGVmZmVjdCBzaXplc+KAmSBzdGFuZGFyZCBlcnJvcnMgKHNlaSkgYXMgdGhlIG9ubHkgbW9kZXJhdG9yLg0KDQpgYGB7ciBzbWFsbC1zdHVkeS1tb2RlbH0NCiMgY3JlYXRpbmcgYSB2YXJpYWJsZSBmb3IgdGhlIHN0YW5kYXJkIGVycm9yIG9mIGVhY2ggZWZmZWN0IHNpemUgKGkuZS4gdGhlIHNxdWFyZSByb290IG9mIHRoZSBzYW1wbGluZyB2YXJpYW5jZSkNCmRmJHNlaSA8LSBzcXJ0KGRmJFZacikNCg0KIyBBcHBsaWNhdGlvbiBvZiBFcXVhdGlvbiAyMSBmcm9tIHRoZSBtYWluIHRleHQgaW4gTmFrYWdhd2EgZXQgYWwuIDIwMjENCnB1YmxpY2F0aW9uLmJpYXMubW9kZWwuci5zZSA8LSBybWEubXYoeWkgPSBacjIsIFYgPSBWQ1YsDQogICAgICAgICAgICAgICAgICAgICAgICAgICAgICAgICAgICAgIG1vZCA9IH4xICsgc2VpLA0KICAgICAgICAgICAgICAgICAgICAgICAgICAgICAgICAgICAgICByYW5kb20gPSBsaXN0KH4xIHwgZXNfSUQsIA0KICAgICAgICAgICAgICAgICAgICAgICAgICAgICAgICAgICAgICAgICAgICAgICAgICAgIH4xIHwgcGFwZXJfSUQsIA0KICAgICAgICAgICAgICAgICAgICAgICAgICAgICAgICAgICAgICAgICAgICAgICAgICAgIH4xIHwgY29ob3J0X0lELCANCiAgICAgICAgICAgICAgICAgICAgICAgICAgICAgICAgICAgICAgICAgICAgICAgICAgICB+MSB8IHNwZWNpZXNfSUQsIA0KICAgICAgICAgICAgICAgICAgICAgICAgICAgICAgICAgICAgICAgICAgICAgICAgICAgIH4xIHwgcGh5bG9nZW55KSwNCiAgICAgICAgICAgICAgICAgICAgICAgICAgICAgICAgICAgICAgUiA9IGxpc3QocGh5bG9nZW55ID0gdmFyY29yKSwgIyBhZGRlZCBpbiBwaHlsb2duZXkNCiAgICAgICAgICAgICAgICAgICAgICAgICAgICAgICAgICAgICAgZGF0YT1kZikNCg0KIyBwcmludChwdWJsaWNhdGlvbi5iaWFzLm1vZGVsLnIuc2UsZGlnaXRzPTMpDQoNCmBgYA0KDQojIyMjIFRhYmxlIFMyMA0KUmVncmVzc2lvbiBjb2VmZmljaWVudHMgKEVzdGltYXRlKSBhbmQgOTUlIGNvbmZpZGVuY2UgaW50ZXJ2YWxzIChDSXMpIGZyb20gdGhlIHVuaXZhcmlhdGUgbWV0YS1yZWdyZXNzaW9uIGZpdHRlZCB3aXRoIGBzZWlgLg0KDQpgYGB7ciBzbWFsbC1zdHVkeS10YWJsZX0NCg0KIyBjcmVhdGluZyBhIHRhYmxlDQp0X3NlaSA8LSB0aWJibGUoDQogIGBGaXhlZCBlZmZlY3RgID0gYygiSW50ZXJjZXB0IiwgInNlaSIpLA0KICBgRXN0aW1hdGVgID0gYyhwdWJsaWNhdGlvbi5iaWFzLm1vZGVsLnIuc2UkYiksDQogIGBMb3dlciBDSSBbMC4wMjVdYCA9IGMocHVibGljYXRpb24uYmlhcy5tb2RlbC5yLnNlJGNpLmxiKSwNCiAgYFVwcGVyIENJICBbMC45NzVdYCA9IGMocHVibGljYXRpb24uYmlhcy5tb2RlbC5yLnNlJGNpLnViKSkNCiMgIGBSMmAgPSBjKG9yY2hhUmQ6OnIyX21sKHB1YmxpY2F0aW9uLmJpYXMubW9kZWwuci5zZSlbWzFdXSwgcmVwKE5BLDEpKSkNCg0KdF9zZWkgJT4lIGthYmxlKCJodG1sIiwgZGlnaXRzID0gMykgJT4lDQogIGthYmxlX3N0eWxpbmcoInN0cmlwZWQiLCBwb3NpdGlvbiA9ICJsZWZ0IikgDQoNCmBgYA0KDQpBY2NvcmRpbmcgdG8gdGhpcyB1bmktbW9kZXJhdG9yIG1ldGEtcmVncmVzc2lvbiwgdGhlcmUgaXMgbm8gZXZpZGVuY2Ugb2Ygc21hbGwtc3R1ZHkgZWZmZWN0cyBzaW5jZSB0aGUgc2xvcGUgb2YgdGhlIG1vZGVyYXRvciDigJhzZWnigJkgaXMgbm90IHN0YXRpc3RpY2FsbHkgc2lnbmlmaWNhbnQgKHNsb3BlID0gYHIgcm91bmQocHVibGljYXRpb24uYmlhcy5tb2RlbC5yLnNlJGJbMiwxXSwzKWAsIDk1JSBDSSA9IFtgciByb3VuZChwdWJsaWNhdGlvbi5iaWFzLm1vZGVsLnIuc2UkY2kubGJbMl0sMylgLCBgciByb3VuZChwdWJsaWNhdGlvbi5iaWFzLm1vZGVsLnIuc2UkY2kudWJbMl0sMylgXTsgKlI8c3VwPjI8L3N1cD48c3ViPm1hcmdpbmFsPC9zdWI+KiA9IGByIHJvdW5kKG9yY2hhUmQ6OnIyX21sKHB1YmxpY2F0aW9uLmJpYXMubW9kZWwuci5zZSlbWzFdXSoxMDAsMSlgJSksIHNob3dpbmcgdGhhdCBlZmZlY3Qgc2l6ZXMgd2l0aCBsYXJnZXIgU0UgKG1vcmUgdW5jZXJ0YWluIGVmZmVjdCBzaXplcykgZG8gbm90IHRlbmQgdG8gYmUgbGFyZ2VyLiBCdXQgd2Ugd2lsbCBjb25maXJtIHRoaXMgYWZ0ZXIgYWNjb3VudGluZyBmb3Igc29tZSBvZiB0aGUgaGV0ZXJvZ2VuZWl0eSBwcmVzZW50IGluIHRoZSBkYXRhIHVzaW5nIHRoZSBhbGwtaW4gcHVibGljYXRpb24gYmlhcyB0ZXN0IChtdWx0aS1tb2RlcmF0b3IgbWV0YS1yZWdyZXNzaW9uKS4NCg0KIyMjIE1ldGEtcmVncmVzc2lvbiB3aXRoIHllYXIgb2YgcHVibGljYXRpb24gKHVuaS1tb2RlcmF0b3IpDQoNClRvIHRlc3QgZm9yIHRpbWUtbGFnIGJpYXMgKGFsc28gY2FsbGVkIGRlY2xpbmUgZWZmZWN0cykgd2UgY2FuIGZpcnN0IGZpdCBhIHVuaS1tb2RlcmF0b3IgcGh5bG9nZW5ldGljIG11bHRpbGV2ZWwgbWV0YS1yZWdyZXNzaW9uIGluY2x1ZGluZyB0aGUgeWVhciBvZiBwdWJsaWNhdGlvbiAobWVhbi1jZW50cmVkKSBhcyB0aGUgb25seSBtb2RlcmF0b3IuDQoNCmBgYHtyIHB1YmxpY2F0aW9uLXllYXItbW9kZWx9DQoNCmRmJHB1Yl95ZWFyLmMgPC0gYXMudmVjdG9yKHNjYWxlKGRmJHB1Yl95ZWFyLCBzY2FsZSA9IEYpKQ0KDQojIEFwcGxpY2F0aW9uIG9mIEVxdWF0aW9uIDIzIGZyb20gdGhlIG1haW4gdGV4dCBpbiBOYWthZ2F3YSBldCBhbC4gMjAyMQ0KcHVibGljYXRpb24uYmlhcy5tb2RlbC5yLnRpbWVsYWcgPC0gcm1hLm12KHlpID0gWnIyLCBWID0gVkNWLA0KICAgICAgICAgICAgICAgICAgICAgICAgICAgICAgICAgICAgICAgICAgIG1vZHM9IH4xICsgcHViX3llYXIuYywNCiAgICAgICAgICAgICAgICAgICAgICAgICAgICAgICAgICAgICAgICAgICByYW5kb20gPSBsaXN0KH4xIHwgZXNfSUQsIA0KICAgICAgICAgICAgICAgICAgICAgICAgICAgICAgICAgICAgICAgICAgICAgICAgICAgICAgICAgfjEgfCBwYXBlcl9JRCwgDQogICAgICAgICAgICAgICAgICAgICAgICAgICAgICAgICAgICAgICAgICAgICAgICAgICAgICAgICB+MSB8IGNvaG9ydF9JRCwgDQogICAgICAgICAgICAgICAgICAgICAgICAgICAgICAgICAgICAgICAgICAgICAgICAgICAgICAgICB+MSB8IHNwZWNpZXNfSUQsIA0KICAgICAgICAgICAgICAgICAgICAgICAgICAgICAgICAgICAgICAgICAgICAgICAgICAgICAgICAgfjEgfCBwaHlsb2dlbnkpLA0KICAgICAgICAgICAgICAgICAgICAgICAgICAgICAgICAgICAgICAgICAgIFIgPSBsaXN0KHBoeWxvZ2VueSA9IHZhcmNvciksICMgYWRkZWQgaW4gcGh5bG9nbmV5DQogICAgICAgICAgICAgICAgICAgICAgICAgICAgICAgICAgICAgICAgICAgZGF0YT1kZikNCg0KYGBgDQoNCiMjIyMgVGFibGUgUzIxDQpSZWdyZXNzaW9uIGNvZWZmaWNpZW50cyAoRXN0aW1hdGUpIGFuZCA5NSUgY29uZmlkZW5jZSBpbnRlcnZhbHMgKENJcykgZnJvbSB0aGUgdW5pdmFyaWF0ZSBtZXRhLXJlZ3Jlc3Npb24gZml0dGVkIHdpdGggcHVibGljYXRpb24geWVhci4NCg0KYGBge3IgcHVibGljYXRpb24teWVhci10YWJsZX0NCg0KIyBjcmVhdGluZyBhIHRhYmxlDQp0X3B1Yl95ZWFyIDwtIHRpYmJsZSgNCiAgYEZpeGVkIGVmZmVjdGAgPSBjKCJJbnRlcmNlcHQiLCAicHViX3llYXIuYyIpLA0KICBgRXN0aW1hdGVgID0gYyhwdWJsaWNhdGlvbi5iaWFzLm1vZGVsLnIudGltZWxhZyRiKSwNCiAgYExvd2VyIENJIFswLjAyNV1gID0gYyhwdWJsaWNhdGlvbi5iaWFzLm1vZGVsLnIudGltZWxhZyRjaS5sYiksDQogIGBVcHBlciBDSSAgWzAuOTc1XWAgPSBjKHB1YmxpY2F0aW9uLmJpYXMubW9kZWwuci50aW1lbGFnJGNpLnViKSkNCiMgIGBSMmAgPSBjKG9yY2hhUmQ6OnIyX21sKHB1YmxpY2F0aW9uLmJpYXMubW9kZWwuci50aW1lbGFnKVtbMV1dLCByZXAoTkEsMSkpKQ0KDQp0X3B1Yl95ZWFyICU+JSBrYWJsZSgiaHRtbCIsIGRpZ2l0cyA9IDMpICU+JQ0KICBrYWJsZV9zdHlsaW5nKCJzdHJpcGVkIiwgcG9zaXRpb24gPSAibGVmdCIpIA0KDQpgYGANCg0KQWNjb3JkaW5nIHRvIHRoaXMgdW5pLW1vZGVyYXRvciBtZXRhLXJlZ3Jlc3Npb24sIHRoZXJlIGlzIG5vIGRlY2xpbmUgZWZmZWN0cyBzaW5jZSB0aGUgc2xvcGUgb2YgdGhlIG1vZGVyYXRvciDigJh5ZWFyIG9mIHB1YmxpY2F0aW9u4oCZIGlzIGVzc2VudGlhbGx5IHplcm8gKHNsb3BlID0gYHIgcm91bmQocHVibGljYXRpb24uYmlhcy5tb2RlbC5yLnRpbWVsYWckYlsyLDFdLDMpYCwgOTUlIENJID0gW2ByIHJvdW5kKHB1YmxpY2F0aW9uLmJpYXMubW9kZWwuci50aW1lbGFnJGNpLmxiWzJdLDMpYCwgYHIgcm91bmQocHVibGljYXRpb24uYmlhcy5tb2RlbC5yLnRpbWVsYWckY2kudWJbMl0sMylgXTsgKlI8c3VwPjI8L3N1cD48c3ViPm1hcmdpbmFsPC9zdWI+KiA9IGByIHJvdW5kKG9yY2hhUmQ6OnIyX21sKHB1YmxpY2F0aW9uLmJpYXMubW9kZWwuci50aW1lbGFnKVtbMV1dKjEwMCwxKWAlKSwgc2hvd2luZyB0aGF0IGVmZmVjdCBzaXplcyBoYXZlIG5vdCBjaGFuZ2VkIGxpbmVhcmx5IG92ZXIgdGltZSBzaW5jZSB0aGUgZmlyc3QgZWZmZWN0IHNpemUgd2FzIHB1Ymxpc2hlZC4gQnV0IGFnYWluLCB3ZSBuZWVkIHRvIGNvbmZpcm0gdGhpcyBwYXR0ZXJuIGFmdGVyIGFjY291bnRpbmcgZm9yIHNvbWUgb2YgdGhlIGhldGVyb2dlbmVpdHkgcHJlc2VudCBpbiB0aGUgZGF0YSB1c2luZyB0aGUgYWxsLWluIHB1YmxpY2F0aW9uIGJpYXMgdGVzdC4NCg0KDQojIyMgQWxsLWluIHB1YmxpY2F0aW9uIGJpYXMgdGVzdCAobXVsdGktbW9kZXJhdG9yKQ0KDQpXaGVuIGhldGVyb2dlbmVpdHkgZXhpc3RzICh3aGljaCBpcyBub3JtYWxseSB0aGUgY2FzZSBpbiBlY29sb2d5IGFuZCBldm9sdXRpb247IFNlbmlvciBldCBhbC4gMjAxNiksIGl0IGlzIGJlc3QgdG8gY29tYmluZSB0aGUgYWJvdmUgdHdvIG1vZGVscyB3aXRoIG90aGVyIG1vZGVyYXRvcnMgc2luY2UgdGhvc2UgYWRkaXRpb25hbCBtb2RlcmF0b3JzIHdpbGwgZ2VuZXJhbGx5IGJlIGV4cGVjdGVkIHRvIGV4cGxhaW4gc29tZSBvZiB0aGUgaGV0ZXJvZ2VuZWl0eS4gVGhhdCBpcywgdGhpcyBhbGwtaW4gcHVibGljYXRpb24gYmlhcyB0ZXN0IChtdWx0aS1tb2RlcmF0b3IgbWV0YS1yZWdyZXNzaW9uKSB3b3VsZCBiZSB0aGUgYmVzdCB0ZXN0IG9mIHNtYWxsLXN0dWR5IChwdWJsaWNhdGlvbiBiaWFzKSBhbmQgZGVjbGluZSBlZmZlY3RzICh0aW1lLWxhZyBiaWFzKSBpbiBtb3N0IG1ldGEtYW5hbHl0aWMgZGF0YXNldHMgW3NlZSBAbmFrYWdhd2FfbWV0aG9kc18yMDIxXS4gRm9yIG91ciBkYXRhLCB3ZSB3aWxsIHJ1biBhIG11bHRpLW1vZGVyYXRvciBwaHlsb2dlbmV0aWMgbXVsdGlsZXZlbCBtZXRhLXJlZ3Jlc3Npb24gaW5jbHVkaW5nIHRoZSBlZmZlY3Qgc2l6ZXPigJkgc3RhbmRhcmQgZXJyb3JzLCB0aGUgeWVhciBvZiBwdWJsaWNhdGlvbiAobWVhbi1jZW50cmVkKSBhbmQgdGhlIDUgbW9kZXJhdG9ycyBpbmNsdWRlZCBpbiBwcmV2aW91cyBtb2RlbHMuDQoNCmBgYHtyIGFsbC1pbi1wdWJsaWNhdGlvbi1tb2RlbH0NCg0KcHVibGljYXRpb24uYmlhcy5tb2RlbC5yLmFsbC5zZSA8LSBybWEubXYoeWkgPSBacjIsIFYgPSBWQ1YsDQogICAgICAgICAgICAgICAgICAgICAgICAgICAgICAgICAgICAgICAgIG1vZHM9IH4xICsgIyAtMSByZW1vdmVzIHRoZSBpbnRlcmNlcHQNCiAgICAgICAgICAgICAgICAgICAgICAgICAgICAgICAgICAgICAgICAgICBzZWkgKw0KICAgICAgICAgICAgICAgICAgICAgICAgICAgICAgICAgICAgICAgICAgIHB1Yl95ZWFyLmMgKw0KICAgICAgICAgICAgICAgICAgICAgICAgICAgICAgICAgICAgICAgICBhbm51YWxfZXZlbnQgKyBtZXRob2QgKyB0YXhhICsgc2V4ICsgaywgDQogICAgICAgICAgICAgICAgICAgICAgICAgICAgICAgICAgICAgICAgIHJhbmRvbSA9IGxpc3QofjEgfCBlc19JRCwgDQogICAgICAgICAgICAgICAgICAgICAgICAgICAgICAgICAgICAgICAgICAgICAgICAgICAgICAgfjEgfCBwYXBlcl9JRCwgDQogICAgICAgICAgICAgICAgICAgICAgICAgICAgICAgICAgICAgICAgICAgICAgICAgICAgICAgfjEgfCBjb2hvcnRfSUQsIA0KICAgICAgICAgICAgICAgICAgICAgICAgICAgICAgICAgICAgICAgICAgICAgICAgICAgICAgIH4xIHwgc3BlY2llc19JRCwgDQogICAgICAgICAgICAgICAgICAgICAgICAgICAgICAgICAgICAgICAgICAgICAgICAgICAgICAgfjEgfCBwaHlsb2dlbnkpLA0KICAgICAgICAgICAgICAgICAgICAgICAgICAgICAgICAgICAgICAgICBSID0gbGlzdChwaHlsb2dlbnkgPSB2YXJjb3IpLCAjIGFkZGVkIGluIHBoeWxvZ25leQ0KICAgICAgICAgICAgICAgICAgICAgICAgICAgICAgICAgICAgICAgICBkYXRhPWRmKQ0KDQpgYGANCg0KIyMjIyBUYWJsZSBTMjINClJlZ3Jlc3Npb24gY29lZmZpY2llbnRzIChFc3RpbWF0ZSkgYW5kIDk1JSBjb25maWRlbmNlIGludGVydmFscyAoQ0lzKSBmcm9tIHRoZSBtdWx0aXZhcmlhdGUgbWV0YS1yZWdyZXNzaW9uIHdpdGggc2VpLCBwdWJsaWNhdGlvbiB5ZWFyLCBhbmQgdGhlIGZpdmUgbW9kZXJhdG9yIHZhcmlhYmxlcy4gKlI8c3VwPjI8L3N1cD48c3ViPm1hcmdpbmFsPC9zdWI+KiA9IGByIHJvdW5kKG9yY2hhUmQ6OnIyX21sKHB1YmxpY2F0aW9uLmJpYXMubW9kZWwuci5hbGwuc2UpW1sxXV0qMTAwLDEpYCUuDQoNCmBgYHtyIGFsbC1pbi1wdWJsaWNhdGlvbi10YWJsZX0NCg0KIyBjcmVhdGluZyBhIHRhYmxlDQp0X2FsbF9pbiA8LSB0aWJibGUoDQogIGBGaXhlZCBlZmZlY3RgID0gYygiSW50ZXJjZXB0IiwgInNlaSIsICJwdWJfeWVhci5jIiwgIkRlcGFydF9icmVlZCIsICJOb25icmVlZF9hcnJpdmFsIiwgIk5vbmJyZWVkX2RlcGFydCIsICJHTFMiLCAiU2F0ZWxsaXRlIiwgIlNlYWJpcmQiLCAiV2F0ZXJiaXJkIiwgIkZlbWFsZSIsICJNYWxlIiwgImsiKSwNCiAgYEVzdGltYXRlYCA9IGMocHVibGljYXRpb24uYmlhcy5tb2RlbC5yLmFsbC5zZSRiKSwNCiAgYExvd2VyIENJIFswLjAyNV1gID0gYyhwdWJsaWNhdGlvbi5iaWFzLm1vZGVsLnIuYWxsLnNlJGNpLmxiKSwNCiAgYFVwcGVyIENJICBbMC45NzVdYCA9IGMocHVibGljYXRpb24uYmlhcy5tb2RlbC5yLmFsbC5zZSRjaS51YikpDQogIyBgUjJgID0gYyhyMl9wdWJsaWNhdGlvbi5iaWFzLm1vZGVsLnIuYWxsLnNlWzFdLCByZXAoTkEsMTIpKSkNCg0KdF9hbGxfaW4gJT4lIGthYmxlKCJodG1sIiwgZGlnaXRzID0gMykgJT4lDQogIGthYmxlX3N0eWxpbmcoInN0cmlwZWQiLCBwb3NpdGlvbiA9ICJsZWZ0IikgDQpgYGANCg0KVGhlIGFsbC1pbiBwdWJsaWNhdGlvbiBiaWFzIHRlc3QgYWdyZWVzIHdpdGggd2hhdCB3ZSBvYnNlcnZlZCBpbiB0aGUgdW5pLW1vZGVyYXRvciBtZXRhLXJlZ3Jlc3Npb25zIGFib3ZlLiBGaXJzdCwgdGhlIG11bHRpLW1vZGVyYXRvciBtZXRhLXJlZ3Jlc3Npb24gc2hvd3Mgbm8gc2lnbmlmaWNhbnQgc2xvcGUgZm9yIHRoZSBtb2RlcmF0b3Ig4oCYc2Vp4oCZIChzbG9wZSA9IGByIHJvdW5kKHB1YmxpY2F0aW9uLmJpYXMubW9kZWwuci5hbGwuc2UkYlsyLDFdLDMpYCwgOTUlIENJID0gW2ByIHJvdW5kKHB1YmxpY2F0aW9uLmJpYXMubW9kZWwuci5hbGwuc2UkY2kubGJbMl0sMylgLGByIHJvdW5kKHB1YmxpY2F0aW9uLmJpYXMubW9kZWwuci5hbGwuc2UkY2kudWJbMl0sMylgXTsgRmlnLiBTNSksIHNob3dpbmcgbm8gZXZpZGVuY2Ugb2Ygc21hbGwtc3R1ZHkgZWZmZWN0cy4gSW4gb3RoZXIgd29yZHMsIHRoZSBsYXJnZXN0IGVmZmVjdCBzaXplcyBpbiB0aGUgZGF0YXNldCBkbyBub3QgdGVuZCB0byBiZSB0aG9zZSB3aXRoIHRoZSBsb3dlc3QgcHJlY2lzaW9uIChpLmUuIGxhcmdlciB1bmNlcnRhaW50eSkuIFNlY29uZCwgdGhlIGFsbC1pbiBwdWJsaWNhdGlvbiBiaWFzIHRlc3QgYWxzbyBjb25maXJtcyB0aGF0IHRoZXJlIGlzIG5vIGV2aWRlbmNlIG9mIGRlY2xpbmUgZWZmZWN0cyBpbiB0aGUgZGF0YSBzaW5jZSB0aGUgc2xvcGUgb2YgdGhlIG1vZGVyYXRvciDigJh5ZWFyIG9mIHB1YmxpY2F0aW9u4oCZIHdhcyBhZ2FpbiBpbmRpc3Rpbmd1aXNoYWJsZSBmcm9tIHplcm8gKHNsb3BlID0gYHIgcm91bmQocHVibGljYXRpb24uYmlhcy5tb2RlbC5yLmFsbC5zZSRiWzMsMV0sMylgLCA5NSUgQ0kgPSBbYHIgcm91bmQocHVibGljYXRpb24uYmlhcy5tb2RlbC5yLmFsbC5zZSRjaS5sYlszXSwzKWAsYHIgcm91bmQocHVibGljYXRpb24uYmlhcy5tb2RlbC5yLmFsbC5zZSRjaS51YlszXSwzKWBdOyBGaWcuIFM2KS4NCg0KIyMjIyBGaWd1cmUgUzUNCg0KYGBge3IgZmlndXJlUzV9DQoNCnByZWRpY3QucHVibGljYXRpb24uYmlhcy5tb2RlbC5yLmFsbC5zZS5wbG90LjEgPC0gcHJlZGljdChwdWJsaWNhdGlvbi5iaWFzLm1vZGVsLnIuYWxsLnNlLA0KICAgICAgICAgICAgICAgICAgICAgICAgICAgICAgICAgICAgICAgICAgICAgICAgICAgICAgICAgIG5ld21vZHM9Y2JpbmQoc2VxKG1pbihkZiRzZWkpLA0KICAgICAgICAgICAgICAgICAgICAgICAgICAgICAgICAgICAgICAgICAgICAgICAgICAgICAgICAgICAgICAgICAgICAgICAgICAgIG1heChkZiRzZWkpLA0KICAgICAgICAgICAgICAgICAgICAgICAgICAgICAgICAgICAgICAgICAgICAgICAgICAgICAgICAgICAgICAgICAgICAgICAgICAgIDAuMDA1KSwNCiAgICAgICAgICAgICAgICAgICAgICAgICAgICAgICAgICAgICAgICAgICAgICAgICAgICAgICAgICAgICAgICAgICAgICAgIGMoMCksIDAsIDAsIDAsIDAsIDAsIDAsIDAsIDAsIDAsIDApKQ0KDQpuZXdkYXQgPC0gZGF0YS5mcmFtZShzZWk9c2VxKG1pbihkZiRzZWkpLA0KICAgICAgICAgICAgICAgICAgICAgICAgICAgICBtYXgoZGYkc2VpKSwNCiAgICAgICAgICAgICAgICAgICAgICAgICAgICAgMC4wMDUpLA0KICAgICAgICAgICAgICAgICAgICAgZml0PXByZWRpY3QucHVibGljYXRpb24uYmlhcy5tb2RlbC5yLmFsbC5zZS5wbG90LjEkcHJlZCwNCiAgICAgICAgICAgICAgICAgICAgIHVwcGVyPXByZWRpY3QucHVibGljYXRpb24uYmlhcy5tb2RlbC5yLmFsbC5zZS5wbG90LjEkY2kudWIsDQogICAgICAgICAgICAgICAgICAgICBsb3dlcj1wcmVkaWN0LnB1YmxpY2F0aW9uLmJpYXMubW9kZWwuci5hbGwuc2UucGxvdC4xJGNpLmxiLA0KICAgICAgICAgICAgICAgICAgICAgc3RyaW5nc0FzRmFjdG9ycz1GQUxTRSkNCg0KZ2dwbG90KGRhdGEgPSBkZiwgYWVzKHggPSBzZWksIHkgPSBacjIpKSArDQogIGdlb21fcG9pbnQoc2hhcGUgPSAyMSwgZmlsbCA9ICJncmV5ODUiLCBjb2xvdXI9ImdyZXk2MCIsIHNpemU9MywgYWxwaGE9MC41KSArDQogIGdlb21faGxpbmUoeWludGVyY2VwdCA9IDAsbGluZXR5cGUgPSAyLCBjb2xvdXIgPSAiYmxhY2siLGFscGhhPTAuNSkgKw0KICBnZW9tX2xpbmUoZGF0YT1uZXdkYXQsIGFlcyh4PXNlaSwgeT1maXQpLCBzaXplID0gMS41LCBjb2xvdXI9ImRhcmtvcmNoaWQ0IikgKw0KICBnZW9tX3JpYmJvbihkYXRhPW5ld2RhdCwgYWVzKHltaW4gPSBsb3dlciwgeW1heCA9IHVwcGVyLCB5PTApLCBhbHBoYSA9IC4zLCBmaWxsPSJkYXJrb3JjaGlkNCIpICsNCiAgbGFicyh4PSJTdGFuZGFyZCBlcnJvciAoc2VpKSIsIHk9IkVmZmVjdCBzaXplIChacikiKSArDQogIHNjYWxlX3lfY29udGludW91cyhsaW1pdHM9YygtMSwgMi41KSwgYnJlYWtzID0gc2VxKC0xLCAyLjIsIGJ5ID0gMSkpICsNCiAgdGhlbWVfYncoKSArDQogIHRoZW1lKHRleHQgPSBlbGVtZW50X3RleHQoc2l6ZSA9IDksIGNvbG91ciA9ICJibGFjayIsIGhqdXN0ID0gMC41KSwNCiAgICAgICAgcGFuZWwuZ3JpZCA9IGVsZW1lbnRfYmxhbmsoKSkNCg0KYGBgDQogIA0KICAqKkZpZ3VyZSBTNS4qKiBBIGJ1YmJsZSBwbG90IHNob3dpbmcgdGhhdCBlZmZlY3Qgc2l6ZXMgd2l0aCBsYXJnZXIgc3RhbmRhcmQgZXJyb3JzIGRvIG5vdCB0ZW5kIHRvIGJlIGxhcmdlciwgcHJvdmlkaW5nIG5vIGV2aWRlbmNlIG9mIHNtYWxsLXN0dWR5IGVmZmVjdHMgaW4gdGhlIG1ldGEtYW5hbHl0aWMgZGF0YXNldC4gVGhlIHNvbGlkIGxpbmUgcmVwcmVzZW50cyB0aGUgbW9kZWwgZXN0aW1hdGUgYW5kIHRoZSBzaGFkaW5nIHNob3dzIGl0cyA5NSUgY29uZmlkZW5jZSBpbnRlcnZhbHMuDQoNCiMjIyMgRmlndXJlIFM2DQoNCmBgYHtyIGZpZ3VyZVM2fQ0KDQpwcmVkaWN0LnB1YmxpY2F0aW9uLmJpYXMubW9kZWwuci5hbGwuc2UucGxvdC4yIDwtIHByZWRpY3QocHVibGljYXRpb24uYmlhcy5tb2RlbC5yLmFsbC5zZSwNCiAgICAgICAgICAgICAgICAgICAgICAgICAgICAgICAgICAgICAgICAgICAgICAgICAgICAgICAgICBuZXdtb2RzPWNiaW5kKG1lYW4oZGYkc2VpKSwNCiAgICAgICAgICAgICAgICAgICAgICAgICAgICAgICAgICAgICAgICAgICAgICAgICAgICAgICAgICAgICAgICAgICAgICAgIHNlcShtaW4oZGYkcHViX3llYXIuYyksDQogICAgICAgICAgICAgICAgICAgICAgICAgICAgICAgICAgICAgICAgICAgICAgICAgICAgICAgICAgICAgICAgICAgICAgICAgICAgbWF4KGRmJHB1Yl95ZWFyLmMpLA0KICAgICAgICAgICAgICAgICAgICAgICAgICAgICAgICAgICAgICAgICAgICAgICAgICAgICAgICAgICAgICAgICAgICAgICAgICAgIDAuMjUpLDAsIDAsMCwwLDAsMCwwLDAsMCwwKSkNCg0KDQpuZXdkYXQyIDwtIGRhdGEuZnJhbWUocHViX3llYXIuYz1zZXEobWluKGRmJHB1Yl95ZWFyLmMpLA0KICAgICAgICAgICAgICAgICAgICAgICAgICAgICAgbWF4KGRmJHB1Yl95ZWFyLmMpLA0KICAgICAgICAgICAgICAgICAgICAgICAgICAgICAgMC4yNSksDQogICAgICAgICAgICAgICAgICAgICBmaXQ9cHJlZGljdC5wdWJsaWNhdGlvbi5iaWFzLm1vZGVsLnIuYWxsLnNlLnBsb3QuMiRwcmVkLA0KICAgICAgICAgICAgICAgICAgICAgdXBwZXI9cHJlZGljdC5wdWJsaWNhdGlvbi5iaWFzLm1vZGVsLnIuYWxsLnNlLnBsb3QuMiRjaS51YiwNCiAgICAgICAgICAgICAgICAgICAgIGxvd2VyPXByZWRpY3QucHVibGljYXRpb24uYmlhcy5tb2RlbC5yLmFsbC5zZS5wbG90LjIkY2kubGIsDQogICAgICAgICAgICAgICAgICAgICBzdHJpbmdzQXNGYWN0b3JzPUZBTFNFKQ0KDQpnZ3Bsb3QoZGF0YSA9IGRmLCBhZXMoeCA9IHB1Yl95ZWFyLmMsIHkgPSBacjIpKSArDQogIGdlb21fcG9pbnQoYWVzKHNpemU9KDEvc3FydChWWnIpKSksIHNoYXBlID0gMjEsIGZpbGwgPSAiZ3JleTg1IiwgY29sb3VyPSJncmV5NjAiLCBhbHBoYT0wLjUpICsNCiAgZ2VvbV9obGluZSh5aW50ZXJjZXB0ID0gMCxsaW5ldHlwZSA9IDIsIGNvbG91ciA9ICJibGFjayIsYWxwaGE9MC41KSArDQogIGdlb21fbGluZShkYXRhPW5ld2RhdDIsIGFlcyh4PXB1Yl95ZWFyLmMsIHk9Zml0KSwgc2l6ZSA9IDEuNSwgY29sb3VyPSJkYXJrb3JjaGlkNCIpICsNCiAgZ2VvbV9yaWJib24oZGF0YT1uZXdkYXQyLCBhZXMoeW1pbiA9IGxvd2VyLCB5bWF4ID0gdXBwZXIsIHk9MCksIGFscGhhID0gLjMsIGZpbGw9ImRhcmtvcmNoaWQ0IikgKw0KICBsYWJzKHg9IlllYXIgb2YgcHVibGljYXRpb24iLCB5PSJFZmZlY3Qgc2l6ZSAoWnIpIiwgc2l6ZSA9ICJQcmVjaXNvbiAoMS9TRSkiKSArDQogIHNjYWxlX3lfY29udGludW91cyhsaW1pdHM9YygtMSwgMi41KSwgYnJlYWtzID0gc2VxKC0xLCAyLjIsIGJ5ID0gMSkpICsNCiAgc2NhbGVfeF9jb250aW51b3VzKGJyZWFrcyA9IGMoLTI0LjA4NjIwNjksIC0xNC4wODYyMDY5LCAtNC4wODYyMDY5LCA1LjkxMzc5MzEpLCBsYWJlbCA9IGMoMTk5MCwyMDAwLCAyMDEwLCAyMDIwKSkgKw0KICB0aGVtZV9idygpICsNCiAgdGhlbWUodGV4dCA9IGVsZW1lbnRfdGV4dChzaXplID0gOSwgY29sb3VyID0gImJsYWNrIiwgaGp1c3QgPSAwLjUpLA0KICAgICAgICBwYW5lbC5ncmlkID0gZWxlbWVudF9ibGFuaygpLA0KICAgICAgICBsZWdlbmQudGV4dD1lbGVtZW50X3RleHQoc2l6ZT04KSwNCiAgICAgICAgICBsZWdlbmQucG9zaXRpb249YygwLDApLCANCiAgICAgICAgICBsZWdlbmQuanVzdGlmaWNhdGlvbiA9IGMoMCwwKSwNCiAgICAgICAgICBsZWdlbmQuYmFja2dyb3VuZCA9IGVsZW1lbnRfYmxhbmsoKSwgDQogICAgICAgICAgbGVnZW5kLmRpcmVjdGlvbj0iaG9yaXpvbnRhbCIsDQogICAgICAgICAgbGVnZW5kLnRpdGxlID0gZWxlbWVudF90ZXh0KHNpemU9OCkpDQoNCmBgYA0KICANCiAgKipGaWd1cmUgUzYuKiogQSBidWJibGUgcGxvdCBzaG93aW5nIHRoYXQgdGhlIG92ZXJhbGwgZWZmZWN0IHNpemUgaGFzIG5vdCBjaGFuZ2VkIG92ZXIgdGltZSwgd2hlcmUgdGhlIHNvbGlkIGxpbmUgcmVwcmVzZW50cyB0aGUgbW9kZWwgZXN0aW1hdGUgYW5kIHRoZSBzaGFkaW5nIHNob3dzIGl0cyA5NSUgY29uZmlkZW5jZSBpbnRlcnZhbHMsIHdpdGggaW5kaXZpZHVhbCBkYXRhIHBvaW50cyBzY2FsZWQgYnkgcHJlY2lzaW9uICgxL1NFKS4NCg0KIyMgQWNrbm93bGVkZ2VtZW50cw0KDQpNYW55IGNvZGluZyBtYXRlcmlhbHMgaGF2ZSBiZWVuIGJvcnJvd2VkIGZyb20gdGhlc2UgcGFwZXJzIFtAaGF5d2FyZF9icm9hZHNjYWxlXzIwMjE7IEBwb3R0aWVyX3NleHVhbF8yMDIxXS4NCg0KIyMgUiBTZXNzaW9uIEluZm9ybWF0aW9uDQoNCmBgYHtyIGVjaG89RkFMU0V9DQojIHBhbmRlciBmb3IgbWFraW5nIGl0IGxvb2sgbmljZXINCnNlc3Npb25JbmZvKCkgJT4lIHBhbmRlcigpDQpgYGANCg0KDQojIyBSZWZlcmVuY2Vz
